# Supplementary material for: 10-year multimorbidity patterns among people with and without rheumatic and musculoskeletal diseases: an observational cohort study using linked electronic health records from Wales, UK
Source: BMJ Open. 2024 Jun 19;14(6):e079169. doi: 10.1136/bmjopen-2023-079169 (PMC11191776; doi:10.1136/bmjopen-2023-079169)
Supplement: Supplementary data [file bmjopen-2023-079169supp002.pdf]

Supplementary

10- year multi-morbidity patterns among people with and without rheumatic and musculoskeletal diseases: An observational cohort study using linked electronic health records from Wales, UK

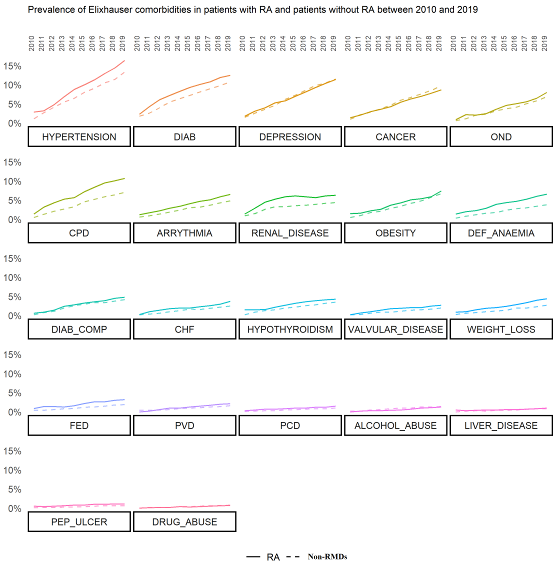

(a) Annual prevalence of Elixhauser co-morbidities in PMR cases and matched comparators 2010-2019

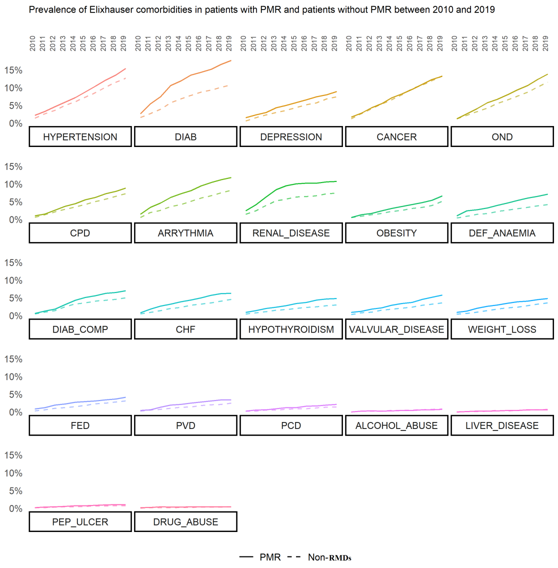

(b) Annual prevalence of Elixhauser co-morbidities in PMR cases and matched comparators 2010-2019

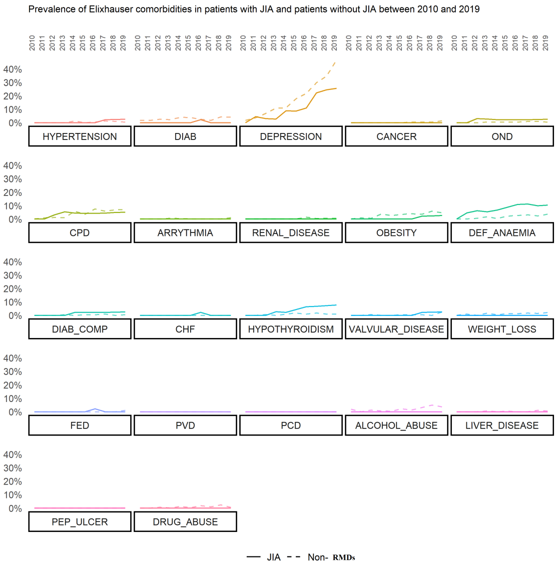

(c) Annual prevalence of Elixhauser co-morbidities in JIA cases and matched comparators 2010-2019

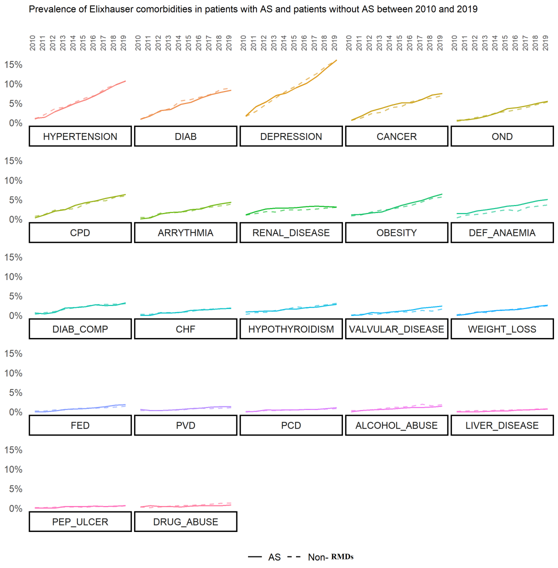

(d) Annual prevalence of Elixhauser co-morbidities in JIA cases and matched comparators 2010-2019

Figure 1. Annual prevalence of Elixhauser co-morbidities in RMDs subtype 2010-2019

The Figures 1 illustrate the annual prevalence for of RMDs subtype: RA(Rheumatoid arthritis), CTD - connective tissue diseases-, PMR (polymyalgia rheumatica and giant cell arteritis cases) and JIA: juvenile arthritis and non RMDs from 2010-2019.

....

The Figures 2 illustrate the age-specific analysis, in relation to specific co-Elixhauser morbidities for RMDs and comparators between 2010-2018.

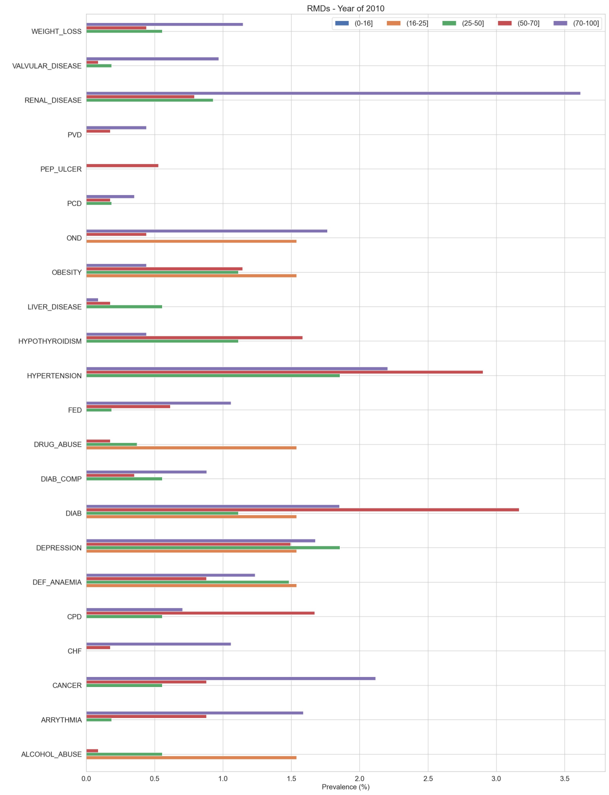

(a) age-specific analysis, especially in relation to specific co-morbidities

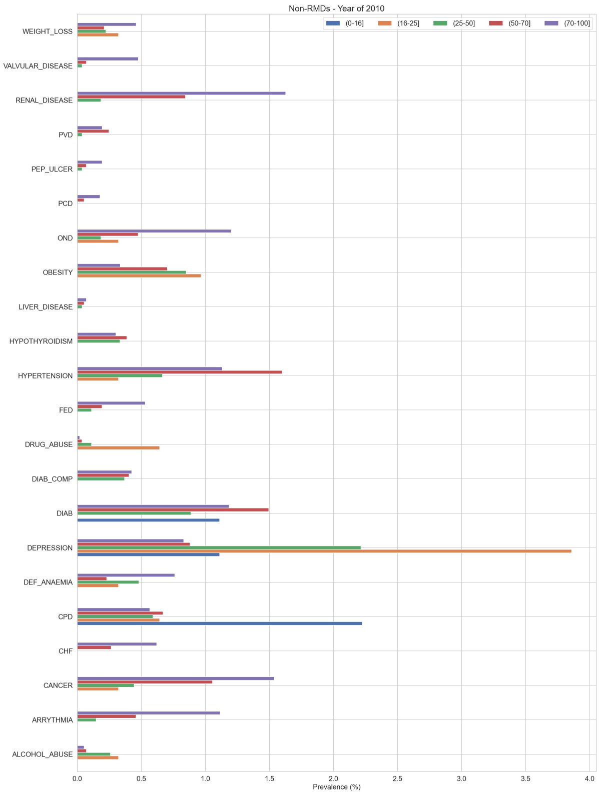

(b) age-specific analysis, especially in relation to specific co-morbidities

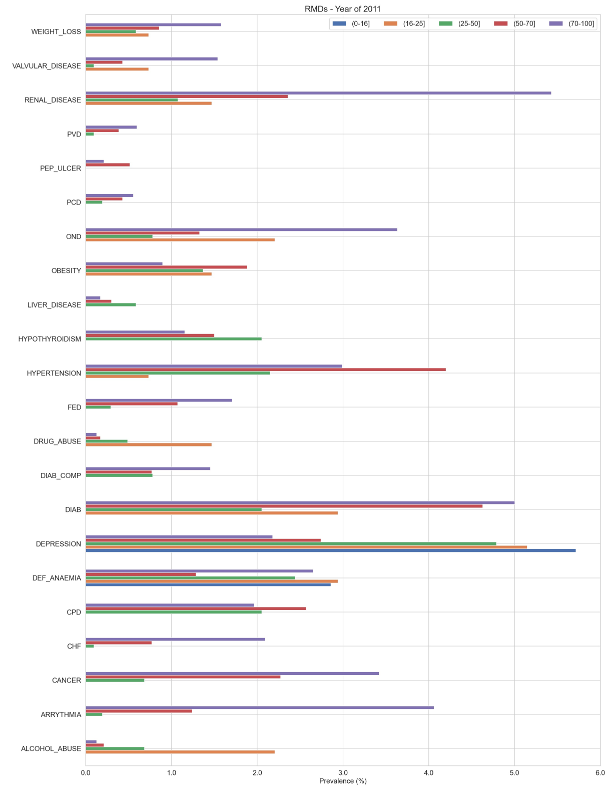

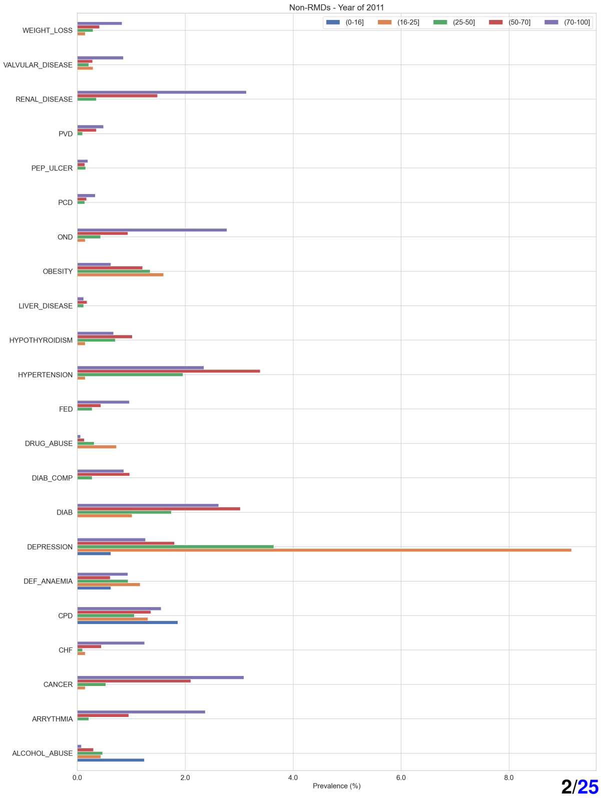

(c) age-specific analysis, especially in relation to specific co-morbidities

(d) age-specific analysis, especially in relation to specific co-morbidities

Figure 2. age-specific analysis, in relation to specific Elixhauser co-morbidities

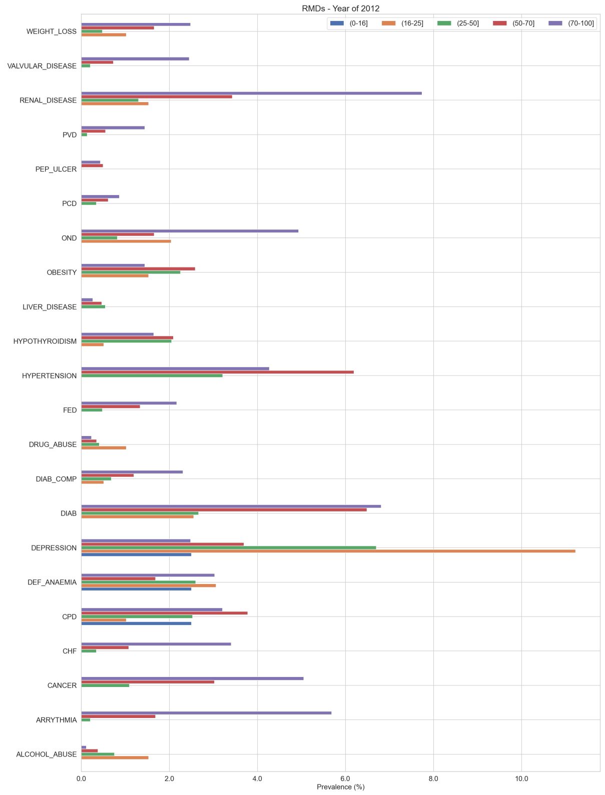

(e) age-specific analysis, especially in relation to specific co-morbidities

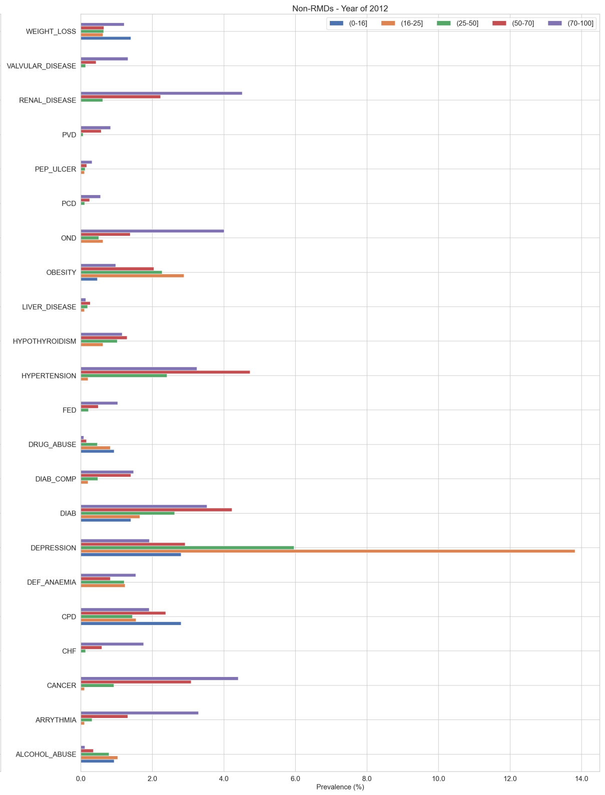

(f) age-specific analysis, especially in relation to specific co-morbidities

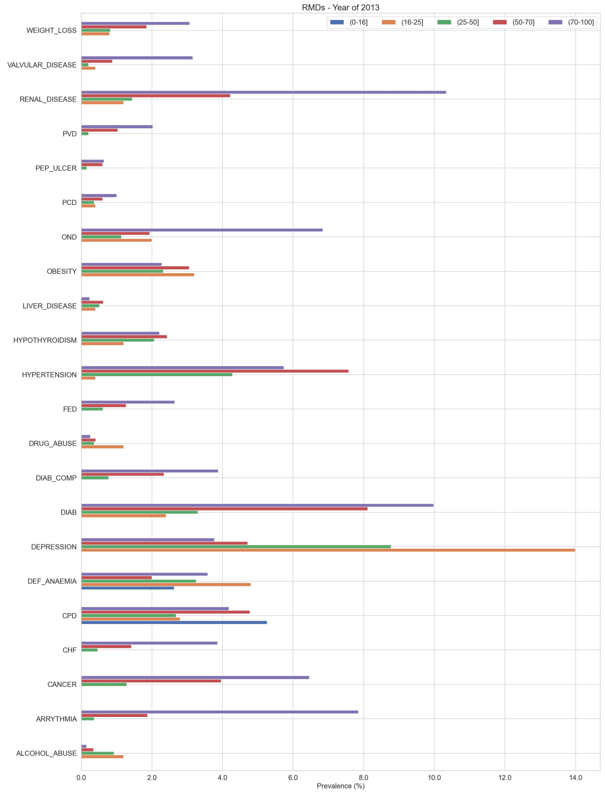

(g) age-specific analysis, especially in relation to specific co-morbidities

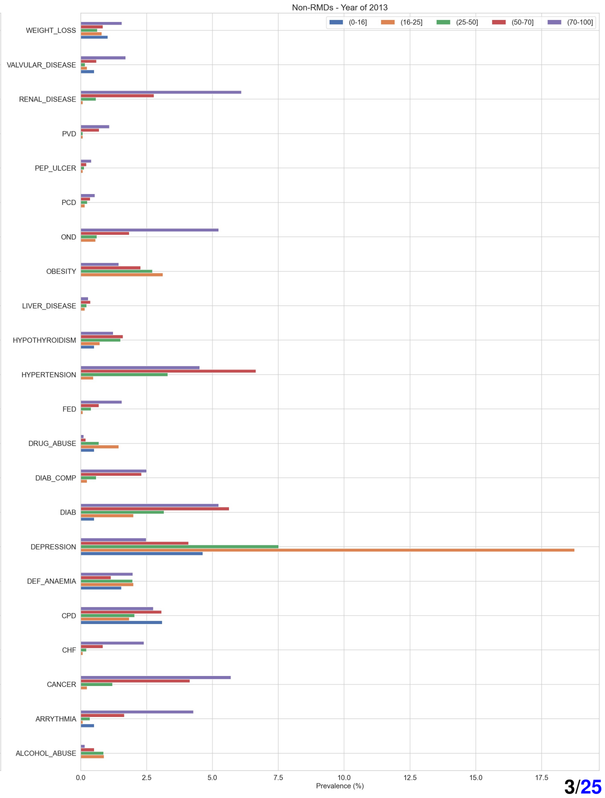

3/25

(h) age-specific analysis, especially in relation to specific co-morbidities

Figure 2. age-specific analysis, in relation to specific Elixhauser co-morbidities

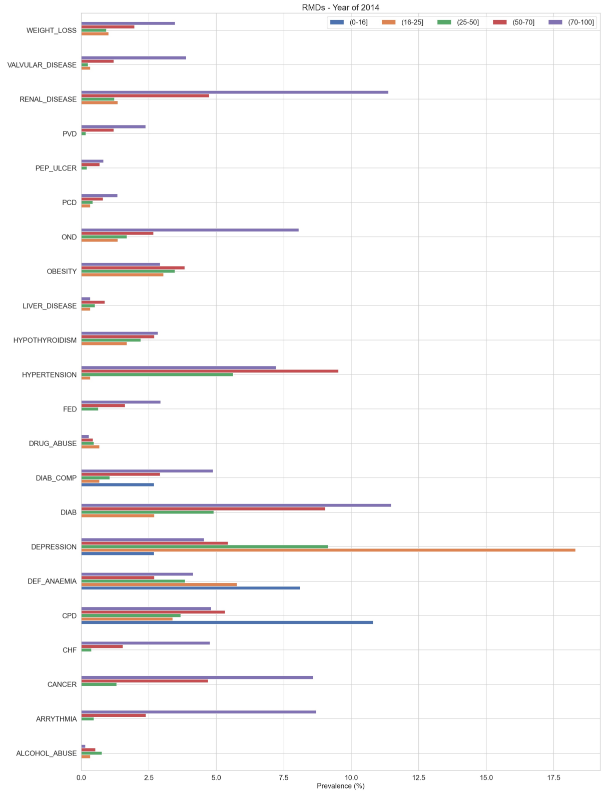

(i) age-specific analysis, especially in relation to specific co-morbidities,

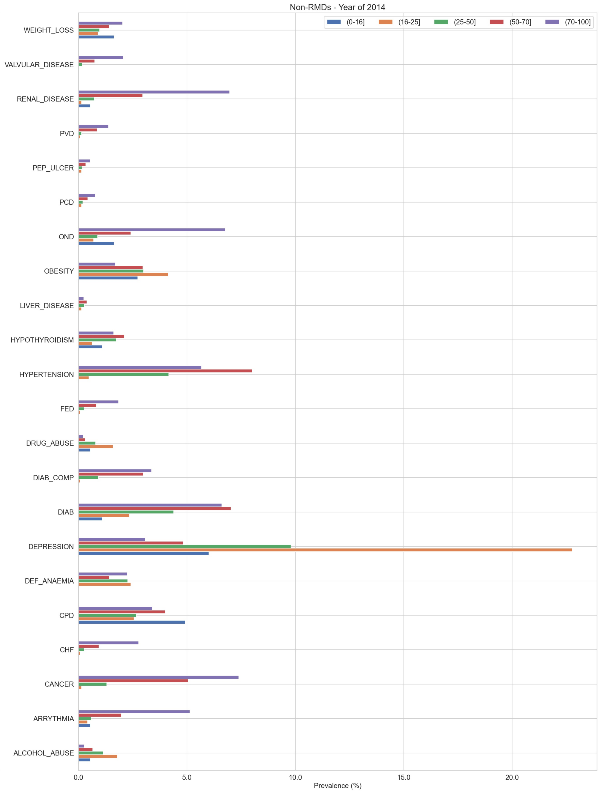

(j) age-specific analysis, especially in relation to specific co-morbidities,

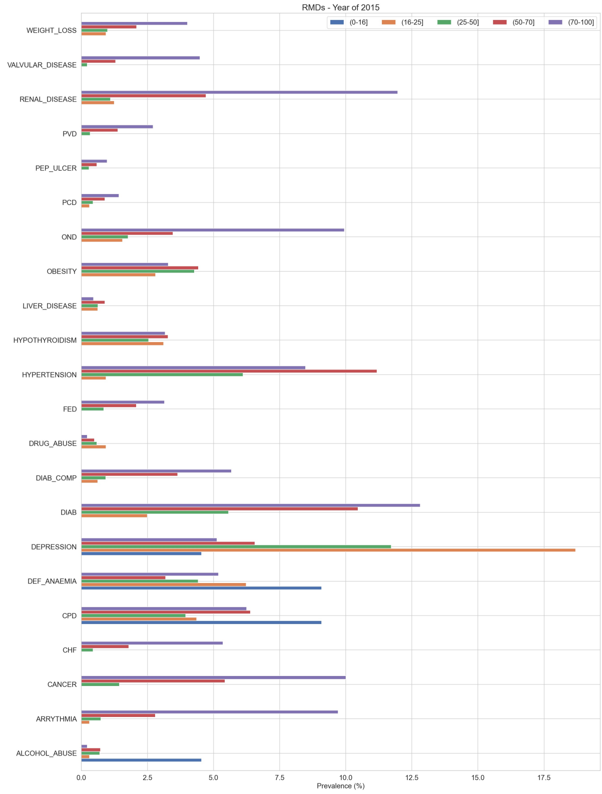

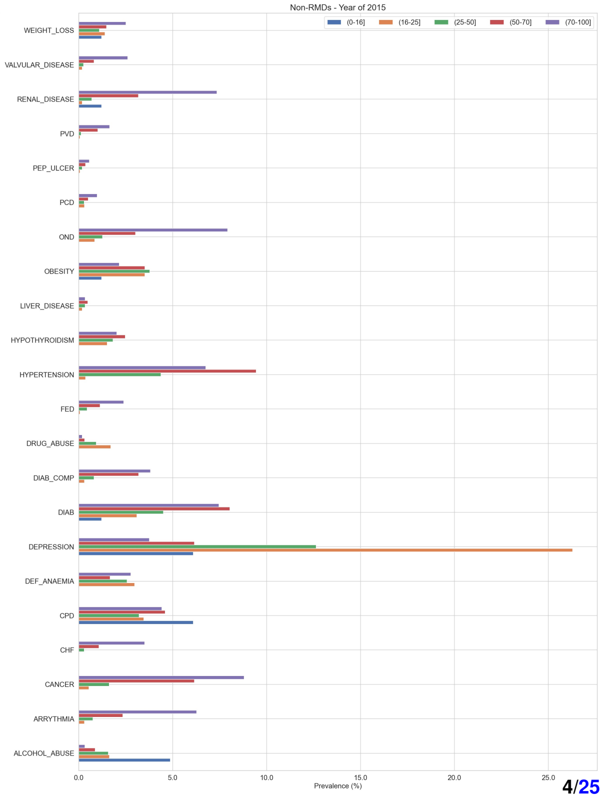

(k) age-specific analysis, especially in relation to specific co-morbidities

(l) age-specific analysis, especially in relation to specific co-morbidities

Figure 2. age-specific analysis, in relation to specific Elixhauser co-morbidities

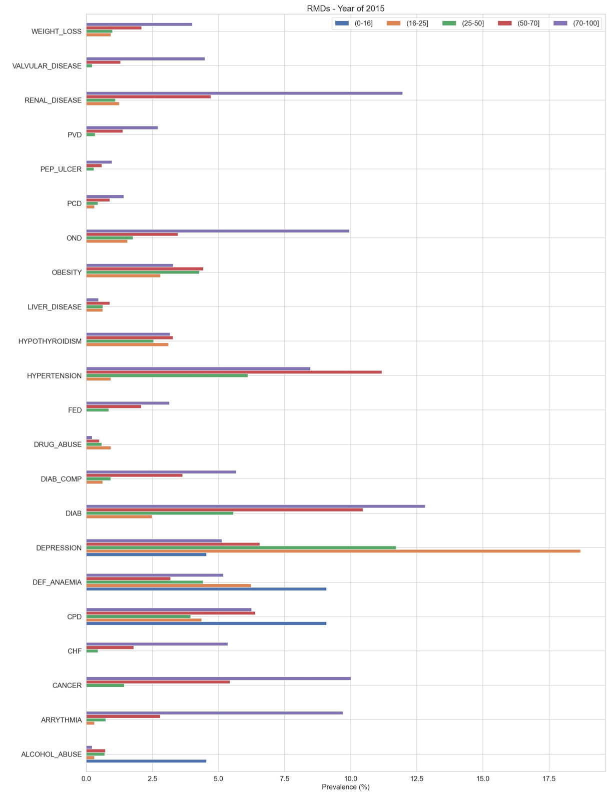

(m) age-specific analysis, especially in relation to specific co-morbidities,

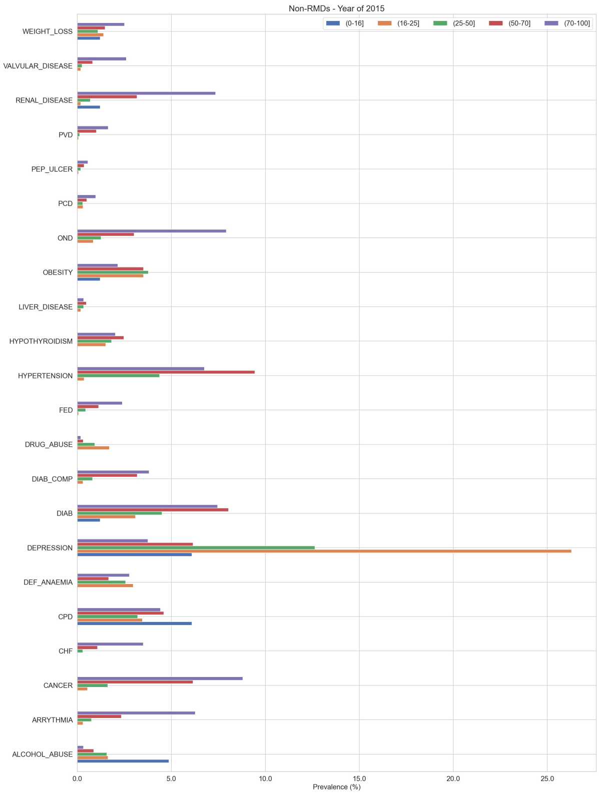

(n) age-specific analysis, especially in relation to specific co-morbidities,

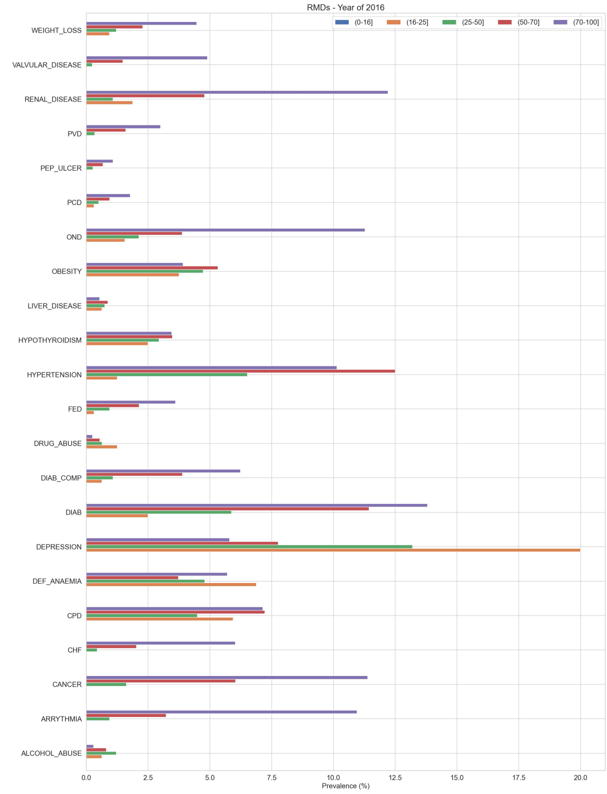

(o) age-specific analysis, especially in relation to specific co-morbidities,

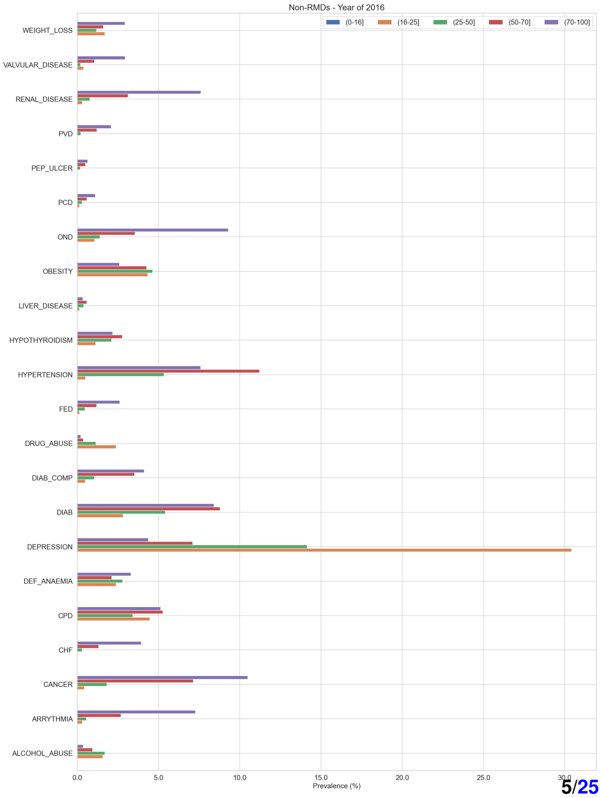

(p) age-specific analysis, especially in relation to specific co-morbidities,

Figure 2. age-specific analysis, in relation to specific Elixhauser co-morbidities

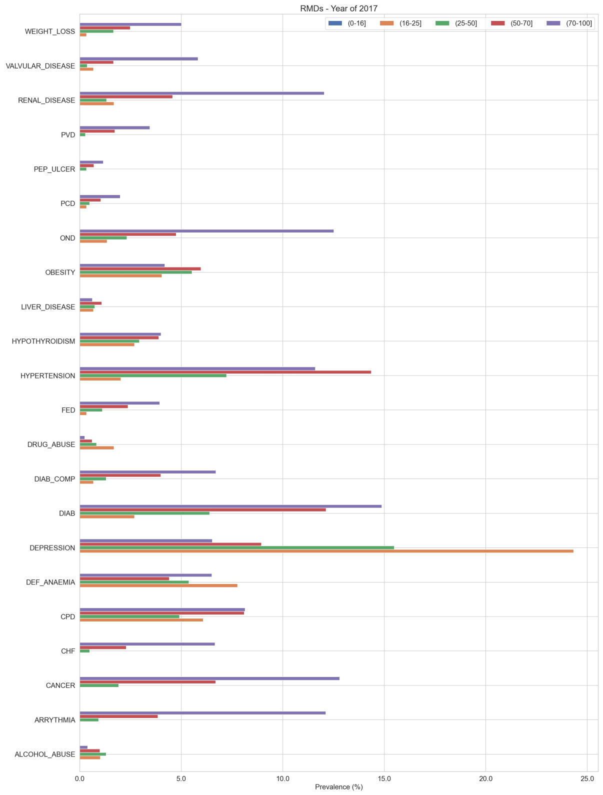

(q) age-specific analysis, especially in relation to specific co-morbidities,

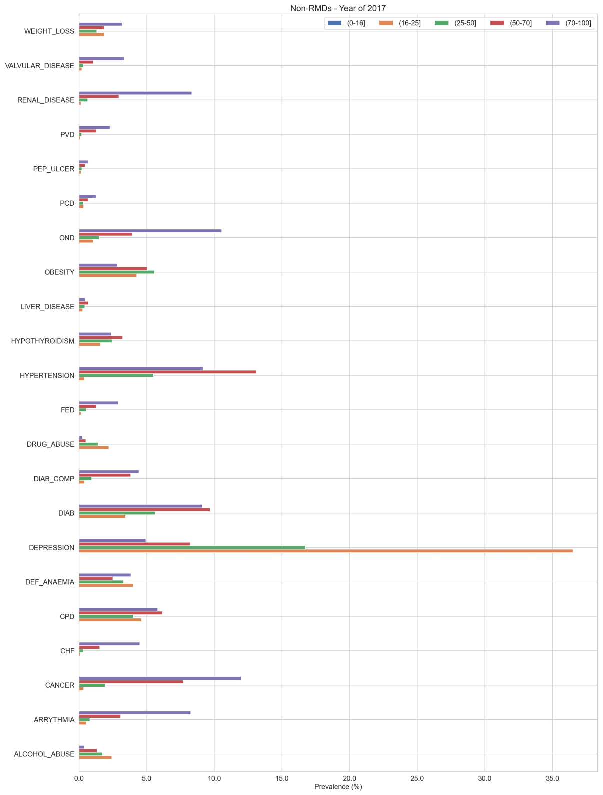

(r) age-specific analysis, especially in relation to specific co-morbidities

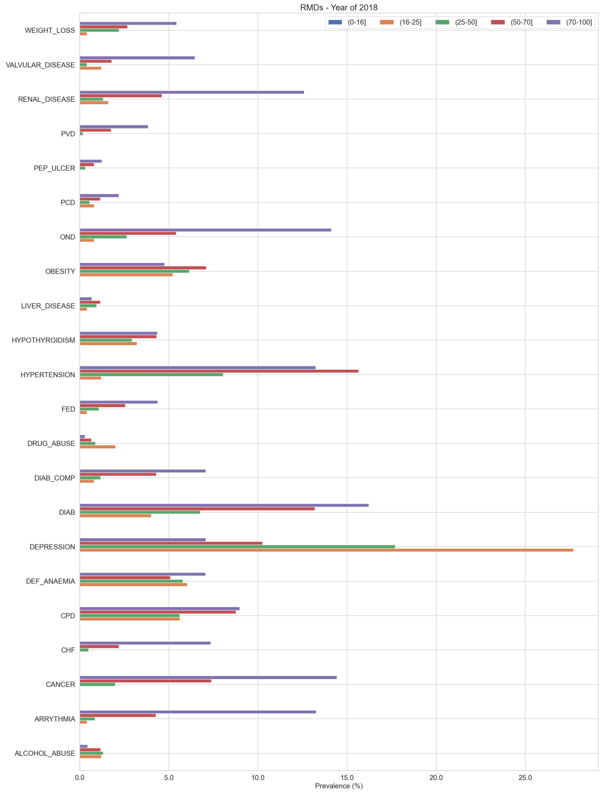

(s) age-specific analysis, especially in relation to specific co-morbidities,

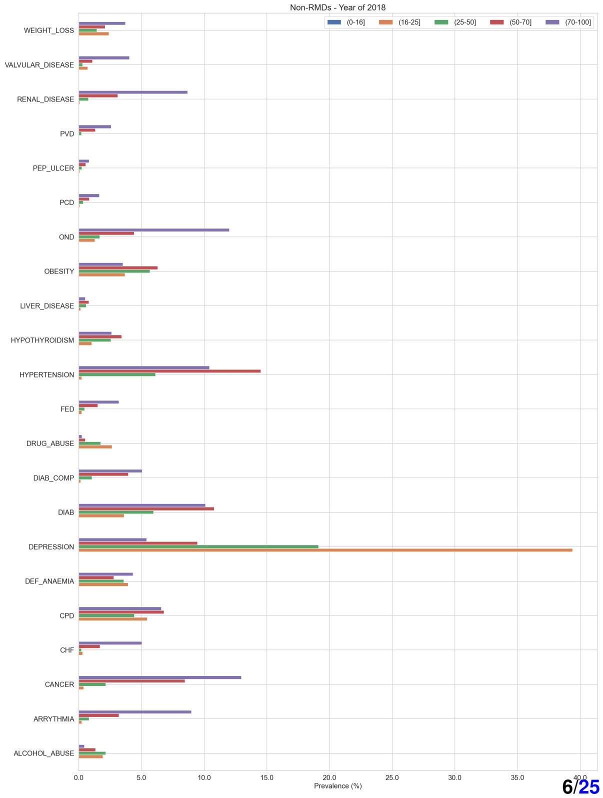

(t) age-specific analysis, especially in relation to specific co-morbidities,

Figure 2. age-specific analysis, in relation to specific Elixhauser co-morbidities

Jalali-najafabadi F, *et al. BMJ Open* 2024; 14:e079169. doi: 10.1136/bmjopen-2023-079169

	2010		2011)		(2012)		2013		2014		2015		2016		2017		2018		2019	
Conditions	RMD	Comp	RMD	Comp	RMD	Comp	RMD	Comp	RMD	Comp	RMD	Comp	RMD	Comp	RMD	Comp	RMD	Comp	RMD	Comp
ALCOHOL_ABUSE	0.2 (0.02 0.32)	0.1 (0.05-0.16)	0.3 (0.17 0.45)	0.2 (0.19-0.31)	0.4 (0.23 0.49)	0.4 (0.30-0.41)	0.4 (0.27 0.49)	0.4 (0.38-0.49)	0.4 (0.30 0.51)	0.6 (0.54-0.65)	0.5 (0.40 0.61)	0.8 (0.72-0.84)	0.6 (0.53 0.76)	0.8 (0.76-0.88)	0.8 (0.66 0.89)	1.0 (0.94-1.06)	0.9 (0.74 0.99)	1.1 (1.02-1.14)	1.0 (0.86 1.11)	1.3 (1.21-1.33)
ARRYTHMIA	1.0 (0.64 1.37)	0.6 (0.51-0.77)	2.1 (1.78 2.52)	1.4 (1.23-1.50)	3.0 (2.64 3.36)	1.9 (1.77-2.03)	4.0 (3.64 4.35)	2.5 (2.33-2.59)	4.6 (4.30 4.98)	3.0 (2.90-3.15)	5.4 (5.01 5.69)	3.7 (3.58-3.84)	6.1 (5.80 6.48)	4.3 (4.16-4.42)	7.0 (6.65 7.34)	5.0 (4.84-5.11)	7.8 (7.45 8.15)	5.4 (5.30-5.57)	8.4 (8.02 8.71)	6.2 (6.11-6.38)
CPD	1.0 (0.67 1.41)	0.6 (0.49-0.75)	2.2 (1.79 2.54)	1.4 (1.25-1.52)	3.3 (2.89 3.64)	2.0 (1.88-2.14)	4.1 (3.78 4.51)	2.7 (2.61-2.88)	4.8 (4.47 5.17)	3.5 (3.38-3.65)	5.9 (5.55 6.26)	4.3 (4.15-4.42)	6.7 (6.38 7.10)	4.9 (4.77-5.04)	7.6 (7.24 7.96)	5.6 (5.51-5.79)	8.4 (7.99 8.72)	6.3 (6.19-6.48)	9.1 (8.74 9.46)	6.9 (6.80-7.09)
CHF	0.5 (0.23 0.74)	0.3 (0.25-0.44)	1.2 (0.89 1.43)	0.7 (0.60-0.79)	1.9 (1.57 2.15)	1.0 (0.87-1.06)	2.2 (1.95 2.48)	1.3 (1.26-1.44)	2.7 (2.39 2.91)	1.6 (1.48-1.66)	3.0 (2.79 3.31)	2.0 (1.86-2.05)	3.5 (3.21 3.73)	2.3 (2.16-2.35)	3.9 (3.65 4.17)	2.6 (2.53-2.72)	4.3 (3.99 4.52)	3.0 (2.87-3.07)	4.5 (4.28 4.81)	3.2 (3.15-3.35)
DEF_ANAEMIA	1.1 (0.75 1.53)	0.5 (0.37-0.60)	2.1 (1.71 2.44)	0.8 (0.71-0.91)	2.4 (2.09 2.74)	1.2 (1.08-1.29)	2.9 (2.61 3.22)	1.6 (1.54-1.75)	3.6 (3.27 3.87)	1.9 (1.83-2.03)	4.3 (4.00 4.61)	2.3 (2.20-2.41)	4.8 (4.50 5.10)	2.7 (2.64-2.84)	5.5 (5.23 5.85)	3.2 (3.12-3.34)	6.1 (5.79 6.41)	3.6 (3.52-3.74)	6.8 (6.44 7.07)	4.0 (3.86-4.08)
DEPRESSION	1.6 (1.16 2.09)	1.2 (1.00-1.35)	2.9 (2.52 3.38)	2.1 (1.91-2.24)	3.9 (3.47 4.29)	3.3 (3.11-3.45)	5.2 (4.79 5.60)	4.3 (4.17-4.50)	5.9 (5.55 6.32)	5.3 (5.12-5.45)	7.0 (6.63 7.40)	6.6 (6.41-6.75)	8.0 (7.59 8.36)	7.4 (7.26-7.59)	9.1 (8.72 9.50)	8.5 (8.31-8.65)	10.1 (9.74 10.53	9.4 (9.25-9.59)	11.2 (10.7 11.58	10.5 (10.31-10.65)
DIAB	2.2 (1.68 2.75)	1.2 (1.05-1.40)	4.3 (3.74 4.78)	2.6 (2.39-2.76)	5.8 (5.35 6.34)	3.6 (3.43-3.78)	7.9 (7.42 8.41)	5.0 (4.79-5.14)	9.2 (8.75 9.69)	6.3 (6.13-6.49)	10.5 (10.0 10.97	7.1 (6.95-7.30)	11.4 (10.9 11.89	8.0 (7.83-8.17)	12.3 (11.8 12.77	8.7 (8.54-8.89)	13.5 (13.0 13.94	9.7 (9.51-9.85)	14.4 (13.9 14.80	10.4 (10.21-10.56)
DIAB_COMP	0.6 (0.31 0.87)	0.4 (0.29-0.50)	1.0 (0.77 1.28)	0.8 (0.68-0.88)	1.5 (1.27 1.79)	1.2 (1.13-1.34)	2.6 (2.35 2.94)	2.0 (1.93-2.16)	3.4 (3.09 3.68)	2.7 (2.62-2.86)	4.0 (3.71 4.30)	3.0 (2.90-3.13)	4.4 (4.11 4.70)	3.3 (3.22-3.45)	4.7 (4.44 5.02)	3.6 (3.48-3.71)	5.1 (4.76 5.34)	4.0 (3.86-4.09)	5.5 (5.18 5.76)	4.3 (4.22-4.45)
DRUG_ABUSE	0.2 (0.02 0.32)	0.1 (0.02-0.09)	0.2 (0.11 0.36)	0.1 (0.10-0.19)	0.3 (0.21 0.45)	0.2 (0.16-0.24)	0.4 (0.25 0.46)	0.3 (0.23-0.31)	0.4 (0.28 0.48)	0.4 (0.33-0.42)	0.4 (0.31 0.50)	0.4 (0.34-0.43)	0.4 (0.35 0.54)	0.5 (0.41-0.50)	0.5 (0.41 0.60)	0.6 (0.51-0.60)	0.5 (0.45 0.64)	0.6 (0.57-0.66)	0.6 (0.53 0.73)	0.7 (0.63-0.72)

Table 1 – continued from previous page

FED	0.7 (0.39 0.99)	0.3 (0.21-0.39)	1.2 (0.89 1.43)	0.6 (0.52-0.70)	1.5 (1.23 1.74)	0.6 (0.57-0.72)	1.7 (1.45 1.92)	1.0 (0.90-1.06)	2.0 (1.74 2.20)	1.1 (1.06-1.21)	2.3 (2.06 2.52)	1.5 (1.45-1.62)	2.6 (2.33 2.78)	1.7 (1.59-1.75)	2.8 (2.62 3.07)	1.9 (1.78-1.94)	3.1 (2.91 3.37)	2.1 (2.03-2.20)	3.4 (3.19 3.65)	2.4 (2.29-2.46)
HYPERTENSION	2.4 (1.80 2.90)	1.2 (1.03-1.38)	3.3 (2.80 3.71)	2.6 (2.45-2.81)	4.7 (4.29 5.19)	3.6 (3.44-3.79)	6.1 (5.66 6.53)	5.1 (4.89-5.25)	7.7 (7.28 8.15)	6.2 (6.06-6.41)	9.0 (8.58 9.45)	7.3 (7.13-7.48)	10.4 (9.93 10.80	8.6 (8.38-8.73)	11.9 (11.4 12.31	10.0 (9.85-10.21)	13.3 (12.8 13.70	11.2 (11.06-11.43)	15.0 (14.5 15.40	12.6 (12.39-12.77)
HYPOTHYROIDISM	1.0 (0.64 1.37)	0.3 (0.24-0.43)	1.4 (1.11 1.72)	0.8 (0.70-0.90)	1.9 (1.57 2.15)	1.2 (1.07-1.27)	2.2 (1.98 2.52)	1.4 (1.32-1.51)	2.7 (2.39 2.91)	1.8 (1.72-1.91)	3.1 (2.85 3.37)	2.2 (2.06-2.26)	3.4 (3.11 3.62)	2.4 (2.29-2.48)	3.8 (3.52 4.04)	2.7 (2.62-2.81)	4.1 (3.86 4.38)	2.9 (2.81-3.01)	4.3 (4.06 4.58)	3.1 (3.02-3.22)
LIVER_DISEASE	0.2 (0.04 0.37)	0.1 (0.02-0.09)	0.3 (0.15 0.43)	0.1 (0.10-0.18)	0.4 (0.25 0.51)	0.2 (0.16-0.24)	0.4 (0.32 0.56)	0.3 (0.26-0.35)	0.6 (0.45 0.70)	0.3 (0.26-0.34)	0.7 (0.54 0.78)	0.4 (0.35-0.43)	0.7 (0.59 0.83)	0.4 (0.39-0.48)	0.8 (0.70 0.94)	0.5 (0.49-0.57)	0.9 (0.78 1.02)	0.6 (0.59-0.69)	1.0 (0.91 1.17)	0.7 (0.68-0.78)
OBESITY	0.9 (0.53 1.20)	0.6 (0.46-0.71)	1.4 (1.08 1.68)	1.0 (0.89-1.12)	2.0 (1.74 2.33)	1.7 (1.54-1.79)	2.6 (2.32 2.90)	2.0 (1.91-2.14)	3.4 (3.07 3.66)	2.5 (2.36-2.59)	3.9 (3.59 4.17)	3.0 (2.87-3.10)	4.6 (4.30 4.90)	3.6 (3.49-3.72)	5.1 (4.79 5.39)	4.1 (4.00-4.25)	5.9 (5.57 6.18)	4.9 (4.80-5.05)	7.0 (6.69 7.33)	5.9 (5.74-6.00)
OND	0.9 (0.56 1.24)	0.7 (0.56-0.83)	2.2 (1.79 2.54)	1.6 (1.42-1.70)	2.8 (2.49 3.19)	2.3 (2.12-2.40)	3.8 (3.44 4.14)	3.0 (2.84-3.11)	4.7 (4.36 5.06)	3.9 (3.79-4.07)	5.9 (5.55 6.26)	4.8 (4.63-4.91)	6.7 (6.39 7.11)	5.6 (5.50-5.79)	7.8 (7.40 8.12)	6.4 (6.29-6.58)	8.9 (8.53 9.27)	7.4 (7.26-7.56)	10.2 (9.86 10.62	8.6 (8.41-8.73)
PEP_ULCER	0.2 (0.04 0.37)	0.1 (0.06-0.16)	0.3 (0.15 0.43)	0.2 (0.11-0.21)	0.4 (0.24 0.50)	0.2 (0.17-0.26)	0.5 (0.40 0.66)	0.3 (0.23-0.32)	0.6 (0.52 0.78)	0.4 (0.34-0.43)	0.7 (0.57 0.82)	0.4 (0.37-0.45)	0.8 (0.65 0.90)	0.5 (0.46-0.55)	0.8 (0.71 0.96)	0.5 (0.47-0.55)	0.9 (0.80 1.05)	0.6 (0.58-0.67)	1.0 (0.85 1.09)	0.7 (0.63-0.72)
PVD	0.2 (0.06 0.42)	0.2 (0.11-0.25)	0.4 (0.25 0.57)	0.4 (0.28-0.42)	0.8 (0.63 1.02)	0.6 (0.50-0.65)	1.3 (1.07 1.48)	0.7 (0.67-0.81)	1.5 (1.30 1.69)	0.9 (0.87-1.01)	1.8 (1.55 1.95)	1.1 (1.05-1.19)	2.0 (1.78 2.17)	1.4 (1.33-1.48)	2.2 (2.04 2.44)	1.5 (1.46-1.61)	2.4 (2.24 2.64)	1.7 (1.64-1.79)	2.5 (2.31 2.71)	1.9 (1.79-1.94)
PCD	0.2 (0.06 0.42)	0.1 (0.04-0.14)	0.4 (0.26 0.59)	0.2 (0.17-0.28)	0.7 (0.48 0.82)	0.3 (0.28-0.39)	0.7 (0.56 0.87)	0.4 (0.35-0.46)	1.0 (0.79 1.11)	0.5 (0.47-0.58)	1.0 (0.88 1.19)	0.7 (0.62-0.73)	1.2 (1.07 1.38)	0.8 (0.70-0.81)	1.4 (1.21 1.52)	0.9 (0.82-0.94)	1.5 (1.37 1.69)	1.1 (1.07-1.19)	1.7 (1.58 1.91)	1.2 (1.10-1.22)
RENAL_DISEASE	1.9 (1.40 2.40)	1.0 (0.84-1.17)	3.3 (2.87 3.78)	1.9 (1.75-2.06)	4.7 (4.29 5.19)	2.8 (2.65-2.97)	6.2 (5.72 6.60)	3.7 (3.53-3.84)	6.8 (6.42 7.24)	4.2 (4.05-4.34)	7.1 (6.75 7.53)	4.5 (4.35-4.63)	7.3 (6.98 7.72)	4.6 (4.51-4.77)	7.3 (6.98 7.68)	4.9 (4.79-5.05)	7.7 (7.36 8.06)	5.3 (5.13-5.39)	7.8 (7.44 8.12)	5.5 (5.39-5.64)

Table 1 – continued from previous page

CANCER	1.3 (0.87 1.69)	1.1 (0.94- 1.28)	2.4 (2.00- 2.78)	2.2 (2.00- 2.33)	3.4 (3.04- 3.81)	3.2 (3.00- 3.33)	4.4 (4.05- 4.80)	4.2 (4.02- 4.35)	5.6 (5.27- 6.03)	5.3 (5.13- 5.46)	6.6 (6.25- 7.00)	6.4 (6.27- 6.60)	7.6 (7.17- 7.93)	7.6 (7.46- 7.80)	8.6 (8.19 8.95)	8.6 (8.43- 8.77)	9.7 (9.31- 10.08	9.5 (9.30- 9.64)	10.5 (10.1- 10.90	10.6 (10.43- 10.78)
VALVULAR_DISEASE	0.4 (0.21 0.69)	0.2 (0.14- 0.30)	0.8 (0.59- 1.05)	0.5 (0.42- 0.58)	1.3 (1.07- 1.55)	0.7 (0.64- 0.80)	1.7 (1.44- 1.91)	1.0 (0.89- 1.05)	2.1 (1.91- 2.38)	1.2 (1.10- 1.26)	2.5 (2.22- 2.68)	1.5 (1.39- 1.55)	2.7 (2.50- 2.96)	1.5 (1.39- 1.55)	3.3 (3.05 3.53)	1.9 (1.85- 2.02)	3.7 (3.46- 3.95)	2.3 (2.22- 2.40)	4.2 (3.92- 4.42)	2.5 (2.46- 2.64)
WEIGHT_LOSS	0.7(0	0.3 (0.22- 0.40)	4)1.1(0	0.5 (0.46- 0.63)	1.8 6)(1.49- 2.05)	0.9 (0.79- 0.96)	2.1 (1.88- 2.41)	1.1 (1.01- 1.18)	2.4 (2.15- 2.65)	1.6 (1.50- 1.68)	2.7 (2.46- 2.95)	1.9 (1.76- 1.94)	3.0 (2.79- 3.28)	2.1 (2.02- 2.20)	3.4 (3.20 3.69)	2.4 (2.26- 2.45)	3.8 (3.59- 4.09)	2.7 (2.65- 2.84)	4.2 (3.92- 4.42)	3.1 (2.98- 3.17)

Table 1. Annual prevalence rates for comorbidities in RMDs cases and matched comparators 2010-2019

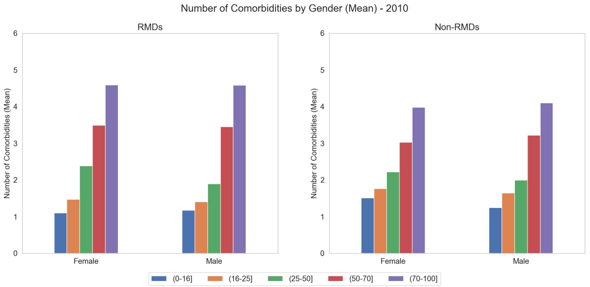

(a) Mean number of comorbidities in patients with RMDs and comparators by age categories and sex 2010

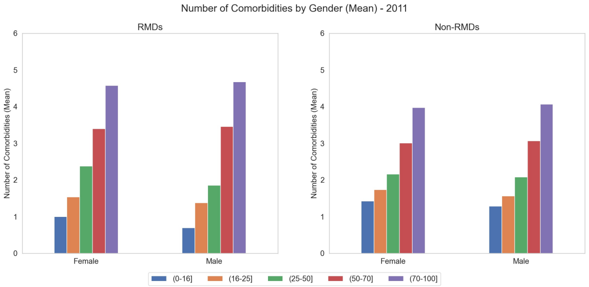

(b) Mean number of comorbidities in patients with RMDs and comparators by age categories and sex 2011

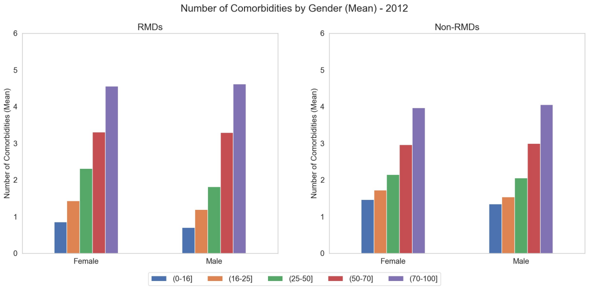

(c) Mean number of comorbidities in patients with RMDs and comparators by age categories and sex 2012

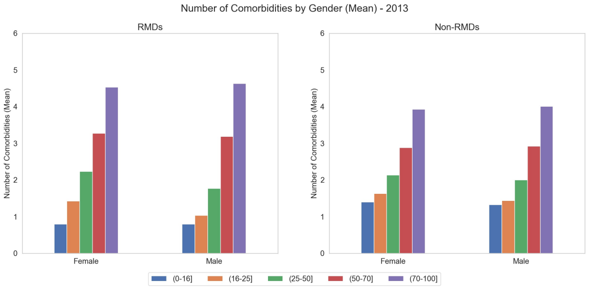

(d) Mean number of comorbidities in patients with RMDs and comparators by age categories and sex 2013

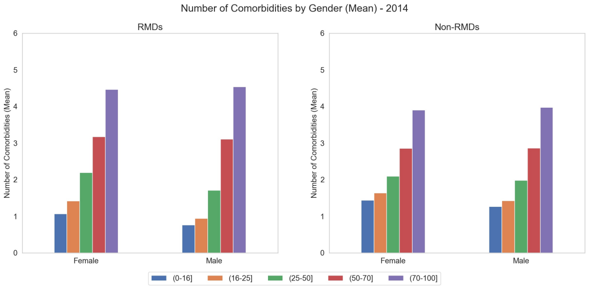

(e) Mean number of comorbidities in patients with RMDs and comparators by age categories and sex 2014

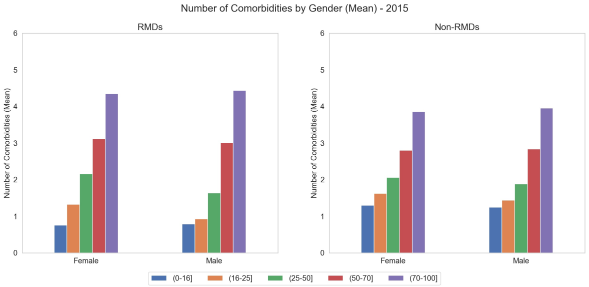

(f) Mean number of comorbidities in patients with RMDs and comparators by age categories and sex 2015

Figure 3. Mean number of comorbidities in patients with RMDs and comparators by age categories and sex 2010 and 2015

1 abbreviations

The Table 2 describes the significance of various abbreviations and acronyms used throughout of this paper.

Jalali-najafabadi F, *et al. BMJ Open* 2024; 14:e079169. doi: 10.1136/bmjopen-2023-079169

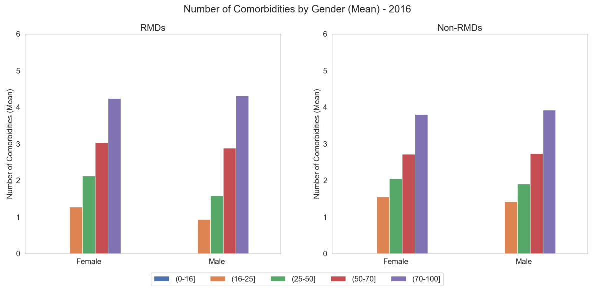

(g) Mean number of comorbidities in patients with RMDs and comparators by age categories and sex 2016

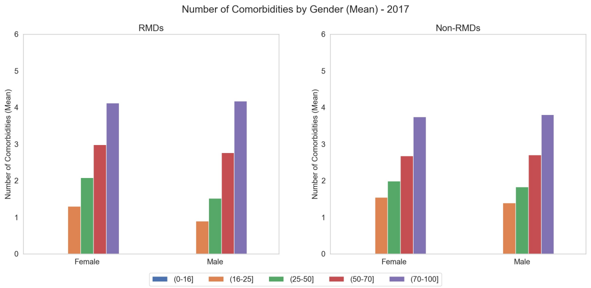

(h) Mean number of comorbidities in patients with RMDs and comparators by age categories and sex 2017

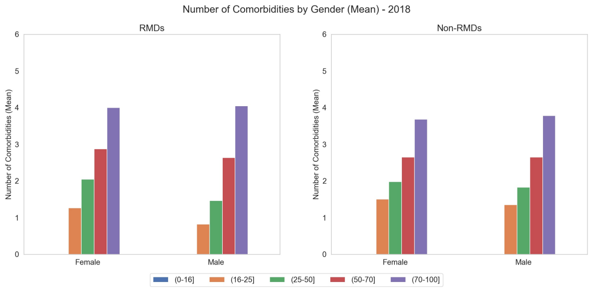

(i) Mean number of comorbidities in patients with RMDs and comparators by age categories and sex 2018

Figure 3. Mean number of comorbidities in patients with RMDs and comparators by age categories and sex 2016 and 2018

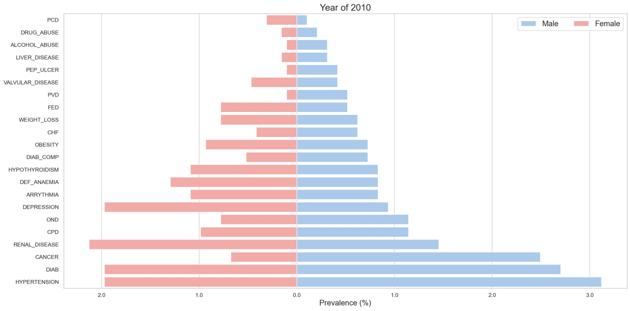

(a) Prevalence of Elixhuaser comorbidities patients with RMDs 2010

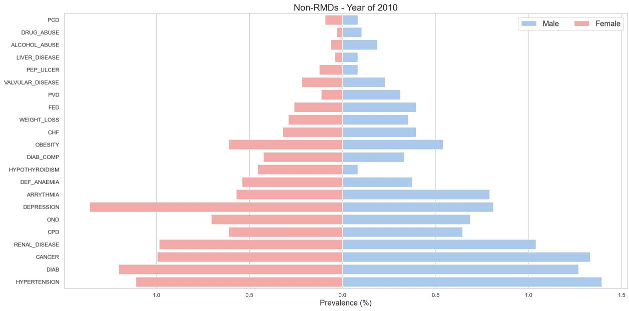

(b) Prevalence of Elixhuaser comorbidities patients with RMDs 2010

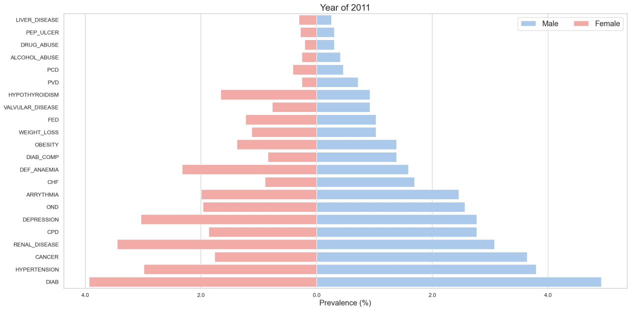

(c) Prevalence of Elixhauser comorbidities in comparators 2011

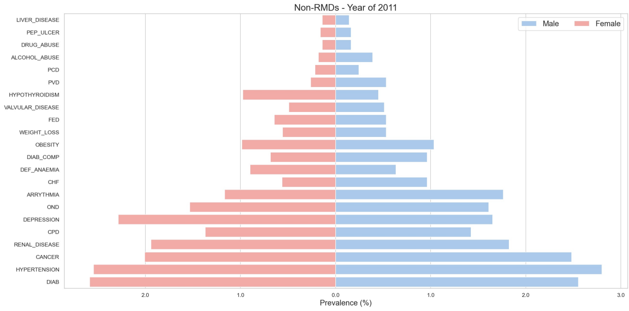

(d) Prevalence of Elixhauser comorbidities in comparators 2011

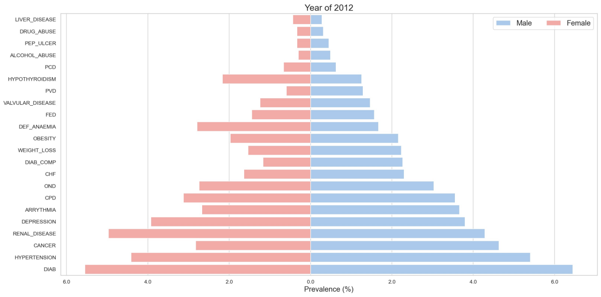

(e) Prevalence of Elixhuaser comorbidities patients with RMDs 2012

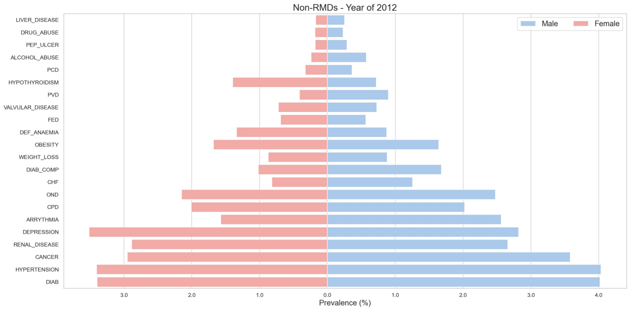

(f) Prevalence of Elixhauser comorbidities in comparators 2012

Figure 4. Prevalence of Elixhauser comorbidities in patients with RMDs and patients without RMDS in 2010 and 2012

Jalali-najafabadi F, *et al. BMJ Open* 2024; 14:e079169. doi: 10.1136/bmjopen-2023-079169

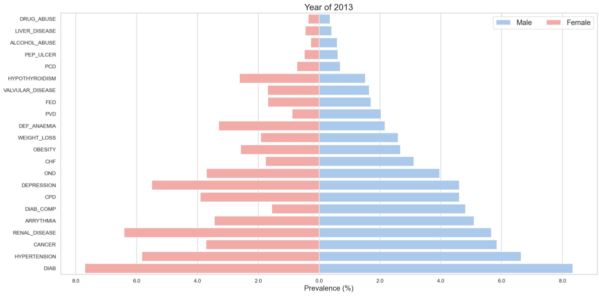

(g) Prevalence of Elixhuaser comorbidities patients with RMDs 2013

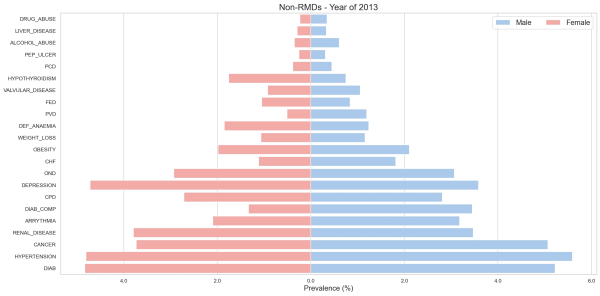

(h) Prevalence of Elixhuaser comorbidities patients without RMDs 2013

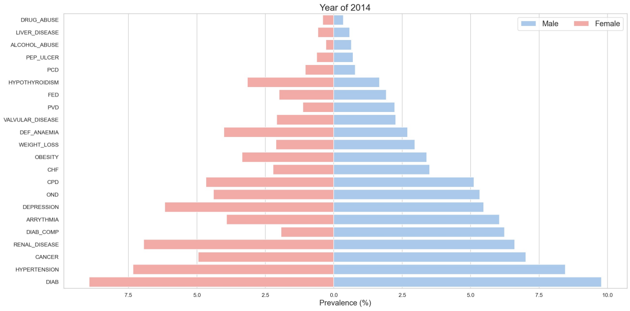

(i) Prevalence of Eixhuaser comorbidities patients with RMDs 2014

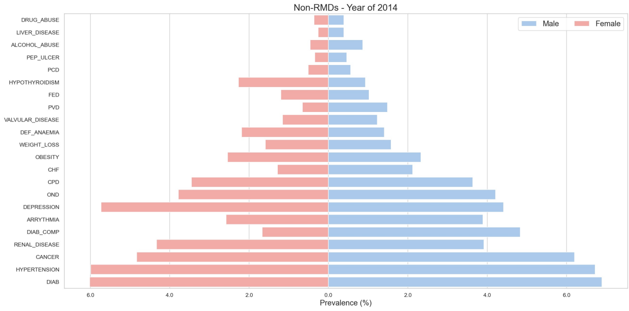

(j) Prevalence of Elixhuaser comorbidities patients with RMDs 2014

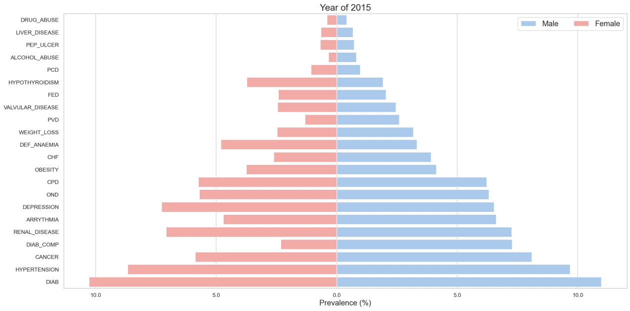

(k) Prevalence of Elixhuaser comorbidities patients without RMDs 2015

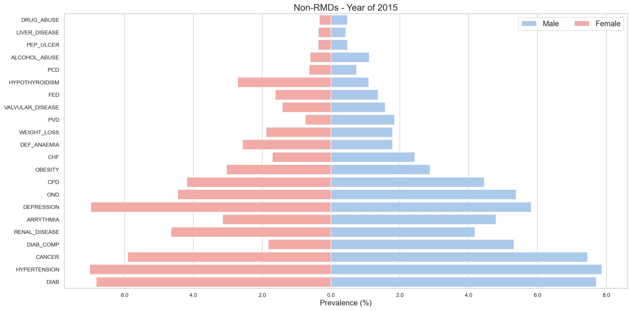

(l) Prevalence of Elixhuaser comorbidities patients with RMDs 2015

Figure 4. Prevalence of Elixhauser comorbidities in patients with RMDs and patients without RMDS in 2013 and 2015

Jalali-najafabadi F, *et al. BMJ Open* 2024; 14:e079169. doi: 10.1136/bmjopen-2023-079169

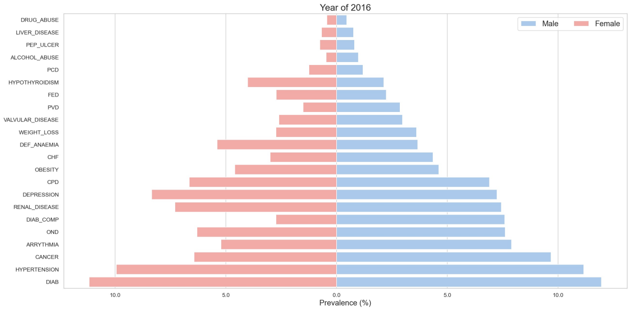

(m) Prevalence of Elixhuaser comorbidities patients with RMDs 2016

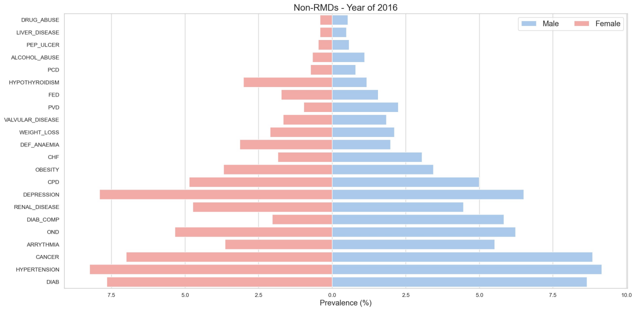

(n) Prevalence of Elixhuaser comorbidities patients with RMDs 2016

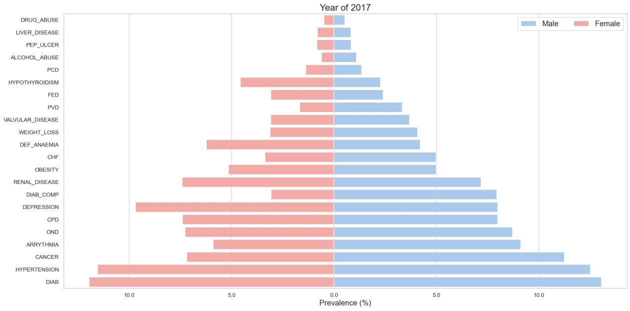

(o) Prevalence of Elixhuaser comorbidities patients without RMDs 2017

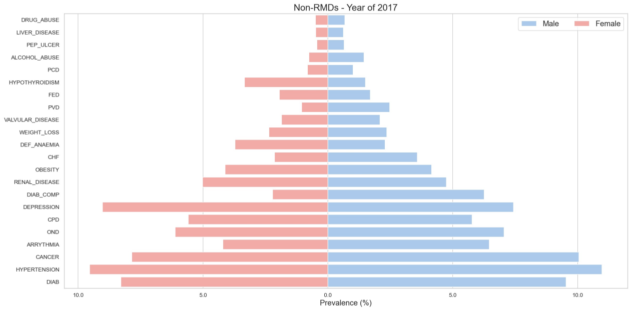

(p) Prevalence of Elixhuaser comorbidities patients with RMDs 2017

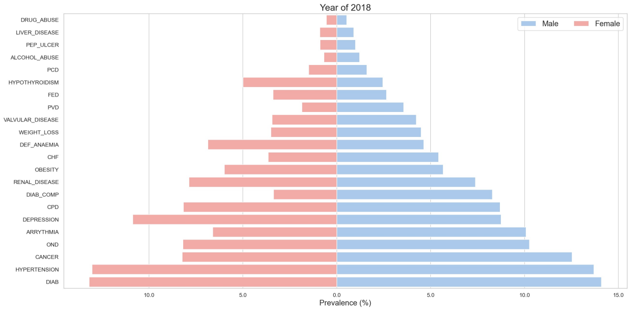

(q) Prevalence of Elixhuaser comorbidities patients without RMDs 2018

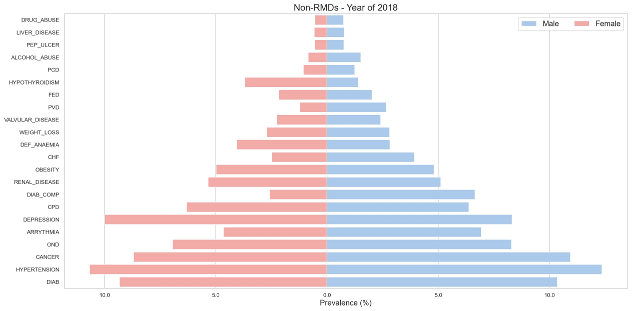

(r) Prevalence of Elixhuaser comorbidities patients with RMDs 2018

Figure 4. Prevalence of Elixhauser comorbidities in patients with RMDs and patients without RMDS in 2016 and 2018

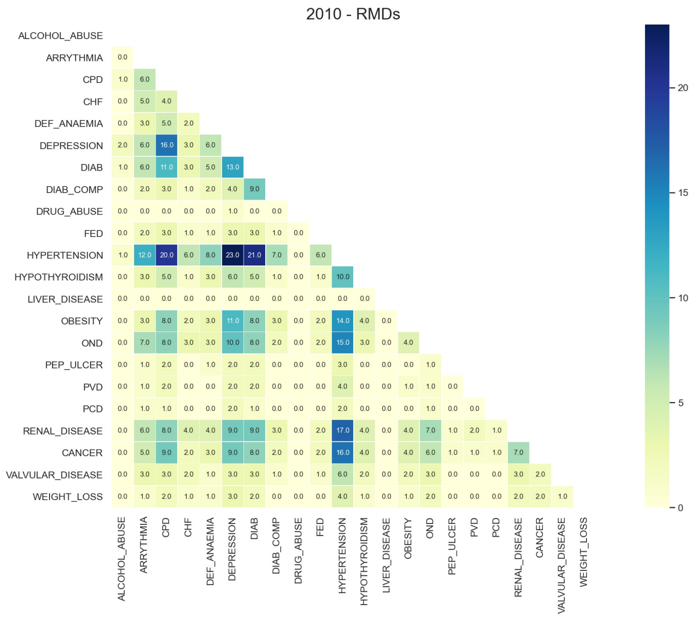

(a) Percentage of patients with RMDs and Comparators

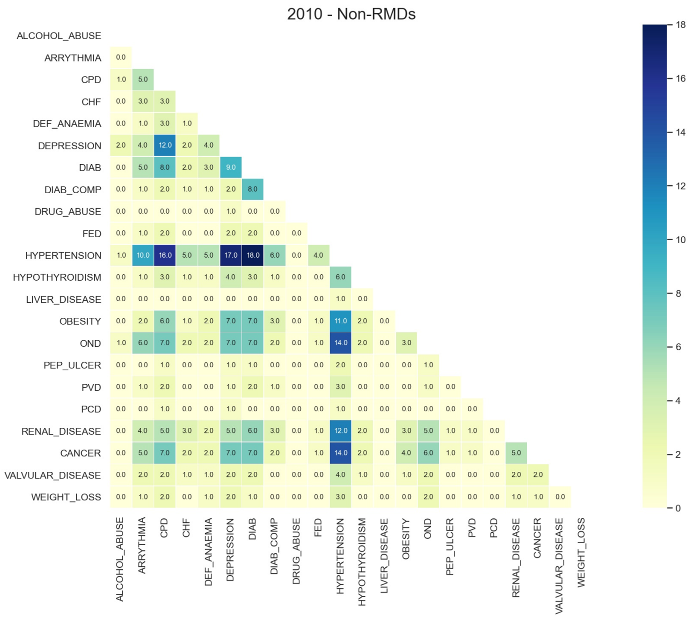

(b) Percentage of patients with RMDs and Comparators

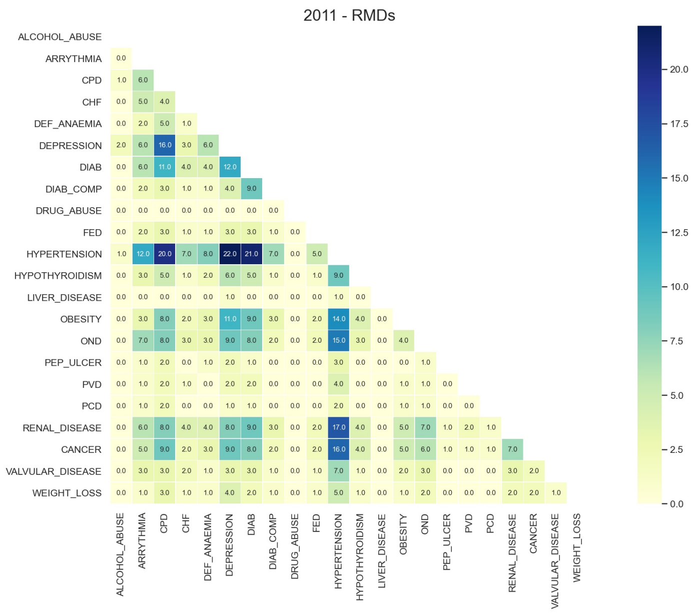

(c) Percentage of patients with RMDs and Comparators

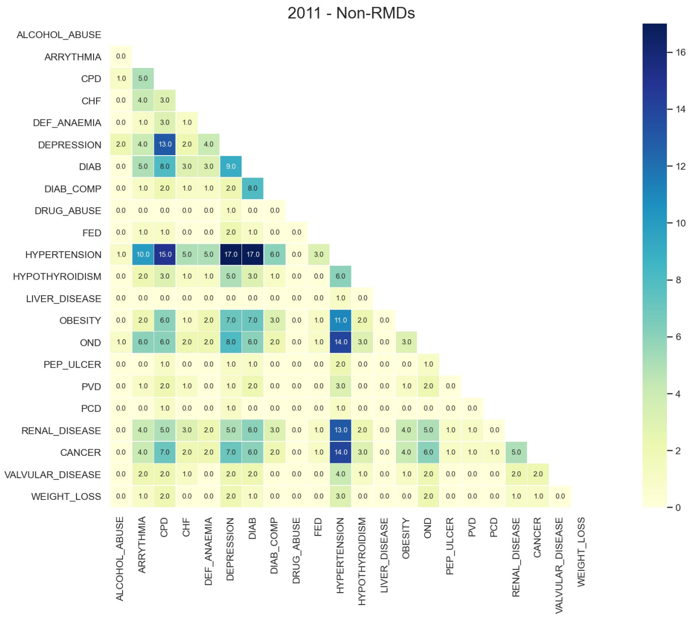

(d) Percentage of patients with RMDs and Comparators

Figure 5. Proportion of patients with RMDs and Comparators

Jalali-najafabadi F, *et al. BMJ Open* 2024; 14:e079169. doi: 10.1136/bmjopen-2023-079169

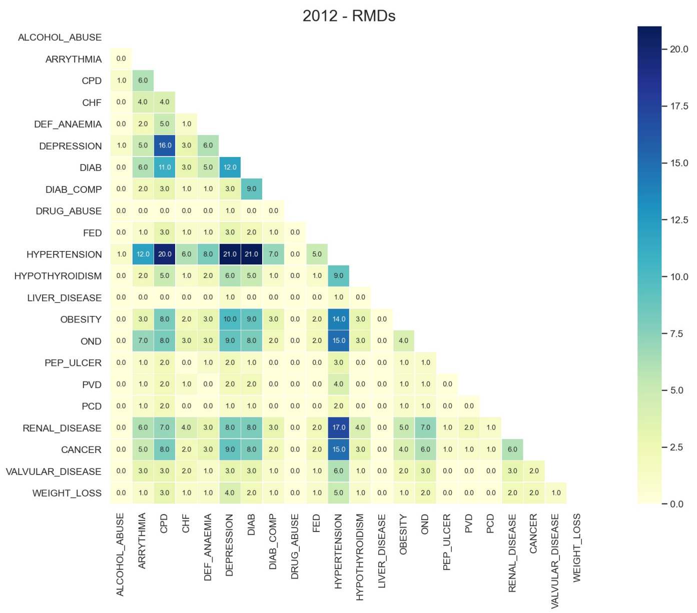

(e) Percentage of patients with RMDs and Comparators

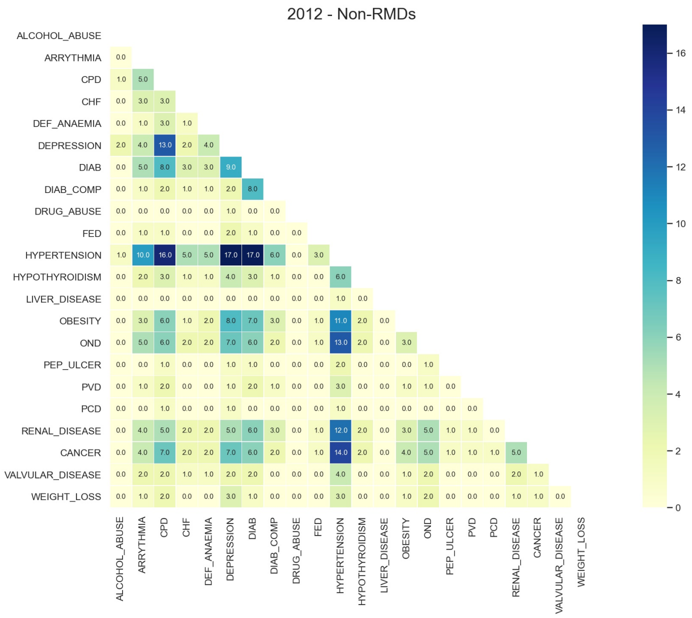

(f) Percentage of patients with RMDs and Comparators

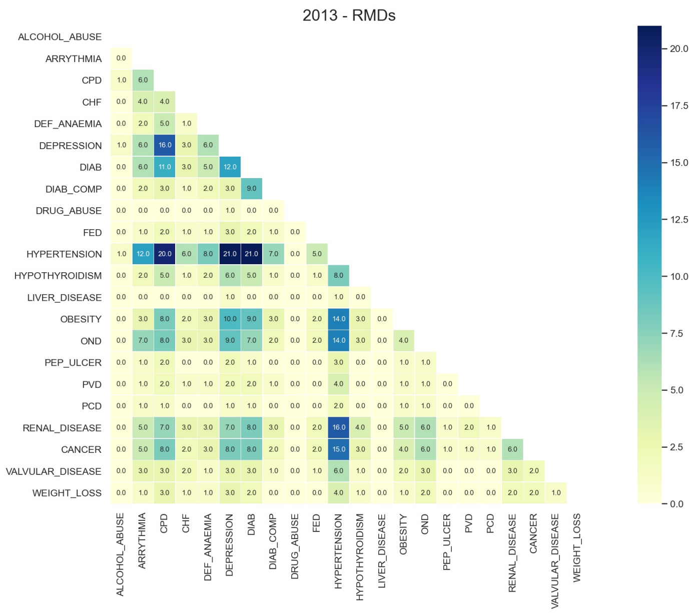

(g) Percentage of patients with RMDs and Comparators

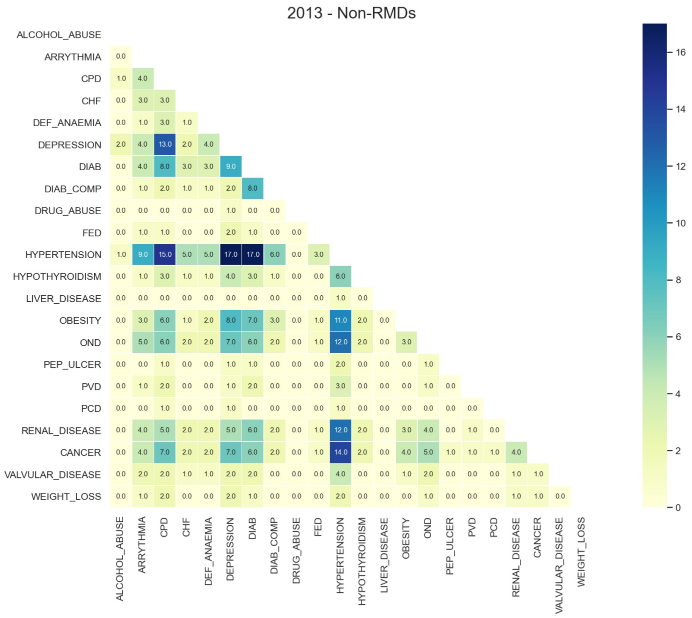

(h) Percentage of patients with RMDs and Comparators

Figure 5. Proportion of patients with RMDs an Comparators

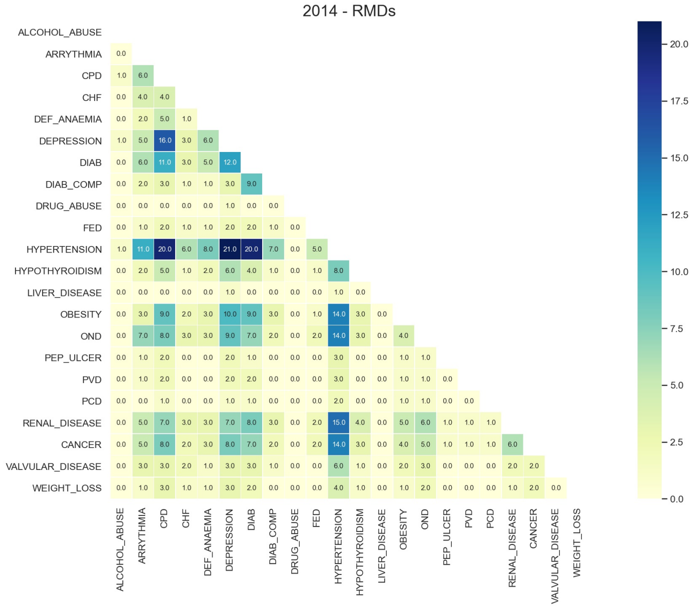

(i) Percentage of patients with RMDs and Comparators

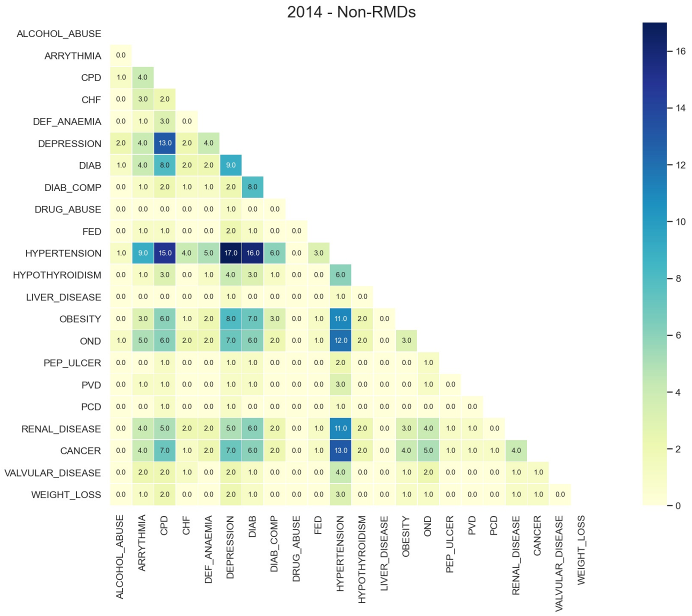

(j) Percentage of patients with RMDs and Comparators

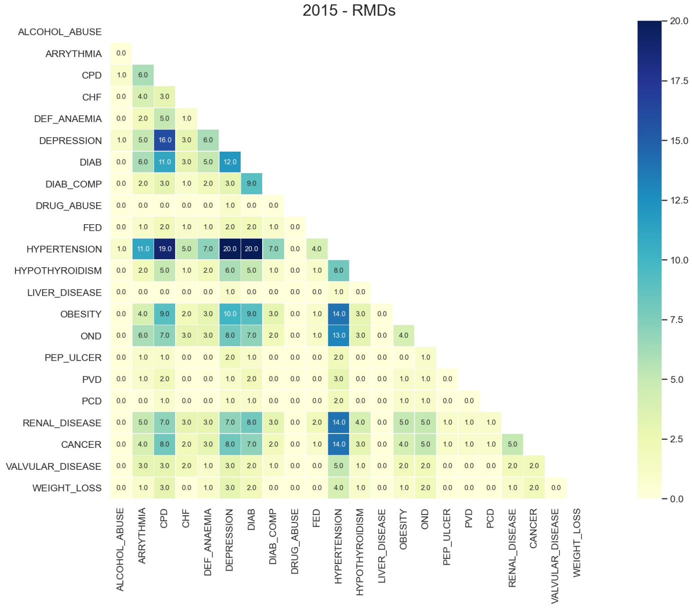

(k) Percentage of patients with RMDs and Comparators

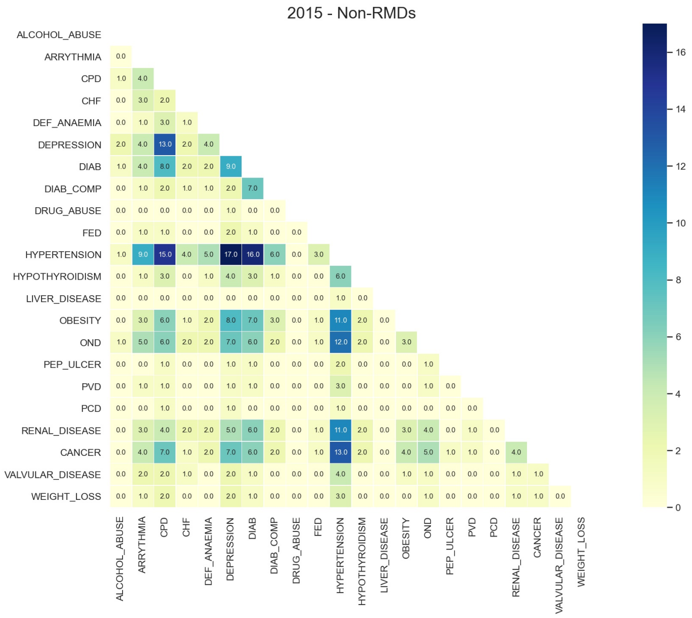

(l) Percentage of patients with RMDs and Comparators

Figure 5. Proportion of patients with RMDs an Comparators

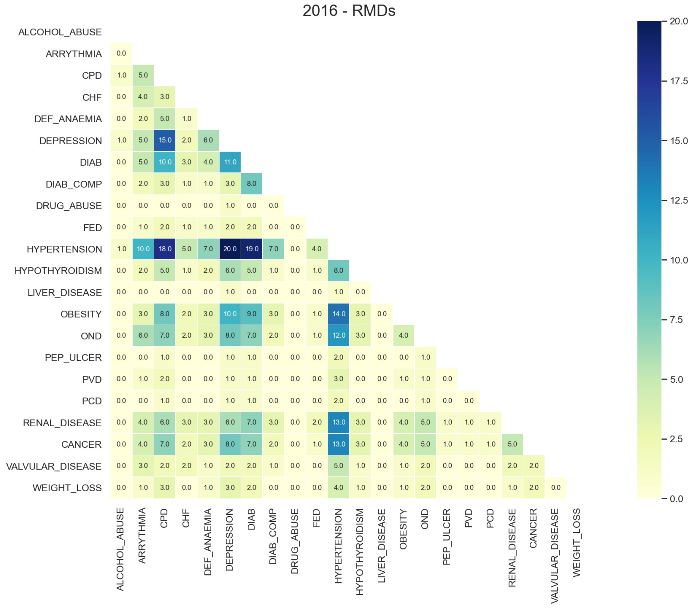

(m) Percentage of patients with RMDs and Comparators

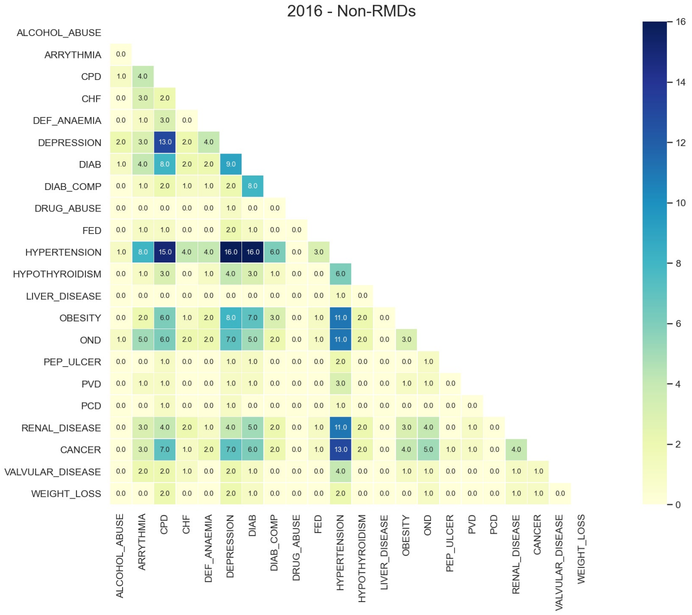

(n) Percentage of patients with RMDs and Comparators

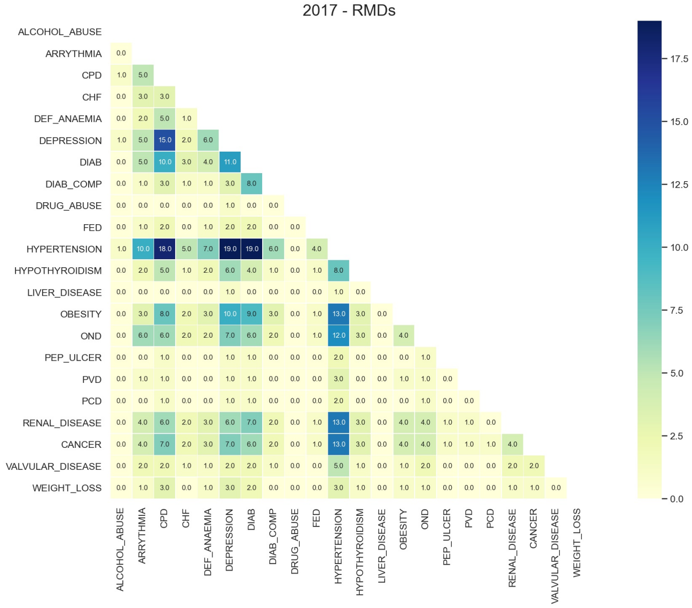

(o) Percentage of patients with RMDs and Comparators

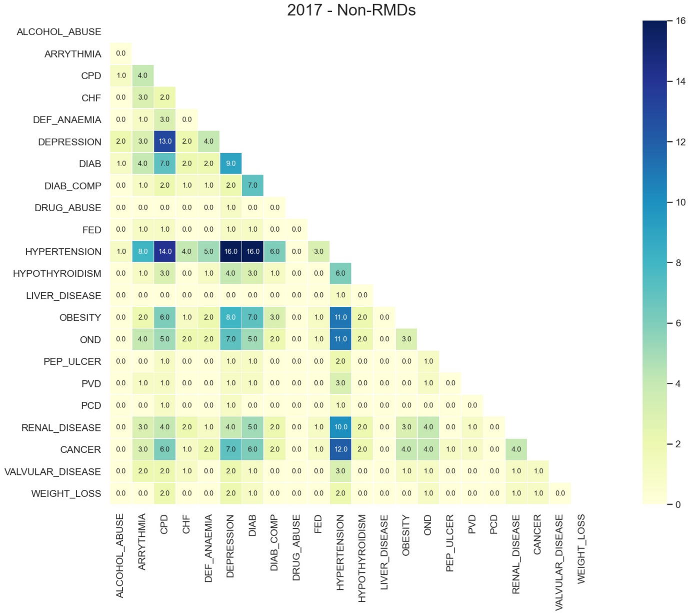

(p) Percentage of patients with RMDs and Comparators

Figure 5. Proportion of patients with RMDs and Comparators

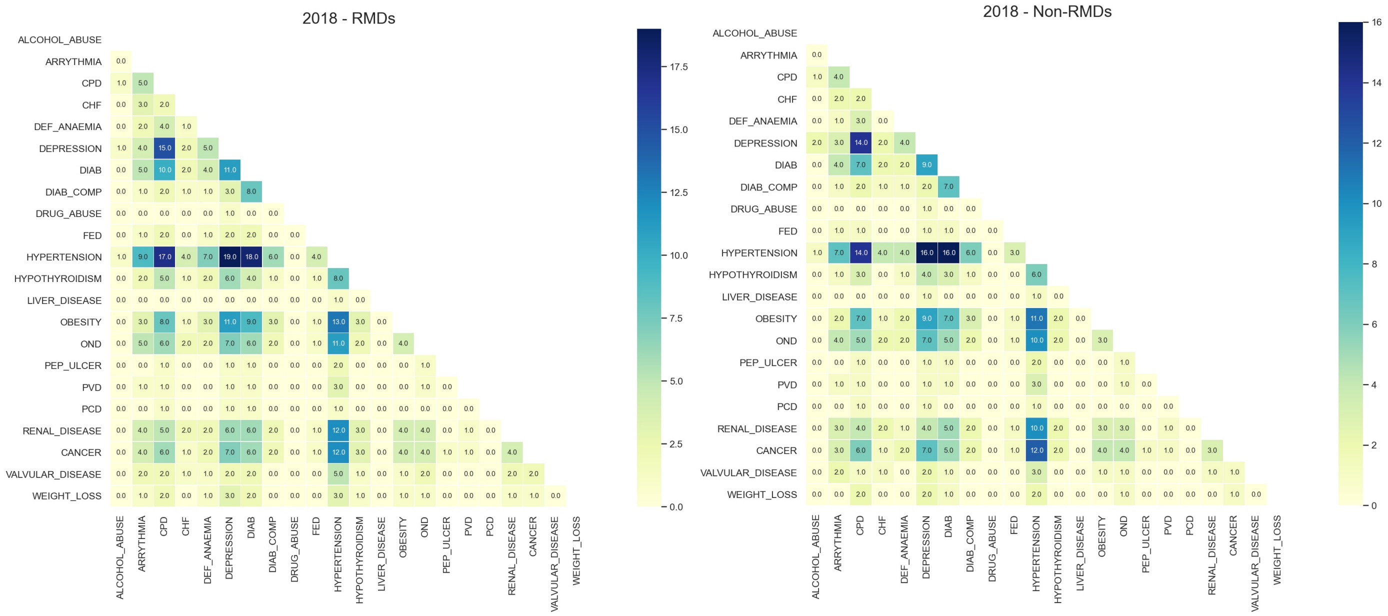

(q) Percentage of patients with RMDs and Comparators **(r)** Percentage of patients with RMDs and Comparators

Figure 5. Proportion of patients with RMDs and Comparators

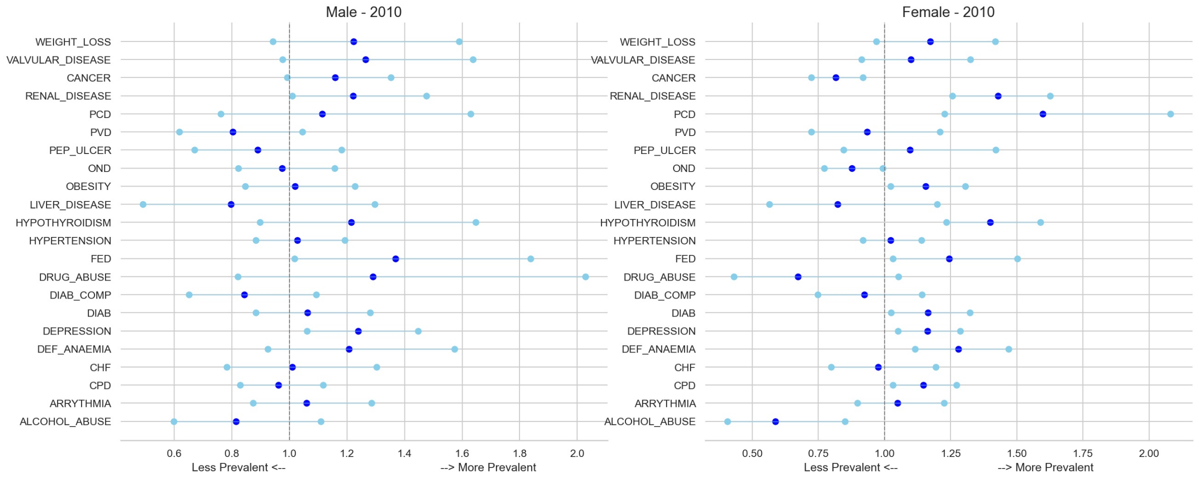

(a) ORs for association of RMDs with comorbidities by gender in 2010

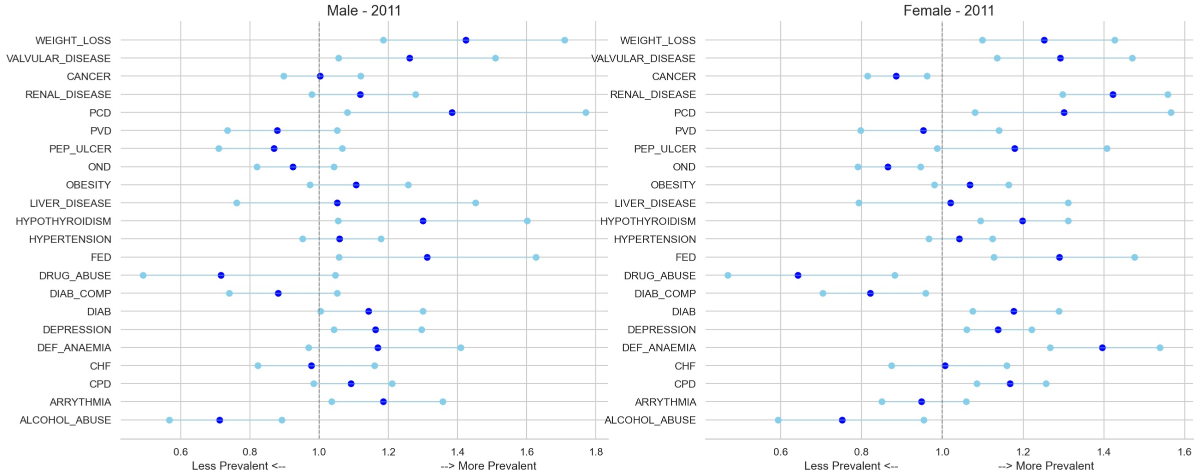

(b) Percentage of patients with RMDs and Comparators

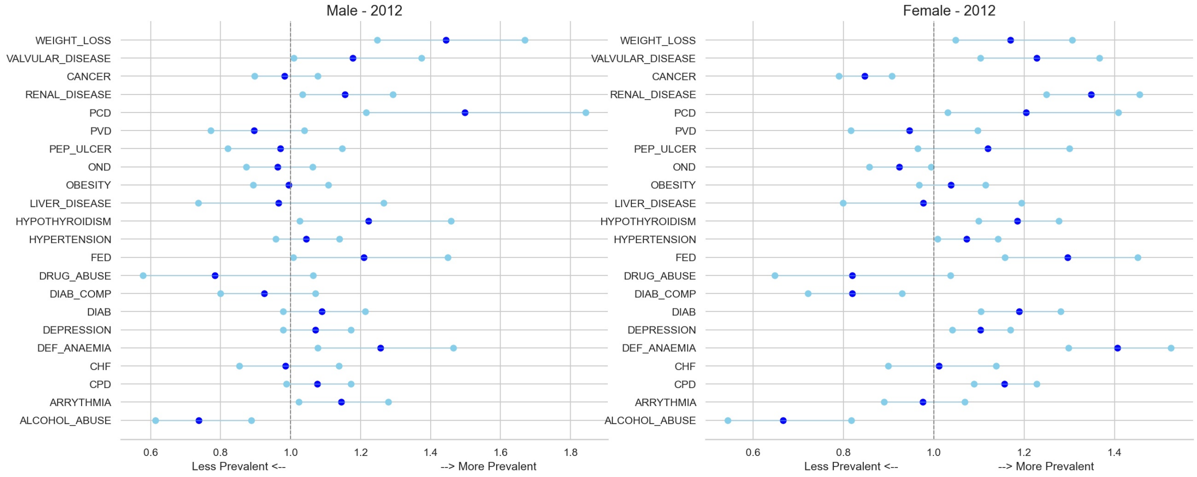

(c) Percentage of patients with RMDs an Comparators

Figure 5. ORs for association of RMDs with comorbidities by sex in 2012-2013

Jalali-najafabadi F, *et al. BMJ Open* 2024; 14:e079169. doi: 10.1136/bmjopen-2023-079169

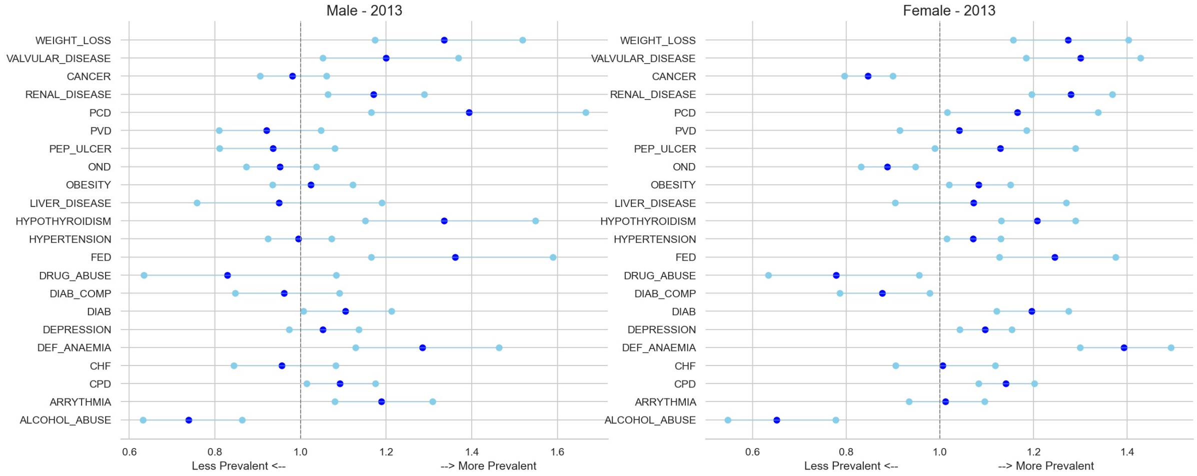

(d) Percentage of patients with RMDs and Comparators

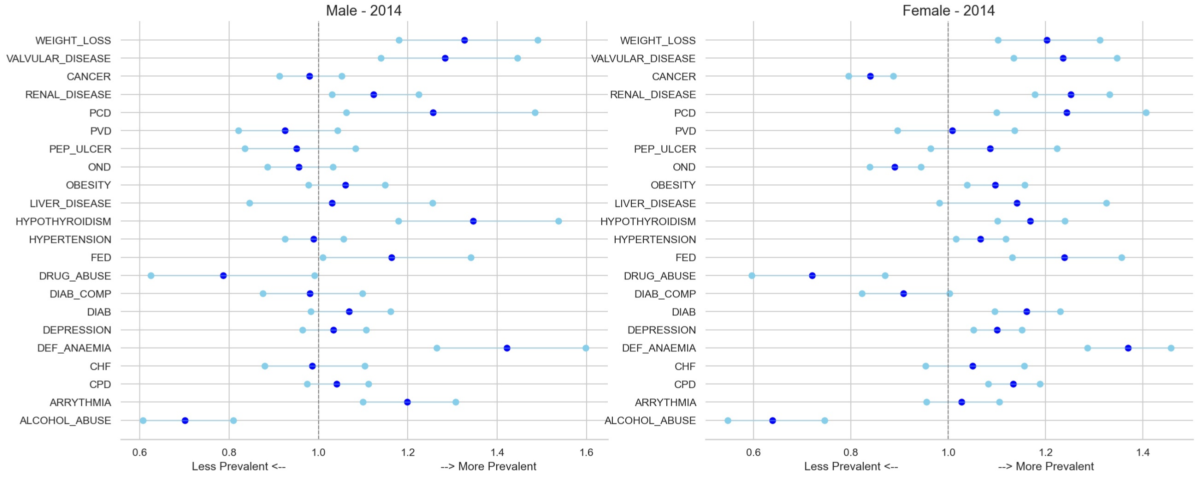

(e) Percentage of patients with RMDs an Comparators

Figure 5. ORs for association of RMDs with comorbidities by sex in 2013-2014

Jalali-najafabadi F, *et al. BMJ Open* 2024; 14:e079169. doi: 10.1136/bmjopen-2023-079169

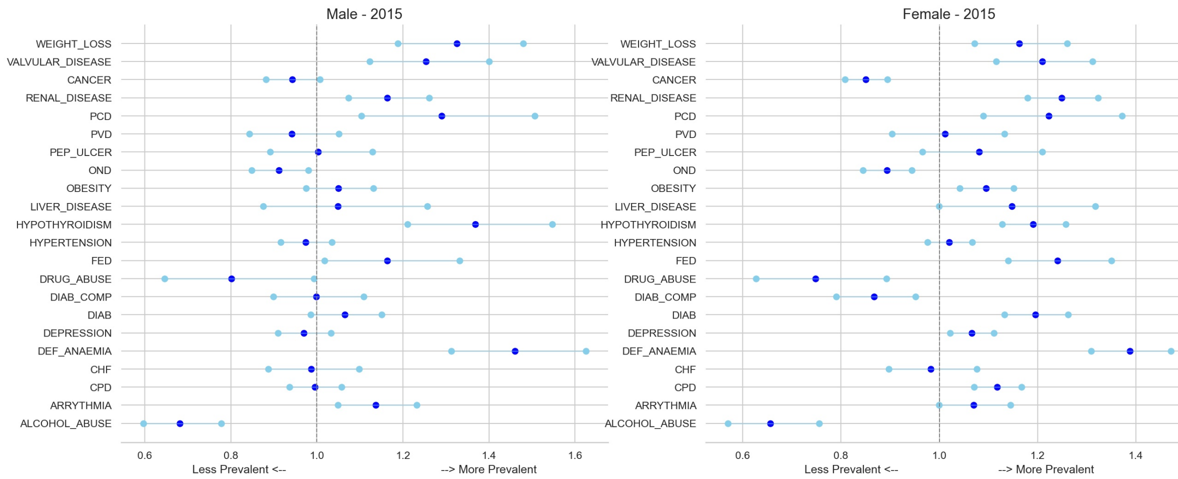

(f) Percentage of patients with RMDs and Comparators

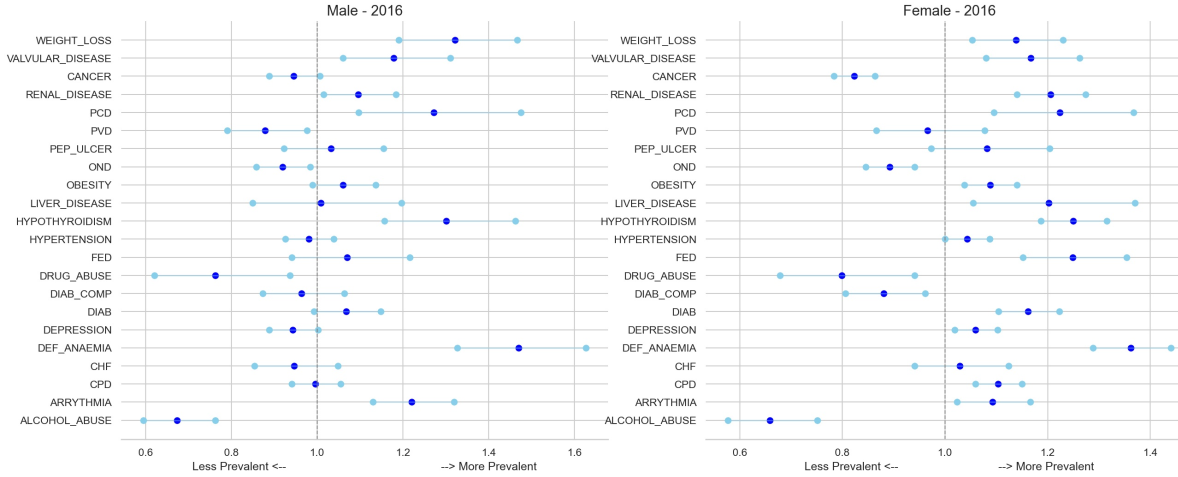

(g) Percentage of patients with RMDs an Comparators

Figure 5. ORs for association of RMDs with comorbidities by sex in 2015-2016

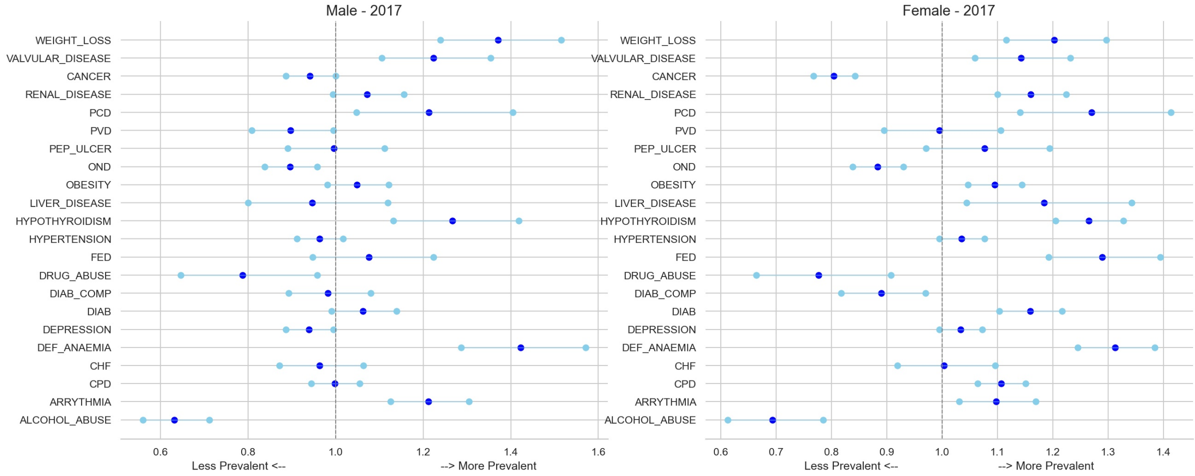

(h) Percentage of patients with RMDs and Comparators

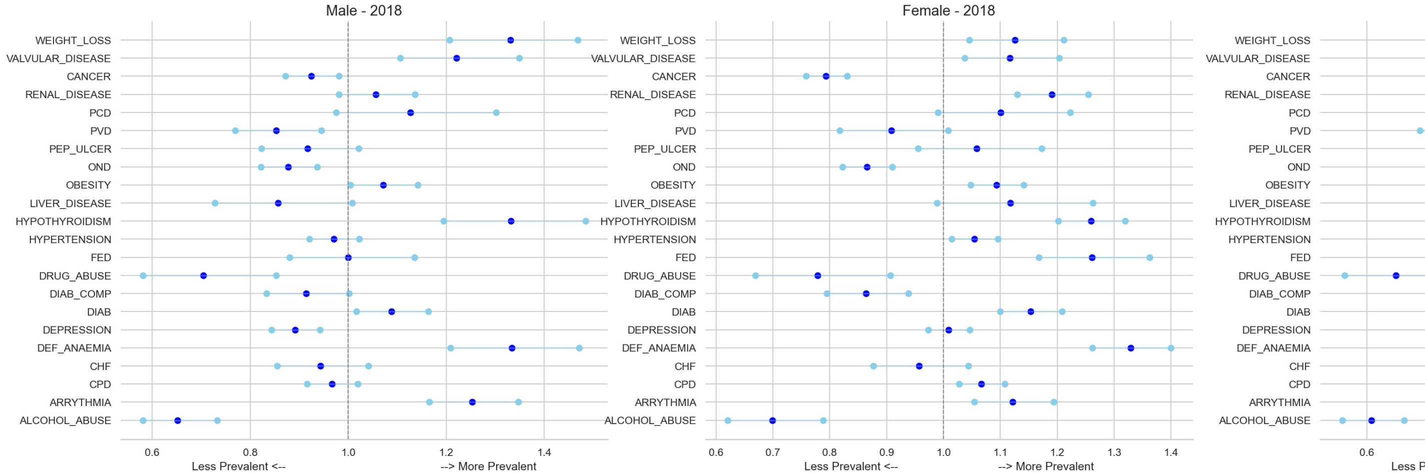

(i) Percentage of patients with RMDs and Comparators

Figure 5. ORs for association of RMDs with comorbidities by sex in 2017-2018

Jalali-najafabadi F, *et al. BMJ Open* 2024; 14:e079169. doi: 10.1136/bmjopen-2023-079169

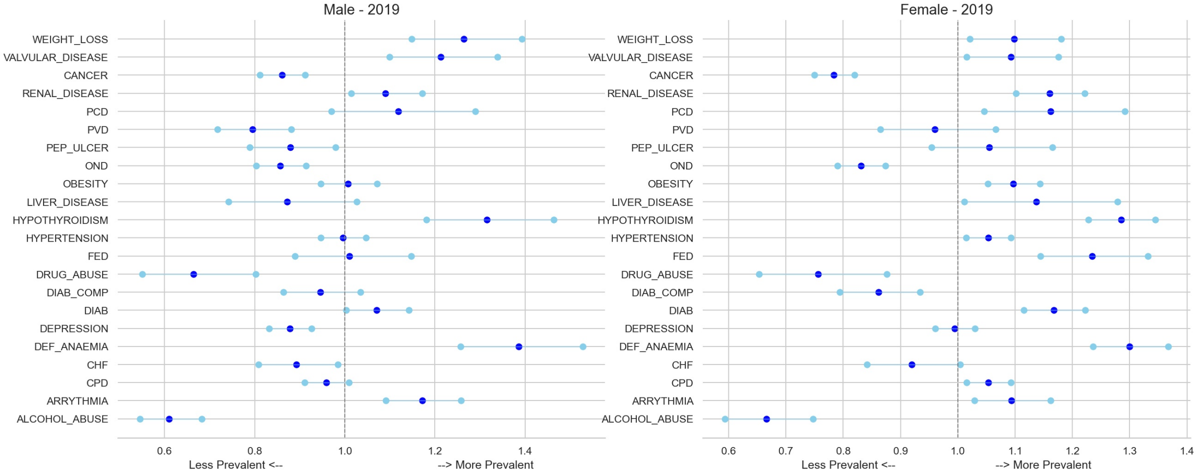

(k) Percentage of patients with RMDs and Comparators

Figure 5. ORs for association of RMDs with comorbidities by sex in 2019

Full description	abbreviations
Rheumatic and musculoskeletal diseases	RMDs
General Practices	GPs
Secure Anonymised Information Linkage	SAIL
Odds ratios	ORs
Trusted Research Environment	TRE
International Classification of Diseases 10th revision	ICD-10 codes
OPCS Classification of Interventions and Procedures version 4	(OPCS-4)
Multiple longterm conditions	MLTCs
Welsh Multi-morbidity e-Cohort	WMC
Alcohol abuse	ALCOHOL_ABUSE
Blood loss anemia	BL_ANAEMIA
Cardiac arrhythmia	CARD_ARRYTHMIA
Chronic pulmonary disease	CPD
Coagulopathy	COAGUL
Congestive heart failure	CHF
Deficiency anemia	DEF_ANAEMIA
Depression	DEPRESSION
Diabetes complicated	DIAB_COMB
Diabetes, uncomplicated	DIAB
Drug abuse	DRUG_ABUSE
FLuid and electrolyte disorders	FED
Hypertension	HYPERTENSION
Hypothyroidism	HYPOTHYROIDISM
Liver disease	LIVER_DIS
Lymphoma	LYMPHOMA
Metastatic cancer	MET_CANCER
Obesity	OBESITY
Other Neurological disorders	OND
Paralysis	PARALYSIS
Peptic ulcer disease excluding bleeding	PEPTIC_ULCER
Periheral vascular disorders	PVD
Psychoses	PSYCHOSES
Pulmonary circulation disorders	PCD
Renal failure	RENAL
Rheumatoid arthritis/collagen vascular diseases	RA
Solid tumor without metastasis	CANCER
Valvular disease	VALVULAR
Weight loss	WEIGHT_LOSS

Table 2. Abbreviations list

Jalali-najafabadi F, *et al. BMJ Open* 2024; 14:e079169. doi: 10.1136/bmjopen-2023-079169
