# Supplementary material for: 10-year multimorbidity patterns among people with and without rheumatic and musculoskeletal diseases: an observational cohort study using linked electronic health records from Wales, UK
Source: BMJ Open. 2024 Jun 19;14(6):e079169. doi: 10.1136/bmjopen-2023-079169 (PMC11191776; doi:10.1136/bmjopen-2023-079169)
Supplement: Supplementary data [file bmjopen-2023-079169supp001.pdf]

GP Data - Full Report

Carys Jones

Date Generated: 2022-03-30

Date Range Applied: 2021-01-01 - 2021-12-31

Tables Used:

- SAILWLGPV.GP_EVENT_CLEANSED_20220101
- SAILWDSDV.AR_PERS_20220131
- SAILREFRV.WELSH_GP_CLUSTER_PRACTICES_20220129
- SAIL0286V.WLGP_CLEAN_GP_REG_BY_PRAC_INCLNONSAIL_MEDIAN_20220201
- SAILX0675V.WDSD_CLEAN_ADD_GEOG_CHAR_LSOA2011_20220328
- SAILREFRV.WALES_GP_PRACTICES_MAIN_SITES_CLUSTERS_20210407

This report describes the General Practice data in SAIL in two ways:

a. the proportion of the Welsh population with a General Practice record in SAIL
b. the proportion of General Practices in Wales that are contributing data to SAIL

Summary Plots

Category	Total Population in SAIL (Percent)
**Complete**	
•	83.6
**Age Group**	
0 to 17	83.9
18 to 64	83.9
65 plus	82.5
**Sex**	
Female	83.7
Male	83.5
**WIMD 2019 Quintile**	
1 (Most Deprived)	87.8
2	86.4
3	81.2
4	78.7
5	85.7
NULL	80.7

Category	Breakdown of SAIL Patients (Percent)	Breakdown of Total Patients (Percent)
**Complete**		
•	100.0	100.0
**Age Group**		
0 to 17	18.8	18.8
18 to 64	60.1	59.9
65 plus	21.0	21.3
**Sex**		
Female	50.1	50.1
Male	49.9	49.9
**WIMD 2019 Quintile**		
1 (Most Deprived)	20.2	19.3
2	19.4	18.8
3	18.3	18.9
4	17.2	18.4
5	19.0	18.5
NULL	5.9	6.1

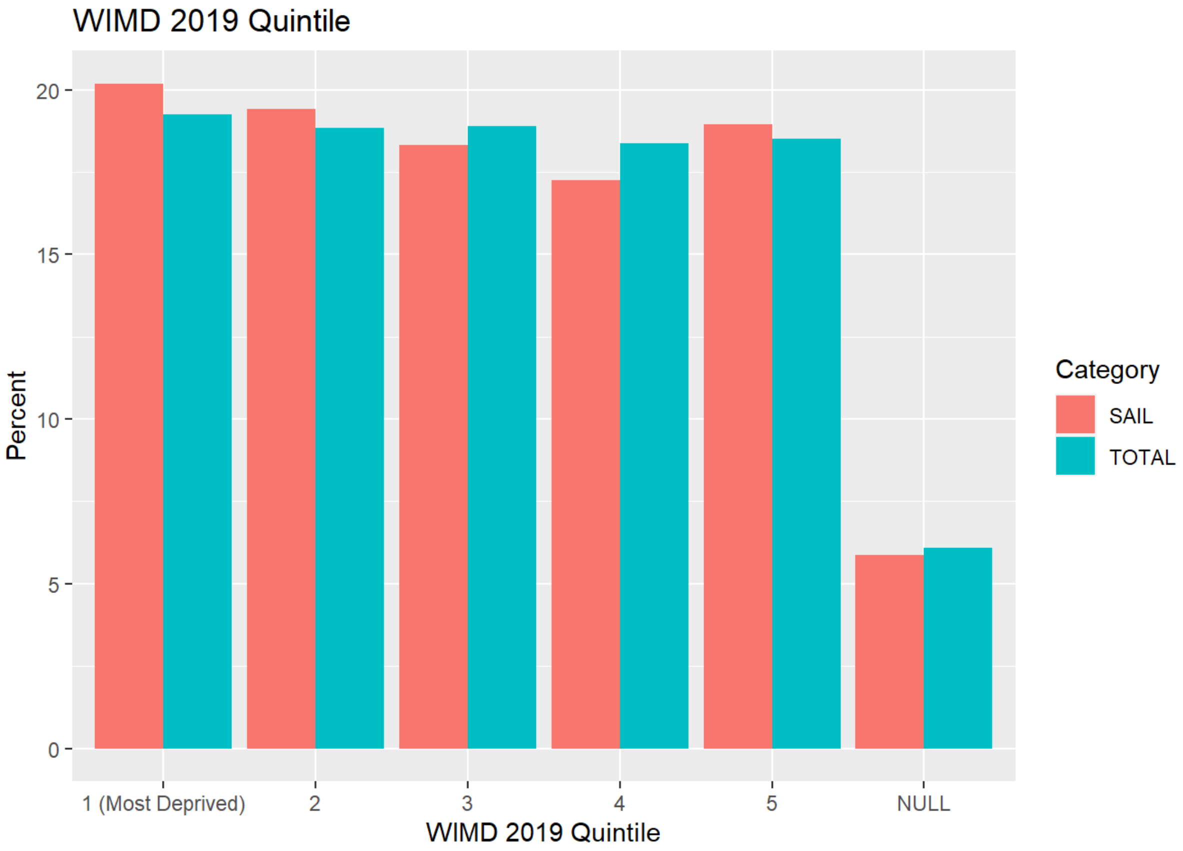
WIMD 2019 Quintile
Percent
20
15
10
5
0
1 (Most Deprived)
2
3
4
5
NULL
WIMD 2019 Quintile
Category
SAIL
TOTAL

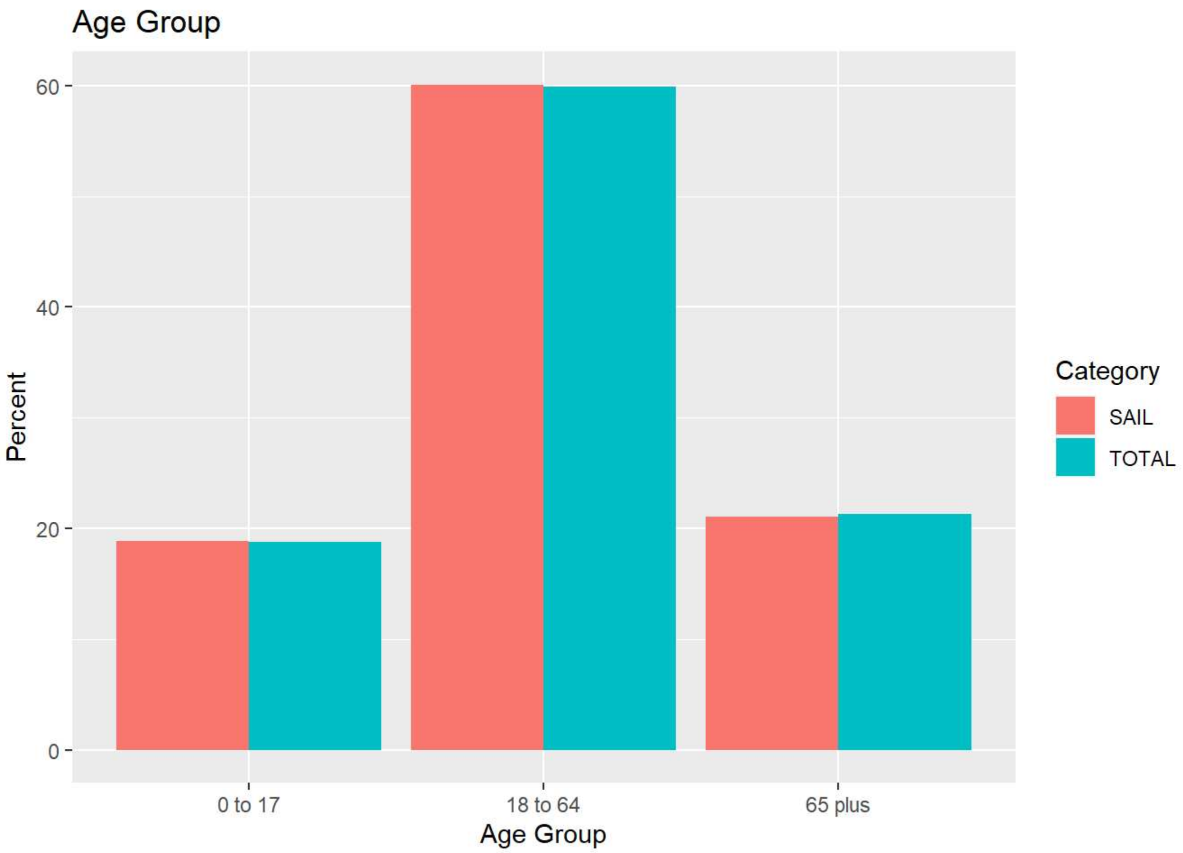
Age Group
Percent
60
40
20
0
0 to 17
18 to 64
65 plus
Age Group
Category
SAIL
TOTAL

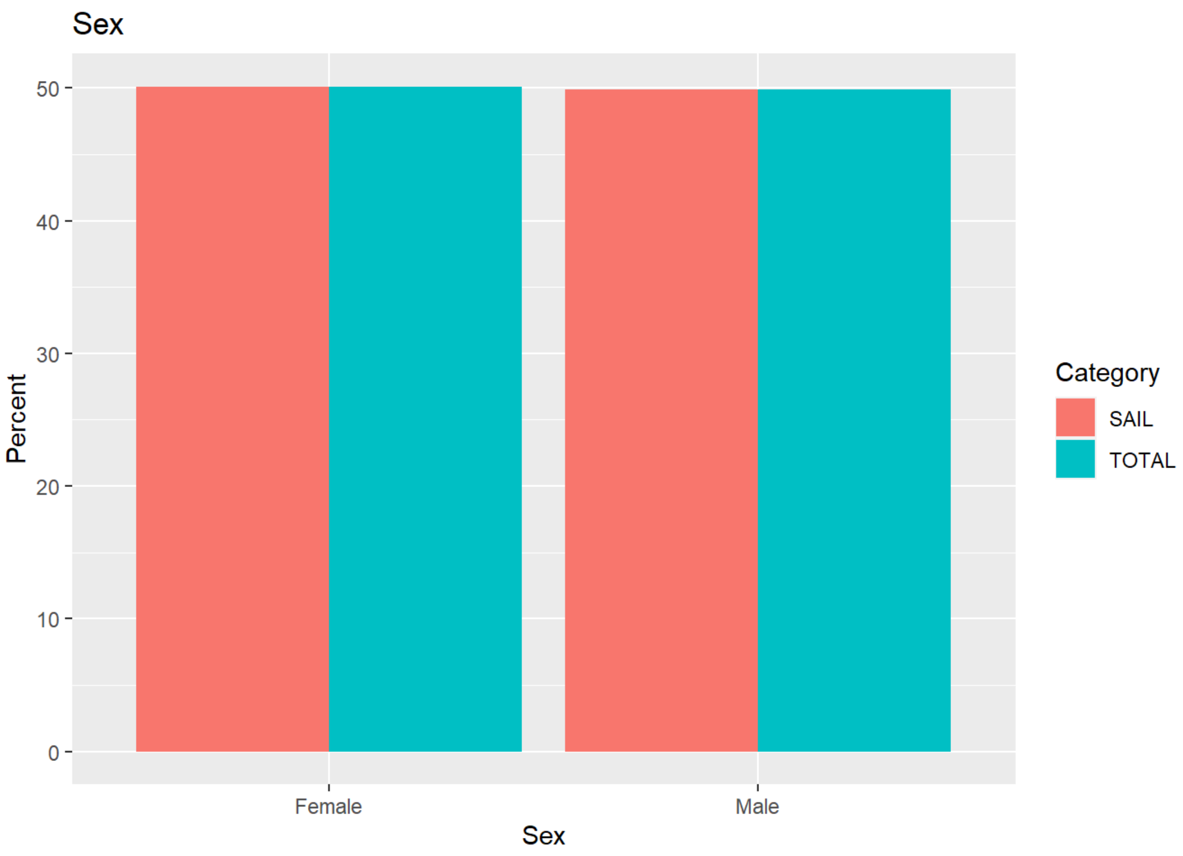

**Health Board**	**SAIL General Practices (Percent by No. of Practices)**
Aneurin Bevan LHB	72.36
Betsi Cadwaladr University LHB	75.75
Cardiff and Vale University LHB	83.60
Cwm Taf Morgannwg University Local Health Board	88.88
Hywel Dda LHB	79.16
Powys Teaching LHB	43.75
Swansea Bay University Local Health Board	95.38
TOTAL	79.80

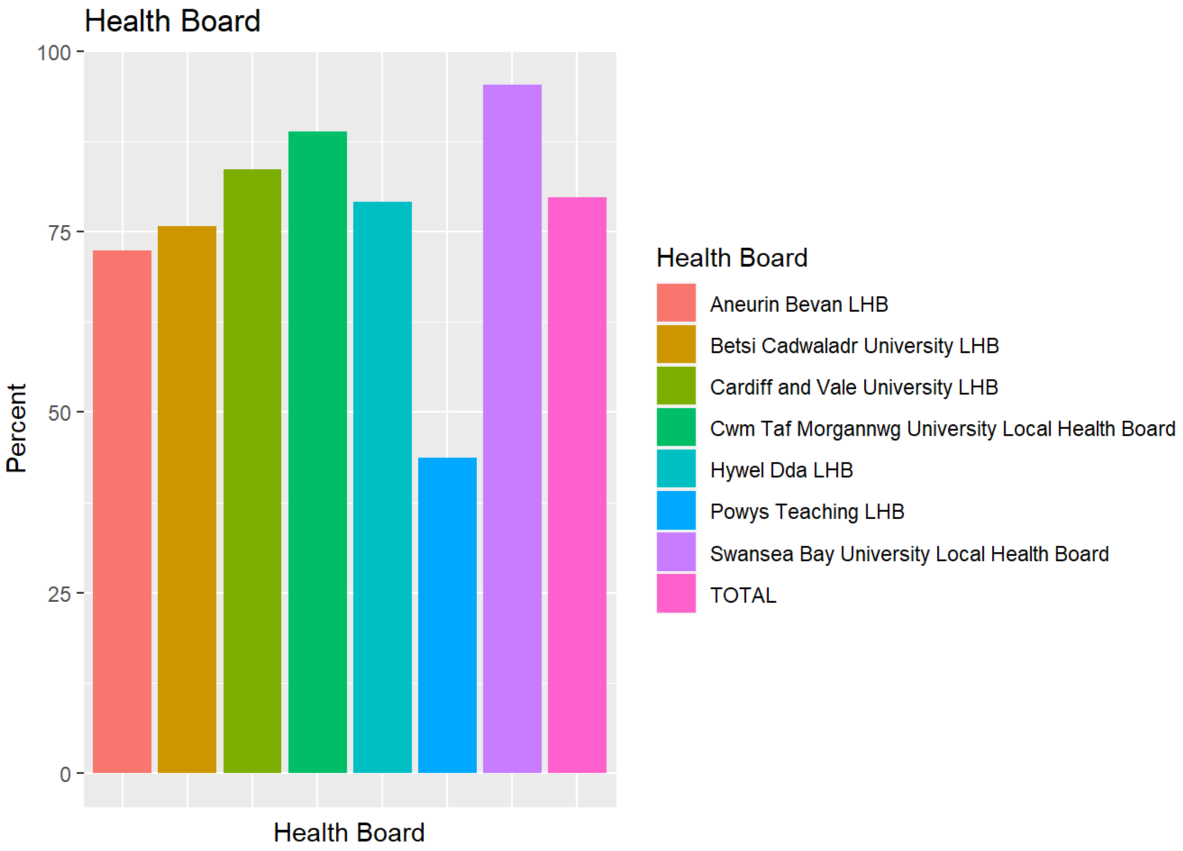
Health Board
100
75
50
25
0
Percent
Health Board
Health Board
Aneurin Bevan LHB
Betsi Cadwaladr University LHB
Cardiff and Vale University LHB
Cwm Taf Morgannwg University Local Health Board
Hywel Dda LHB
Powys Teaching LHB
Swansea Bay University Local Health Board
TOTAL
