# Supplementary material for: 10-year multimorbidity patterns among people with and without rheumatic and musculoskeletal diseases: an observational cohort study using linked electronic health records from Wales, UK
Source: BMJ Open. 2024 Jun 19;14(6):e079169. doi: 10.1136/bmjopen-2023-079169 (PMC11191776; doi:10.1136/bmjopen-2023-079169)
Supplement: Author's manuscript [file bmjopen-2023-079169.draft_revisions.pdf]

BMJ Open

BMJ Open is committed to open peer review. As part of this commitment we make the peer review history of every article we publish publicly available.

When an article is published we post the peer reviewers' comments and the authors' responses online. We also post the versions of the paper that were used during peer review. These are the versions that the peer review comments apply to.

The versions of the paper that follow are the versions that were submitted during the peer review process. They are not the versions of record or the final published versions. They should not be cited or distributed as the published version of this manuscript.

BMJ Open is an open access journal and the full, final, typeset and author-corrected version of record of the manuscript is available on our site with no access controls, subscription charges or pay-per-view fees (http://bmjopen.bmj.com).

If you have any questions on BMJ Open's open peer review process please email info.bmjopen@bmj.com

BMJ Open

10 years multi-morbidity patterns among people with and without Rheumatic and Musculoskeletal diseases using Wales primary care data

Journal:	*BMJ Open*
Manuscript ID	bmjopen-2023-079169
Article Type:	Original research
Date Submitted by the Author:	23-Aug-2023

Complete List of Authors:	Jalali-najafabadi, Farideh; The University of Manchester,<br>Bailey, Rowena; Swansea University Medical School, Population Data Science<br>Lyons, Jane; Swansea University Medical School,<br>Akbari, Ashley; Swansea University Medical School,<br>Badhafari, Tthamer; University of Manchester<br>Azadbakht, Narges; The University of Manchester<br>Rafferty, James; Swansea University Medical School<br>Watkins, Alan; Swansea University, College of Medicine<br>Martin, Glen; University of Manchester Institute of Population Health, Farr Institute<br>Bowes, John; The University of Manchester<br>Lyons, Ronan; University of Wales Swansea, Swansea Clinical School<br>Barton, Anne; University of Manchester, Centre for Musculoskeletal Research<br>Peek, N; The University of Manchester, Division of Informatics, Imaging and Data Science

Keywords:	Electronic Health Records, RHEUMATOLOGY, Epidemiology < ONCOLOGY

SCHOLARONE™
Manuscripts

I, the Submitting Author has the right to grant and does grant on behalf of all authors of the Work (as defined in the below author licence), an exclusive licence and/or a non-exclusive licence for contributions from authors who are: i) UK Crown employees; ii) where BMJ has agreed a CC-BY licence shall apply, and/or iii) in accordance with the terms applicable for US Federal Government officers or employees acting as part of their official duties; on a worldwide, perpetual, irrevocable, royalty-free basis to BMJ Publishing Group Ltd ("BMJ") its licensees and where the relevant Journal is co-owned by BMJ to the co-owners of the Journal, to publish the Work in this journal and any other BMJ products and to exploit all rights, as set out in our licence.

The Submitting Author accepts and understands that any supply made under these terms is made by BMJ to the Submitting Author unless you are acting as an employee on behalf of your employer or a postgraduate student of an affiliated institution which is paying any applicable article publishing charge ("APC") for Open Access articles. Where the Submitting Author wishes to make the Work available on an Open Access basis (and intends to pay the relevant APC), the terms of reuse of such Open Access shall be governed by a Creative Commons licence – details of these licences and which Creative Commons licence will apply to this Work are set out in our licence referred to above.

Other than as permitted in any relevant BMJ Author's Self Archiving Policies, I confirm this Work has not been accepted for publication elsewhere, is not being considered for publication elsewhere and does not duplicate material already published. I confirm all authors consent to publication of this Work and authorise the granting of this licence.

For peer review only

10 years multi-morbidity patterns among people with and without Rheumatic and Musculoskeletal diseases using Wales primary care data

Farideh Jalali-najafabadi[1+], Rowena Bailey[2], Jane Lyons[2], Ashley Akbari[2], Thamer Ba Dhafari[3], Narges Azadbakht[3], James Rafferty[2], Alan Watkins[2], Glen P. Martin[3], John Bowes[1,4], Ronan A. Lyons[2], Anne Barton[1,4], and Niels Peek[3,5,6]

[1]Centre for Genetics and Genomics Versus Arthritis, Centre for Musculoskeletal Research, Faculty of Biology, Medicine and Health, Manchester Academic Health Science Centre, The University of Manchester, Manchester, UK.
[2]Population Data Science, Health Data Research-UK, Faculty of Medicine, Health and Life Science, Swansea University, Swansea, UK.
[3]Division of Informatics, Imaging and Data Science, School of Health Sciences, The University of Manchester, Manchester, UK.
[4]NIHR Manchester Musculoskeletal Biomedical Research Unit,Central Manchester NHS Foundation Trust, Manchester Academic Health Science Centre, UK.
[5]Alan Turing Institute, London, UK.
[6]NIHR Manchester Biomedical Research Centre, Manchester University NHS Foundation Trust, Manchester Academic Health Science Centre, UK.

(+) Corresponding author. Email address: [+]farideh.jalali@manchester.ac.uk

For peer review only

ABSTRACT

Objectives

To compare patterns of multi-morbidity between people with and without rheumatic and musculoskeletal diseases (RMDs) and to describe how these patterns change by age and sex over time, between 2010 and 2019.

Participants

103,426 people with RMDs and 2.9 million comparators registered in 395 Wales general practices. Each patient with RMDs aged 0 − 100 years between January 2010 and December 2019 registered in Clinical Practice Research Welsh practices was matched to up to five comparators without RMDs on age, gender and general practice (GP) code.

Primary outcome measures

Prevalence of 29 Elixhuaser in people with RMDs and comparators categorised by age, gender and GP practices. Odds for association between RMDs diagnosis and comorbidities versus compactors were computed.

Methods

Each patient with an RMD diagnosis, were matched with comparators without an RMDs diagnosis by age, sex and GP code to create the RMD cohort and the comparator cohort, respectively. Conditional logistic regression models were fitted to calculate differences (OR, 95% CI) in associations with morbidity between cohorts.

Results

The most prevalent comorbidities were cardiovascular risk factors, hypertension and diabetes. Having an RMD diagnosis was significantly associated with higher odds for many conditions including deficiency anaemia (OR 1.39, 95% CI(1.32-1.46)), hypothyroidism (OR 1.34, 95% CI(1.19-1.50)), diabetes (OR 1.17, 95% CI(1.11-1.23)), fluid and electrolyte disorders (OR 1.27, 95% CI(1.17-1.38)). RMDs have a higher proportion of multi-morbidity (2 or more conditions in addition to RMDs) compared to non-RMDs (81% and 73% respectively 2019) and the mean number of co-morbidities was higher in woman from the age of 25 and 50 in men than in non-RMDs group and were higher in females than males.

Conclusion

People with RMDs are approximately 1.5 times as likely to have multi-morbidity as the general population and provide a high-risk group for targeted intervention studies.

Key words

electronic health records, primary care, multi-morbidity, rheumatic and musculoskeletal diseases

For peer review only

1 Rheumatology key messages

What is already known about this subject

People with RMDs are known to be living with the burden of additional chronic conditions and are at a higher risk of multi-morbidity than people without RMDs. Indeed, RMDs appear to form a principal component of certain multi-morbidity clusters.

What does this study add?

This is the first study to provide a comprehensive assessment of a range of morbidities across the life-course, including under-reported conditions, in patients with RMDs patients compared to those without RMDs using a primary-care database of electronic health records from the resident population in Wales. The mean number of co-morbidities was higher in patients with RMDs than those without and, in females, the higher mean number of co-morbidities was observed from the age of 25.

How might this impact on clinical practice or future developments in multi-morbidity?

The issue of increased risk for multi-morbidity among RMD patients makes healthcare decision-making more difficult than it is for people who do not have RMDs conditions. These findings highlight where resources might be best directed to provision care, design preventative or allocate increased diagnostic and screening services for patients with RMDs

2 Strengths and limitations

- To our knowledge, this is the first study to present analysis using different approaches in large population of Wales in people with and without RMDs using routine primary care data.

- Our large study cohort provides sufficient statistical power to calculate overall and age- and sex-specific prevalence rates of individual co-morbidities between 2010 and 2019.

- In addition to the recognised and frequently reported co-morbidities such as cardiovascular risk factors, our findings highlight a number of co-morbidities such as deficiency anaemia, fluid and electrolyte disorders that are highly prevalent in people with RMDs but which are under-reported in literature.

- This study depends on diagnoses reported in electronic health records, it is possible that patients with undiagnosed conditions, such as those who did not visit a general practitioner, were missed.

- We have analysed only pairs of diseases, but higher-order combinations of diseases probably interact in more complicated ways. In terms of the application of these findings, it should be noted that the prevalence measure was based on Elixhauser conditions; there is no established gold standard list of diagnoses used to define multi-morbidity and definitions of multi-morbidity vary in the medical literature.

For peer review only

3 Introduction

Multi-morbidity is a global public health concern and refers to the existence of multiple long-term conditions (MLTCs) in a single individual[1–3]. The prevalence of multi-morbidity appears to have increased in many regions of the world over the past 20 years. It is anticipated to continue to rise and evidence from high-income countries suggests that while multi-morbidity is highly prevalent in older populations (typically those over 65 years of age), it also affects younger people[4]. Epidemiologically, the term co-morbidity is used when estimating co-occurring conditions among populations with an index condition and the term ‘multi-morbidity’ is used when estimating the prevalence of co-occurring conditions among the wider population[5,6].

Rheumatic and musculoskeletal diseases (RMDs) comprise over 200 diseases and syndromes, which can affect the joints, connective tissue, cartilage, and tendons; they are usually progressive, associated with pain and are among the most common chronic non-communicable diseases. The prevalence of disability, premature mortality and physical impairment[7,8] in RMDs is increased meaning they are a major burden on individuals, social care systems and health systems and this burden has been recognised by WHO and the United Nations[9]. For example, in the UK, an estimated 8.75 million people have sought treatment for osteoarthritis, the equivalent to a third of all people over 45 years of age[10]. In addition, an estimated 1 in 5 men and 1 in 2 women aged over 50 years will sustain a low-trauma fracture as a result of osteoporosis[11–13] with the situation set to worsen with ageing populations. Previous studies have found that people with RMDs are often living with additional chronic conditions[14–16] and RMDs appear to form a principal component of certain multi-morbidity clusters[17–19]. Certainly, a substantial proportion of people with RMDs now live with multi-morbidity[20] but most current clinical guidelines have ‘single condition’ model of care without considering multimorbidity. Multimorbidity has a number of negative outcomes, including disability and death, reduced quality of life, and higher healthcare utilisation and expenses[21].

Several studies have described co-morbidities in the RMDs population[22,23], but it remains to be determined if the increased co-morbidity in patients with RMDs relates to specific co-morbid conditions, occurs across the life-course or differs by sex. The longitudinal nature of the (SAIL) Databank in Wales, UK enables for the analysis of information on diagnoses and consultations at multiple time points for RMDs in general[24,25]. Therefore, using a large electronic linked primary care data set on the resident population in Wales, this study aimed to examine the patterns of 28 chronic conditions in people with and without RMDs over a 10-year study period. We further stratified the co-morbidities prevalence rates by age, gender, and assessed the association between diagnosis of RMDs and the presence of a co-morbidity and described the patterns of all two coexisting co-morbidities.

4 Methods

4.1 Data Sources

Data used for this study were accessed via the Secure Anonymised Information Linkage (SAIL) Databank (www.saildatabank.com). SAIL is a state of the art, remotely accessible, privacy-protecting Trusted Research Environment (TRE), accredited under the Digital Economy Act. SAIL contains linked anonymised population-scale data sources at an individual person-level, household-level and multiple-ecological-levels, for the population of Wales[24,25]. SAIL Databank sources include inpatient, outpatient, critical care, emergency department, mortality, birth register and demographic information for people that use healthcare services in Wales.

Morbidities were defined based on Elixhuaser categories, a well-established scheme distinguishing 30 classes of chronic diseases. HIV was not included as data is not available in SAIL due to data privacy policies. International Classification of Diseases 10th revision (ICD-10) codes, OPCS Classification of Interventions and Procedures version 4 (OPCS-4) codes and primary care Read version 2 (CTV2) codes were used to identify Elixhauser morbidities in healthcare records. The Elixhauser coding algorithms used to establish the presence of each Elixhauser morbidity were downloaded from supplementary of Metcalfe et al[26] and are listed in Table 1 supplementary and resulted in a set of 28 comorbidities and Rheumatoid arthritis/collagen vascular diseases (RMDs).

4.2 Participants

Study participants were selected from the Welsh Multi-morbidity e-Cohort (WMC), which consists of 2,902,101 million people living in Wales on the 1st January 2000 with follow-up until 31st December 2019 and was created to provide accessible research ready data for the understanding of multi-morbidity[27].

4.2.1 RMDs cohort

Patients aged 0 – 100 years with a first diagnostic RMDs medical code between 1st January 2010 and 31st December 2019 were identified in the WMC. The index date was defined as the earliest date of RMDs diagnosis. Patients were followed until movement out of a SAIL providing practice, death or end of the study observation period (31st December 2019). Our sample

For peer review only - http://bmjopen.bmj.com/site/about/guidelines.xhtml

was restricted to patients having >= 1 year follow-up for each matched case and comparator (e.g., cases and comparators in the matched cohort in 2019).

4.2.2 Non-RMDs cohort

We performed a matched, retrospective cohort study within the WMC. Within each year, up to five patients without RMDs (comparators) were identified and matched to each patient with RMDs on the following variables at the RMDs diagnosis date: age (±2 years difference), sex and general practice code. We required each comparator to be enrolled with the same GP on the index date of the accompanying patient with RMDs and assigned the same index date.

4.2.3 Outcomes

Overall, the presence of morbidities was defined as conditions recorded before and after RMDs diagnosis date (index date) and up to the end of the calendar year. We defined multi-morbidity as the presence of at least two conditions from the aforementioned Elixhauser comorbidity index, excluding RMDs as one of the conditions, to avoid bias in our results towards greater multi-morbidity in the RMDs cohort.

4.3 Statistical analyses

Statistical analyses were used to map co-morbidity data obtained from WMC primary care data sources using four approaches: the trends over time for specific Elixhauser conditions were explored using prevalence rates of multi-morbidity. The prevalence rate of comorbidities was stratified by age and sex. The patterns of co-morbidities were examined using percentage of pairwise morbidity conditions and logistic regression models to derive odds for the presence/absence of each co-morbidity between groups.

- Annual prevalence rates and 95% CI for each co-morbidity over the 10-year period were calculated separately for each cohort by dividing the number of persons with diagnosed with the co-morbidity in or before the study year by the total number of persons contributing throughout the study year.
- The prevalence rates were also calculated stratified by (1) 20-year age groups, (2) sex with RMDs and matched comparator patients in the last study year.
- Patterns of coexisting pairs of morbidities were compared between cohorts. The percentage of patients with both morbidities was calculated for each pair of conditions for each cohort.
- Conditional logistic regression models adjusted for age and sex were fitted to estimate OR and 95% CI for the differences between cohorts for associations with Elixhauser co-morbidities. Regression models restricted to males or females were also fitted to calculate sex-specific estimates.

All analyses we conducted using Python software 3.7. We used the STROBE cohort checklist when writing our report[28]

5 Results

5.1 Patient population

Of 2,902,101 million individuals enrolled in WMC between 2000-2019, we identified 102,042 who fulfilled our eligibility criteria for people with RMDs. Patients with RMDs were matched to 1,637,632 million people without RMDs (comparators). Figure 1 shows the flow chart for identification of patients with and without RMDs registered in linked general practices.

5.2 Annual prevalence of co-morbidities

We investigated the prevalence of 28 co-morbidities overall, but results for 7 co-morbidities with number less than 5 cases have been not reported due to statistical disclosure control in SAIL Databank. The annual prevalence rates of all remaining 21 co-morbidities between 2010-2019 are presented in Figure 2. The most common co-morbidities were cardiovascular risk factors, hypertension and diabetes without complications. Other neurological disorders (OND), increased sharply over the study period in people with RMD’s (from 0.9% (95% CI 0.56% to 1.24%) in 2010 to 10.2% (95% CI 9.86% to 10.62%) in 2019 in patients with RMDs; and from 0.7% (95% CI 0.56% to 0.83%) in 2010 to 8.6% (95% CI 8.41% to 8.73%) in 2018 in comparators). Drug abuse, peptic ulcer disease and liver diseases were the least common chronic conditions in both people with and without RMDs. The prevalence rates (and confidence intervals) of all remaining 21 comorbidities in RMDs and comparators in 2010 and 2019 are presented in Table 2 supplementary.

For peer review only - http://bmjopen.bmj.com/site/about/guidelines.xhtml

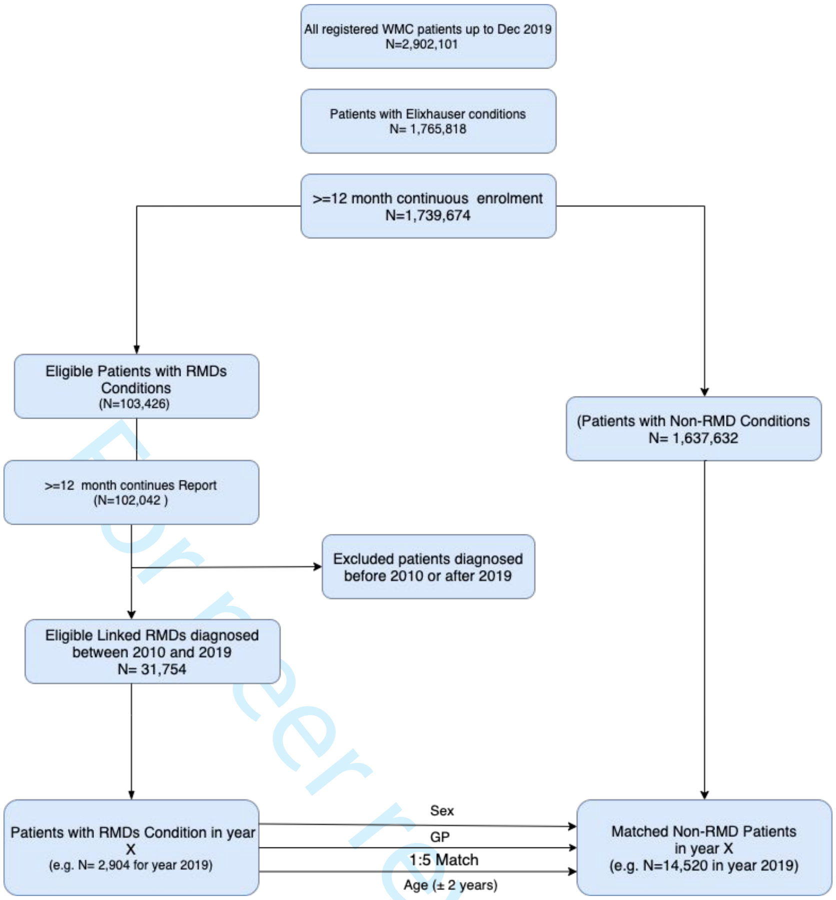

Figure 1. Flow chart for identification of patients with and without RMDs registered in WMC, using year 2019 as an example. WMC, Welsh Multi-morbidity e-Cohort (WMC); Rheumatic and musculoskeletal diseases (RMDs)

5.3 Number of comorbidities, age-specific, sex-specific

2019, Patients with RMDs had a higher proportion of multi-morbidity (2 or more conditions in addition to RMDs) compared to the comparator cohort (81% and 73% respectively) Figure 3(a), and the mean number of comorbidities in increased with age for both groups (Figure 3(b)). Interestingly, the mean number of co-morbidities (Figure 3(b)-2019 and Figure (2) other years supplementary) was higher in females between the ages of 25-100 compared to the comparator group but in males, the significant increase in mean number of co-morbidities was noted only from the age of 50. Sex-specific percentage prevalence of Elixhauser comorbidities at the study end in 2019 in patients with RMDs and comparators are presented in Figure 2 supplementary. The prevalence of hypertension was nearly 4% higher in females and males than comparators (Figure 2 supplementary).

5.4 Proportion of patients with two co-morbidities

The most common pairs of coexisting morbidities across both the RMDs and comparator groups in 2019 were combinations of hypertension, chronic pulmonary disease, depression, diabetes and obesity. The proportion of patients with two co-morbidities was higher in people with RMDs compared with comparators, and increased between 2010 and 2019. The pattern of all two coexisting co-morbidities in people with and without RMDs in 2019 are illustrated in heatmap Figure 4. The dark blue colour in the heatmap is determined by a higher percentage of patients with the two co-morbidities in the same plot and not between separate plots. The plot for other years is presented in supplementary Figure 3.

5.5 Odds ratios (95% CI) for associations of RMDs with specific co-morbidities in annual cohorts 2010-2019

Patients with RMDs were at least 30% more likely to have: deficiency anaemia (OR 1.39, 95% CI 1.32 to 1.46); hypothyroidism (OR 1.34, 95% CI 1.19 to 1.50); pulmonary circulation disorders (OR 1.39, 95% CI 1.12 to 1.73); renal disease (OR 1.35, 95% CI 1.22 to 1.50) and weight loss (OR 1.30, 95% CI 1.17 to 1.45). RMDs patients were also 27% more likely to have both fluid and electrolyte disorders (OR 1.27, 95% CI 1.17 to 1.38) and valvular disease (OR 1.27, 95% CI 1.15 to 1.41), 17% more

For peer review only - http://bmjopen.bmj.com/site/about/guidelines.xhtml

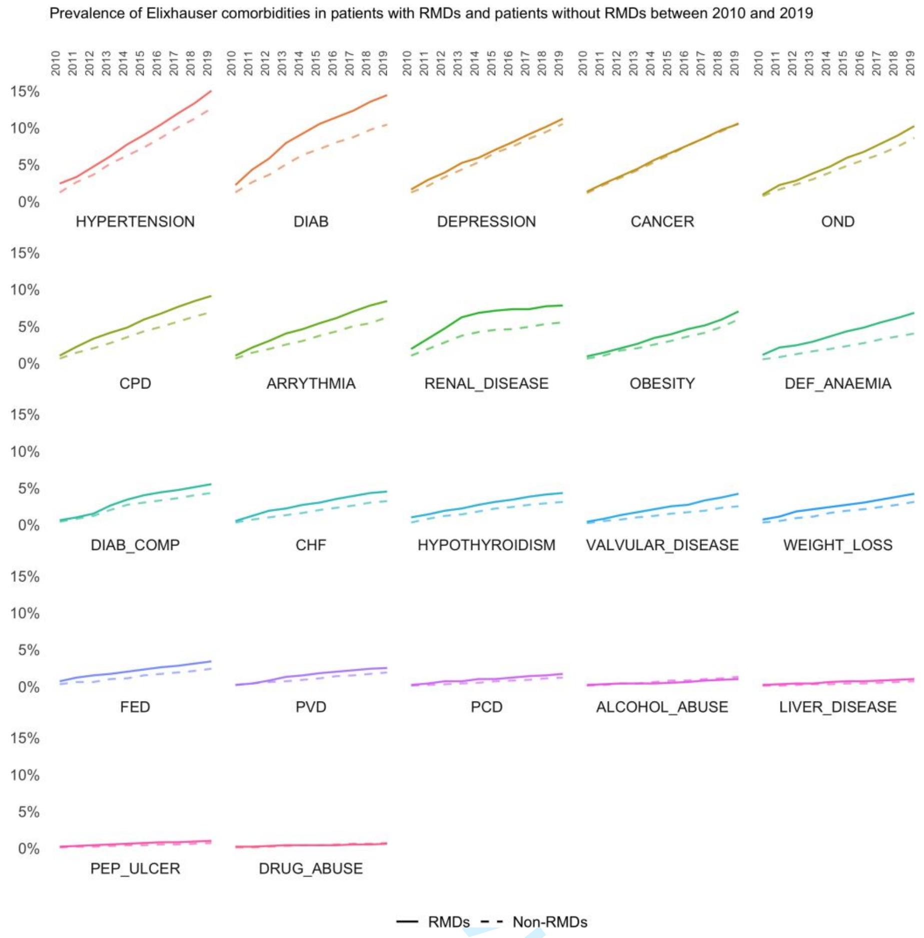

Figure 2. Annual prevalence of Elixhauser co-morbidities in RMDs cases and matched comparators 2010-2019

likely to have diabetes (OR 1.17, 95% CI 1.11 to 1.23), and 9% more likely to have a diagnosis of obesity (OR 1.09, 95% CI 1.05 to 1.13) when compared people without RMDs. A cancer diagnosis was 19% less likely for the RMDs group. Similarly, other neurological disorders, diabetes with complications, periheral vascular disorders, drug abuse and alcohol abuse were all significantly less likely to be diagnosed among people with RMDs compared to people without. ORs were generally stable over the study period between 2010 and 2019, with the exception of depression which was found to be significantly higher among patients with RMDs between 2010 and 2015 but was significantly (albeit slightly) lower in 2018 and 2019. The odds ratio's (ORs) are presented in Table 1. The ORs for the gender-specific analyses is presented in Figure 3 Supplementary.

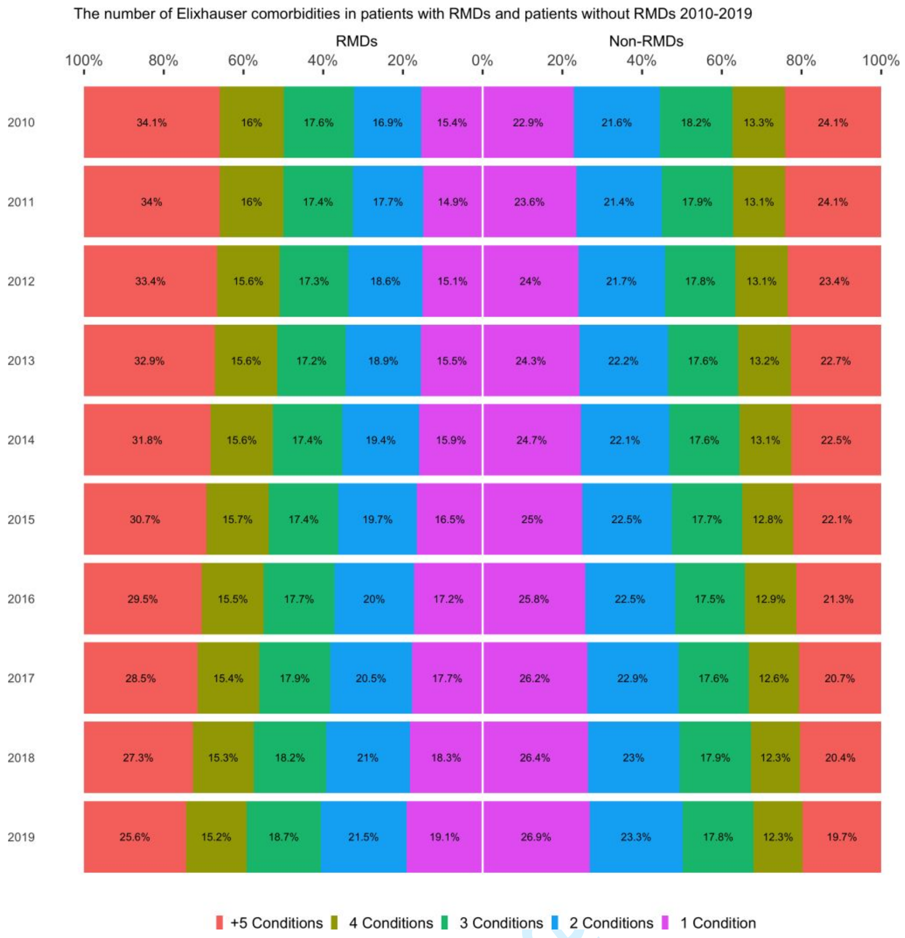

(a) The number of conditions in patients with RMDs and matched comparators without RMDs in 2010-2019.

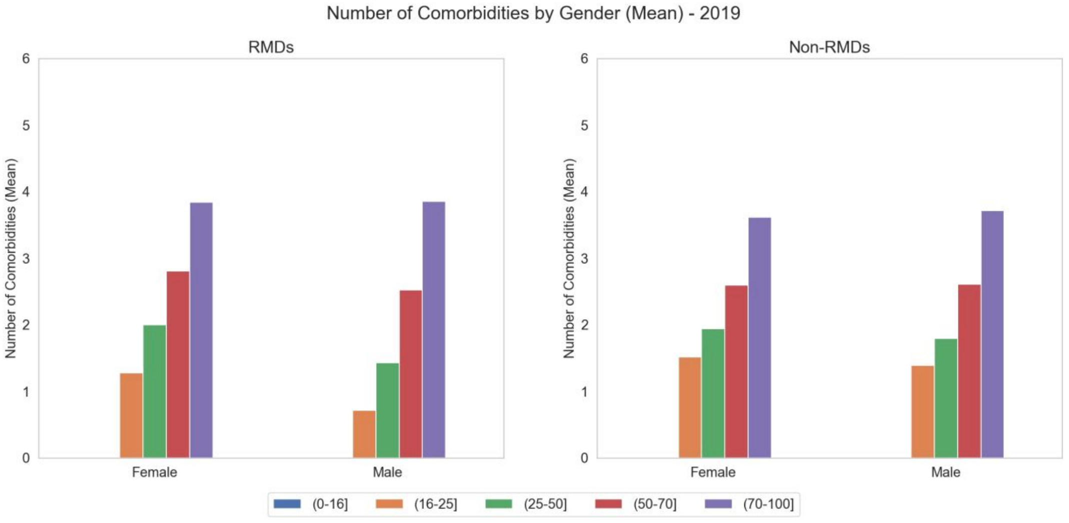

(b) Mean number of comorbidities in patients with RMDs and comparators by age categories and sex 2019

Figure 3. Trends of co-morbidities in patients with RMDs and matched comparators

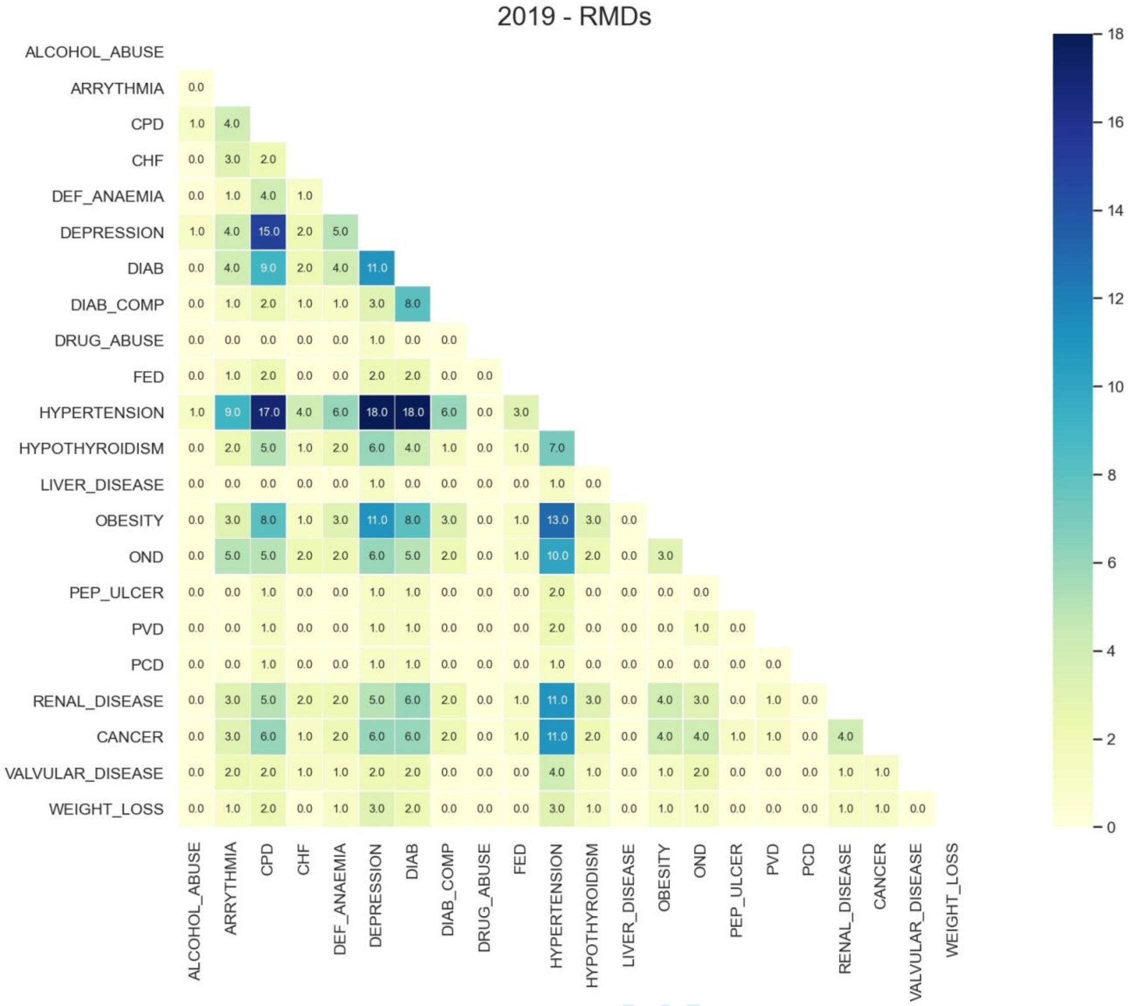

(a) RMDs patients

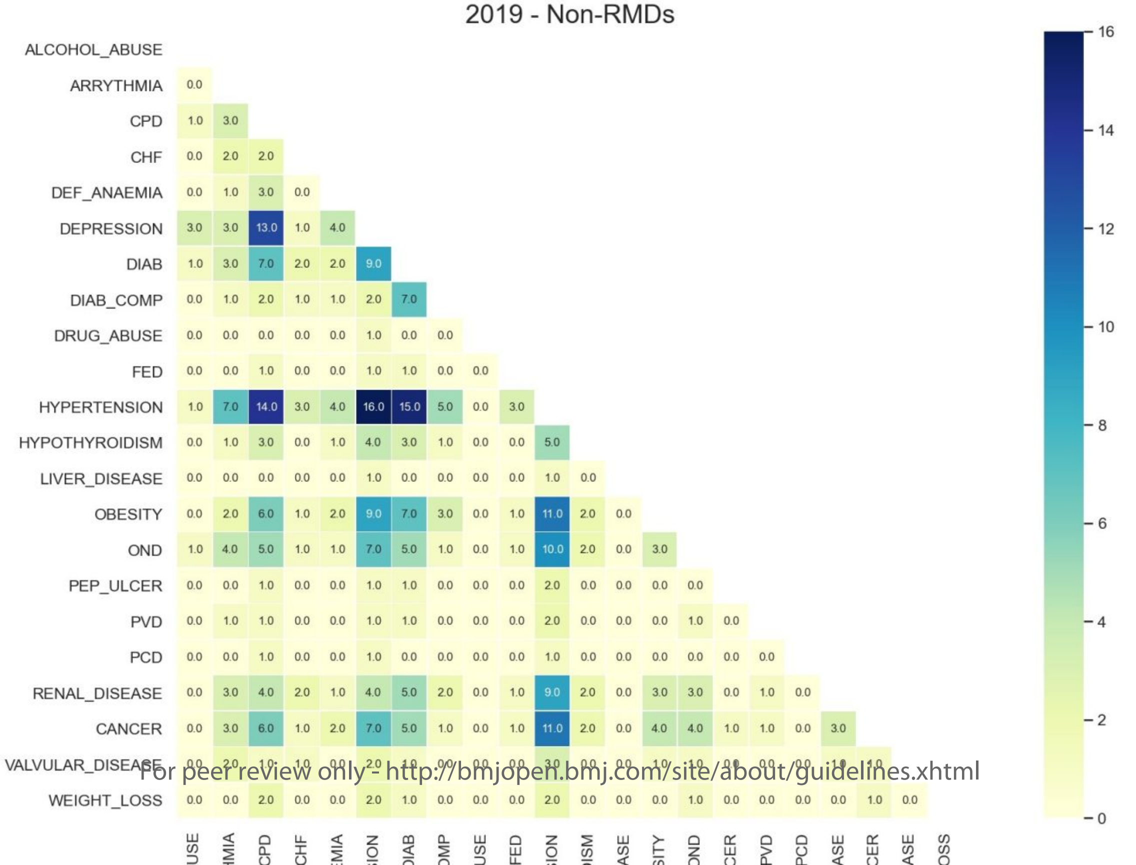

Conditions	2010	2011	2012	2013	2014	2015	2016	2017	2018	2019
ALCOHOL ABUSE	0.73 (0.58-0.93)	0.75 (0.63-0.88)	0.71 (0.62-0.82)	0.71 (0.63-0.80)	0.68 (0.61-0.75)	0.67 (0.61-0.74)	0.67 (0.61-0.73)	0.66 (0.61-0.72)	0.68 (0.62-0.74)	0.64 (0.59-0.69)
ARRYTHMIA	1.06 (0.94-1.20)	1.04 (0.96-1.14)	1.04 (0.97-1.12)	1.09 (1.02-1.16)	1.10 (1.04-1.16)	1.10 (1.05-1.16)	1.15 (1.09-1.21)	1.15 (1.10-1.21)	1.18 (1.13-1.24)	1.13 (1.08-1.19)
CPD	1.09 (1.00-1.18)	1.14 (1.08-1.21)	1.13 (1.08-1.19)	1.13 (1.08-1.18)	1.10 (1.06-1.15)	1.08 (1.04-1.12)	1.07 (1.03-1.10)	1.07 (1.04-1.11)	1.03 (1.00-1.07)	1.02 (0.99-1.05)
CHF	0.99 (0.85-1.16)	1.00 (0.89-1.11)	1.00 (0.91-1.10)	0.99 (0.91-1.07)	1.03 (0.95-1.10)	0.99 (0.92-1.06)	0.99 (0.93-1.06)	0.99 (0.93-1.06)	0.95 (0.89-1.02)	0.91 (0.85-0.97)
DEF_ANAEMIA	1.26 (1.11-1.42)	1.33 (1.22-1.45)	1.36 (1.27-1.46)	1.36 (1.28-1.44)	1.37 (1.29-1.44)	1.39 (1.32-1.46)	1.37 (1.31-1.44)	1.32 (1.26-1.39)	1.32 (1.26-1.38)	1.30 (1.25-1.36)
DEPRESSION	1.17 (1.07-1.27)	1.13 (1.07-1.20)	1.08 (1.03-1.14)	1.07 (1.03-1.12)	1.07 (1.03-1.11)	1.03 (0.99-1.06)	1.01 (0.98-1.05)	1.00 (0.97-1.03)	0.96 (0.94-0.99)	0.95 (0.92-0.98)
DIAB	1.13 (1.02-1.26)	1.17 (1.08-1.26)	1.16 (1.09-1.23)	1.17 (1.11-1.23)	1.13 (1.08-1.19)	1.15 (1.10-1.21)	1.13 (1.09-1.18)	1.13 (1.09-1.18)	1.13 (1.09-1.18)	1.14 (1.10-1.18)
DIAB_COMP	0.89 (0.76-1.05)	0.86 (0.77-0.96)	0.87 (0.79-0.95)	0.92 (0.84-0.99)	0.94 (0.87-1.01)	0.92 (0.86-0.99)	0.92 (0.86-0.98)	0.93 (0.87-0.99)	0.88 (0.83-0.94)	0.90 (0.85-0.95)
DRUG_ABUSE	0.91 (0.66-1.25)	0.68 (0.53-0.86)	0.81 (0.67-0.98)	0.80 (0.68-0.94)	0.75 (0.65-0.87)	0.77 (0.67-0.89)	0.79 (0.69-0.90)	0.79 (0.70-0.89)	0.75 (0.67-0.85)	0.73 (0.65-0.82)
FED	1.26 (1.07-1.47)	1.28 (1.14-1.43)	1.26 (1.14-1.39)	1.27 (1.17-1.38)	1.21 (1.12-1.31)	1.21 (1.13-1.30)	1.19 (1.11-1.27)	1.22 (1.14-1.30)	1.18 (1.10-1.26)	1.16 (1.09-1.24)
HYPERTENSION	1.02 (0.94-1.12)	1.05 (0.99-1.12)	1.06 (1.01-1.12)	1.05 (1.00-1.09)	1.04 (1.00-1.08)	1.01 (0.97-1.04)	1.02 (0.99-1.06)	1.01 (0.98-1.05)	1.03 (1.00-1.06)	1.04 (1.01-1.07)
HYPOTHYROIDI	1.34 (1.19-1.50)	1.19 (1.10-1.29)	1.18 (1.10-1.26)	1.21 (1.14-1.29)	1.18 (1.12-1.24)	1.20 (1.14-1.26)	1.24 (1.19-1.30)	1.25 (1.20-1.31)	1.25 (1.20-1.31)	1.27 (1.22-1.32)
LIVER_DISEASE	0.83 (0.62-1.12)	1.03 (0.84-1.26)	0.97 (0.83-1.14)	1.03 (0.90-1.18)	1.10 (0.98-1.24)	1.11 (1.00-1.24)	1.13 (1.02-1.25)	1.09 (0.99-1.21)	1.01 (0.92-1.12)	1.03 (0.94-1.14)
OBESITY	1.11 (1.00-1.23)	1.08 (1.00-1.16)	1.02 (0.96-1.08)	1.06 (1.01-1.12)	1.08 (1.04-1.13)	1.08 (1.04-1.13)	1.08 (1.04-1.12)	1.08 (1.04-1.12)	1.09 (1.05-1.13)	1.07 (1.03-1.11)
OND	0.91 (0.82-1.01)	0.89 (0.83-0.95)	0.94 (0.89-1.00)	0.91 (0.87-0.96)	0.92 (0.88-0.96)	0.90 (0.86-0.94)	0.91 (0.87-0.95)	0.89 (0.86-0.93)	0.87 (0.84-0.91)	0.84 (0.81-0.88)
PEP_ULCER	1.02 (0.84-1.23)	1.04 (0.91-1.19)	1.06 (0.95-1.19)	1.04 (0.95-1.15)	1.03 (0.94-1.12)	1.05 (0.97-1.14)	1.07 (0.99-1.16)	1.05 (0.97-1.13)	1.00 (0.93-1.07)	0.98 (0.91-1.05)
PVD	0.88 (0.73-1.05)	0.92 (0.82-1.05)	0.93 (0.84-1.03)	0.99 (0.90-1.08)	0.97 (0.89-1.06)	0.98 (0.91-1.06)	0.93 (0.86-1.00)	0.95 (0.88-1.02)	0.89 (0.82-0.95)	0.88 (0.82-0.94)
PCD	1.39 (1.12-1.73)	1.33 (1.15-1.55)	1.30 (1.14-1.47)	1.24 (1.11-1.38)	1.25 (1.13-1.38)	1.25 (1.13-1.37)	1.24 (1.14-1.36)	1.25 (1.15-1.37)	1.11 (1.02-1.21)	1.15 (1.06-1.25)
RENAL failure	1.35 (1.22-1.50)	1.31 (1.22-1.41)	1.28 (1.20-1.36)	1.24 (1.17-1.31)	1.21 (1.15-1.27)	1.22 (1.16-1.28)	1.16 (1.11-1.22)	1.13 (1.08-1.18)	1.14 (1.09-1.19)	1.13 (1.09-1.18)
CANCER	0.92 (0.84-1.02)	0.93 (0.87-0.99)	0.90 (0.85-0.95)	0.90 (0.85-0.94)	0.89 (0.85-0.93)	0.89 (0.85-0.92)	0.87 (0.84-0.90)	0.85 (0.82-0.89)	0.84 (0.81-0.87)	0.81 (0.78-0.84)
VALVULAR _DISEASE	1.16 (1.00-1.35)	1.27 (1.15-1.41)	1.20 (1.10-1.31)	1.26 (1.17-1.36)	1.24 (1.16-1.33)	1.22 (1.15-1.31)	1.17 (1.10-1.25)	1.17 (1.10-1.24)	1.15 (1.08-1.22)	1.13 (1.07-1.20)
WEIGHT_LOSS	1.20 (1.03-1.39)	1.30 (1.17-1.45)	1.26 (1.16-1.38)	1.30 (1.20-1.40)	1.24 (1.16-1.33)	1.22 (1.14-1.30)	1.20 (1.13-1.28)	1.26 (1.19-1.34)	1.20 (1.13-1.27)	1.16 (1.09-1.23)

Table 1. Odds ratios (95% CI) for associations of RMDs with specific co-morbidities in annual cohorts 2010-2019. Alcohol abuse, Blood loss anemia, Cardiac arrhythmia, Chronic pulmonary disease (CPD), Congestive heart failure (CHF), Deficiency anemia, Depression, Diabetes complicated, Diabetes, Drug abuse, FLuid and electrolyte disorder (FED), Hypertension, Hypothyroidism, Liver disease, Other neurological disorders (OND), Paralysis, Peptic ulcer disease excluding bleeding (PEPTIC_ULCER), Periheral vascular disorders (PVD), Psychoses, Pulmonary circulation disorders (PCD), Renal failure, Cancer, Valvular disease, Weight loss

For peer review only - http://bmjopen.bmj.com/site/about/guidelines.xhtml

6 Discussion

To our knowledge, this is the first study to examine and compare the patterns of 28 Elixhauser co-morbidities using different statistical approaches in a large, well-characterized population of people with RMDs and people without RMDs in Wales. The primary care database that we used to estimate the prevalence of multi-morbidity covered over 85.14% of the population in the Wales so the findings should be generalizable in that population. Our findings show that co-morbidities were more prevalent in people with RMDs than comparators throughout the 10-year study period. Overall, co-morbidities were more prevalent in women than in men and the prevalence rates increased with age. Our results demonstrate people with RMDs are approximately 1.5 as likely to have multi-morbidity as the general population throughout the 10-year study period. This is well illustrated by considering the results for prevalence rate and Odds ratio (OR). Overall, prevalence rates increased with age, and co-morbidities were more prevalent in women than in men. Diabetes and hypertension are the epicenter of RMDs disease and potentially part of the trajectories of several other chronic conditions suggesting a need for a more integrative multidisciplinary approach focusing on better management and prevention of these conditions[2].

An Australian systematic review of population-based studies in the elderly[15] has previously reported that over 50% of elderly patients with arthritis also had hypertension, followed by cardiovascular diseases, dyslipidemia, diabetes, and mental health problems, in keeping with our findings. Our study was not restricted to elderly patients but shows that the mean number of co-morbidities is also higher in females at younger ages (25-100 years) whilst in males, the increased mean number of co-morbidities was observed from the age of 50. Consistent with our findings, a recent study[22] found that in Beirut population of 769 people with RMDs , the prevalence of cardiovascular risk factors, hypertension hypercholesterolemia, obesity, diabetes and depression were all increased. Therefore, we have confirmed this finding in a larger, independent cohort and findings appear widely generalizable in European populations. When looking at specific RMDs, patients with rheumatoid arthritis (RA) have been reported to have a significantly increased prevalence of hypothyroidism and chronic pulmonary disease at index date compared to non-RA patients[29]. Patients with RA have also been reported to have a greater prevalence of diabetes mellitus and hypertension, two key risk factors for chronic kidney disease (CKD), possibly arising from the use of corticosteroids to manage RA[30]. Some studies show that weight loss is a strong predictor of death in patients with RA[31–33] so it is important to recognize that weight loss is more highly prevalent across all RMDs from our study. For patients with axial spondyloarthritis (axSpA) identified from health insurance data in Germany, the most prevalent comorbid Elixhuaser conditions were 'hypertension' followed by 'depression' and 'chronic pulmonary' disease[34], illustrating that these co-morbid conditions are common regardless of the specific RMD.

Strengths and limitations

Our study has several strengths, first, to our knowledge, this is the first study to present analysis using different approaches in large population of Wales in people with and without RMDs using routine primary care data so avoiding biased sampling strategies. Furthermore, the study design minimised bias by matching groups on relevant confounders. Second, our large cohort provides sufficient statistical power to calculate overall and age- and sex-specific prevalence rates of individual co-morbidities between 2010 and 2019. Finally, in addition to the recognised and frequently reported co-morbidities such as cardiovascular risk factors, our findings highlight a number of co-morbidities such as deficiency anaemia, fluid and electrolyte disorders that are highly prevalent in people with RMDs but which are under-reported in literature.

There are various limitations to the study that should be considered when interpreting the results. For example, because this study depends on diagnoses reported in electronic health records, it is possible that patients with undiagnosed conditions, such as those who did not visit a general practitioner, were missed. Due to statistical disclosure control, seven Elixhauser conditions were excluded from all the analysis because of the low prevalence rates of these conditions, but a comprehensive assessment of common conditions was possible. A further limitation is that we have analysed only pairs of diseases, but higher-order combinations of diseases probably interact in more complicated ways. In terms of the application of these findings, it should be noted that the prevalence measure was based on Elixhauser conditions; there is no established gold standard list of diagnoses used to define multi-morbidity and definitions of multi-morbidity vary in the medical literature[35], making direct comparisons across studies and different populations more challenging. However, separately both the Charlson and Elixhauser indices are often used[36] and the Cambridge multi-morbidity score has been published recently[35] and there is some overlap between these indices in defining co-morbidity.

7 Conclusion

In conclusion, we provide the first detailed 10-year epidemiological analysis of multi-morbidity burden in people with RMDs in comparison with individuals without RMDs using Welsh primary care data. We found a substantially higher prevalence and burden of multi-morbidity in patients with RMDs relative to those without, suggesting that RMDs patients represent a high-risk group for multi-morbidity. A greater understanding of multi-morbidity could aid clinicians in developing and

For peer review only - http://bmjopen.bmj.com/site/about/guidelines.xhtml

delivering comprehensive treatment pathways and to action primary and secondary prevention of co-morbidities in people with RMDs.

This higher mean number of co-morbidities is seen from the age of 25 in women and 50 in men. Further research is required to determine whether targeted interventions could reduce overall disease burden from multi-morbidity in that group. It is feasible that the prevalence of multi-morbidity might vary for subtypes of RMDs included in each definition but it remains to be determined whether RMDs in general, or only particular subgroups of RMDs, are associated with increased prevalence of multi-morbidity.

8 Abbreviations

The Table 2 describes the significance of various abbreviations and acronyms used throughout of this paper.

Full description	abbreviations
Rheumatic and musculoskeletal diseases	RMDs
General Practices	GPs
Secure Anonymised Information Linkage	SAIL
Odds ratios	ORs
Trusted Research Environment	TRE
International Classification of Diseases 10th revision	ICD-10 codes
OPCS Classification of Interventions and Procedures version 4	(OPCS-4)
Multiple longterm conditions	MLTCs
Welsh Multi-morbidity e-Cohort	WMC
Alcohol abuse	ALCOHOL_ABUSE
Blood loss anemia	BL_ANAEMIA
Cardiac arrhythmia	CARD_ARRYTHMIA
Chronic pulmonary disease	CPD
Coagulopathy	COAGUL
Congestive heart failure	CHF
Deficiency anemia	DEF_ANAEMIA
Depression	DEPRESSION
Diabetes complicated	DIAB_COMB
Diabetes, uncomplicated	DIAB
Drug abuse	DRUG_ABUSE
FLuid and electrolyte disorders	FED
Hypertension	HYPERTENSION
Hypothyroidism	HYPOTHYROIDISM
Liver disease	LIVER_DIS
Lymphoma	LYMPHOMA
Metastatic cancer	MET_CANCER
Obesity	OBESITY
Other Neurological disorders	OND
Paralysis	PARALYSIS
Peptic ulcer disease excluding bleeding	PEPTIC_ULCER
Periheral vascular disorders	PVD
Psychoses	PSYCHOSES
Pulmonary circulation disorders	PCD
Renal failure	RENAL
Rheumatoid arthritis/collagen vascular diseases	RA
Solid tumor without metastasis	CANCER
Valvular disease	VALVULAR
Weight loss	WEIGHT_LOSS

Table 2. Abbreviations list

For peer review only

References

1. Wu, S.-Y. & Green, A. Projection of chronic illness prevalence and cost inflation. *Santa Monica, CA: RAND Heal.* **18** (2000).
2. Buttorff, C., Ruder, T. & Bauman, M. *Multiple chronic conditions in the United States*, vol. 10 (Rand Santa Monica, CA, 2017).
3. Whitty, C. J. *et al.* Rising to the challenge of multimorbidity (2020).
4. Barnett, K. *et al.* Epidemiology of multimorbidity and implications for health care, research, and medical education: a cross-sectional study. *The Lancet* **380**, 37–43 (2012).
5. Haregu, T. N., Oldenburg, B. F., Setswe, G. & Elliott, J. Perspectives, constructs and methods in the measurement of multimorbidity and comorbidity: a critical review. *The Internet J. Epidemiol.* **10**, 1–11 (2012).
6. Zghebi, S. S., Steinke, D. T., Rutter, M. K. & Ashcroft, D. M. Eleven-year multimorbidity burden among 637 255 people with and without type 2 diabetes: a population-based study using primary care and linked hospitalisation data. *BMJ open* **10**, e033866 (2020).
7. Sherrer, Y. S., Bloch, D. A., Mitchell, D. M., Young, D. Y. & Fries, J. F. The development of disability in rheumatoid arthritis. *Arthritis & Rheum. Off. J. Am. Coll. Rheumatol.* **29**, 494–500 (1986).
8. Sparks, J. A. *et al.* Rheumatoid arthritis and mortality among women during 36 years of prospective follow-up: results from the nurses' health study. *Arthritis care & research* **68**, 753–762 (2016).
9. Woolf, A. D. & Pfleger, B. Burden of major musculoskeletal conditions. *Bull. world health organization* **81**, 646–656 (2003).
10. Ellis, B. M. & Conaghan, P. G. Reducing arthritis pain through physical activity: a new public health, tiered approach (2017).
11. Melton III, L. & EA, C. Cooper c. *Lane AW. Riggs BL. Perspective. How many women have osteoporosis* 5–1 (1992).
12. Kanis, J. *et al.* Long-term risk of osteoporotic fracture in malmö. *Osteoporos. international* **11**, 669–674 (2000).
13. Melton III, L. J., Atkinson, E. J., O'Connor, M. K., O'Fallon, W. M. & Riggs, B. L. Bone density and fracture risk in men. *J. Bone Miner. Res.* **13**, 1915–1923 (1998).
14. Taylor, A. W. *et al.* Multimorbidity-not just an older person's issue. results from an australian biomedical study. *BMC public health* **10**, 1–10 (2010).
15. Caughey, G. E., Vitry, A. I., Gilbert, A. L. & Roughead, E. E. Prevalence of comorbidity of chronic diseases in australia. *BMC public health* **8**, 1–13 (2008).
16. Vogeli, C. *et al.* Multiple chronic conditions: prevalence, health consequences, and implications for quality, care management, and costs. *J. general internal medicine* **22**, 391–395 (2007).
17. Prados-Torres, A., Calderón-Larrañaga, A., Hancco-Saavedra, J., Poblador-Plou, B. & van den Akker, M. Multimorbidity patterns: a systematic review. *J. clinical epidemiology* **67**, 254–266 (2014).
18. Diederichs, C., Berger, K. & Bartels, D. B. The measurement of multiple chronic diseases—a systematic review on existing multimorbidity indices. *Journals Gerontol. Ser. A: Biomed. Sci. Med. Sci.* **66**, 301–311 (2011).
19. Violan, C. *et al.* Prevalence, determinants and patterns of multimorbidity in primary care: a systematic review of observational studies. *PloS one* **9**, e102149 (2014).
20. Lowe, D. B., Taylor, M. J. & Hill, S. J. Cross-sectional examination of musculoskeletal conditions and multimorbidity: influence of different thresholds and definitions on prevalence and association estimates. *BMC research notes* **10**, 1–13 (2017).
21. Salive, M. E. Multimorbidity in older adults. *Epidemiol. reviews* **35**, 75–83 (2013).
22. Ziade, N. *et al.* Prevalence and pattern of comorbidities in chronic rheumatic and musculoskeletal diseases: The comord study. *Sci. reports* **10**, 1–10 (2020).
23. England, B. R. *et al.* Burden and trajectory of multimorbidity in rheumatoid arthritis: a matched cohort study from 2006 to 2015. *Annals rheumatic diseases* **80**, 286–292 (2021).
24. Lyons, R. A. *et al.* The sail databank: linking multiple health and social care datasets. *BMC medical informatics decision making* **9**, 3 (2009).

For peer review only

25. Ford, D. V. *et al.* The sail databank: building a national architecture for e-health research and evaluation. *BMC health services research* **9**, 157 (2009).

26. Metcalfe, D. *et al.* Coding algorithms for defining charlson and elixhauser co-morbidities in read-coded databases. *BMC medical research methodology* **19**, 1–9 (2019).

27. Lyons, J. *et al.* Protocol for the development of the wales multimorbidity e-cohort (wmc): data sources and methods to construct a population-based research platform to investigate multimorbidity. *BMJ open* **11**, e047101 (2021).

28. Von Elm, E. *et al.* The strengthening the reporting of observational studies in epidemiology (strobe) statement: guidelines for reporting observational studies. *The Lancet* **370**, 1453–1457 (2007).

29. Gunderson, T. M., Myasoedova, E., Davis, J. M. & Crowson, C. S. Multimorbidity burden in rheumatoid arthritis: a population-based cohort study. *The J. Rheumatol.* (2021).

30. Adcock, I. M. & Mumby, S. Glucocorticoids. *Pharmacol. Ther. Asthma COPD* 171–196 (2016).

31. Baker, J. F. *et al.* Weight loss, the obesity paradox, and the risk of death in rheumatoid arthritis. *Arthritis & Rheumatol.* **67**, 1711–1717 (2015).

32. England, B. R. *et al.* Body mass index, weight loss, and cause-specific mortality in rheumatoid arthritis. *Arthritis care & research* **70**, 11–18 (2018).

33. Willadsen, T. G. *et al.* The role of diseases, risk factors and symptoms in the definition of multimorbidity–a systematic review. *Scand. journal primary health care* **34**, 112–121 (2016).

34. Redeker, I. *et al.* The prevalence and impact of comorbidities on patients with axial spondyloarthritis: results from a nationwide population-based study. *Arthritis research & therapy* **22**, 1–11 (2020).

35. Payne, R. A. *et al.* Development and validation of the cambridge multimorbidity score. *Cmaj* **192**, E107–E114 (2020).

36. Johnston, M. C., Crilly, M., Black, C., Prescott, G. J. & Mercer, S. W. Defining and measuring multimorbidity: a systematic review of systematic reviews. *Eur. J. Public Heal.* **29**, 182–189 (2019).

Ethics approval and consent to participate

This study makes use of anonymised data held in the Secure Anonymised Information Linkage (SAIL) Databank. This work uses data provided by patients and collected by the NHS as part of their care and support. The data used in this study are available in the SAIL Databank at Swansea University, Swansea, UK, but as restrictions apply they are not publicly available. All proposals to use SAIL data are subject to review by an independent Information Governance Review Panel (IGRP). Before any data can be accessed, approval must be given by the IGRP. The IGRP gives careful consideration to each project to ensure proper and appropriate use of SAIL data. When access has been granted, it is gained through a privacy protecting safe haven and remote access system referred to as the SAIL Gateway. SAIL has established an application process to be followed by anyone who would like to access data via SAIL at https://www.saildatabank.com/application-process

Author's contributions

Farideh Jalali-najafabadi: Methodology, Software, Formal analysis, Writing original draft. Rowena Bailey: Formal analysis, Writing – review & editing. Jane Lyons: Data Curation, Writing – review & editing. Alan Watkins: Writing – review & editing. Ashley Akbari: Writing – review & editing. Thamer Ba Dhafari: Writing – review & editing. Glen P. Martin: Writing – review & editing. John Bowes: Supervision, Writing – review & editing. Anne Barton: Supervision, Writing – review & editing. Ronan A. Lyons: Project administration, Writing – review & editing. Niels Peek: Project administration, Supervision, Writing – review & editing.

Acknowledgements

We would also like to acknowledge all data providers who make anonymised data available for research. All research conducted has been completed under the permission and approval of the SAIL independent Information Governance Review Panel (IGRP) project number 0911. This work uses data provided by patients and collected by the NHS as part of their care and support. We would like to acknowledge all data providers who make anonymised data available for research. We also acknowledge help and advice from Dr.Salwa S Zghebi from school of health science Manchester University, UK.

For peer review only

Competing interests

The creation of the Wales Multi-morbidity Cohort was co-funded by MRC and NIHR through grant number MR/S027750/1. Anne Barton has received grant funding and/or consultancy/speaker fees from Bristol Myers Squibb, Pfizer, Galapagos, Scipher Medicine and Roche-Chugai but not in relation to the current work.

Availability of data and materials

The data used in this study are available in the SAIL Databank at Swansea University, Swansea, UK, but as restrictions apply they are not publicly available. All proposals to use SAIL data are subject to review by an independent Information Governance Review Panel (IGRP). Before any data can be accessed, approval must be given by the IGRP. The IGRP gives careful consideration to each project to ensure proper and appropriate use of SAIL data. When access has been granted, it is gained through a privacy protecting safe haven and remote access system referred to as the SAIL Gateway. SAIL has established an application process to be followed by anyone who would like to access data via SAIL at https://www.saildatabank.com/application-process

Funding

The datasets used in this study were supported by grants co-funded by Medical Research Council (MRC) and NIHR (grant number:MR/S027750/1); and supported by Health Data Research UK (grant number: HDR-9006), which receives its funding from the UK Medical Research Council, Engineering and Physical Sciences Research Council, Economic and Social Research Council, Department of Health and Social Care (England), Chief Scientist Office of the Scottish Government Health and Social Care Directorates, Health and Social Care Research and Development Division (Welsh Government), Public Health Agency (Northern Ireland), British Heart Foundation (BHF) and the Wellcome Trust. This work was supported by the ADR Wales programme of work. The ADR Wales programme of work is aligned to the priority themes as identified in the Welsh Government's national strategy: Prosperity for All. ADR Wales brings together data science experts at Swansea University Medical School, staff from the Wales Institute of Social and Economic Research, Data and Methods (WISERD) at Cardiff University and specialist teams within the Welsh Government to develop new evidence which supports Prosperity for All by using the SAIL Databank at Swansea University, to link and analyse anonymised data. ADR Wales is part of the Economic and Social Research Council (part of UK Research and Innovation) funded ADR UK (grant ES/S007393/1). This research was partially funded by the National Institute for Health Research (NIHR)'s Manchester Biomedical Research Centre. The views expressed are those of the author(s) and not necessarily those of the NHS, the National Institute for Health research or the Department of Health and Social Care. The work is supported by the Centre for Genetics and Genomics Versus Arthritis (UK grant number 21754). AB is an NIHR Senior Investigator. Farideh Jalali-najafabadi's research is supported by an MRC/University of Manchester Skills Development Fellowship (grant number MR/R016615).

Availability of data and materials

The data used in this study are available in the SAIL Databank at Swansea University, Swansea, UK, but as restrictions apply they are not publicly available. All proposals to use SAIL data are subject to review by an independent Information Governance Review Panel (IGRP). Before any data can be accessed, approval must be given by the IGRP. The IGRP gives careful consideration to each project to ensure proper and appropriate use of SAIL data. When access has been granted, it is gained through a privacy protecting safe haven and remote access system referred to as the SAIL Gateway. SAIL has established an application process to be ned by anyone who would like to access data via SAIL at https://www.saildatabank.com/application-process.

Supplementary

10 years multimorbidity patterns among people with and without Rheumatic and Musculoskeletal diseases: A population-based study using Wales primary care data

For peer review only

GP Elixhauser condition description	Variable description
Alcohol abuse	ALCOHOL_ABUSE
Blood loss anemia	BL_ANAEMIA
Cardiac arrhythmia	CARD_ARRYTHMIA
Chronic pulmonary disease	CPD
Coagulopathy	COAGUL
Congestive heart failure	CHF
Deficiency anemia	DEF_ANAEMIA
Depression	DEPRESSION
Diabetes complicated	DIAB_COMB
Diabetes, uncomplicated	DIAB
Drug abuse	DRUG_ABUSE
FLuid and electrolyte disorders	FED
Hypertension	HYPERTENSION
Hypothyroidism	HYPOTHYROIDISM
Liver disease	LIVER_DIS
Lymphoma	LYMPHOMA
Metastatic cancer	MET_CANCER
Obesity	OBESITY
Other Neurological disorders	OND
Paralysis	PARALYSIS
Peptic ulcer disease excluding bleeding	PEPTIC_ULCER
Periheral vascular disorders	PVD
Psychoses	PSYCHOSES
Pulmonary circulation disorders	PCD
Renal failure	RENAL
Rheumatoid arthritis/collagen vascular diseases	RA
Solid tumor without metastasis	CANCER
Valvular disease	VALVULAR
Weight loss	WEIGHT_LOSS

Table 1. Elixhauser Comorbidities.

For peer review only

Conditions	2010 RMD	2010 Comp	2011) RMD	2011) Comp	(2012) RMD	(2012) Comp	2013 RMD	2013 Comp	2014 RMD	2014 Comp	2015 RMD	2015 Comp	2016 RMD	2016 Comp	2017 RMD	2017 Comp	2018 RMD	2018 Comp	2019 RMD	2019 Comp
ALCOHOL_ABUSE	0.2 (0.02 0.32)	0.1 (0.05- 0.16)	0.3 (0.17 0.45)	0.2 (0.19- 0.31)	0.4 (0.23 0.49)	0.4 (0.30- 0.41)	0.4 (0.27 0.49)	0.4 (0.38- 0.49)	0.4 (0.30 0.51)	0.6 (0.54- 0.65)	0.5 (0.40 0.61)	0.8 (0.72- 0.84)	0.6 (0.53 0.76)	0.8 (0.76- 0.88)	0.8 (0.66 0.89)	1.0 (0.94- 1.06)	0.9 (0.74 0.99)	1.1 (1.02- 1.14)	1.0 (0.86 1.11)	1.3 (1.21- 1.33)
ARRYTHMIA	1.0 (0.64 1.37)	0.6 (0.51- 0.77)	2.1 (1.78 2.52)	1.4 (1.23- 1.50)	3.0 (2.64 3.36)	1.9 (1.77- 2.03)	4.0 (3.64 4.35)	2.5 (2.33- 2.59)	4.6 (4.30 4.98)	3.0 (2.90- 3.15)	5.4 (5.01 5.69)	3.7 (3.58- 3.84)	6.1 (5.80 6.48)	4.3 (4.16- 4.42)	7.0 (6.65 7.34)	5.0 (4.84- 5.11)	7.8 (7.45 8.15)	5.4 (5.30- 5.57)	8.4 (8.02 8.71)	6.2 (6.11- 6.38)
CPD	1.0 (0.67 1.41)	0.6 (0.49- 0.75)	2.2 (1.79 2.54)	1.4 (1.25- 1.52)	3.3 (2.89 3.64)	2.0 (1.88- 2.14)	4.1 (3.78 4.51)	2.7 (2.61- 2.88)	4.8 (4.47 5.17)	3.5 (3.38- 3.65)	5.9 (5.55 6.26)	4.3 (4.15- 4.42)	6.7 (6.38 7.10)	4.9 (4.77- 5.04)	7.6 (7.24 7.96)	5.6 (5.51- 5.79)	8.4 (7.99 8.72)	6.3 (6.19- 6.48)	9.1 (8.74 9.46)	6.9 (6.80- 7.09)
CHF	0.5 (0.23 0.74)	0.3 (0.25- 0.44)	1.2 (0.89 1.43)	0.7 (0.60- 0.79)	1.9 (1.57 2.15)	1.0 (0.87- 1.06)	2.2 (1.95 2.48)	1.3 (1.26- 1.44)	2.7 (2.39 2.91)	1.6 (1.48- 1.66)	3.0 (2.79 3.31)	2.0 (1.86- 2.05)	3.5 (3.21 3.73)	2.3 (2.16- 2.35)	3.9 (3.65 4.17)	2.6 (2.53- 2.72)	4.3 (3.99 4.52)	3.0 (2.87- 3.07)	4.5 (4.28 4.81)	3.2 (3.15- 3.35)
DEF_ANAEMIA	1.1 (0.75 1.53)	0.5 (0.37- 0.60)	2.1 (1.71 2.44)	0.8 (0.71- 0.91)	2.4 (2.09 2.74)	1.2 (1.08- 1.29)	2.9 (2.61 3.22)	1.6 (1.54- 1.75)	3.6 (3.27 3.87)	1.9 (1.83- 2.03)	4.3 (4.00 4.61)	2.3 (2.20- 2.41)	4.8 (4.50 5.10)	2.7 (2.64- 2.84)	5.5 (5.23 5.85)	3.2 (3.12- 3.34)	6.1 (5.79 6.41)	3.6 (3.52- 3.74)	6.8 (6.44 7.07)	4.0 (3.86- 4.08)
DEPRESSION	1.6 (1.16 2.09)	1.2 (1.00- 1.35)	2.9 (2.52 3.38)	2.1 (1.91- 2.24)	3.9 (3.47 4.29)	3.3 (3.11- 3.45)	5.2 (4.79 5.60)	4.3 (4.17- 4.50)	5.9 (5.55 6.32)	5.3 (5.12- 5.45)	7.0 (6.63 7.40)	6.6 (6.41- 6.75)	8.0 (7.59 8.36)	7.4 (7.26- 7.59)	9.1 (8.72 9.50)	8.5 (8.31- 8.65)	10.1 (9.74 10.53	9.4 (9.25- 9.59)	11.2 (10.7 11.58	10.5 (10.31- 10.65)
DIAB	2.2 (1.68 2.75)	1.2 (1.05- 1.40)	4.3 (3.74 4.78)	2.6 (2.39- 2.76)	5.8 (5.35 6.34)	3.6 (3.43- 3.78)	7.9 (7.42 8.41)	5.0 (4.79- 5.14)	9.2 (8.75 9.69)	6.3 (6.13- 6.49)	10.5 (10.0 10.97	7.1 (6.95- 7.30)	11.4 (10.9 11.89	8.0 (7.83- 8.17)	12.3 (11.8 12.77	8.7 (8.54- 8.89)	13.5 (13.0 13.94	9.7 (9.51- 9.85)	14.4 (13.9 14.80	10.4 (10.21- 10.56)
DIAB_COMP	0.6 (0.31 0.87)	0.4 (0.29- 0.50)	1.0 (0.77 1.28)	0.8 (0.68- 0.88)	1.5 (1.27 1.79)	1.2 (1.13- 1.34)	2.6 (2.35 2.94)	2.0 (1.93- 2.16)	3.4 (3.09 3.68)	2.7 (2.62- 2.86)	4.0 (3.71 4.30)	3.0 (2.90- 3.13)	4.4 (4.11 4.70)	3.3 (3.22- 3.45)	4.7 (4.44 5.02)	3.6 (3.48- 3.71)	5.1 (4.76 5.34)	4.0 (3.86- 4.09)	5.5 (5.18 5.76)	4.3 (4.22- 4.45)
DRUG_ABUSE	0.2 (0.02 0.32)	0.1 (0.02- 0.09)	0.2 (0.11 0.36)	0.1 (0.10- 0.19)	0.3 (0.21 0.45)	0.2 (0.16- 0.24)	0.4 (0.25 0.46)	0.3 (0.23- 0.31)	0.4 (0.28 0.48)	0.4 (0.33- 0.42)	0.4 (0.31 0.50)	0.4 (0.34- 0.43)	0.4 (0.35 0.54)	0.5 (0.41- 0.50)	0.5 (0.41 0.60)	0.6 (0.51- 0.60)	0.5 (0.45 0.64)	0.6 (0.57- 0.66)	0.6 (0.53 0.73)	0.7 (0.63- 0.72)

Table 2 – continued from previous page

FED	0.7 (0.39 0.99)	0.3 (0.21-0.39)	1.2 (0.89 1.43)	0.6 (0.52-0.70)	1.5 (1.23 1.74)	0.6 (0.57-0.72)	1.7 (1.45 1.92)	1.0 (0.90-1.06)	2.0 (1.74 2.20)	1.1 (1.06-1.21)	2.3 (2.06 2.52)	1.5 (1.45-1.62)	2.6 (2.33 2.78)	1.7 (1.59-1.75)	2.8 (2.62 3.07)	1.9 (1.78-1.94)	3.1 (2.91 3.37)	2.1 (2.03-2.20)	3.4 (3.19 3.65)	2.4 (2.29-2.46)
HYPERTENSION	2.4 (1.80 2.90)	1.2 (1.03-1.38)	3.3 (2.80 3.71)	2.6 (2.45-2.81)	4.7 (4.29 5.19)	3.6 (3.44-3.79)	6.1 (5.66 6.53)	5.1 (4.89-5.25)	7.7 (7.28 8.15)	6.2 (6.06-6.41)	9.0 (8.58 9.45)	7.3 (7.13-7.48)	10.4 (9.93 10.80)	8.6 (8.38-8.73)	11.9 (11.4 12.31)	10.0 (9.85-10.21)	13.3 (12.8 13.70)	11.2 (11.06-11.43)	15.0 (14.5 15.40)	12.6 (12.39-12.77)
HYPOTHYROIDISM	1.0 (0.64 1.37)	0.3 (0.24-0.43)	1.4 (1.11 1.72)	0.8 (0.70-0.90)	1.9 (1.57 2.15)	1.2 (1.07-1.27)	2.2 (1.98 2.52)	1.4 (1.32-1.51)	2.7 (2.39 2.91)	1.8 (1.72-1.91)	3.1 (2.85 3.37)	2.2 (2.06-2.26)	3.4 (3.11 3.62)	2.4 (2.29-2.48)	3.8 (3.52 4.04)	2.7 (2.62-2.81)	4.1 (3.86 4.38)	2.9 (2.81-3.01)	4.3 (4.06 4.58)	3.1 (3.02-3.22)
LIVER_DISEASE	0.2 (0.04 0.37)	0.1 (0.02-0.09)	0.3 (0.15 0.43)	0.1 (0.10-0.18)	0.4 (0.25 0.51)	0.2 (0.16-0.24)	0.4 (0.32 0.56)	0.3 (0.26-0.35)	0.6 (0.45 0.70)	0.3 (0.26-0.34)	0.7 (0.54 0.78)	0.4 (0.35-0.43)	0.7 (0.59 0.83)	0.4 (0.39-0.48)	0.8 (0.70 0.94)	0.5 (0.49-0.57)	0.9 (0.78 1.02)	0.6 (0.59-0.69)	1.0 (0.91 1.17)	0.7 (0.68-0.78)
OBESITY	0.9 (0.53 1.20)	0.6 (0.46-0.71)	1.4 (1.08 1.68)	1.0 (0.89-1.12)	2.0 (1.74 2.33)	1.7 (1.54-1.79)	2.6 (2.32 2.90)	2.0 (1.91-2.14)	3.4 (3.07 3.66)	2.5 (2.36-2.59)	3.9 (3.59 4.17)	3.0 (2.87-3.10)	4.6 (4.30 4.90)	3.6 (3.49-3.72)	5.1 (4.79 5.39)	4.1 (4.00-4.25)	5.9 (5.57 6.18)	4.9 (4.80-5.05)	7.0 (6.69 7.33)	5.9 (5.74-6.00)
OND	0.9 (0.56 1.24)	0.7 (0.56-0.83)	2.2 (1.79 2.54)	1.6 (1.42-1.70)	2.8 (2.49 3.19)	2.3 (2.12-2.40)	3.8 (3.44 4.14)	3.0 (2.84-3.11)	4.7 (4.36 5.06)	3.9 (3.79-4.07)	5.9 (5.55 6.26)	4.8 (4.63-4.91)	6.7 (6.39 7.11)	5.6 (5.50-5.79)	7.8 (7.40 8.12)	6.4 (6.29-6.58)	8.9 (8.53 9.27)	7.4 (7.26-7.56)	10.2 (9.86 10.62)	8.6 (8.41-8.73)
PEP_ULCER	0.2 (0.04 0.37)	0.1 (0.06-0.16)	0.3 (0.15 0.43)	0.2 (0.11-0.21)	0.4 (0.24 0.50)	0.2 (0.17-0.26)	0.5 (0.40 0.66)	0.3 (0.23-0.32)	0.6 (0.52 0.78)	0.4 (0.34-0.43)	0.7 (0.57 0.82)	0.4 (0.37-0.45)	0.8 (0.65 0.90)	0.5 (0.46-0.55)	0.8 (0.71 0.96)	0.5 (0.47-0.55)	0.9 (0.80 1.05)	0.6 (0.58-0.67)	1.0 (0.85 1.09)	0.7 (0.63-0.72)
PVD	0.2 (0.06 0.42)	0.2 (0.11-0.25)	0.4 (0.25 0.57)	0.4 (0.28-0.42)	0.8 (0.63 1.02)	0.6 (0.50-0.65)	1.3 (1.07 1.48)	0.7 (0.67-0.81)	1.5 (1.30 1.69)	0.9 (0.87-1.01)	1.8 (1.55 1.95)	1.1 (1.05-1.19)	2.0 (1.78 2.17)	1.4 (1.33-1.48)	2.2 (2.04 2.44)	1.5 (1.46-1.61)	2.4 (2.24 2.64)	1.7 (1.64-1.79)	2.5 (2.31 2.71)	1.9 (1.79-1.94)
PCD	0.2 (0.06 0.42)	0.1 (0.04-0.14)	0.4 (0.26 0.59)	0.2 (0.17-0.28)	0.7 (0.48 0.82)	0.3 (0.28-0.39)	0.7 (0.56 0.87)	0.4 (0.35-0.46)	1.0 (0.79 1.11)	0.5 (0.47-0.58)	1.0 (0.88 1.19)	0.7 (0.62-0.73)	1.2 (1.07 1.38)	0.8 (0.70-0.81)	1.4 (1.21 1.52)	0.9 (0.82-0.94)	1.5 (1.37 1.69)	1.1 (1.07-1.19)	1.7 (1.58 1.91)	1.2 (1.10-1.22)
RENAL_DISEASE	1.9 (1.40 2.40)	1.0 (0.84-1.17)	3.3 (2.87 3.78)	1.9 (1.75-2.06)	4.7 (4.29 5.19)	2.8 (2.65-2.97)	6.2 (5.72 6.60)	3.7 (3.53-3.84)	6.8 (6.42 7.24)	4.2 (4.05-4.34)	7.1 (6.75 7.53)	4.5 (4.35-4.63)	7.3 (6.98 7.72)	4.6 (4.51-4.77)	7.3 (6.98 7.68)	4.9 (4.79-5.05)	7.7 (7.36 8.06)	5.3 (5.13-5.39)	7.8 (7.44 8.12)	5.5 (5.39-5.64)

Table 2 – continued from previous page

CANCER	1.3 (0.87 1.69)	1.1 (0.94- 1.28)	2.4 (2.00 2.78)	2.2 (2.00- 2.33)	3.4 (3.04 3.81)	3.2 (3.00- 3.33)	4.4 (4.05 4.80)	4.2 (4.02- 4.35)	5.6 (5.27 6.03)	5.3 (5.13- 5.46)	6.6 (6.25 7.00)	6.4 (6.27- 6.60)	7.6 (7.17 7.93)	7.6 (7.46- 7.80)	8.6 (8.19 8.95)	8.6 (8.43- 8.77)	9.7 (9.31 10.08	9.5 (9.30- 9.64)	10.5 (10.1 10.90	10.6 (10.43- 10.78)
VALVULAR_DISEASE	0.4 (0.21 0.69)	0.2 (0.14- 0.30)	0.8 (0.59 1.05)	0.5 (0.42- 0.58)	1.3 (1.07 1.55)	0.7 (0.64- 0.80)	1.7 (1.44 1.91)	1.0 (0.89- 1.05)	2.1 (1.91 2.38)	1.2 (1.10- 1.26)	2.5 (2.22 2.68)	1.5 (1.39- 1.55)	2.7 (2.50 2.96)	1.5 (1.39- 1.55)	3.3 (3.05 3.53)	1.9 (1.85- 2.02)	3.7 (3.46 3.95)	2.3 (2.22- 2.40)	4.2 (3.92 4.42)	2.5 (2.46- 2.64)
WEIGHT_LOSS	0.7(0	0.3 (0.22- 0.40)	4)1.1(0	0.5 (0.46- 0.63)	1.8 6)(1.49 2.05)	0.9 (0.79- 0.96)	2.1 (1.88 2.41)	1.1 (1.01- 1.18)	2.4 (2.15 2.65)	1.6 (1.50- 1.68)	2.7 (2.46 2.95)	1.9 (1.76- 1.94)	3.0 (2.79 3.28)	2.1 (2.02- 2.20)	3.4 (3.20 3.69)	2.4 (2.26- 2.45)	3.8 (3.59 4.09)	2.7 (2.65- 2.84)	4.2 (3.92 4.42)	3.1 (2.98- 3.17)

Table 2. Annual prevalence rates for comorbidities in RMDs cases and matched comparators 2010-2019

For peer review only

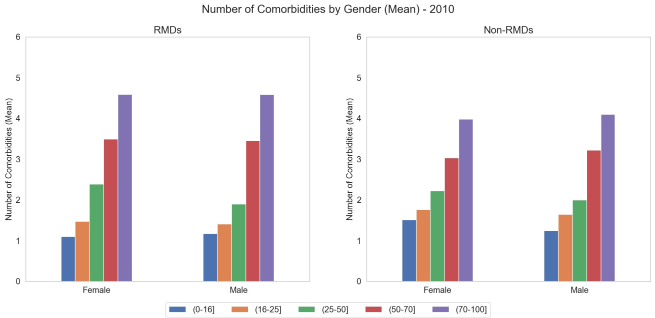

(a) Mean number of comorbidities in patients with RMDs and comparators by age categories and sex 2010

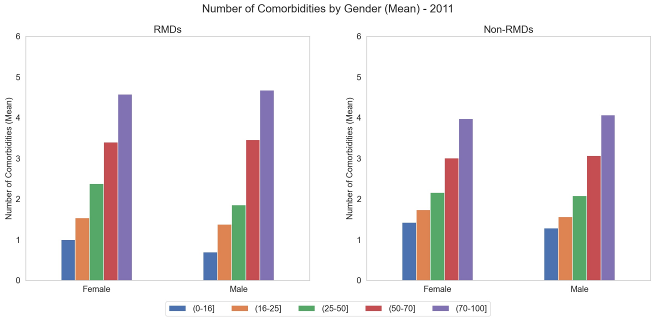

(b) Mean number of comorbidities in patients with RMDs and comparators by age categories and sex 2011

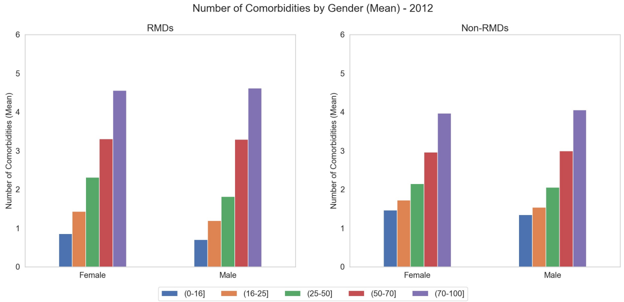

(c) Mean number of comorbidities in patients with RMDs and comparators by age categories and sex 2012

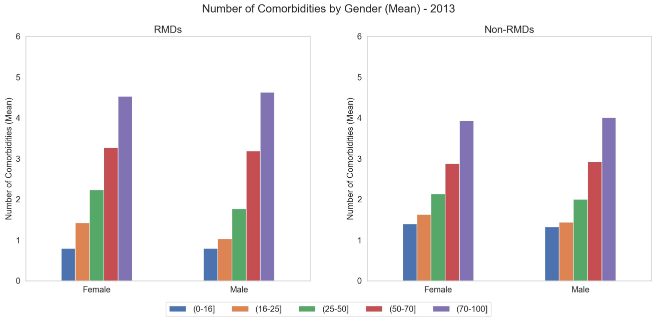

(d) Mean number of comorbidities in patients with RMDs and comparators by age categories and sex 2013

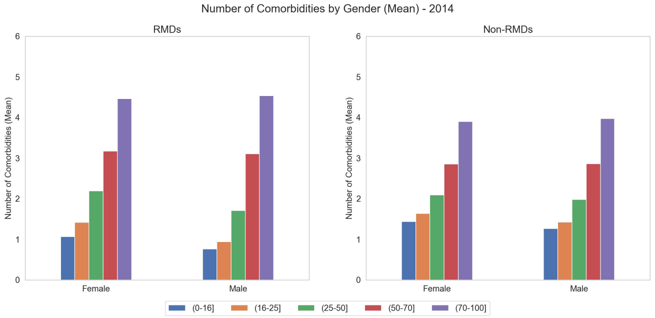

(e) Mean number of comorbidities in patients with RMDs and comparators by age categories and sex 2014

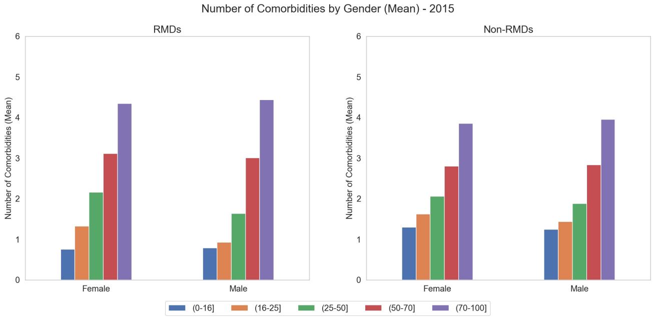

(f) Mean number of comorbidities in patients with RMDs and comparators by age categories and sex 2015

Figure 1. Mean number of comorbidities in patients with RMDs and comparators by age categories and sex 2010 and 2015

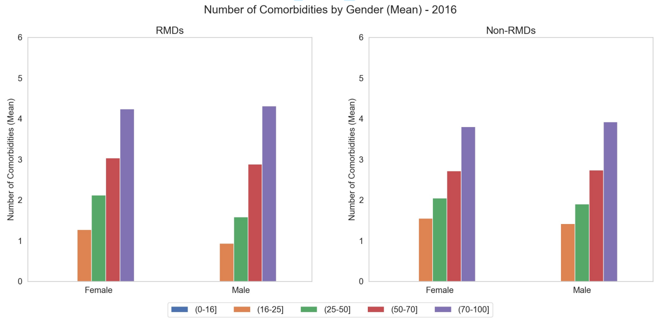

(g) Mean number of comorbidities in patients with RMDs and comparators by age categories and sex 2016

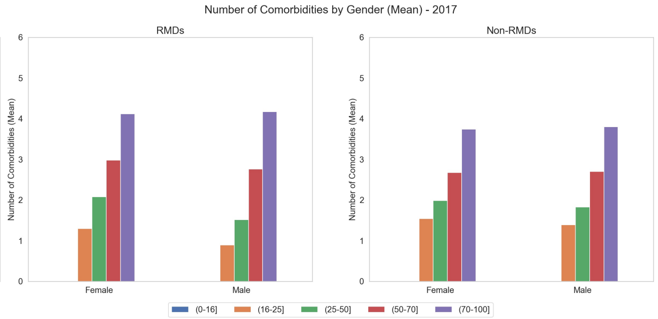

(h) Mean number of comorbidities in patients with RMDs and comparators by age categories and sex 2017

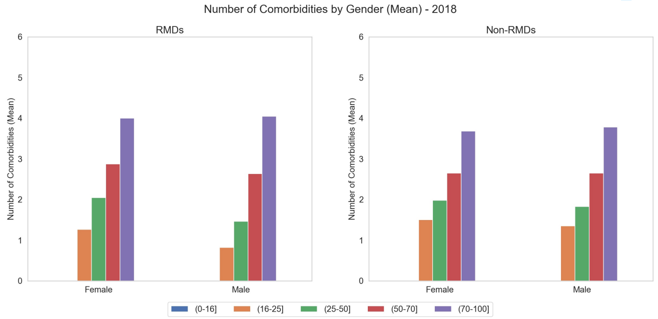

(i) Mean number of comorbidities in patients with RMDs and comparators by age categories and sex 2018

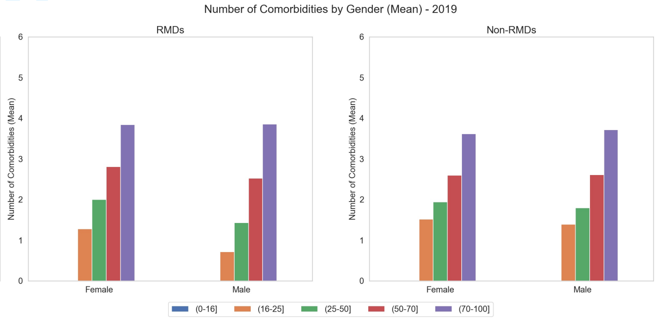

(j) Mean number of comorbidities in patients with RMDs and comparators by age categories and sex

Figure 1. Mean number of comorbidities in patients with RMDs and comparators by age categories and sex 2016 and 2018

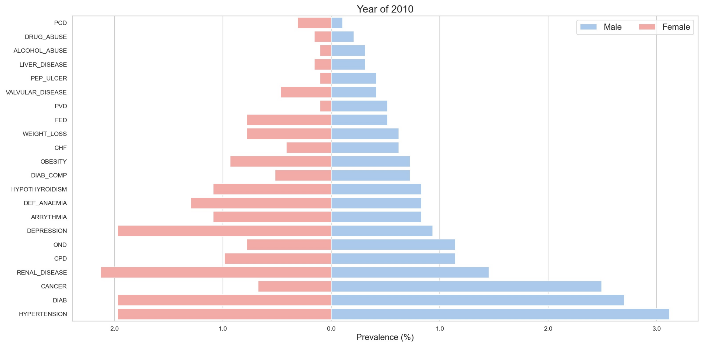

(a) Prevalence of Elixhuaser comorbidities patients with RMDs 2010

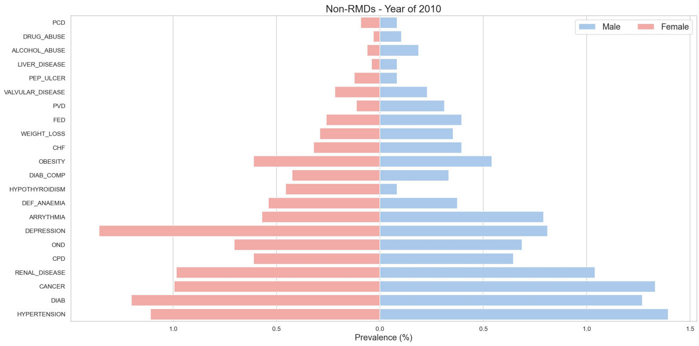

(b) Prevalence of Elixhuaser comorbidities patients with RMDs 2010

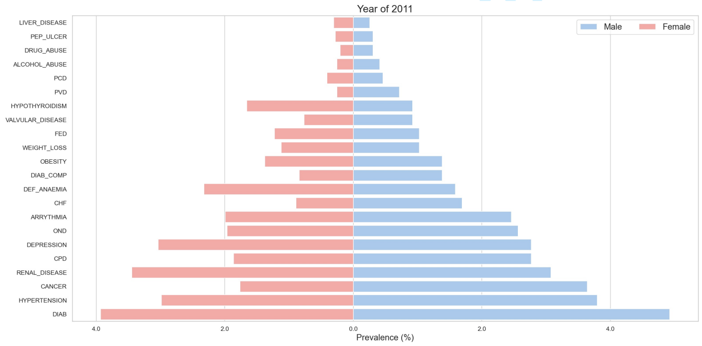

(c) Prevalence of Elixhauser comorbidities in comparators 2011

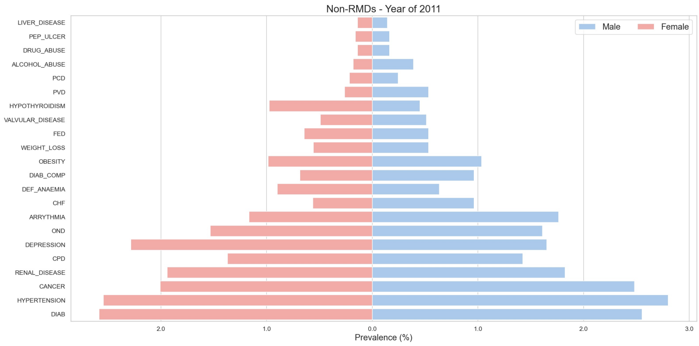

(d) Prevalence of Elixhauser comorbidities in comparators 2011

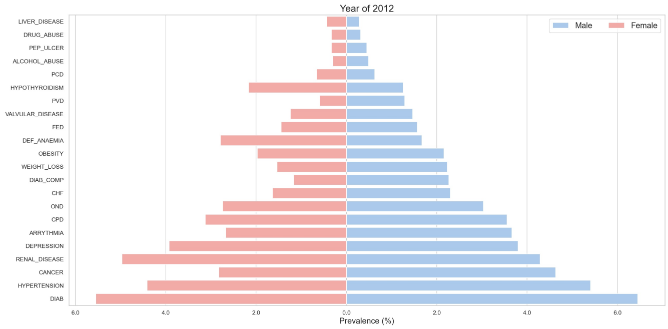

(e) Prevalence of Elixhuaser comorbidities patients with RMDs 2012

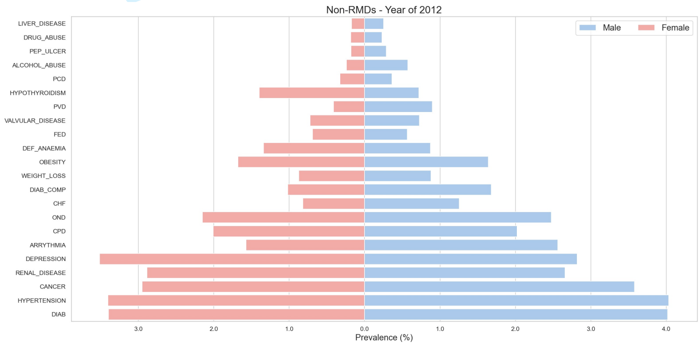

(f) Prevalence of Elixhauser comorbidities in comparators 2012

Figure 2. Prevalence of Elixhauser comorbidities in patients with RMDs and patients without RMDS in 2010 and 2012

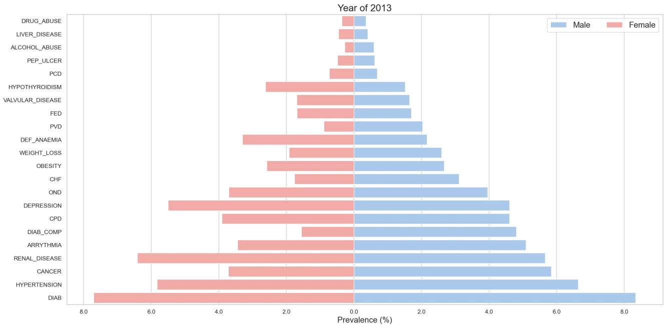

(g) Prevalence of Elixhuaser comorbidities patients with RMDs 2013

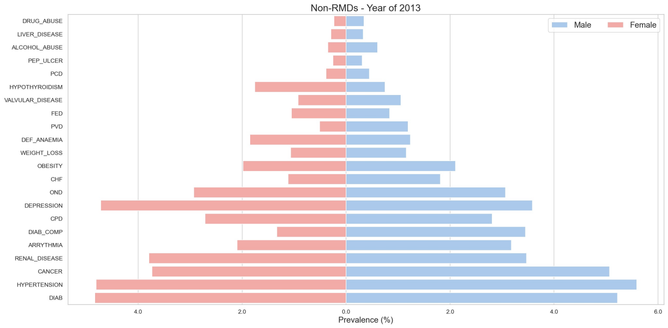

(h) Prevalence of Elixhuaser comorbidities patients without RMDs 2013

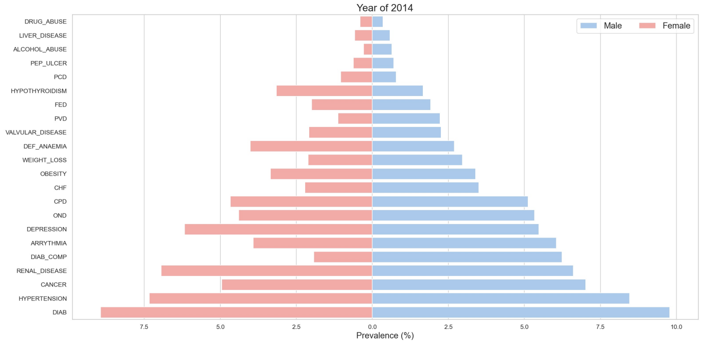

(i) Prevalence of Eixhuaser comorbidities patients with RMDs 2014

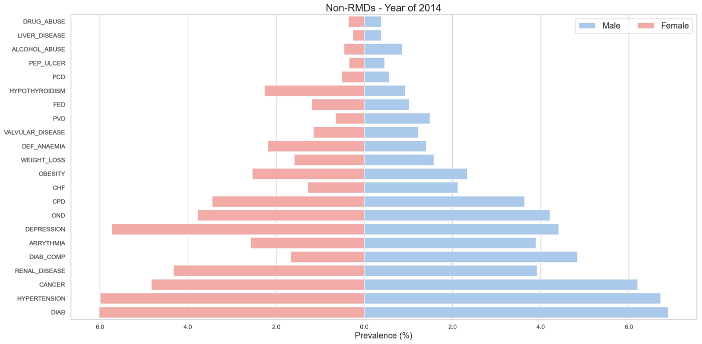

(j) Prevalence of Elixhuaser comorbidities patients with RMDs 2014

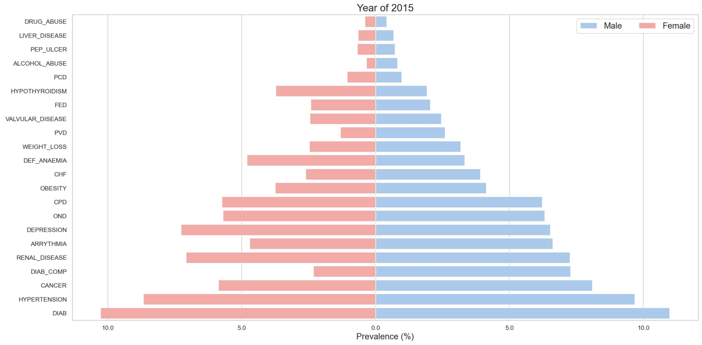

(k) Prevalence of Elixhuaser comorbidities patients without RMDs 2015

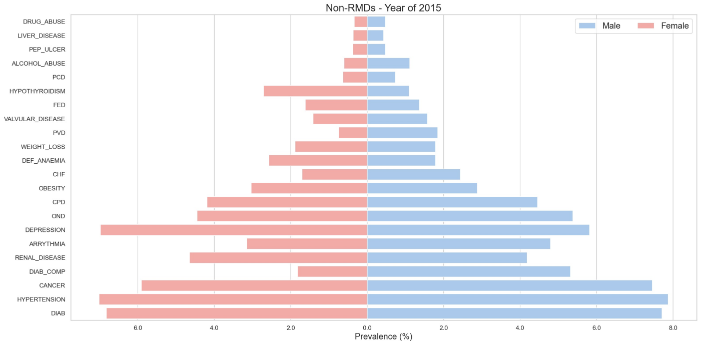

(l) Prevalence of Elixhuaser comorbidities patients with RMDs 2015

Figure 2. Prevalence of Elixhauser comorbidities in patients with RMDs and patients without RMDS in 2013 and 2015

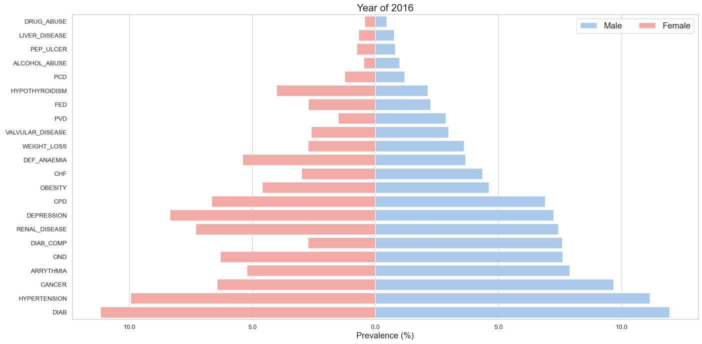

(m) Prevalence of Elixhuaser comorbidities patients with RMDs 2016

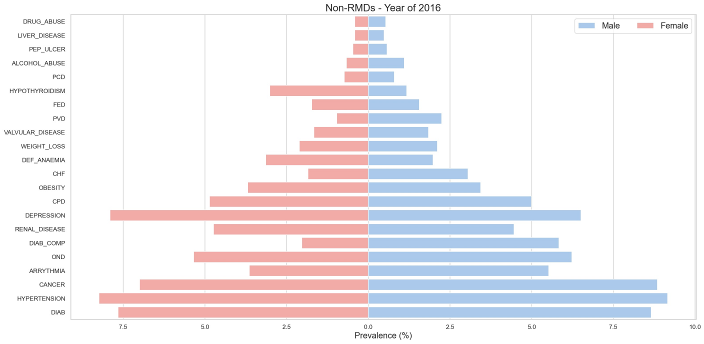

(n) Prevalence of Elixhuaser comorbidities patients with RMDs 2016

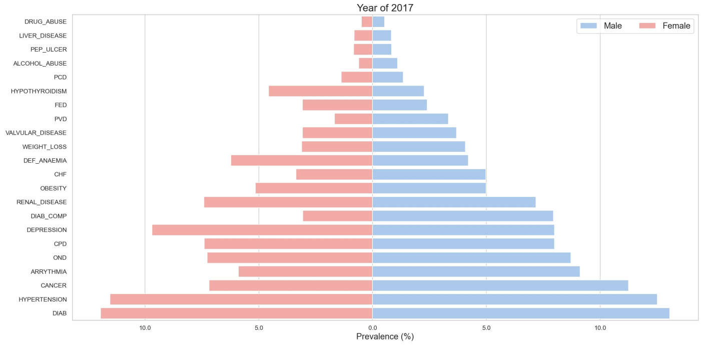

(o) Prevalence of Elixhuaser comorbidities patients without RMDs 2017

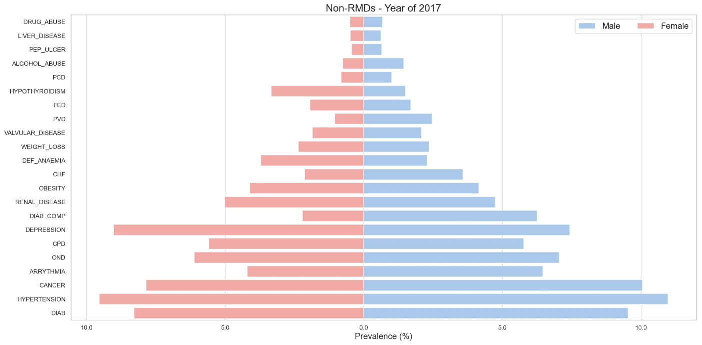

(p) Prevalence of Elixhuaser comorbidities patients with RMDs 2017

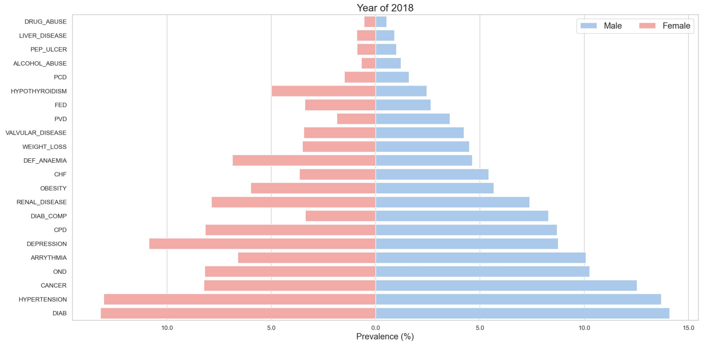

(q) Prevalence of Elixhuaser comorbidities patients without RMDs 2018

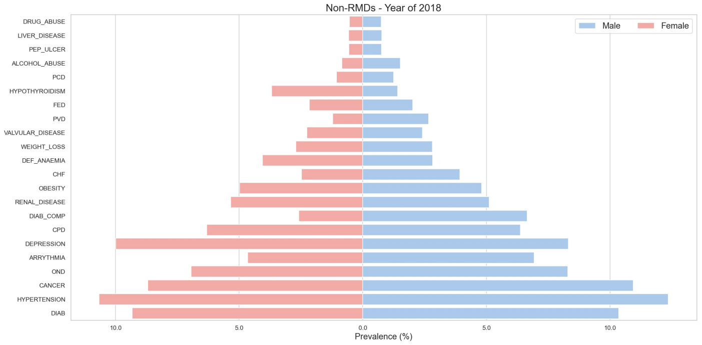

(r) Prevalence of Elixhuaser comorbidities patients with RMDs 2018

Figure 2. Prevalence of Elixhauser comorbidities in patients with RMDs and patients without RMDS in 2016 and 2018

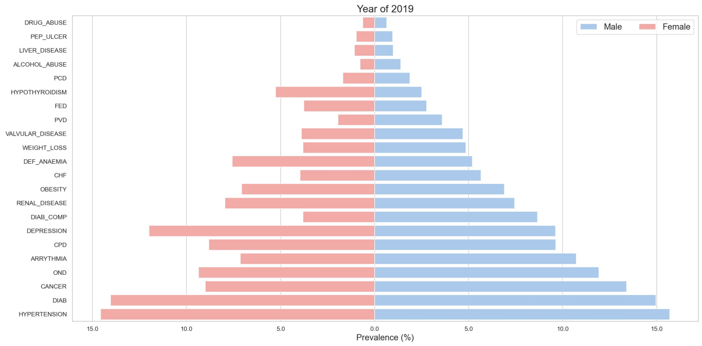

(s) Prevalence of Eixhuaser comorbidities patients with RMDs 2016

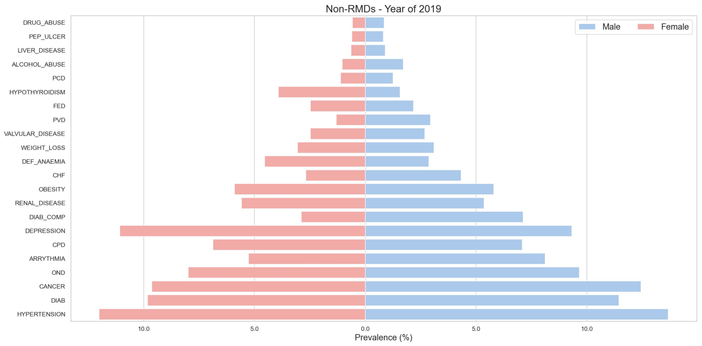

(t) Prevalence of Eixhuaser comorbidities patients with RMDs 2016

Figure 2. Prevalence of Elixhauser comorbidities in patients with RMDs and patients without RMDS in 2019

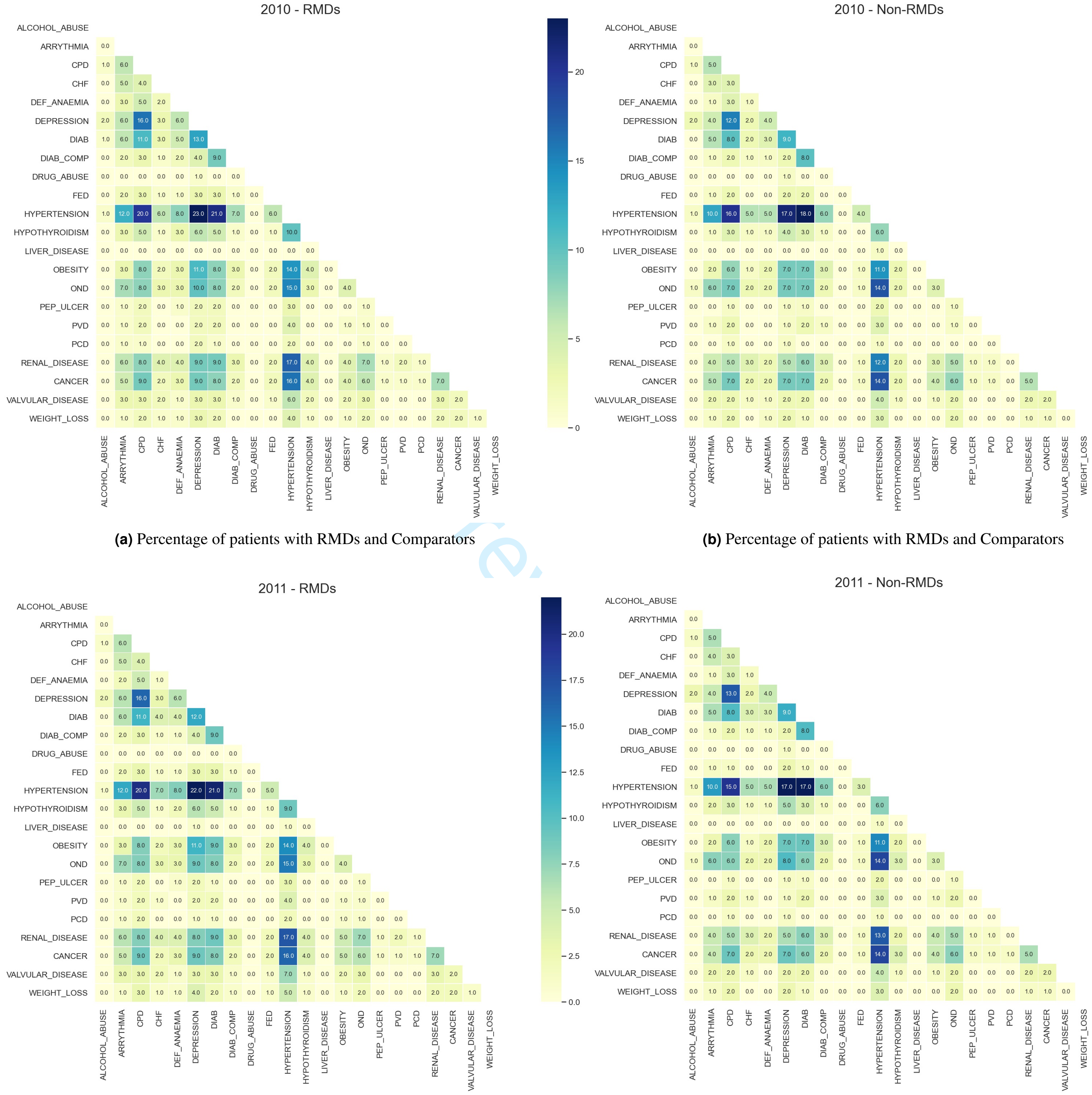

(a) Percentage of patients with RMDs and Comparators

(b) Percentage of patients with RMDs and Comparators

(c) Percentage of patients with RMDs and Comparators

(d) Percentage of patients with RMDs and Comparators

Figure 3. Proportion of patients with RMDs and Comparators

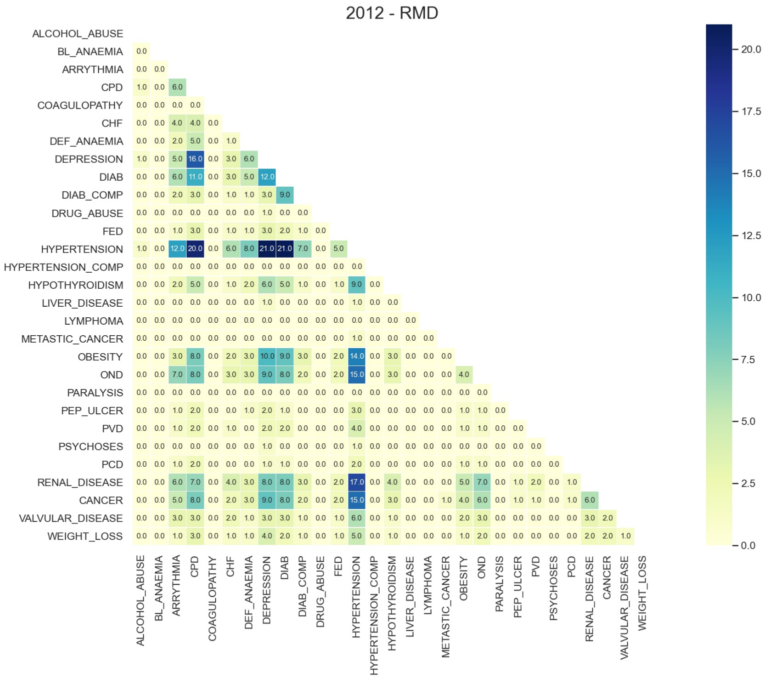

(e) Percentage of patients with RMDs and Comparators

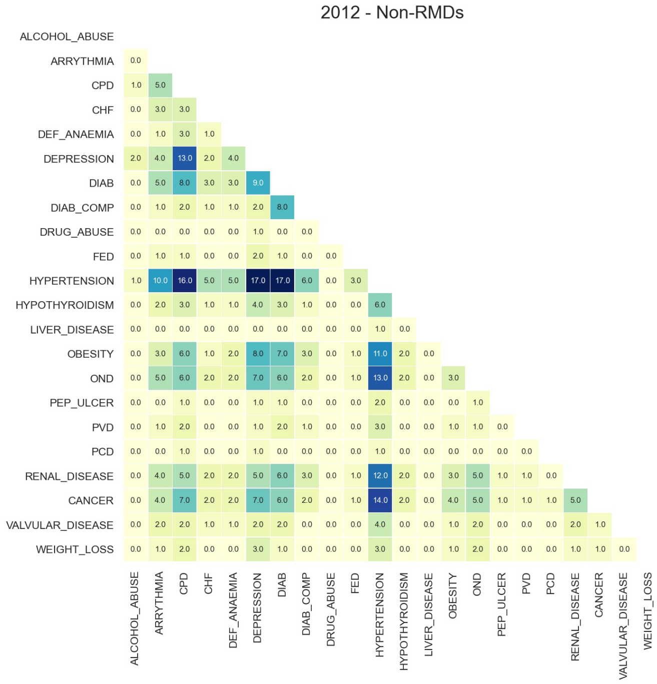

(f) Percentage of patients with RMDs and Comparators

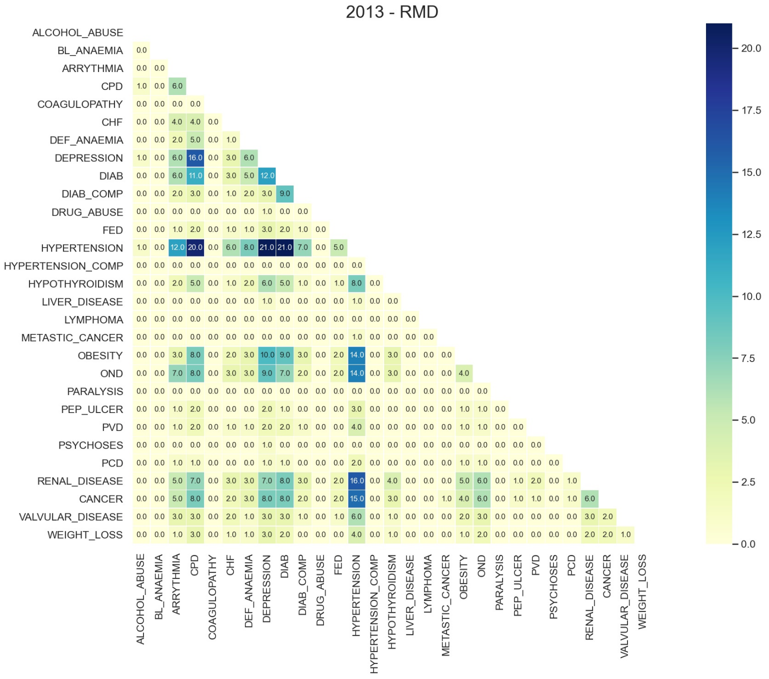

(g) Percentage of patients with RMDs and Comparators

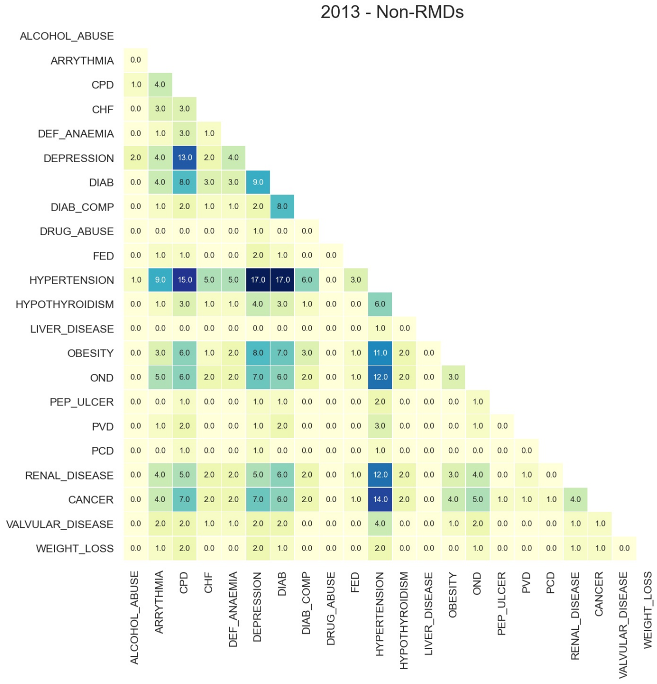

(h) Percentage of patients with RMDs and Comparators

Figure 3. Proportion of patients with RMDs an Comparators

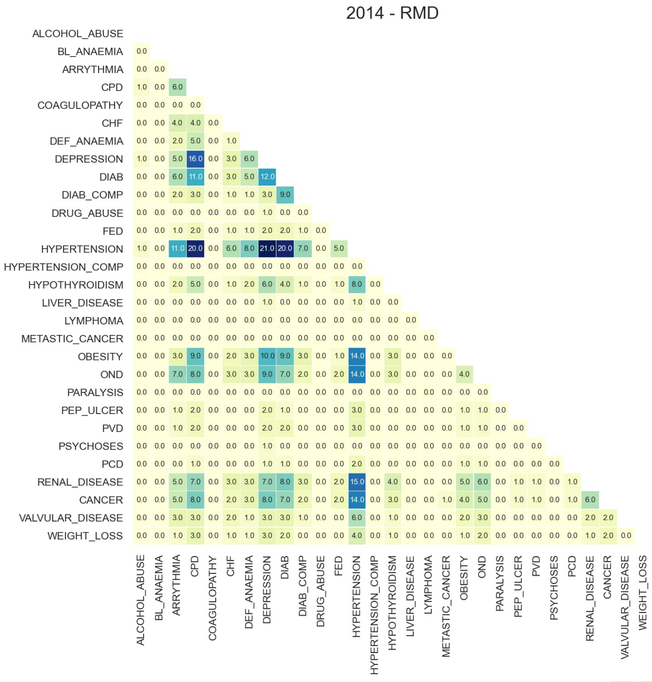

(i) Percentage of patients with RMDs and Comparators

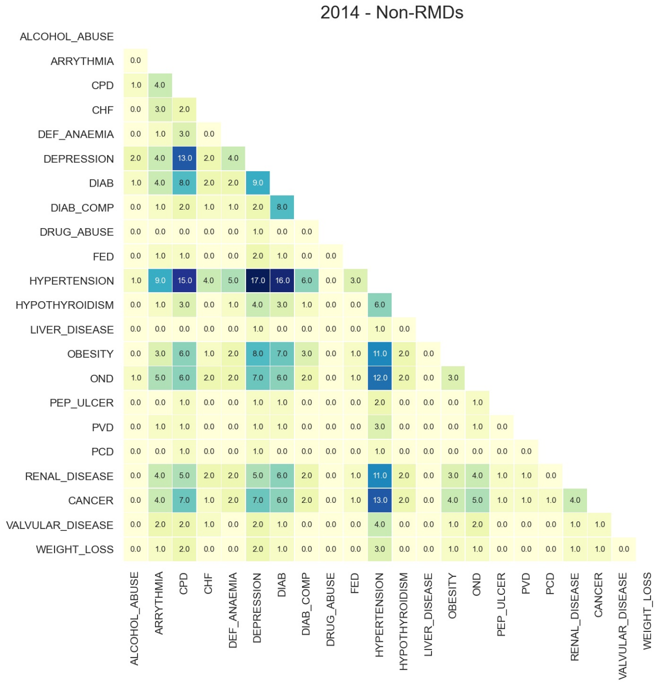

(j) Percentage of patients with RMDs and Comparators

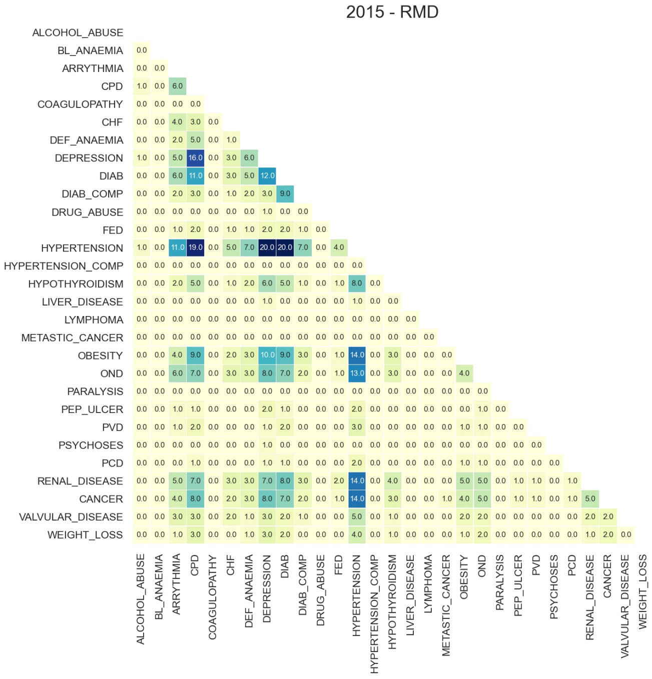

(k) Percentage of patients with RMDs and Comparators

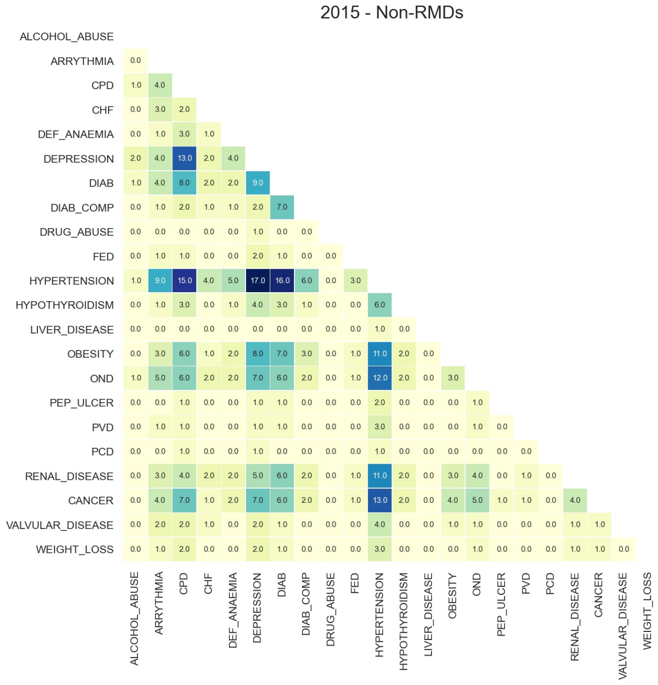

(l) Percentage of patients with RMDs and Comparators

Figure 3. Proportion of patients with RMDs an Comparators

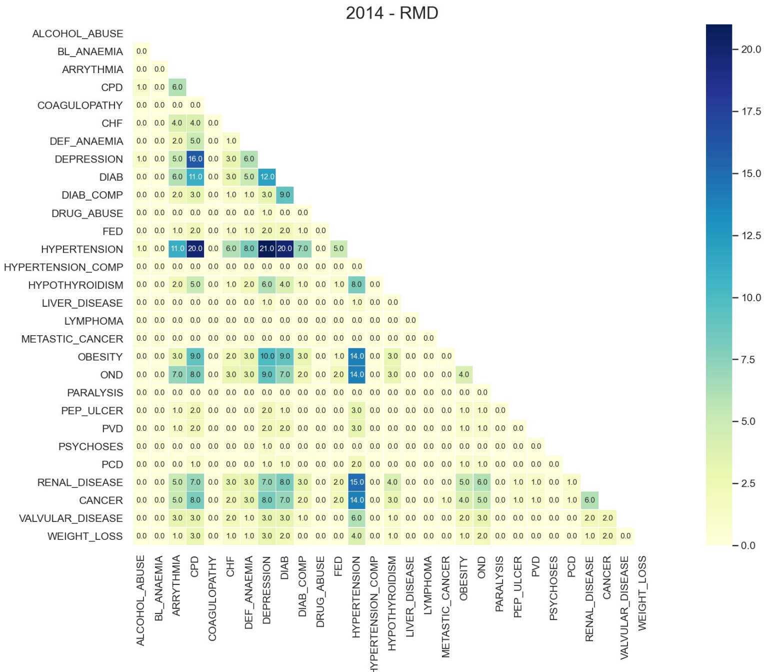

(m) Percentage of patients with RMDs and Comparators

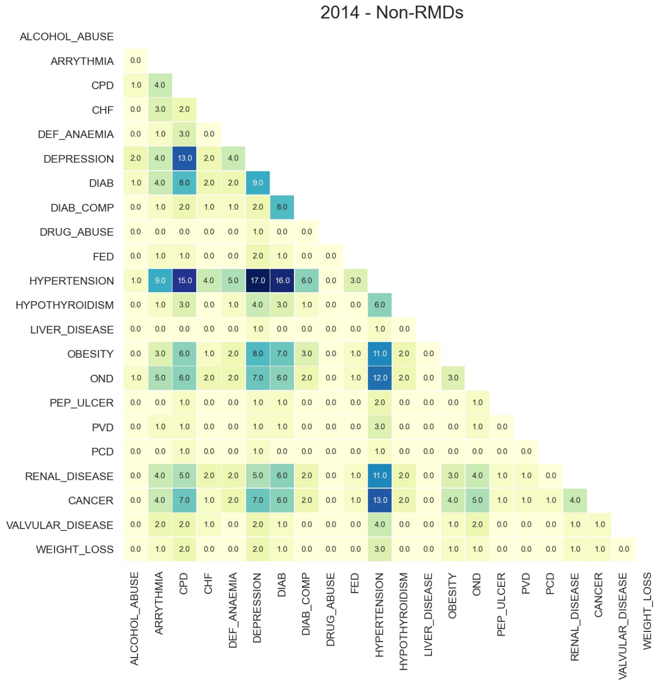

(n) Percentage of patients with RMDs and Comparators

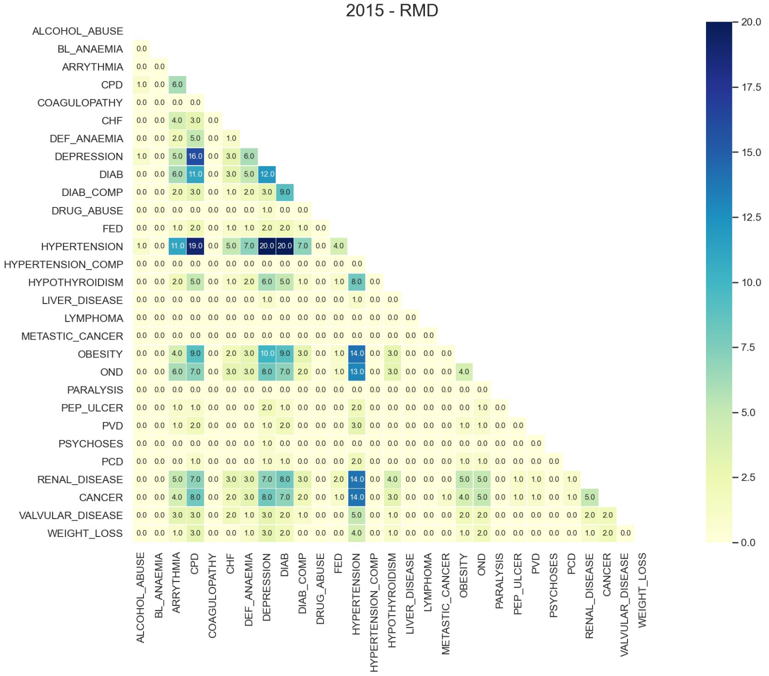

(o) Percentage of patients with RMDs and Comparators

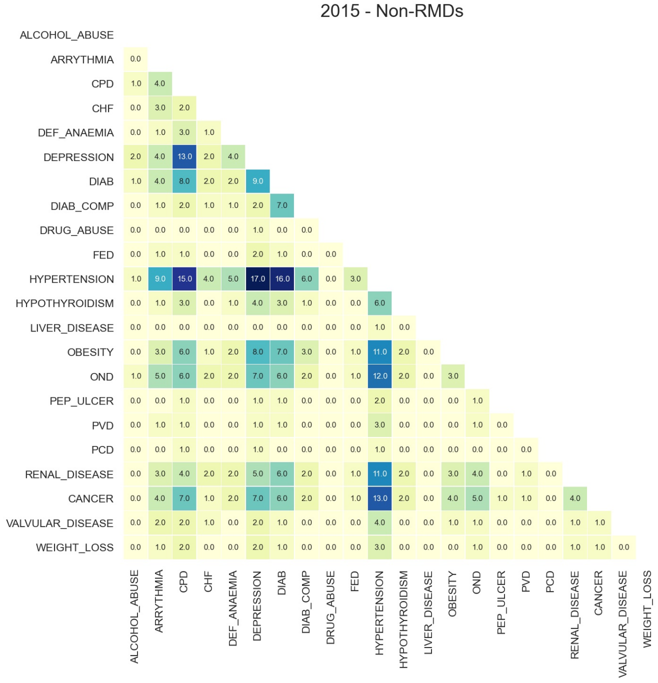

(p) Percentage of patients with RMDs and Comparators

Figure 3. Proportion of patients with RMDs and Comparators

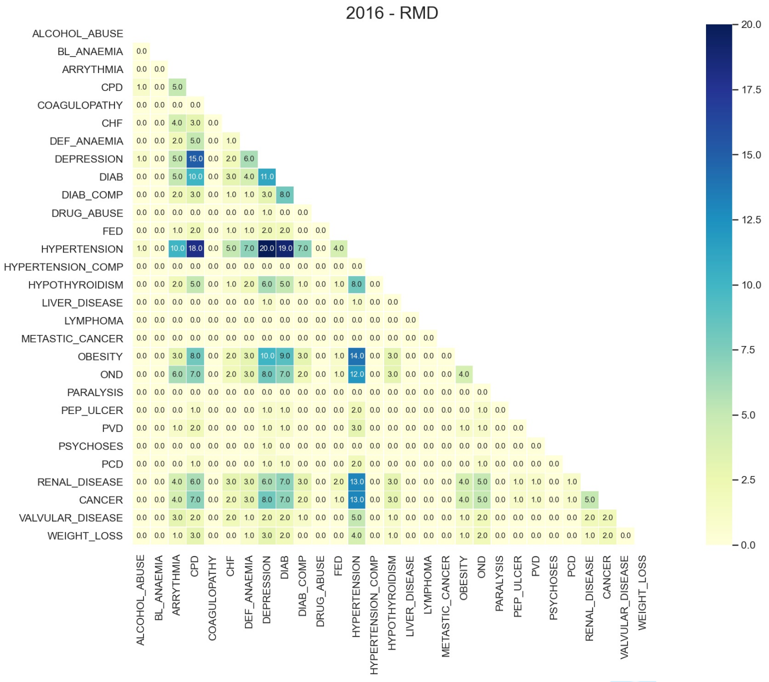

(q) Percentage of patients with RMDs and Comparators

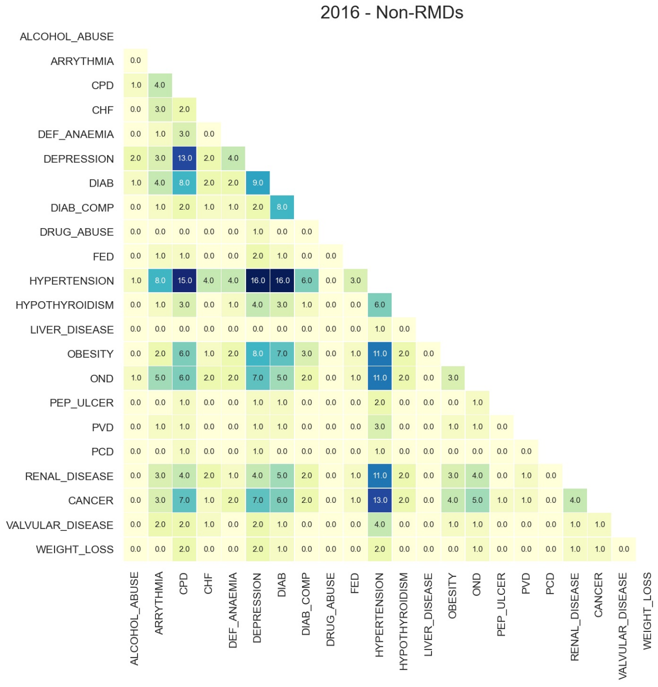

(r) Percentage of patients with RMDs and Comparators

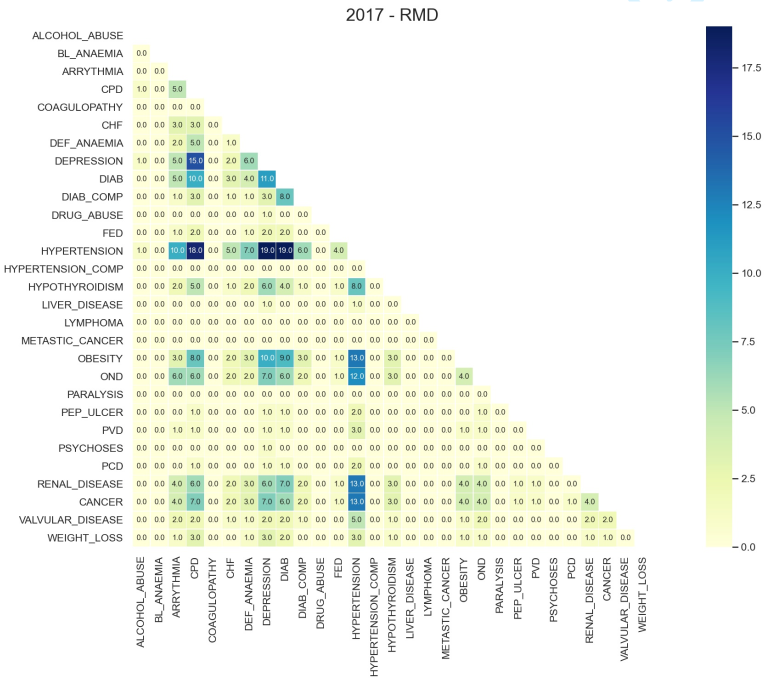

(s) Percentage of patients with RMDs and Comparators

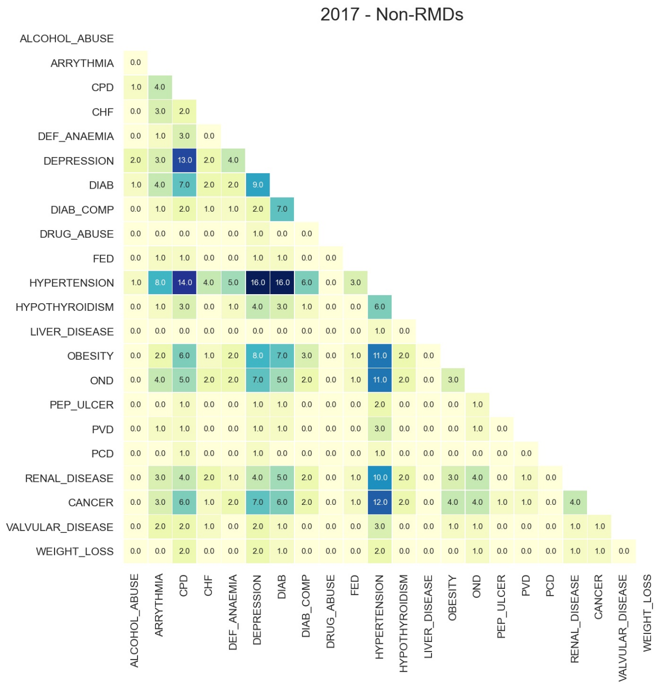

(t) Percentage of patients with RMDs and Comparators

Figure 3. Proportion of patients with RMDs and Comparators

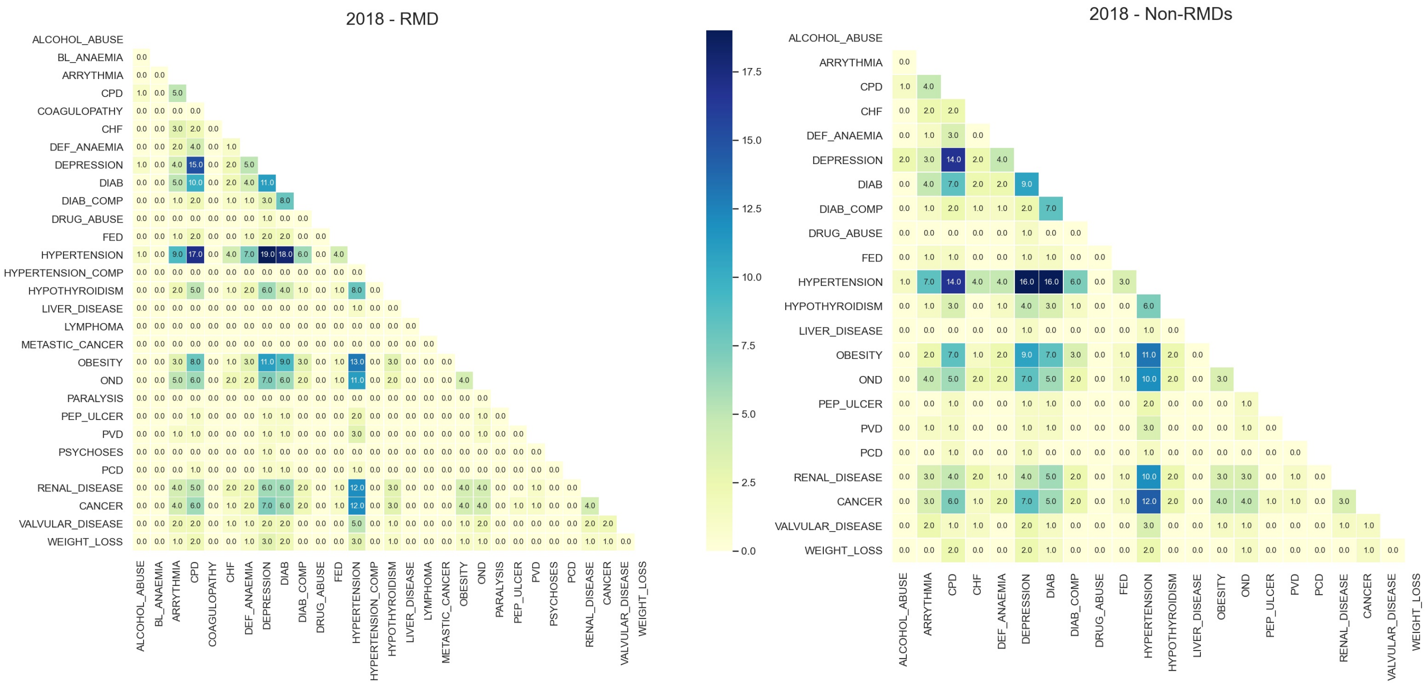

(u) Percentage of patients with RMDs and Comparators

(v) Percentage of patients with RMDs and Comparators

Figure 3. Proportion of patients with RMDs and Comparators

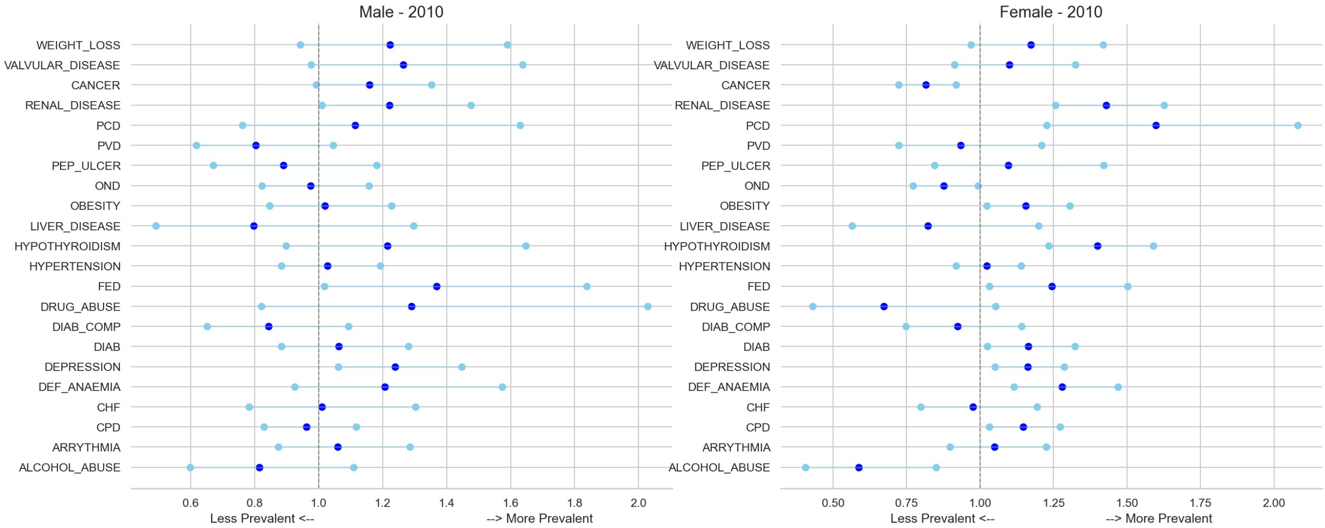

(a) ORs for association of RMDs with comorbidities by gender in 2010

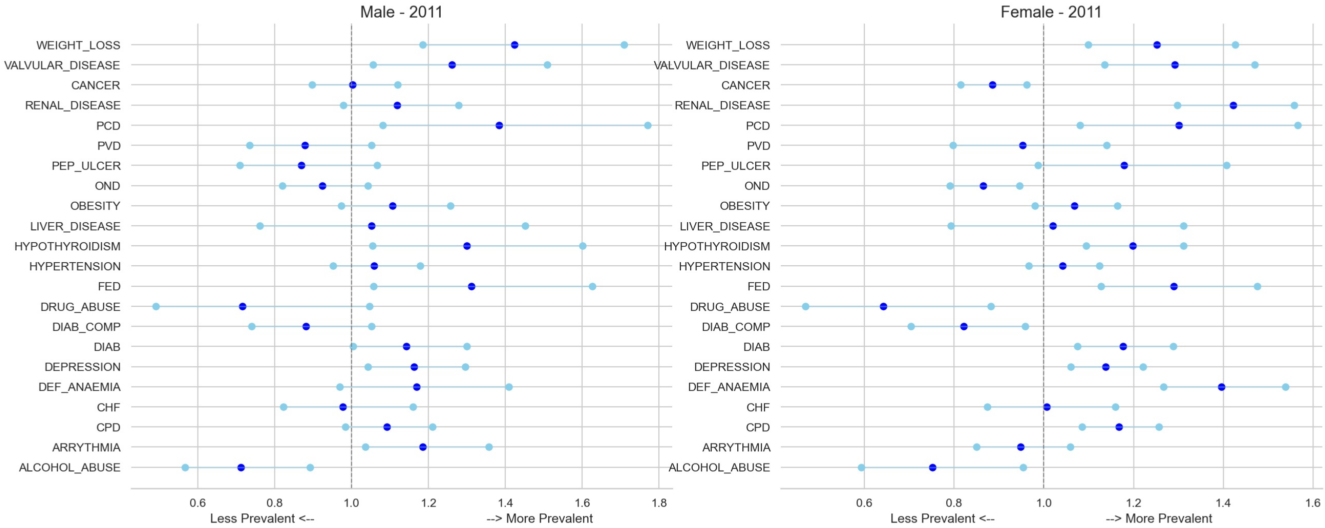

(b) Percentage of patients with RMDs and Comparators

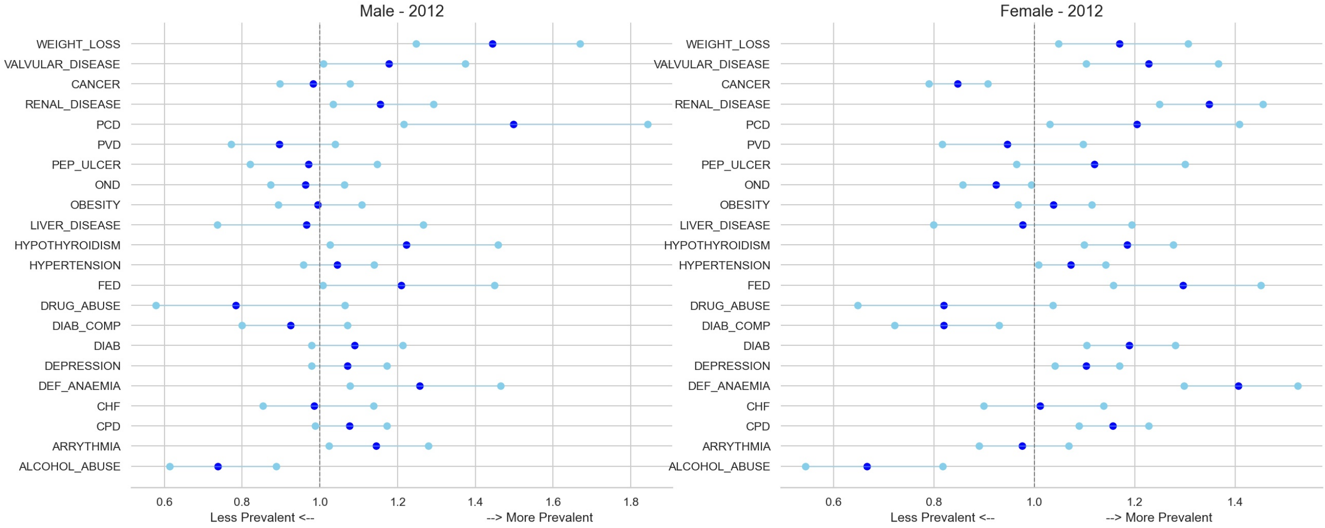

(c) Percentage of patients with RMDs an Comparators

Figure 3. ORs for association of RMDs with comorbidities by sex in 2012-2013

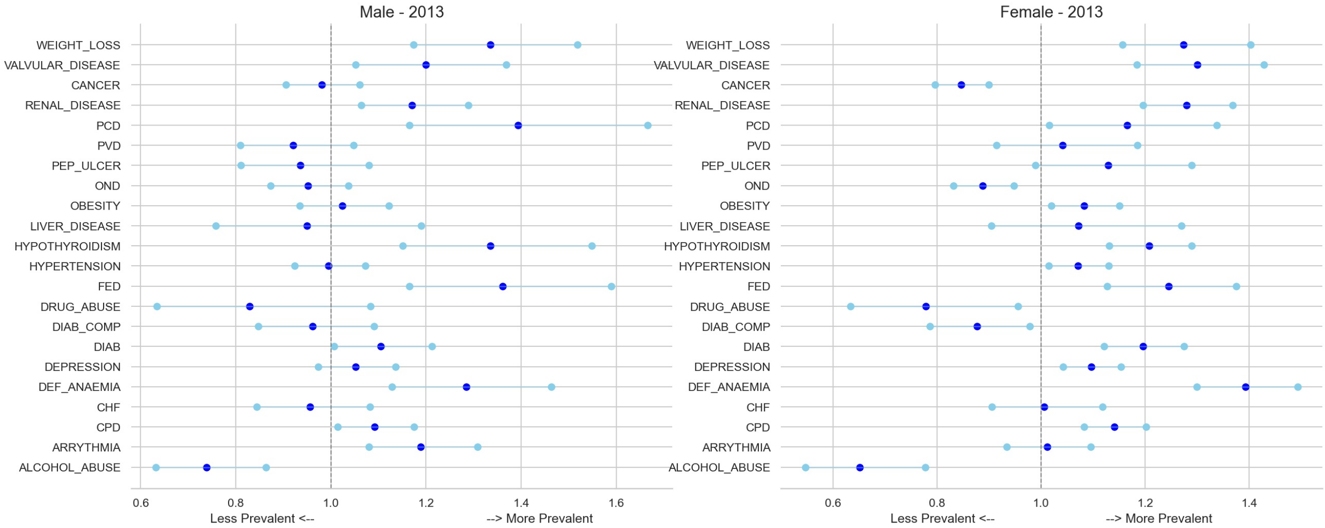

(d) Percentage of patients with RMDs and Comparators

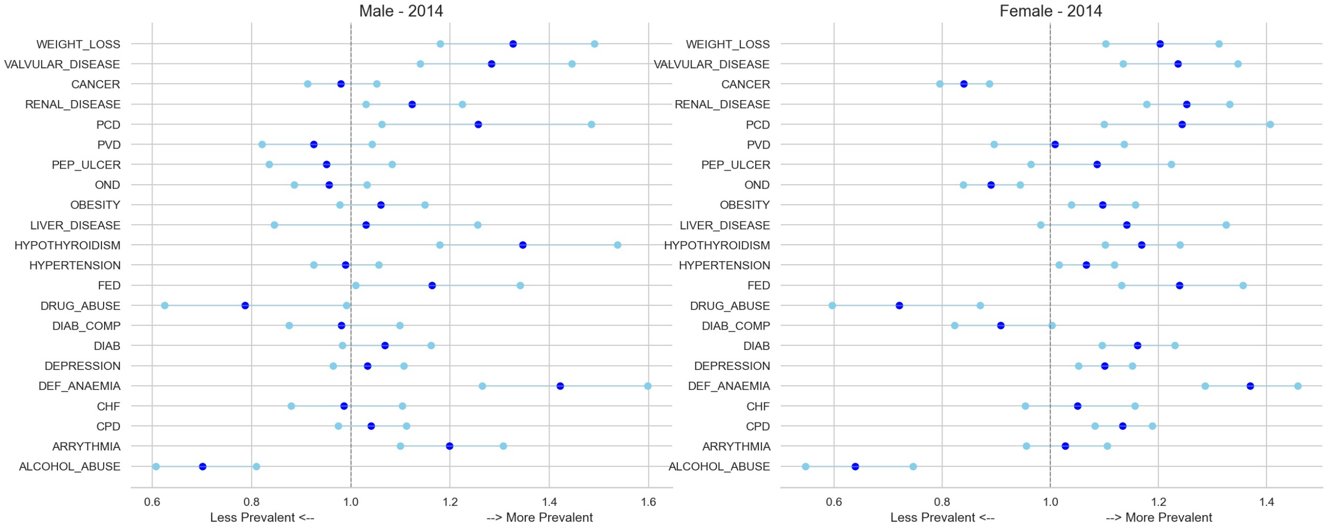

(e) Percentage of patients with RMDs an Comparators

Figure 3. ORs for association of RMDs with comorbidities by sex in 2013-2014

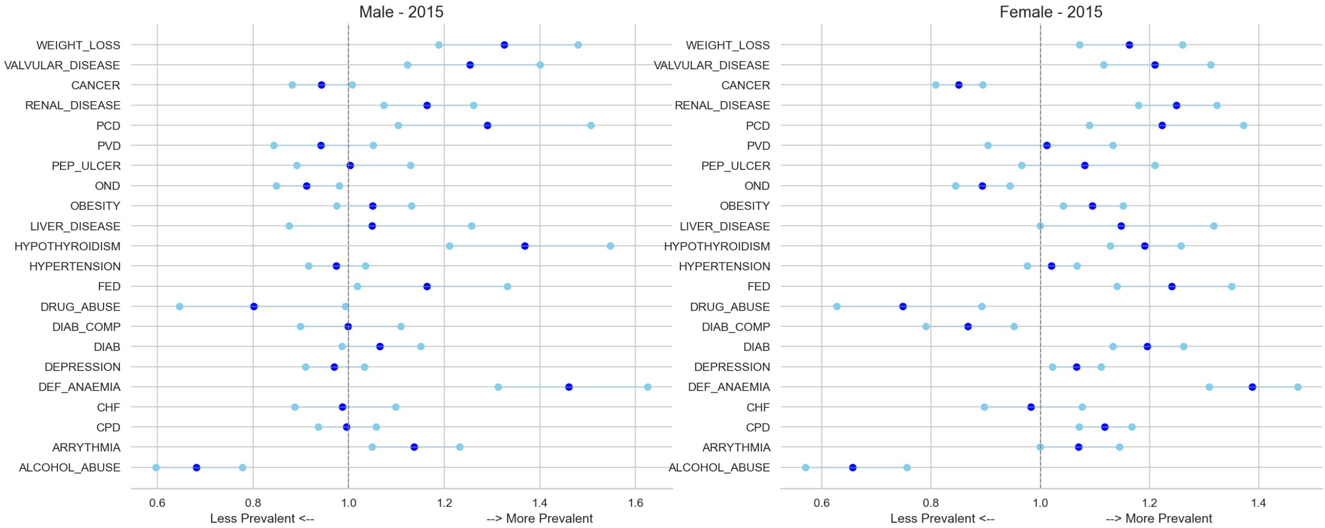

(f) Percentage of patients with RMDs and Comparators

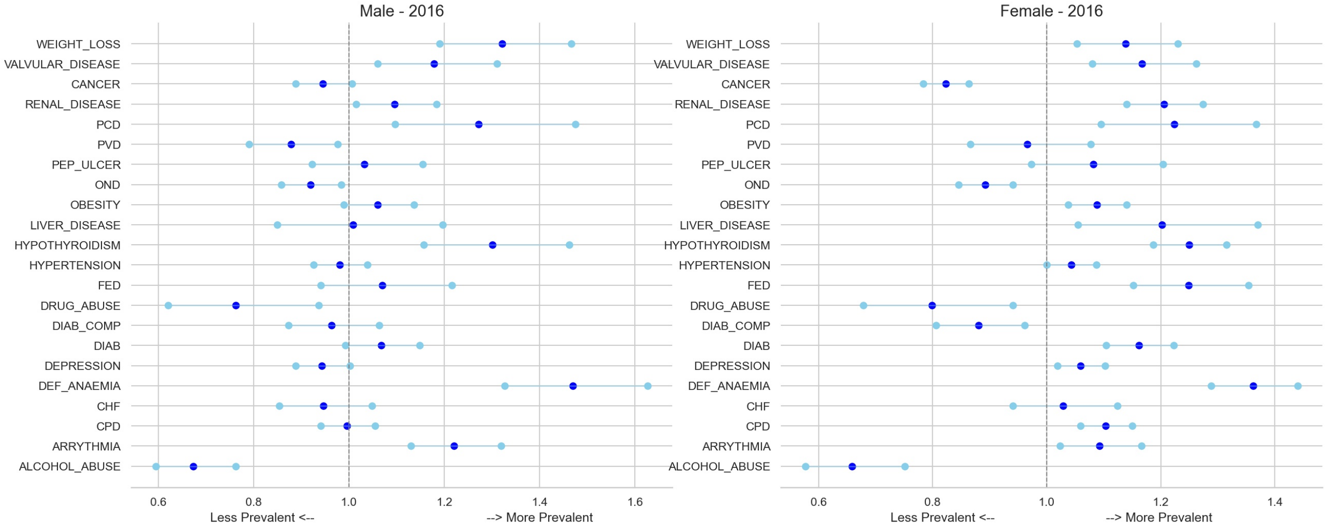

(g) Percentage of patients with RMDs an Comparators

Figure 3. ORs for association of RMDs with comorbidities by sex in 2015-2016

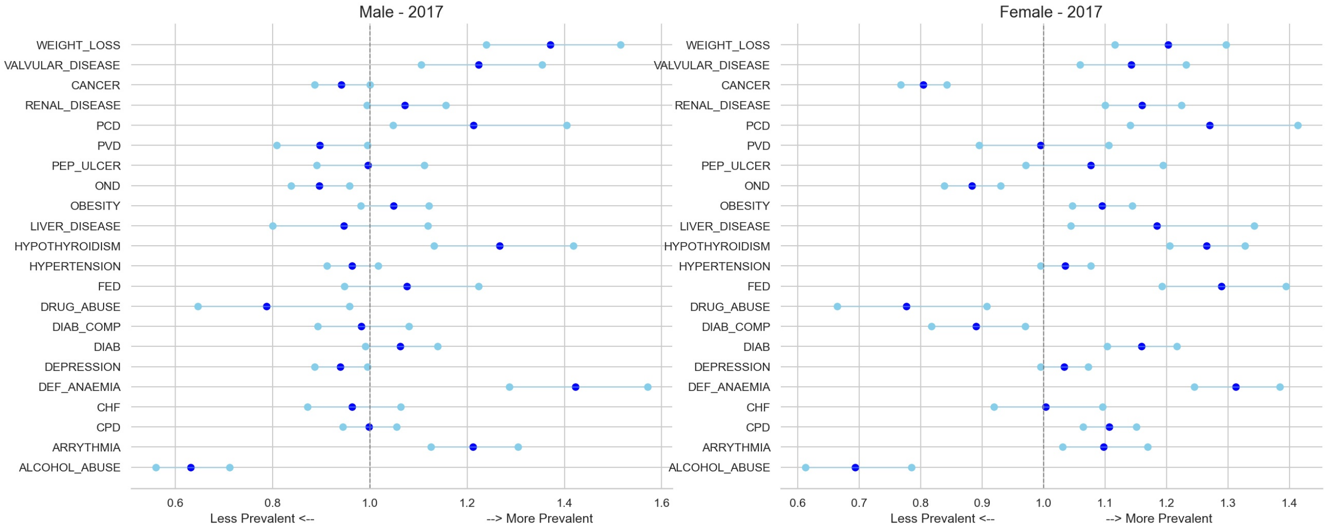

(h) Percentage of patients with RMDs and Comparators

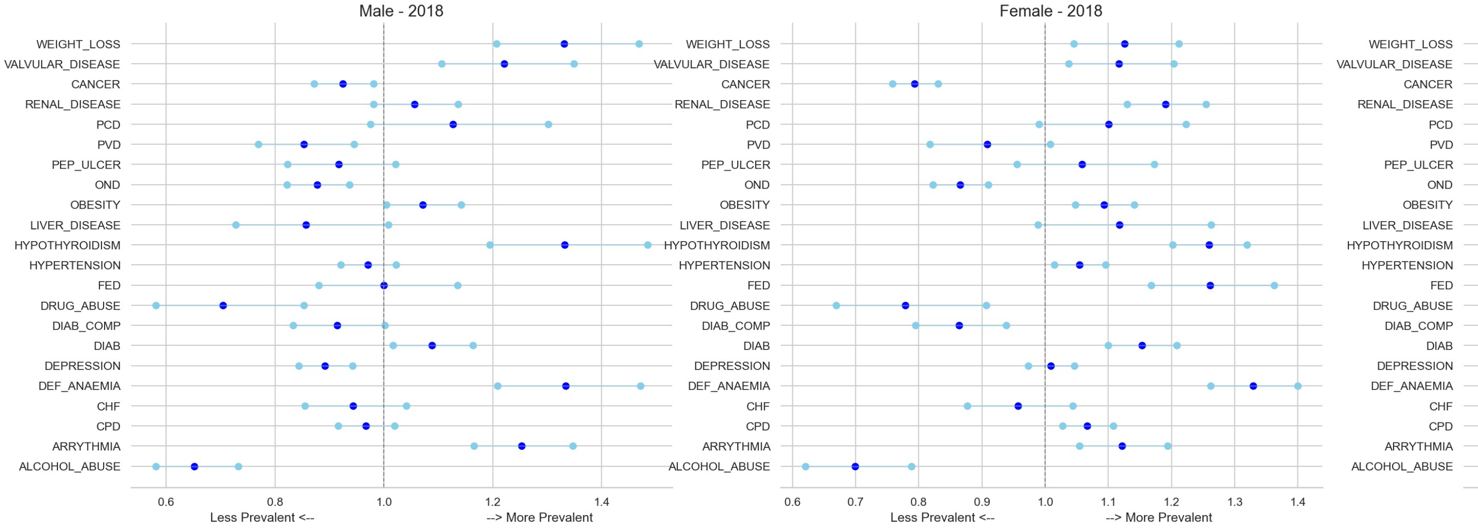

(i) Percentage of patients with RMDs and Comparators

Figure 3. ORs for association of RMDs with comorbidities by sex in 2017-2018

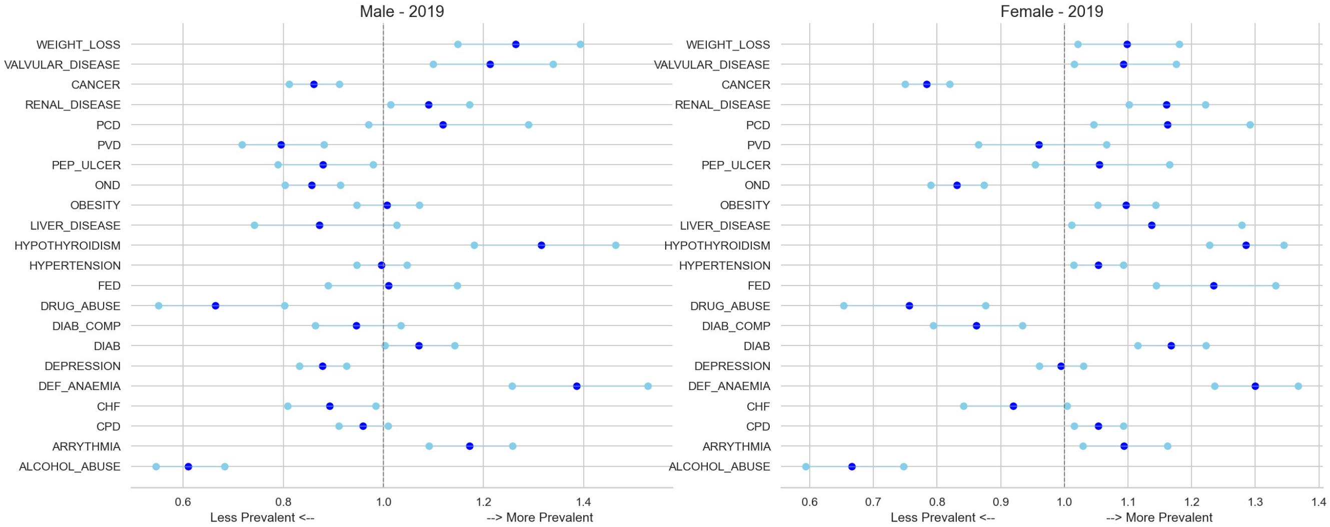

(k) Percentage of patients with RMDs and Comparators

Figure 3. ORs for association of RMDs with comorbidities by sex in 2019

For peer review only

Reporting checklist for cohort study.

Based on the STROBE cohort guidelines.

Instructions to authors

Complete this checklist by entering the page numbers from your manuscript where readers will find each of the items listed below.

Your article may not currently address all the items on the checklist. Please modify your text to include the missing information. If you are certain that an item does not apply, please write "n/a" and provide a short explanation.

Upload your completed checklist as an extra file when you submit to a journal.

In your methods section, say that you used the STROBE cohortreporting guidelines, and cite them as:

von Elm E, Altman DG, Egger M, Pocock SJ, Gotzsche PC, Vandenbroucke JP. The Strengthening the Reporting of Observational Studies in Epidemiology (STROBE) Statement: guidelines for reporting observational studies.

		Reporting Item	Page Number
**Title and abstract**			
Title	#1a	Indicate the study's design with a commonly used term in the title or the abstract	1
Abstract	#1b	Provide in the abstract an informative and balanced summary of what was done and what was found	2
**Introduction**			
Background / rationale	#2	Explain the scientific background and rationale for the investigation being reported	4,5
Objectives	#3	State specific objectives, including any prespecified hypotheses	1
**Methods**			
Study design	#4	Present key elements of study design early in the paper	2,4

For peer review only

Setting	#5	Describe the setting, locations, and relevant dates, including periods of recruitment, exposure, follow-up, and data collection	2,4
Eligibility criteria	#6a	Give the eligibility criteria, and the sources and methods of selection of participants. Describe methods of follow-up.	2,4
Eligibility criteria	#6b	For matched studies, give matching criteria and number of exposed and unexposed	4
Variables	#7	Clearly define all outcomes, exposures, predictors, potential confounders, and effect modifiers. Give diagnostic criteria, if applicable	5
Data sources / measurement	#8	For each variable of interest give sources of data and details of methods of assessment (measurement). Describe comparability of assessment methods if there is more than one group. Give information separately for for exposed and unexposed groups if applicable.	2,4
Bias	#9	Describe any efforts to address potential sources of bias	n/a
Study size	#10	Explain how the study size was arrived at	2,4
Quantitative variables	#11	Explain how quantitative variables were handled in the analyses. If applicable, describe which groupings were chosen, and why	4
Statistical methods	#12a	Describe all statistical methods, including those used to control for confounding	
5			
Statistical methods	#12b	Describe any methods used to examine subgroups and interactions	n/a
Statistical methods	#12c	Explain how missing data were addressed	n/a
Statistical methods	#12d	If applicable, explain how loss to follow-up was addressed	5
Statistical methods	#12e	Describe any sensitivity analyses	
n/a			

Results

For peer review only

Participants	#13a	Report numbers of individuals at each stage of study—eg numbers potentially eligible, examined for eligibility, confirmed eligible, included in the study, completing follow-up, and analysed. Give information separately for for exposed and unexposed groups if applicable.	2,4
Participants	#13b	Give reasons for non-participation at each stage	n/a
Participants	#13c	Consider use of a flow diagram	
5			
Descriptive data	#14a	Give characteristics of study participants (eg demographic, clinical, social) and information on exposures and potential confounders. Give information separately for exposed and unexposed groups if applicable.	5
Descriptive data	#14b	Indicate number of participants with missing data for each variable of interest	
n/a			
Descriptive data	#14c	Summarise follow-up time (eg, average and total amount)	
4			
Outcome data	#15	Report numbers of outcome events or summary measures over time. Give information separately for exposed and unexposed groups if applicable.	
4			
Main results	#16a	Give unadjusted estimates and, if applicable, confounder-adjusted estimates and their precision (eg, 95% confidence interval). Make clear which confounders were adjusted for and why they were included	5
Main results	#16b	Report category boundaries when continuous variables were categorized	n/a
Main results	#16c	If relevant, consider translating estimates of relative risk into absolute risk for a meaningful time period	
n/a			

For peer review only

Other analyses	#17	Report other analyses done—eg analyses of subgroups and interactions, and sensitivity analyses	n/a
**Discussion**			
Key results	#18	Summarise key results with reference to study objectives	10
Limitations	#19	Discuss limitations of the study, taking into account sources of potential bias or imprecision. Discuss both direction and magnitude of any potential bias.	10
Interpretation	#20	Give a cautious overall interpretation considering objectives, limitations, multiplicity of analyses, results from similar studies, and other relevant evidence.	10
Generalisability	#21	Discuss the generalisability (external validity) of the study results	na
**Other Information**			
Funding	#22	Give the source of funding and the role of the funders for the present study and, if applicable, for the original study on which the present article is based	14

The STROBE checklist is distributed under the terms of the Creative Commons Attribution License CC-BY. This checklist was completed on 09. August 2023 using https://www.goodreports.org/, a tool made by the EQUATOR Network in collaboration with Penelope.ai

For peer review only

BMJ Open

10-year multi-morbidity patterns among people with and without rheumatic and musculoskeletal diseases: An observational cohort study using linked electronic health records from Wales, UK

Journal:	*BMJ Open*
Manuscript ID	bmjopen-2023-079169.R1
Article Type:	Original research
Date Submitted by the Author:	26-Mar-2024
Complete List of Authors:	Jalali-najafabadi, Farideh; The University of Manchester,<br>Bailey, Rowena; Swansea University Medical School, Population Data Science<br>Lyons, Jane; Swansea University Medical School,<br>Akbari, Ashley; Swansea University Medical School,<br>Badhafari, Tthamer; The University of Manchester<br>Azadbakht, Narges; The University of Manchester<br>Rafferty, James; Swansea University Medical School<br>Watkins, Alan; Swansea University, College of Medicine<br>Martin, Glen; University of Manchester Institute of Population Health, Farr Institute<br>Bowes, John; The University of Manchester<br>Lyons, Ronan; Swansea University, Swansea Clinical School<br>Barton, Anne; The University of Manchester, Centre for Musculoskeletal Research<br>Peek, N; The University of Manchester, Division of Informatics, Imaging and Data Science
**Primary Subject Heading**:	Rheumatology
Secondary Subject Heading:	Epidemiology
Keywords:	Electronic Health Records, RHEUMATOLOGY, Epidemiology < ONCOLOGY

SCHOLARONE™
Manuscripts

I, the Submitting Author has the right to grant and does grant on behalf of all authors of the Work (as defined in the below author licence), an exclusive licence and/or a non-exclusive licence for contributions from authors who are: i) UK Crown employees; ii) where BMJ has agreed a CC-BY licence shall apply, and/or iii) in accordance with the terms applicable for US Federal Government officers or employees acting as part of their official duties; on a worldwide, perpetual, irrevocable, royalty-free basis to BMJ Publishing Group Ltd ("BMJ") its licensees and where the relevant Journal is co-owned by BMJ to the co-owners of the Journal, to publish the Work in this journal and any other BMJ products and to exploit all rights, as set out in our licence.

The Submitting Author accepts and understands that any supply made under these terms is made by BMJ to the Submitting Author unless you are acting as an employee on behalf of your employer or a postgraduate student of an affiliated institution which is paying any applicable article publishing charge ("APC") for Open Access articles. Where the Submitting Author wishes to make the Work available on an Open Access basis (and intends to pay the relevant APC), the terms of reuse of such Open Access shall be governed by a Creative Commons licence – details of these licences and which Creative Commons licence will apply to this Work are set out in our licence referred to above.

Other than as permitted in any relevant BMJ Author's Self Archiving Policies, I confirm this Work has not been accepted for publication elsewhere, is not being considered for publication elsewhere and does not duplicate material already published. I confirm all authors consent to publication of this Work and authorise the granting of this licence.

For peer review only

10-year multi-morbidity patterns among people with and without rheumatic and musculoskeletal diseases: An observational cohort study using linked electronic health records from Wales, UK

Farideh Jalali-najafabadi[1+], Rowena Bailey[2], Jane Lyons[2], Ashley Akbari[2], Thamer Ba Dhafari[3], Narges Azadbakht[3], James Rafferty[2], Alan Watkins[2], Glen P. Martin[3], John Bowes[1,4], Ronan A. Lyons[2], Anne Barton[1,4], and Niels Peek[3,5,6]

[1]Centre for Genetics and Genomics Versus Arthritis, Centre for Musculoskeletal Research, Faculty of Biology, Medicine and Health, Manchester Academic Health Science Centre, The University of Manchester, Manchester, UK.
[2]Population Data Science, Health Data Research-UK, Faculty of Medicine, Health and Life Science, Swansea University, Swansea, UK.
[3]Division of Informatics, Imaging and Data Science, School of Health Sciences, The University of Manchester, Manchester, UK. [4]NIHR Manchester Musculoskeletal Biomedical Research Unit, Central Manchester NHS Foundation Trust, Manchester Academic Health Science Centre, UK.
[5]Alan Turing Institute, London, UK.
[6]NIHR Manchester Biomedical Research Centre, Manchester University NHS Foundation Trust, Manchester Academic Health Science Centre, UK.
[+]farideh.jalali@manchester.ac.uk

For peer review only

For peer review only - http://bmjopen.bmj.com/site/about/guidelines.xhtml

ABSTRACT

Objectives

To compare the patterns of multi-morbidity between people with and without rheumatic and musculoskeletal diseases (RMDs) and to describe how these patterns change by age and sex over time, between 2010 and 2019.

Participants

103,426 people with RMDs and 2.9 million comparators registered in 395 Wales general practices. Each patient with an RMDs aged 0- 100 years between January 2010 and December 2019 registered in Clinical Practice Research Welsh practices was matched with up to five comparators without an RMD, based on age, gender, and general practice (GP) code.

Primary outcome measures

Prevalence of 29 Elixhuaser-defined co-morbidities in people with RMDs and comparators categorised by age, gender, and GP practices. Conditional logistic regression models were fitted to calculate differences (OR, 95% CI) in associations with co-morbidities between cohorts.

Results

The most prevalent co-morbidities were cardiovascular risk factors, hypertension, and diabetes. Having an RMD diagnosis was associated with a significantly higher odds for many conditions including deficiency anaemia (OR 1.39, 95% CI (1.32-1.46)), hypothyroidism (OR 1.34, 95% CI (1.19-1.50)), Pulmonary circulation disorders (OR 1.39, 95% CI 1.12 - 1.73) diabetes (OR 1.17, 95% CI (1.11-1.23)), and fluid and electrolyte disorders (OR 1.27, 95% CI (1.17-1.38)). RMDs have a higher proportion of multi-morbidity (2 or more conditions in addition to the RMD) compared to non-RMD group (81% and 73%, respectively in 2019) and the mean number of co-morbidities was higher in woman from the age of 25 and 50 in men than in non-RMDs group.

Conclusion

People with RMDs are approximately 1.5 times as likely to have multi-morbidity as the general population and provide a high-risk group for targeted intervention studies. The individuals with RMDs experience a greater load of co-existing health conditions, which tend to manifest at earlier ages. This phenomenon is particularly pronounced among women. Additionally, there is an underreporting of comorbidities in individuals with RMDs.

Key words

electronic health records, primary care, multi-morbidity, rheumatic and musculoskeletal diseases

For peer review only

1 Rheumatology key messages

What is already known about this subject?

People with RMDs are known to be living with the burden of additional chronic conditions and are at a higher risk of multi- morbidity than people without RMDs. Indeed, RMDs appear to form a principal component of certain multi-morbidity clusters.

What does this study add?

This is the first study to provide a comprehensive assessment of a range of morbidities across the life-course, including under-reported conditions, in patients with RMDs patients compared to those without RMDs using a primary-care database of electronic health records from the resident population in Wales. The mean number of co-morbidities was higher in patients with RMDs than those without and, in females, the higher mean number of co-morbidities was observed from the age of 25.

How might this impact on clinical practice or future developments in multi-morbidity?

The increased risk for multi-morbidity among RMD patients highlight where resources might be best directed to provision care, design preventative studies or allocate increased diagnostic and screening services for patients with RMDs.

2 Strengths and limitations

- To our knowledge, this is the first study to present analysis using different approaches in a large population of Wales in people with and without RMDs using routine primary care data.

- Our large study cohort provides sufficient statistical power to calculate overall and age- and sex-specific prevalence rates of individual co-morbidities between 2010 and 2019.

- In addition to the recognised and frequently reported co-morbidities such as cardiovascular risk factors, our findings highlight several co-morbidities such as deficiency anaemia, fluid and electrolyte disorders and valvular disease that are highly prevalent in people with RMDs, but which are under-reported in literature. We prioritise the early identification of RMDs and related health conditions, with a particular focus on younger women.

- We have analysed only pairs of diseases, but higher-order combinations of diseases probably interact in more complicated ways. In terms of the application of these findings, it should be noted that the prevalence measure was based on Elixhauser conditions; there is no established gold standard list of diagnoses used to define multi-morbidity and definitions of multi-morbidity vary in the medical literature.

- The origins of RMD in Wales are probably similar to those in other UK nations with comparable access to the National Health Service (NHS) and genetic links related to RMD. As a result, we expect that the risk factors and the prevalence of co-existing health conditions will exhibit similarities. However, when applying this approach to other countries, it is crucial to recognize that the specific co-morbidities may vary based on the distinct health characteristics of each nation. To enhance the applicability of our findings, validation across diverse healthcare systems and patient populations is essential.

For peer review only - http://bmjopen.bmj.com/site/about/guidelines.xhtml

3 Introduction

Multi-morbidity is a global public health concern and refers to the existence of multiple long-term conditions (MLTCs) in a single individual (1-3). Morbidity refers to individual long-term morbid conditions. The prevalence of multi-morbidity appears to have increased in many regions of the world over the past 20 years. It is anticipated to continue to rise and evidence from high-income countries suggests that, while multi-morbidity is highly prevalent in older populations (typically those over 65 years of age), it also affects younger people (4). Epidemiologically, the term co-morbidity is used when estimating co-occurring conditions among populations with an index condition and the term 'multi-morbidity' is used when estimating the prevalence of co-occurring conditions among the wider population (5-6).

Rheumatic and musculoskeletal diseases (RMDs) comprise over 200 diseases and syndromes, which can affect the joints, connective tissue, cartilage, and tendons; they are usually progressive, associated with pain and are among the most common chronic non-communicable diseases. The prevalence of disability, premature mortality and physical impairment (7-8) in RMDs is increased meaning they are a major burden on individuals, social care systems and health systems and this burden has been recognised by WHO and the United Nations (9). For example, in the UK, an estimated 8.75 million people have sought treatment for osteoarthritis, the equivalent to a third of all people over 45 years of age (10). In addition, an estimated 1 in 5 men and 1 in 2 women aged over 50 years will sustain a low-trauma fracture as a result of osteoporosis (11-13) with the situation set to worsen with ageing populations. Previous studies have found that people with RMDs are often living with additional chronic conditions (14 -16) and RMDs appear to form a principal component of certain multi-morbidity clusters (17-19). Certainly, a substantial proportion of people with RMDs now live with multi-morbidity (20) but most current clinical guidelines have 'single condition' model of care without considering multi-morbidity. Multi-morbidity has a number of negative outcomes, including disability and death, reduced quality of life, and higher healthcare utilisation and expenses (21).

Several studies have described co-morbidities in the RMDs population (22-23), but it remains to be determined if the increased co-morbidity in patients with RMDs relates to specific co-morbid conditions, occurs across the life-course or differs by sex. The longitudinal nature of the (SAIL) Databank in Wales, UK enables the analysis of information on diagnoses and consultations at multiple time points for RMDs in general (24,25).Therefore, using a large electronic linked primary care data set on the resident population in Wales, this study aimed to examine the patterns of 28 chronic conditions in people with and without RMDs over a 10-year study period. We further stratified the co-morbidities prevalence rates by age, gender, and assessed the association between diagnosis of RMDs and the presence of a co-morbidity and described the patterns of all two coexisting co-morbidities.

4 Ethics approval and consent to participate.

Approval for the use of anonymised data in this study, provisioned within the Secure Anonymised Information Linkage (SAIL) Databank, was granted by an independent Information Governance Review Panel (IGRP) under project 0911. The IGRP has a membership comprised of senior representatives from the British Medical Association (BMA), the National Research Ethics Service (NRES), Public Health Wales and Digital Health and Care Wales (DHCW) and members of the public. The SAIL Databank is compliant with General Data Protection Regulations (GDPR) and the UK Data Protection Act. SAIL provides access to de-identified data and, as such, informed consent was not applicable, as per NRES guidance. All research was performed in accordance with SAIL Databank processes and guidelines and relevant regulations.

5 Methods

5.1 Data Sources

Data used for this study were accessed via the Secure Anonymised Information Linkage (SAIL) Databank (www.saildatabank.com). SAIL is a state of the art, remotely accessible, privacy-protecting Trusted Research Environment accredited under the Digital Economy Act. SAIL contains linked anonymised population-scale data sources at an individual person-level, household-level and multiple-ecological-levels, for the population of Wales (24, 25). SAIL Databank sources include inpatient, outpatient, critical care, emergency department, mortality, birth register and demographic information for people that use healthcare services in Wales.

Morbidities were defined based on Elixhuaser categories, a well-established scheme distinguishing 30 classes of chronic diseases. HIV was not included as data is not available in SAIL due to data privacy policies. The primary care Read version 2 (CTV2) codes were used to identify Elixhauser morbidities in healthcare records.

The WMC is based on the 83% of the Welsh population covered by GP practices in Wales that contribute to the SAIL databank. A report by Cary Jones in 2022 (26) ("Supplementary material") showed how this compared to the 100% of the population. The distributions of sex and age-group are identical, and the distribution of deprivation scores are very similar. The Elixhauser coding algorithms used to establish the presence of each Elixhauser morbidity were downloaded from Supplementary of Metcalfe et al (27) and resulted in a set of 28 co-morbidities and Rheumatoid arthritis/collagen vascular

For peer review only - http://bmjopen.bmj.com/site/about/guidelines.xhtml

diseases (RMDs).

5.2 Patient and Public Involvement

None

5.3 Missing data

This analysis used age, sex and the presence or absence of co-morbidities. Age and sex were complete as they were derived from the algorithm creating the anonymised linkage fields (ALFs) from NHS registration data. The presence of co-morbidities was defined by the presence of the relevant diagnostic codes in the electronic health records. When there was no diagnostic code for a particular condition in a patient's record, it was assumed that that patient did not have that condition. This is a common approach when using data from electronic health records. As a result, there were no missing data in the data that we analysed.

5.4 Participants

Study participants were selected from the Welsh Multi-morbidity e-Cohort (WMC), which consists of 2,902,101 million people living in Wales on the 1st January 2000 with follow-up until 31st December 2019 and was created to provide accessible research ready data for the understanding of multi-morbidity (28).

5.4.1 RMDs cohort

Patients aged 0 – 100 years with a first diagnostic RMDs medical code between 1st January 2010 and 31st December 2019 were identified. The index date was defined as the earliest date of RMDs diagnosis. Patients were followed until movement out of a SAIL providing practice, death, or end of the study observation period (31st December 2019). Our sample was restricted to patients having >= 1 year follow-up for each matched case and comparator (e.g., cases and comparators in the matched cohort in 2019).

5.4.2 Non-RMD cohort

We performed a matched, retrospective cohort study within the WMC. Within each year, up to five patients without RMDs (comparators) were identified and matched to each patient with RMDs on the following variables at the RMDs diagnosis date: age (±2 years difference), sex and general practice code.

±2 year time period was used for matching to increase the comparator sample after discussion with clinicians that age groups within this interval are clinically comparable. Read codes version 2 for GP records were used to identify patient records that had a RMD diagnosis recorded. We required each comparator to be enrolled with the same GP on the index date of the accompanying patient with RMDs and assigned the same index date.

5.4.3 Outcomes

Overall, the presence of morbidities was defined as conditions recorded before and after RMDs diagnosis date (index date) and up to the end of the calendar year. We defined multi-morbidity as the presence of at least two conditions from the Elixhauser comorbidity index, excluding RMDs as one of the conditions, to avoid bias in our results towards greater multi-morbidity in the RMDs cohort.

5.5 Statistical analyses

Descriptive statistics of the differences between the cohorts are reported as the total percentage of multi- morbid persons (two or more) conditions, the percentage distribution of the total number of co-morbidities and the mean number of co-morbidities by sex and age-group. To explore the patterns of multi-morbidity, the data were analysed using four approaches:

- Temporal trends for specific Elixhauser conditions were explored using prevalence rates. Annual prevalence rates were derived for each cohort over the 10-year period and were calculated separately for each co-morbidity by dividing the number of persons diagnosed in or before the study year by the total number of persons contributing throughout the same study year. Corresponding 95% confidence intervals were calculated.
- The prevalence rates were also calculated stratified by (1) 20-year age groups, (2) sex, with RMDs and matched comparator patients in the last study year.
- The patterns of morbidity conditions were examined using pairwise associations between all co-morbidities. The percentage of patients with both co-morbidities was calculated for each pair of conditions for each cohort.
- logistic regression models were used to explore association between the presence/absence of each comorbidity and diagnosis of RMD. Conditional models, adjusted for age and sex, were fitted to estimate differences between cohorts with associations for Elixhauser co-morbidities. The coefficients were exponentiated and reported as odds ratios (ORs)

along with corresponding 95% confidence intervals (CIs). Regression models restricted to males and females were also fitted to calculate sex-specific estimates.

All analyses we conducted using Python software 3.7. We used the STROBE cohort checklist when writing our report (29)

6 Results

6.1 Patient population

Of 2,902,101 million individuals enrolled in WMC between 2000-2019, we identified 102,042 who fulfilled our eligibility criteria for people with RMDs. Patients with RMDs were matched to 1,637,632 million people without RMDs (comparators). Figure 1 shows the flow chart for identification of patients with and without RMDs registered in linked general practices.

6.2 Annual prevalence of co-morbidities

We investigated the prevalence of 28 co-morbidities overall, but results for 7 co-morbidities with less than 5 subjects have been not reported due to statistical disclosure control requirements in SAIL Databank. Statistical disclosure control rules meant that cells with less than 5 subjects cannot be reported in order to prevent the inadvertent disclosure of individuals. The guidance was developed by the UK's Office of National Statistics as part of its work on protecting privacy through design and statistical disclosure control as described in their handbook https://ukdataservice.ac.uk/app/uploads/thf_datareport_aw_web.pdf . SAIL follows the ONS disclosure rules.

The annual prevalence rates of the remaining 21 co-morbidities between 2010-2019 are presented in Figure 2. The most common co-morbidities were cardiovascular risk factors, hypertension, and diabetes without complications. Other neurological disorders (OND), increased sharply over the study period in people with RMD from 0.9% (95% CI 0.56% to 1.24%) in 2010 to 10.2% (95% CI 9.86% to 10.62%) in 2019; and from 0.7% (95% CI 0.56% to 0.83%) in 2010 to 8.6% (95% CI 8.41% to 8.73%) in 2018 in comparators. Drug abuse, peptic ulcer disease and liver diseases were the least common chronic conditions in both people with and without RMDs. The prevalence rates (and confidence intervals) of all remaining 21 co-morbidities in RMDs and comparators in 2010 and 2019 are presented in "Supplementary" Table 1.

Rheumatoid arthritis (RA), connective tissue diseases (CTD), polymyalgia rheumatica and giant cell arteritis (PMR) and juvenile idiopathic arthritis (JIA) are different type of RMDs. For all these subtypes similar to the RMD group as a whole, we found the most common co-morbidities to be cardiovascular risk factors, hypertension and diabetes without complications. The annual prevalence of Elixhauser co-morbidities for each RMDs subtype between 2010-2019 is presented in "Supplementary" Figure 1.

6.3 Number of comorbidities, age-specific, sex-specific

In 2019, Patients with RMDs had a higher proportion of multi-morbidity (2 or more conditions in addition to the RMDs) compared to the comparator cohort (81% and 73% respectively) Figure 3(a). The mean number of co-morbidities in increased with age for both groups but was higher in females with RMDs between the ages of 25-100 compared to the comparator group while in males, the mean number of co-morbidities was higher among patients with RMD only from the age of 50, Figure 3(b). The age-gender specific analysis, in relation to the specific co-morbidities for RMDs and comparators are presented in 2019, Figure 4. Depression was observed as the most prevalent condition in the age groups 16-50 years for RMDs and non-RMDs, with diabetes without complications being the second most prevalent condition in patients with RMDs in those age-groups. Other neurological disorders (OND) and cancer were the second and third most prevalent condition for non RMD patients, respectively. In 2019, the prevalence of hypertension was nearly 4% higher in females and males than comparators. The plot for other years in age-gender specific analysis is presented "Supplementary" Figure 2, 3 and 4.

6.4 Proportion of patients with two co-morbidities

The most common pairs of coexisting morbidities across both the RMD and comparator groups in 2019 were combinations of hypertension, chronic pulmonary disease, depression, diabetes, and obesity. The pattern of all two coexisting co-morbidities in people with and without RMDs in 2019 are illustrated in a Figure 5. The dark blue colour in the heatmap is determined by a higher percentage of patients with the two co-morbidities in the same plot and not between separate plots. The plot for other years is presented in "Supplementary" Figure 5.

6.5 Odds ratios (95% CI) for associations of RMDs with specific co-morbidities in annual cohorts 2010-2019

Patients with RMDs were at least 30% more likely to have: deficiency anaemia (OR 1.39, 95% CI 1.32 to 1.46);

For peer review only

hypothyroidism (OR 1.34, 95% CI 1.19 to 1.50); pulmonary circulation disorders (OR 1.39, 95% CI 1.12 to 1.73); renal disease (OR 1.35, 95% CI 1.22 to 1.50) and weight loss (OR 1.30, 95% CI 1.17 to 1.45) compared to those without RMDs. RMDs patients were also 27% more likely to have both and electrolyte disorders (OR 1.27, 95% CI 1.17 to 1.38) and valvular disease (OR 1.27, 95% CI 1.15 to 1.41), 17% more likely to have diabetes (OR 1.17, 95% CI 1.11 to 1.23), and 9% more likely to have a diagnosis of obesity (OR 1.09, 95% CI 1.05 to 1.13) when compared people without RMDs. A cancer diagnosis was 19% less likely for the RMDs group. Similarly, other neurological disorders, diabetes with complications, periheral vascular disorders, drug abuse and alcohol abuse were all significantly less likely to be diagnosed among people with RMDs compared to people without. ORs were generally stable over the study period between 2010 and 2019, with the exception of depression which was found to be significantly higher among patients with RMDs between 2010 and 2015 but was significantly (albeit slightly) lower in 2018 and 2019. The odds ratios (Ors) are presented in Table 1. The Ors for the gender-specific analyses is presented in “Supplementary” Figure 6.

For peer review only

Conditions	2010	2011	2012	2013	2014	2015	2016	2017	2018	2019
ALCOHOL ABUSE	0.73 (0.58-0.93)	0.75 (0.63-0.88)	0.71 (0.62-0.82)	0.71 (0.63-0.80)	0.68 (0.61-0.75)	0.67 (0.61-0.74)	0.67 (0.61-0.73)	0.66 (0.61-0.72)	0.68 (0.62-0.74)	0.64 (0.59-0.69)
ARRYTHMIA	1.06 (0.94-1.20)	1.04 (0.96-1.14)	1.04 (0.97-1.12)	1.09 (1.02-1.16)	1.10 (1.04-1.16)	1.10 (1.05-1.16)	1.15 (1.09-1.21)	1.15 (1.10-1.21)	1.18 (1.13-1.24)	1.13 (1.08-1.19)
CPD	1.09 (1.00-1.18)	1.14 (1.08-1.21)	1.13 (1.08-1.19)	1.13 (1.08-1.18)	1.10 (1.06-1.15)	1.08 (1.04-1.12)	1.07 (1.03-1.10)	1.07 (1.04-1.11)	1.03 (1.00-1.07)	1.02 (0.99-1.05)
CHF	0.99 (0.85-1.16)	1.00 (0.89-1.11)	1.00 (0.91-1.10)	0.99 (0.91-1.07)	1.03 (0.95-1.10)	0.99 (0.92-1.06)	0.99 (0.93-1.06)	0.99 (0.93-1.06)	0.95 (0.89-1.02)	0.91 (0.85-0.97)
DEF_ANAEMIA	1.26 (1.11-1.42)	1.33 (1.22-1.45)	1.36 (1.27-1.46)	1.36 (1.28-1.44)	1.37 (1.29-1.44)	1.39 (1.32-1.46)	1.37 (1.31-1.44)	1.32 (1.26-1.39)	1.32 (1.26-1.38)	1.30 (1.25-1.36)
DEPRESSION	1.17 (1.07-1.27)	1.13 (1.07-1.20)	1.08 (1.03-1.14)	1.07 (1.03-1.12)	1.07 (1.03-1.11)	1.03 (0.99-1.06)	1.01 (0.98-1.05)	1.00 (0.97-1.03)	0.96 (0.94-0.99)	0.95 (0.92-0.98)
DIAB	1.13 (1.02-1.26)	1.17 (1.08-1.26)	1.16 (1.09-1.23)	1.17 (1.11-1.23)	1.13 (1.08-1.19)	1.15 (1.10-1.21)	1.13 (1.09-1.18)	1.13 (1.09-1.18)	1.13 (1.09-1.18)	1.14 (1.10-1.18)
DIAB_COMP	0.89 (0.76-1.05)	0.86 (0.77-0.96)	0.87 (0.79-0.95)	0.92 (0.84-0.99)	0.94 (0.87-1.01)	0.92 (0.86-0.99)	0.92 (0.86-0.98)	0.93 (0.87-0.99)	0.88 (0.83-0.94)	0.90 (0.85-0.95)
DRUG_ABUSE	0.91 (0.66-1.25)	0.68 (0.53-0.86)	0.81 (0.67-0.98)	0.80 (0.68-0.94)	0.75 (0.65-0.87)	0.77 (0.67-0.89)	0.79 (0.69-0.90)	0.79 (0.70-0.89)	0.75 (0.67-0.85)	0.73 (0.65-0.82)
FED	1.26 (1.07-1.47)	1.28 (1.14-1.43)	1.26 (1.14-1.39)	1.27 (1.17-1.38)	1.21 (1.12-1.31)	1.21 (1.13-1.30)	1.19 (1.11-1.27)	1.22 (1.14-1.30)	1.18 (1.10-1.26)	1.16 (1.09-1.24)
HYPERTENSION	1.02 (0.94-1.12)	1.05 (0.99-1.12)	1.06 (1.01-1.12)	1.05 (1.00-1.09)	1.04 (1.00-1.08)	1.01 (0.97-1.04)	1.02 (0.99-1.06)	1.01 (0.98-1.05)	1.03 (1.00-1.06)	1.04 (1.01-1.07)
HYPOTHYROID	1.34 (1.19-1.50)	1.19 (1.10-1.29)	1.18 (1.10-1.26)	1.21 (1.14-1.29)	1.18 (1.12-1.24)	1.20 (1.14-1.26)	1.24 (1.19-1.30)	1.25 (1.20-1.31)	1.25 (1.20-1.31)	1.27 (1.22-1.32)
LIVER_DISEASE	0.83 (0.62-1.12)	1.03 (0.84-1.26)	0.97 (0.83-1.14)	1.03 (0.90-1.18)	1.10 (0.98-1.24)	1.11 (1.00-1.24)	1.13 (1.02-1.25)	1.09 (0.99-1.21)	1.01 (0.92-1.12)	1.03 (0.94-1.14)
OBESITY	1.11 (1.00-1.23)	1.08 (1.00-1.16)	1.02 (0.96-1.08)	1.06 (1.01-1.12)	1.08 (1.04-1.13)	1.08 (1.04-1.13)	1.08 (1.04-1.12)	1.08 (1.04-1.12)	1.09 (1.05-1.13)	1.07 (1.03-1.11)
OND	0.91 (0.82-1.01)	0.89 (0.83-0.95)	0.94 (0.89-1.00)	0.91 (0.87-0.96)	0.92 (0.88-0.96)	0.90 (0.86-0.94)	0.91 (0.87-0.95)	0.89 (0.86-0.93)	0.87 (0.84-0.91)	0.84 (0.81-0.88)
PEP_ULCER	1.02 (0.84-1.23)	1.04 (0.91-1.19)	1.06 (0.95-1.19)	1.04 (0.95-1.15)	1.03 (0.94-1.12)	1.05 (0.97-1.14)	1.07 (0.99-1.16)	1.05 (0.97-1.13)	1.00 (0.93-1.07)	0.98 (0.91-1.05)
PVD	0.88 (0.73-1.05)	0.92 (0.82-1.05)	0.93 (0.84-1.03)	0.99 (0.90-1.08)	0.97 (0.89-1.06)	0.98 (0.91-1.06)	0.93 (0.86-1.00)	0.95 (0.88-1.02)	0.89 (0.82-0.95)	0.88 (0.82-0.94)
PCD	1.39 (1.12-1.73)	1.33 (1.15-1.55)	1.30 (1.14-1.47)	1.24 (1.11-1.38)	1.25 (1.13-1.38)	1.25 (1.13-1.37)	1.24 (1.14-1.36)	1.25 (1.15-1.37)	1.11 (1.02-1.21)	1.15 (1.06-1.25)
RENAL failure	1.35 (1.22-1.50)	1.31 (1.22-1.41)	1.28 (1.20-1.36)	1.24 (1.17-1.31)	1.21 (1.15-1.27)	1.22 (1.16-1.28)	1.16 (1.11-1.22)	1.13 (1.08-1.18)	1.14 (1.09-1.19)	1.13 (1.09-1.18)
CANCER	0.92 (0.84-1.02)	0.93 (0.87-0.99)	0.90 (0.85-0.95)	0.90 (0.85-0.94)	0.89 (0.85-0.93)	0.89 (0.85-0.92)	0.87 (0.84-0.90)	0.85 (0.82-0.89)	0.84 (0.81-0.87)	0.81 (0.78-0.84)
VALVULAR _DISEASE	1.16 (1.00-1.35)	1.27 (1.15-1.41)	1.20 (1.10-1.31)	1.26 (1.17-1.36)	1.24 (1.16-1.33)	1.22 (1.15-1.31)	1.17 (1.10-1.25)	1.17 (1.10-1.24)	1.15 (1.08-1.22)	1.13 (1.07-1.20)
WEIGHT_LOSS	1.20 (1.03-1.39)	1.30 (1.17-1.45)	1.26 (1.16-1.38)	1.30 (1.20-1.40)	1.24 (1.16-1.33)	1.22 (1.14-1.30)	1.20 (1.13-1.28)	1.26 (1.19-1.34)	1.20 (1.13-1.27)	1.16 (1.09-1.23)

For peer review only - http://bmjopen.bmj.com/site/about/guidelines.xhtml

Table 1. Odds ratios (95% CI) for associations of RMDs with specific co-morbidities in annual cohorts 2010-2019. Alcohol abuse, Blood loss anemia, Cardiac arrhythmia, Chronic pulmonary disease (CPD), Congestive heart failure (CHF), Deficiency anemia, Depression, Diabetes complicated, Diabetes, Drug abuse, FLuid and electrolyte disorder (FED), Hypertension, Hypothyroidism, Liver disease, Other neurological disorders (OND), Paralysis, Peptic ulcer disease excluding bleeding (PEPTIC_ULCER), Periheral vascular disorders (PVD), Psychoses, Pulmonary circulation disorders (PCD), Renal failure, Cancer, Valvular disease, Weight loss

9/15

For peer review only

7 Discussion

To our knowledge, this is the first study to examine and compare the patterns of 28 Elixhauser-defined co-morbidities using different statistical approaches in a large, well-characterized population of people with RMDs and people without RMDs in Wales. The primary care database that we used to estimate the prevalence of multimorbidity covered over 83% of the population in the Wales so the findings should be generalisable in that population.

Our findings show that co-morbidities were more prevalent in people with RMDs than comparators throughout the 10-year study period. Overall, co-morbidities were more prevalent in women than in men and the prevalence rates increased with age. Our results demonstrate people with RMDs are approximately 1.5 as likely to have multimorbidity as the general population throughout the 10-year study period. Diabetes and hypertension are the epicenter of RMD disease and potentially part of the trajectories of several other chronic conditions, suggesting a need for a more integrative multidisciplinary approach focusing on better management and prevention of these conditions (2).

An Australian systematic review of population-based studies in the elderly (15) has previously reported that over 50% of elderly patients with arthritis also had hypertension, followed by cardiovascular diseases, dyslipidemia, diabetes, and mental health problems, in keeping with our findings. Our study was not restricted to elderly patients but shows that the mean number of co-morbidities is also higher in females at younger ages (25-100 years) whilst in males, the increased mean number of co-morbidities was observed from the age of 50.

Patients with RA have also been reported to have a greater prevalence of diabetes mellitus and hypertension, two key risk factors for chronic kidney disease (CKD), possibly arising from the use of corticosteroids to manage RA (30). Some studies show that weight loss is a strong predictor of death in patients with RA (31- 33) so it is important to recognise that weight loss is more highly prevalent across all RMDs from our study.

For patients with axial spondyloarthritis (axSpA) identified from health insurance data in Germany, the most prevalent comorbid Elixhuaser conditions were ‘hypertension’ followed by ’depression’ and ’chronic pulmonary’ disease (34), illustrating that these co-morbid conditions are common regardless of the specific RMD. Based on the findings, it will be important for international guideline committees to assess co-morbidities, rather than specific co-morbidities.

The European League Against Rheumatism (EULAR) guidelines provide specific recommendations for the management of several co-morbidities in individuals with RMDs, including cardiovascular disease, osteoporosis, depression, and anxiety (35). For example, the guidelines recommend that individuals with RMDs and cardiovascular disease should be treated with statins and antiplatelet agents; (35) Individuals with RMDs and osteoporosis should be treated with bisphosphonates and that depression or anxiety should be treated with cognitive behavioural therapy or antidepressants (35). However, other co-morbidities are not considered specifically. The implications of our findings are that holistic assessment of all co-morbidities is required and our results suggest this is applicable to RMDs as a whole.

Strengths and limitations

Our study has several strengths: first, to our knowledge, this is the first study to present analysis using different approaches in the large population of Wales in people with and without RMDs using routine primary care data so avoiding biased sampling strategies. Furthermore, the study design minimised bias by matching groups on relevant confounders. Second, our large cohort provides sufficient statistical power to calculate overall and age- and sex-specific prevalence rates of individual co-morbidities between 2010 and 2019. Finally, in addition to the recognised and frequently reported co-morbidities such as cardiovascular risk factors, our findings highlight a number of co-morbidities such as deficiency anaemia, fluid and electrolyte disorders and valvular disease that are highly prevalent in people with RMDs but which are under-reported in literature.

The study replicates and confirms the finding from previous studies, for example Ziyade et al (22), in a 769 Lebanese population, who report the most frequent co-morbidities for patients with RMDs were cardiovascular risk factors (hypertension, and diabetes), hypercholesterolemia, depression. Consistent with our findings, all reports listed hypertension as the most common individual comorbidity, but we also add renal disease, weight loss, fluid and electrolyte disorders and valvular disease as new pair of co-morbidities, that could be tested directly in other populations with data available. It’s essential to recognize that our focus is on investigating patterns of multi-morbidity rather than establishing causal relationships.

Our study is a first step towards service transformation for multi-morbid RMD patients. It does not provide directly actionable

recommendations but can be used as a starting point to consider creating dedicated pathways for patients with co-morbidities that commonly co-occur with RMD, similar to EULAR guidelines.

The aetiology of RMD in Wales is likely comparable to other UK nations with similar access to the National Health Service (NHS) and genetic associations related to RMD. Consequently, we anticipate that the risk factors and the pattern of co-existing health conditions will be similar. However, when applying this method to other countries, it's essential to consider that the specific co-morbidities may differ based on the unique health characteristics of each country. To enhance the generalisability, the findings should be validated in diverse healthcare systems and patient populations.

The findings could inform policies around resource allocation, healthcare planning, and the development of integrated care models for individuals with RMDs and co-morbidities; whilst that is beyond the scope of the current work, our findings will provide valuable information from a large population, using robust and established methods to highlight that patients with RMDs have a higher burden of co-morbidities that start to occur at younger ages, particularly in women.

The SAIL databank holds records from administrative sources within the NHS; efforts to link non-health data such as socioeconomic status, education and lifestyle factors is underway, but it is likely to be some years before such linkage is available. This a limitation of the current analysis, as well as a focus for future work.

A further limitation is that we have analysed only pairs of diseases, but higher-order combinations of diseases probably interact in more complicated ways. While pairwise comparison is a simple and easy-to-understand method, advanced statistical methods such as network analysis, (36) and clustering algorithms can provide a more nuanced understanding of comorbidities by considering the interactions between multiple diseases and could be applied in future work.

Further research is required to explore potential variation within subpopulations, such as differences among rural and urban areas and we like to note that our point about generalisability of these findings with regards to ethnic background are based on the fact that the population of Wales are majorly white (above 80%) (37). In terms of the application of these findings, it should be noted that the prevalence measure was based on Elixhauser conditions; there is no established gold standard list of diagnoses used to define multi-morbidity and definitions of multi- morbidity vary in the medical literature (38), making direct comparisons across studies and different populations more challenging. However, separately both the Charlson and Elixhauser indices are often used (39) and the Cambridge multi-morbidity score has been published recently (38) and there is some overlap between these indices in defining co-morbidity.

For peer review only

Clinical implications

We emphasise early detection of RMDs and associated conditions, especially in younger women. Healthcare practitioners should facilitate patient access to community resources like exercise programs and support groups to enhance overall well-being and address depression in the age range of 16 to 50 years based on our study findings. We also introduce focused screening initiatives for prevalent comorbidities linked to RMDs, including renal disease, weight loss, and fluid and electrolyte disorder and valvular disease.

Given the prevalence of RMDs in the population, the finding that patients are at significantly higher risk of a number of co-morbidities is an important finding and could inform targeted screening for primary/secondary prevention in clinical settings. This is particularly relevant for the common co-morbidities such as hypertension, where multiple treatments are available and screening at health care visits in this high-risk population would provide an opportunity to introduce prevention measures for subsequent conditions such as cardiovascular disease and stroke. The finding that risks are increased from a younger age in RMD patients and in women implies that screening should be extended to the whole RMD population rather than the general population screening, which focusses on older age groups.

Conclusion

In conclusion, we provide the first detailed 10-year epidemiological analysis of multi-morbidity burden in people with RMDs in comparison with individuals without RMDs using Welsh primary care data. We found a substantially higher prevalence and burden of multi-morbidity in patients with RMDs relative to those without, suggesting that RMDs patients represent a high-risk group for multi-morbidity. A greater understanding of multi-morbidity could aid clinicians in developing and delivering comprehensive treatment pathways and to action primary and secondary prevention of co-morbidities in people with RMDs. This higher mean number of co-morbidities is seen from the age of 25 in women and 50 in men. Further research is required to determine whether targeted interventions could reduce overall disease burden from multi-morbidity in that group. This study depends on diagnoses reported in electronic health records, it is possible that patients with undiagnosed conditions, such as those who did not visit a general practitioner, were missed in our study.

1 abbreviations

The Table 2 in Supplementary describes the significance of various abbreviations and acronyms used throughout of this paper.

Figures Caption

Figure 1. Flow chart for identification of patients with and without RMDs registered in WMC, using year 2019 as an example.

Welsh multi-morbidity e-Cohort (WMC); Rheumatic and musculoskeletal diseases (RMDs)

Figure 2. Annual prevalence of Elixhauser co-morbidities in RMDs cases and matched comparators 2010-2019

Figure 3. Trends of co-morbidities in patients with RMDs and matched comparators.

(a) The number of conditions in patients with RMDs and matched comparators without RMDs in 2010-2019,

(b) Mean number of comorbidities in patients with RMDs and comparators by age categories and sex 2019

Figure 4. age-gender specific analysis, in relation to specific Elixhauser co-morbidities in 2019

(a) Age specific analysis in patients with RMDs (b) Age specific analysis in comparators

(c) Gender specific analysis in patients with RMDs (d) Gender specific analysis in comparators

Figure 5. Proportion of patients in relation to specific Elixhauser co-morbidities in 2019

(a) RMDs

(b) Comparators

For peer review only

References

1. Wu, S.-Y. & Green, A. Projection of chronic illness prevalence and cost inflation. *Santa Monica, CA: RAND Heal.* **18** (2000).
2. Buttorff, C., Ruder, T. & Bauman, M. *Multiple chronic conditions in the United States*, vol. 10 (Rand Santa Monica, CA, 2017).
3. Whitty, C. J. *et al.* Rising to the challenge of multimorbidity (2020)
4. Barnett, K. *et al.* Epidemiology of multimorbidity and implications for health care, research, and medical education: a cross-sectional study. *The Lancet* **380**, 37–43 (2012).
5. Haregu, T. N., Oldenburg, B. F., Setswe, G. & Elliott, J. Perspectives, constructs and methods in the measurement of multimorbidity and comorbidity: a critical review. *The Internet J. Epidemiol.* **10**, 1–11 (2012).
6. Zghebi, S. S., Steinke, D. T., Rutter, M. K. & Ashcroft, D. M. Eleven-year multimorbidity burden among 637 255 people with and without type 2 diabetes: a population-based study using primary care and linked hospitalisation data. *BMJ open* **10**, e033866 (2020).
7. Palazzo, C., Ravaud, J.-F., Papelard, A., Ravaud, P. & Poiraudeau, S. The burden of musculoskeletal conditions. *PloS one* **9**, e90633 (2014).
8. Sparks, J. A. *et al.* Rheumatoid arthritis and mortality among women during 36 years of prospective follow-up: results from the nurses' health study. *Arthritis care & research* **68**, 753–762 (2016).
9. Woolf, A. D. & Pfleger, B. Burden of major musculoskeletal conditions. Bull. world health organization 81, 646–656 (2003)
10. Ellis, B. M. & Conaghan, P. G. Reducing arthritis pain through physical activity: a new public health, tiered approach (2017).
11. Melton III, L. & EA, C. Cooper c. *Lane AW. Riggs BL. Perspective. How many women have osteoporosis* 5–1 (1992).
12. Kanis, J. *et al.* Long-term risk of osteoporotic fracture in malmö. *Osteoporos. international* **11**, 669–674 (2000).
13. Melton III, L. J., Atkinson, E. J., O'Connor, M. K., O'Fallon, W. M. & Riggs, B. L. Bone density and fracture risk in men. *J. Bone Miner. Res.* **13**, 1915–1923 (1998).
14. Taylor, A. W. *et al.* Multimorbidity-not just an older person's issue. results from an australian biomedical study. *BMC public health* **10**, 1–10 (2010).
15. Caughey, G. E., Vitry, A. I., Gilbert, A. L. & Roughead, E. E. Prevalence of comorbidity of chronic diseases in australia. *BMC public health* **8**, 1–13 (2008).
16. Vogeli, C. *et al.* Multiple chronic conditions: prevalence, health consequences, and implications for quality, care management, and costs. *J. general internal medicine* **22**, 391–395 (2007).
17. Prados-Torres, A., Calderón-Larrañaga, A., Hancco-Saavedra, J., Poblador-Plou, B. & van den Akker, M. Multimorbidity patterns: a systematic review. *J. clinical epidemiology* **67**, 254–266 (2014).
18. Diederichs, C., Berger, K. & Bartels, D. B. The measurement of multiple chronic diseases—a systematic review on existing multimorbidity indices. *Journals Gerontol. Ser. A: Biomed. Sci. Med. Sci.* **66**, 301–311 (2011).
19. Violan, C. *et al.* Prevalence, determinants and patterns of multimorbidity in primary care: a systematic review of observational studies. *PloS one* **9**, e102149 (2014).
20. Lowe, D. B., Taylor, M. J. & Hill, S. J. Cross-sectional examination of musculoskeletal conditions and multimorbidity: influence of different thresholds and definitions on prevalence and association estimates. *BMC research notes* **10**, 1–13 (2017).
21. Salive, M. E. Multimorbidity in older adults. *Epidemiol. reviews* **35**, 75–83 (2013).
22. Ziade, N. *et al.* Prevalence and pattern of comorbidities in chronic rheumatic and musculoskeletal diseases: The comord study. *Sci. reports* **10**, 1–10 (2020).
23. England, B. R. *et al.* Burden and trajectory of multimorbidity in rheumatoid arthritis: a matched cohort study from 2006 to 2015. *Annals rheumatic diseases* **80**, 286–292 (2021).
24. Lyons, R. A. *et al.* The sail databank: linking multiple health and social care datasets. *BMC medical informatics decision making* **9**, 3 (2009).
25. Ford, D. V. *et al.* The sail databank: building a national architecture for e-health research and evaluation. *BMC health services research* **9**, 157 (2009).

For peer review only

26. Jones, C. Gp data - full report (2022-03-30).

27. Metcalfe, D. *et al.* Coding algorithms for defining charlson and elixhauser co-morbidities in read-coded databases. *BMC medical research methodology* **19**, 1–9 (2019).

28. Lyons, J. *et al.* Protocol for the development of the wales multimorbidity e-cohort (wmc): data sources and methods to construct a population-based research platform to investigate multimorbidity. *BMJ open* **11**, e047101 (2021).

29. Von Elm, E. *et al.* The strengthening the reporting of observational studies in epidemiology (strobe) statement: guidelines for reporting observational studies. *The Lancet* **370**, 1453–1457 (2007).

30. Adcock, I. M. & Mumby, S. Glucocorticoids. *Pharmacol. Ther. Asthma COPD* 171–196 (2016).

31. Baker, J. F. *et al.* Weight loss, the obesity paradox, and the risk of death in rheumatoid arthritis. *Arthritis & Rheumatol.* **67**, 1711–1717 (2015).

32. England, B. R. *et al.* Body mass index, weight loss, and cause-specific mortality in rheumatoid arthritis. *Arthritis care & research* **70**, 11–18 (2018).

33. Willadsen, T. G. *et al.* The role of diseases, risk factors and symptoms in the definition of multimorbidity–a systematic review. *Scand. journal primary health care* **34**, 112–121 (2016).

34. Redeker, I. *et al.* The prevalence and impact of comorbidities on patients with axial spondyloarthritis: results from a nationwide population-based study. *Arthritis research & therapy* **22**, 1–11 (2020).

35. Damarell, R. A., Morgan, D. D. & Tieman, J. J. General practitioner strategies for managing patients with multimorbidity: a systematic review and thematic synthesis of qualitative research. *BMC Fam. Pract.* **21**, 1–23 (2020).

36. Rafferty, J. *et al.* Ranking sets of morbidities using hypergraph centrality. *J. Biomed. Informatics* **122**, 103916 (2021).

37. Akbari, A. *et al.* Developing a research ready population-scale linked data ethnicity-spine in wales. *medRxiv* 2022–11 (2022).

38. Payne, R. A. *et al.* Development and validation of the cambridge multimorbidity score. *Cmaj* **192**, E107–E114 (2020).

39. Johnston, M. C., Crilly, M., Black, C., Prescott, G. J. & Mercer, S. W. Defining and measuring multimorbidity: a systematic review of systematic reviews. *Eur. J. Public Heal.* **29**, 182–189 (2019).

Author's contributions

Farideh Jalali-najafabadi: Methodology, Software, Formal analysis, Writing original draft. Rowena Bailey: Formal analysis, Writing – review & editing. Jane Lyons: Data Curation, Writing – review & editing. Ashley Akbari: Writing – review & editing. Thamer Ba Dhafari: Writing – review & editing. Narges Azadbakht: Writing – review & editing. James Rafferty: Writing – review & editing. Alan Watkins: Writing – review & editing. Glen P. Martin: Writing – review & editing. John Bowes: Supervision, Writing – review & editing. Ronan A. Lyons: Project administration, Writing – review & editing. Anne Barton: Supervision, Writing – review & editing. Niels Peek: Project administration, Supervision, Writing – review & editing.

Acknowledgements

FJ is supported by an MRC/University of Manchester Skills Development Fellowship. This study was supported by the NIHR Manchester BRC. The views expressed are those of the authors and not necessarily of the NHS, the National Institute for Health Research or the Department of Health. We would also like to acknowledge all data providers who make anonymised data available for research. All research conducted has been completed under the permission and approval of the SAIL independent Information Governance Review Panel (IGRP) project number 0911. This work uses data provided by patients and collected by the NHS as part of their care and support. We would like to acknowledge all data providers who make anonymised data available for research. We also acknowledge help and advice from Dr.Salwa S Zghebi from school of health science Manchester University, UK.

Competing interests

The creation of the Wales Multi-morbidity Cohort was co-funded by MRC and NIHR through grant number MR/S027750/1. Anne Barton has received grant funding and/or consultancy/speaker fees from Bristol Myers Squibb, Pfizer, Galapagos, Scipher Medicine and Roche-Chugai but not in relation to the current work. FJ, RB, JL, AA, TB, NA, JR, AW, GM, JB, RL, AB and NP do not have any competing interests.

For peer review only

Availability of data and materials

The data used in this study are available in the SAIL Databank at Swansea University, Swansea, UK, but as restrictions apply they are not publicly available. All proposals to use SAIL data are subject to review by an independent Information Governance Review Panel (IGRP). Before any data can be accessed, approval must be given by the IGRP. The IGRP gives careful consideration to each project to ensure proper and appropriate use of SAIL data. When access has been granted, it is gained through a privacy protecting safe haven and remote access system referred to as the SAIL Gateway. SAIL has established an application process to be followed by anyone who would like to access data via SAIL at https://www.saildatabank.com/application-process

Funding

The datasets used in this study were supported by grants co-funded by Medical Research Council (MRC) and NIHR (grant number:MR/S027750/1); and supported by Health Data Research UK (grant number: HDR-9006), which receives its funding from the UK Medical Research Council, Engineering and Physical Sciences Research Council, Economic and Social Research Council, Department of Health and Social Care (England), Chief Scientist Office of the Scottish Government Health and Social Care Directorates, Health and Social Care Research and Development Division (Welsh Government), Public Health Agency (Northern Ireland), British Heart Foundation (BHF) and the Wellcome Trust. This work was supported by the ADR Wales programme of work. The ADR Wales programme of work is aligned to the priority themes as identified in the Welsh Government's national strategy: Prosperity for All. ADR Wales brings together data science experts at Swansea University Medical School, staff from the Wales Institute of Social and Economic Research, Data and Methods (WISERD) at Cardiff University and specialist teams within the Welsh Government to develop new evidence which supports Prosperity for All by using the SAIL Databank at Swansea University, to link and analyse anonymised data. ADR Wales is part of the Economic and Social Research Council (part of UK Research and Innovation) funded ADR UK (grant ES/S007393/1). This research was partially funded by the National Institute for Health Research (NIHR)'s Manchester Biomedical Research Centre. The views expressed are those of the author(s) and not necessarily those of the NHS, the National Institute for Health research or the Department of Health and Social Care. The work is supported by the Centre for Genetics and Genomics Versus Arthritis (UK grant number 21754). AB is an NIHR Senior Investigator. Farideh Jalali-najafabadi's research is supported by an MRC/University of Manchester Skills Development Fellowship (grant number MR/R016615).

For peer review only

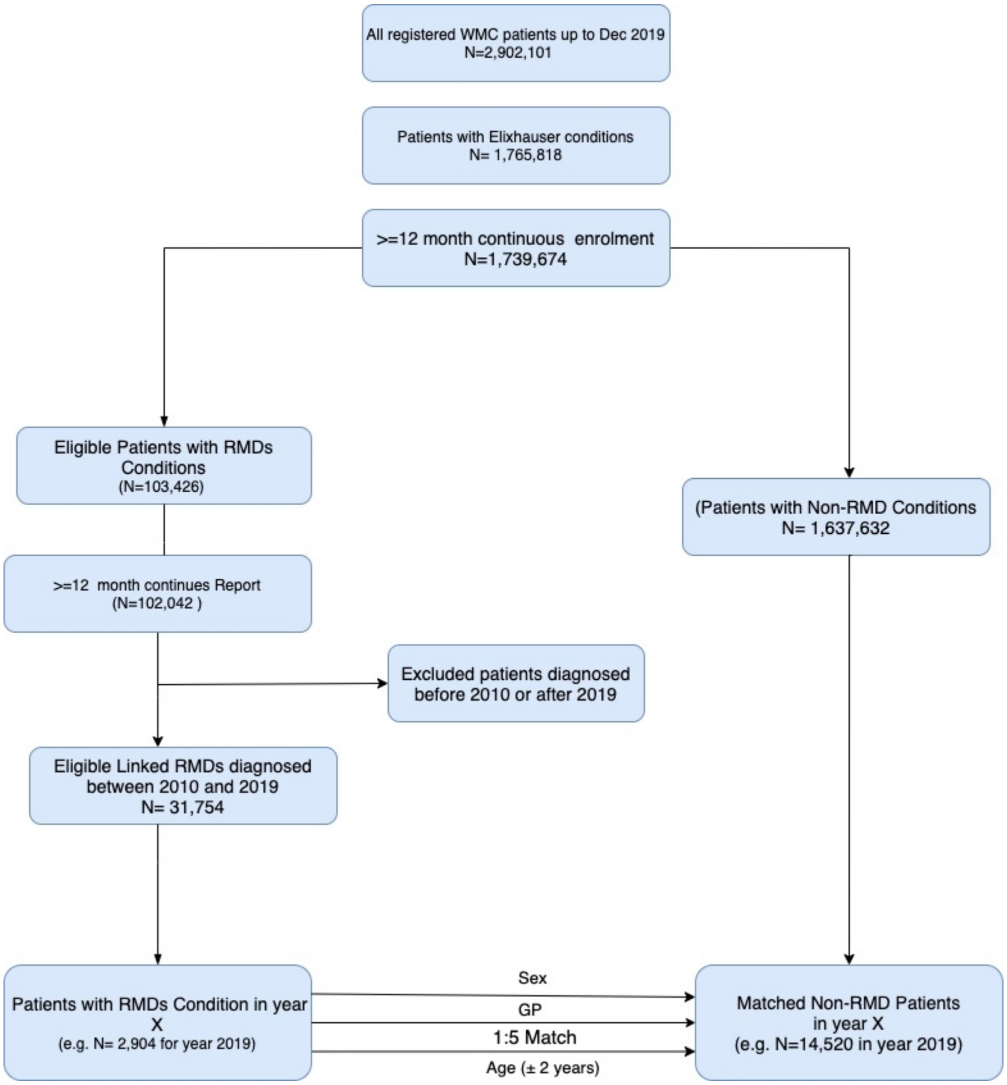

Figure 1. Flow chart for identification of patients with and without RMDs registered in WMC, using year 2019 as an example.
Welsh multi-morbidity e-Cohort (WMC); Rheumatic and musculoskeletal diseases (RMDs)

66x71mm (300 x 300 DPI)

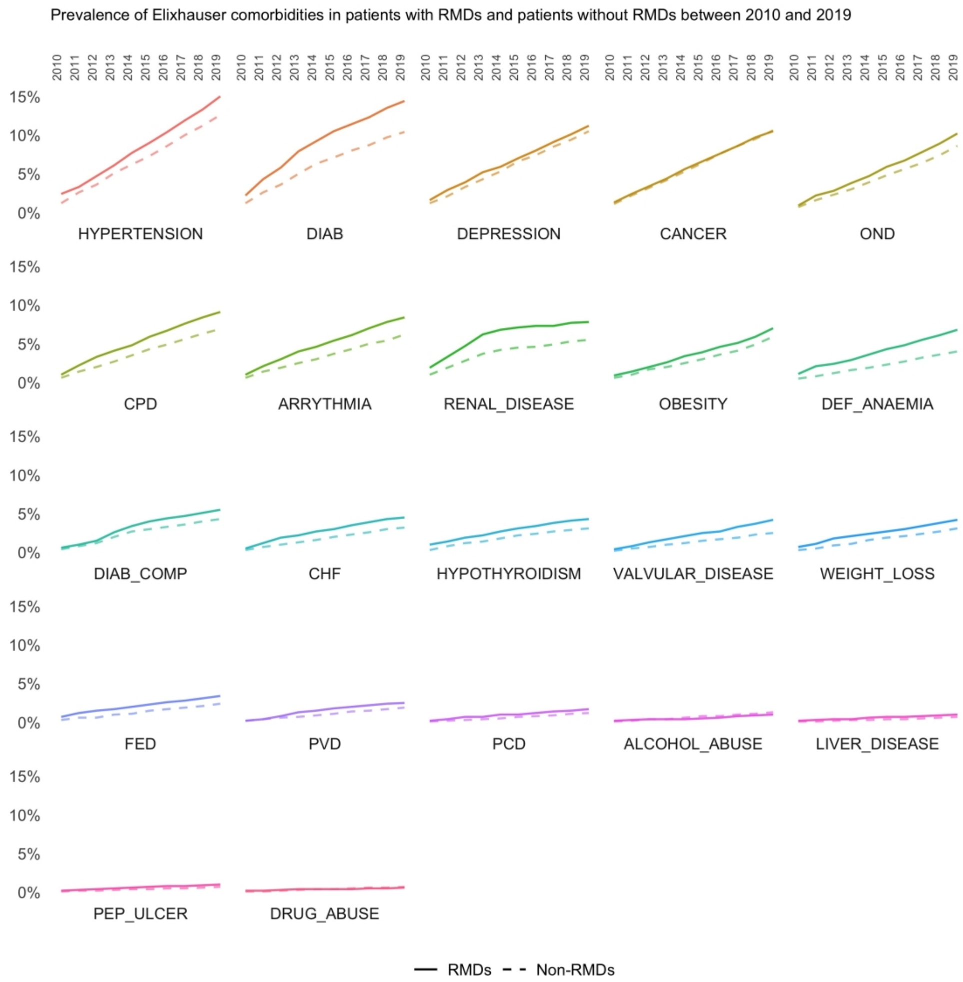

Figure 2. Annual prevalence of Elixhauser co-morbidities in RMDs cases and matched comparators 2010-2019

236x236mm (300 x 300 DPI)

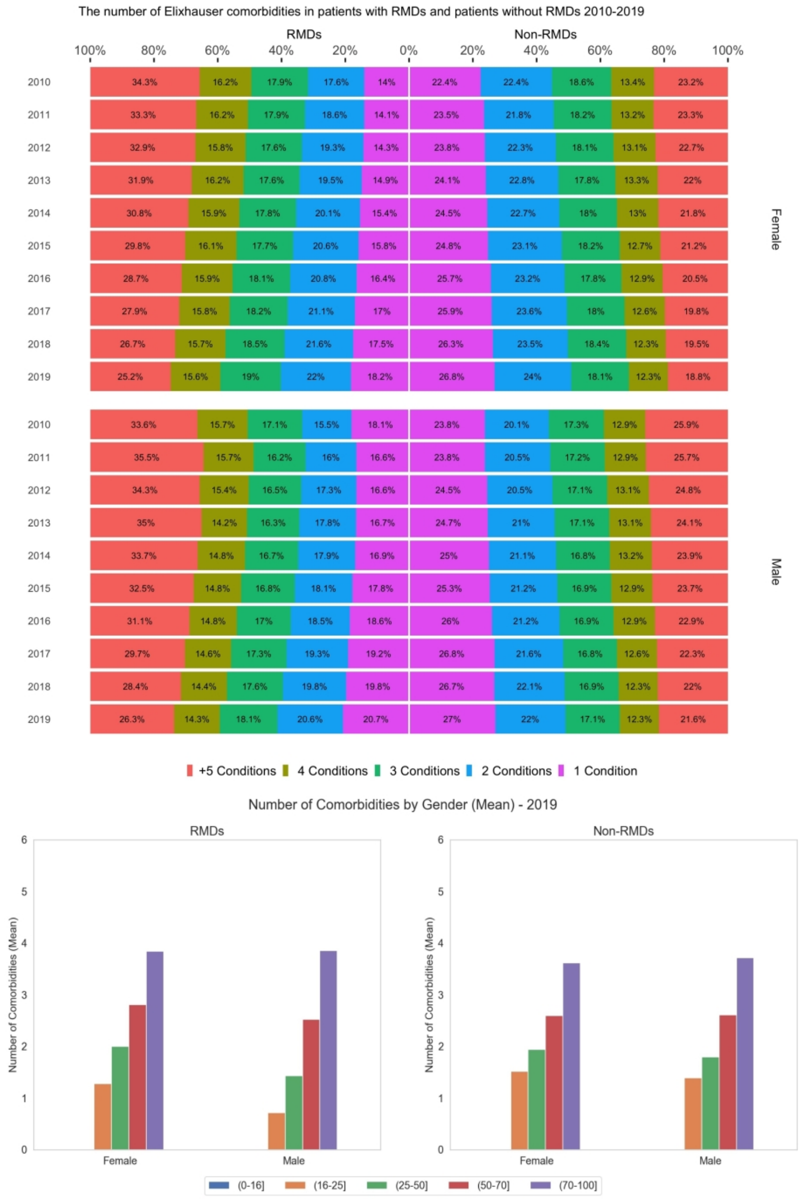

Figure 3. Trends of co-morbidities in patients with RMDs and matched comparators.
(a) The number of conditions in patients with RMDs and matched comparators without RMDs in 2010-2019,
(b) Mean number of comorbidities in patients with RMDs and comparators by age categories and sex 2019

236x357mm (300 x 300 DPI)

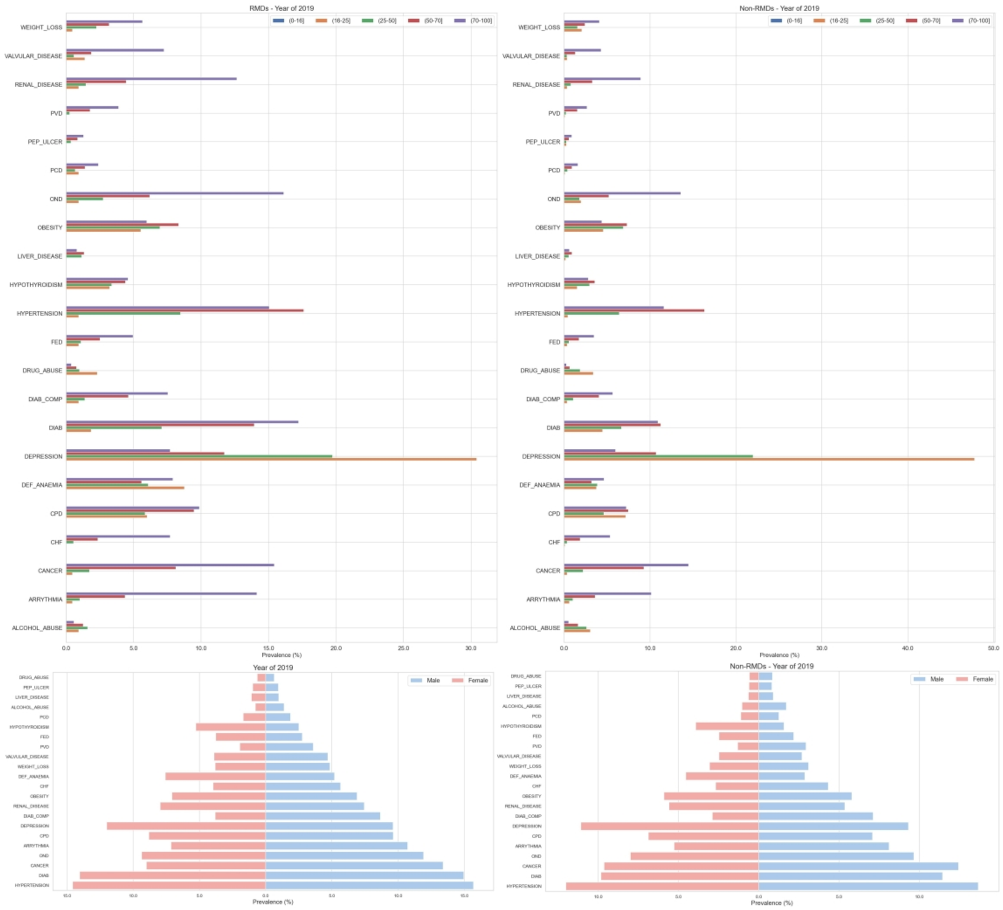

Figure 4. age-gender specific analysis, in relation to specific Elixhauser co-morbidities in 2019
(a) Age specific analysis in patients with RMDs (b) Age specific analysis in comparators
(c) Gender specific analysis in patients with RMDs (d) Gender specific analysis in comparators

456x414mm (300 x 300 DPI)

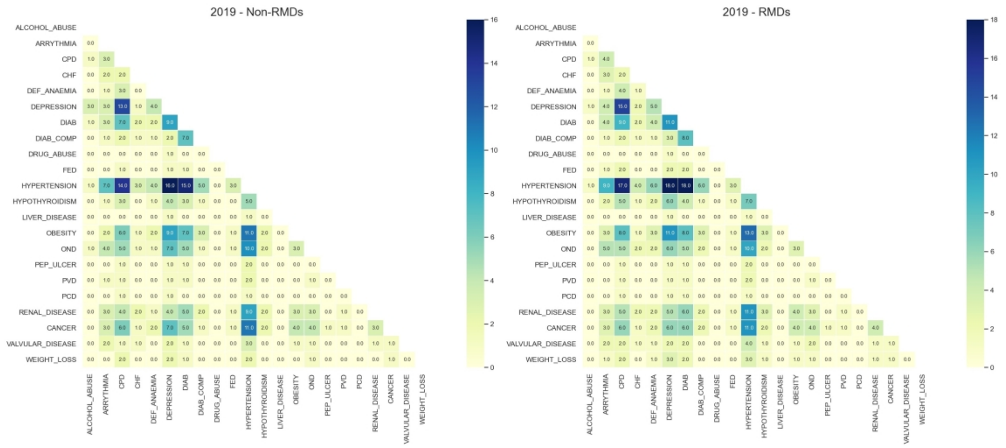

Figure 5. Proportion of patients in relation to specific Elixhauser co-morbidities in 2019
(a) RMDs
(b) Comparators

275x122mm (300 x 300 DPI)

GP Data - Full Report

Carys Jones

Date Generated: 2022-03-30

Date Range Applied: 2021-01-01 - 2021-12-31

Tables Used:

- SAILWLGPV.GP_EVENT_CLEANSED_20220101
- SAILWDSDV.AR_PERS_20220131
- SAILREFRV.WELSH_GP_CLUSTER_PRACTICES_20220129
- SAIL0286V.WLGP_CLEAN_GP_REG_BY_PRAC_INCLNONSAIL_MEDIAN_20220201
- SAILX0675V.WDSD_CLEAN_ADD_GEOG_CHAR_LSOA2011_20220328
- SAILREFRV.WALES_GP_PRACTICES_MAIN_SITES_CLUSTERS_20210407

This report describes the General Practice data in SAIL in two ways:

a. the proportion of the Welsh population with a General Practice record in SAIL
b. the proportion of General Practices in Wales that are contributing data to SAIL

Summary Plots

Category	Total Population in SAIL (Percent)
**Complete**	
•	83.6
**Age Group**	
0 to 17	83.9
18 to 64	83.9
65 plus	82.5
**Sex**	
Female	83.7
Male	83.5
**WIMD 2019 Quintile**	
1 (Most Deprived)	87.8
2	86.4
3	81.2
4	78.7
5	85.7
NULL	80.7

Category	Breakdown of SAIL Patients (Percent)	Breakdown of Total Patients (Percent)
**Complete**		
•	100.0	100.0
**Age Group**		
0 to 17	18.8	18.8
18 to 64	60.1	59.9
65 plus	21.0	21.3
**Sex**		
Female	50.1	50.1
Male	49.9	49.9
**WIMD 2019 Quintile**		
1 (Most Deprived)	20.2	19.3
2	19.4	18.8
3	18.3	18.9
4	17.2	18.4
5	19.0	18.5
NULL	5.9	6.1

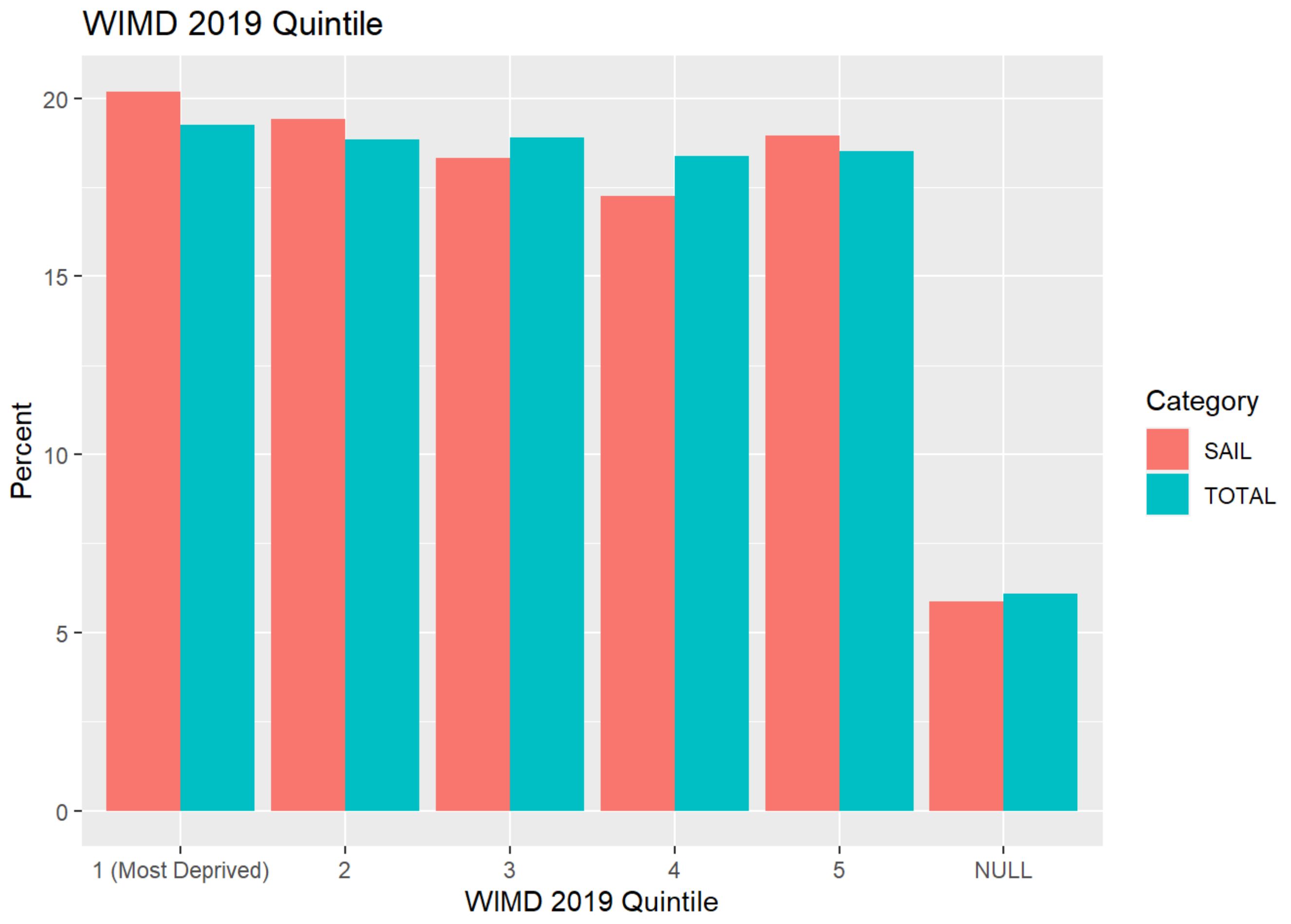
WIMD 2019 Quintile
20
15
10
5
0
Percent
1 (Most Deprived)
2
3
4
5
NULL
WIMD 2019 Quintile
Category
SAIL
TOTAL

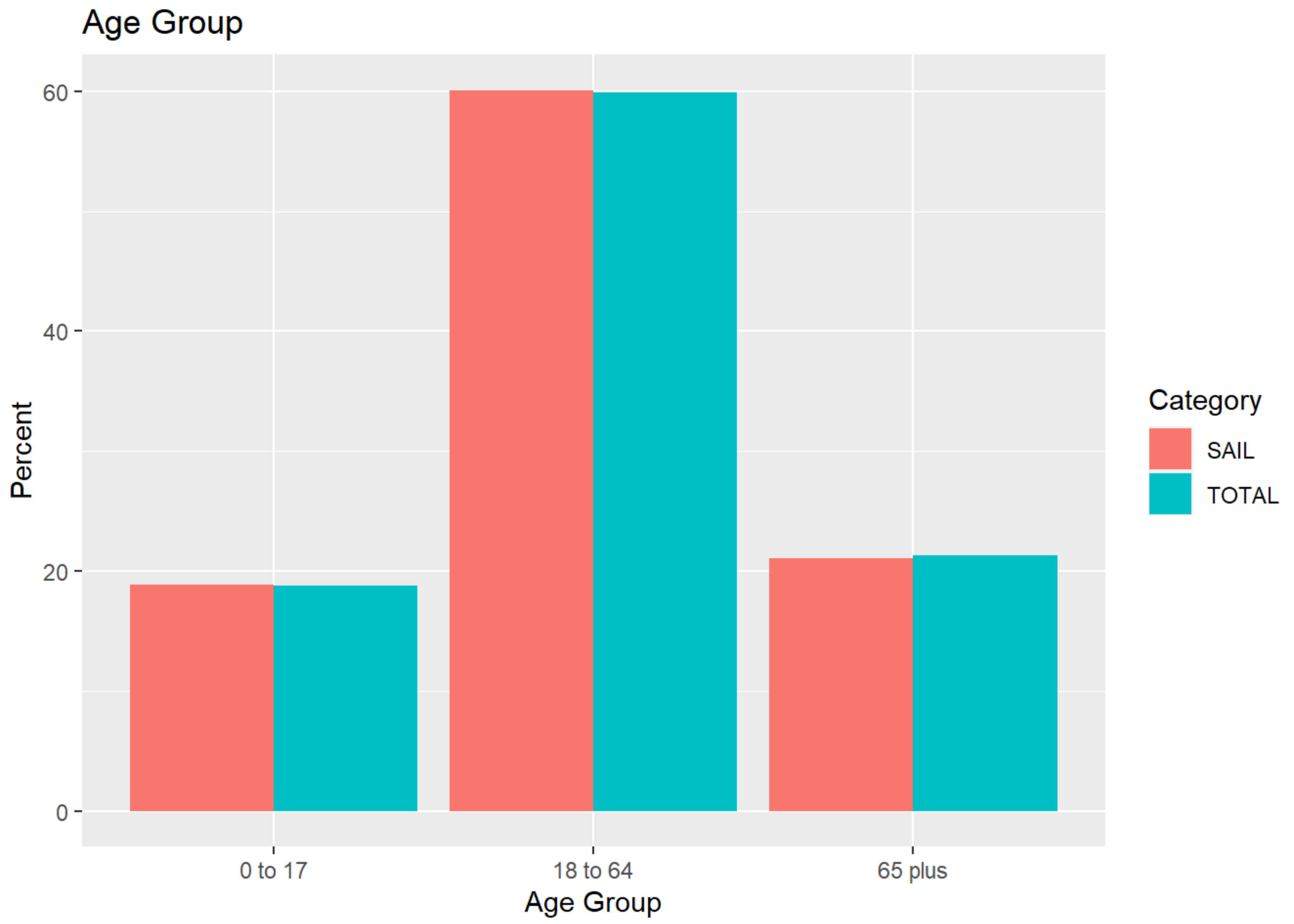
Age Group
60
40
20
0
Percent
0 to 17
18 to 64
65 plus
Age Group
Category
SAIL
TOTAL

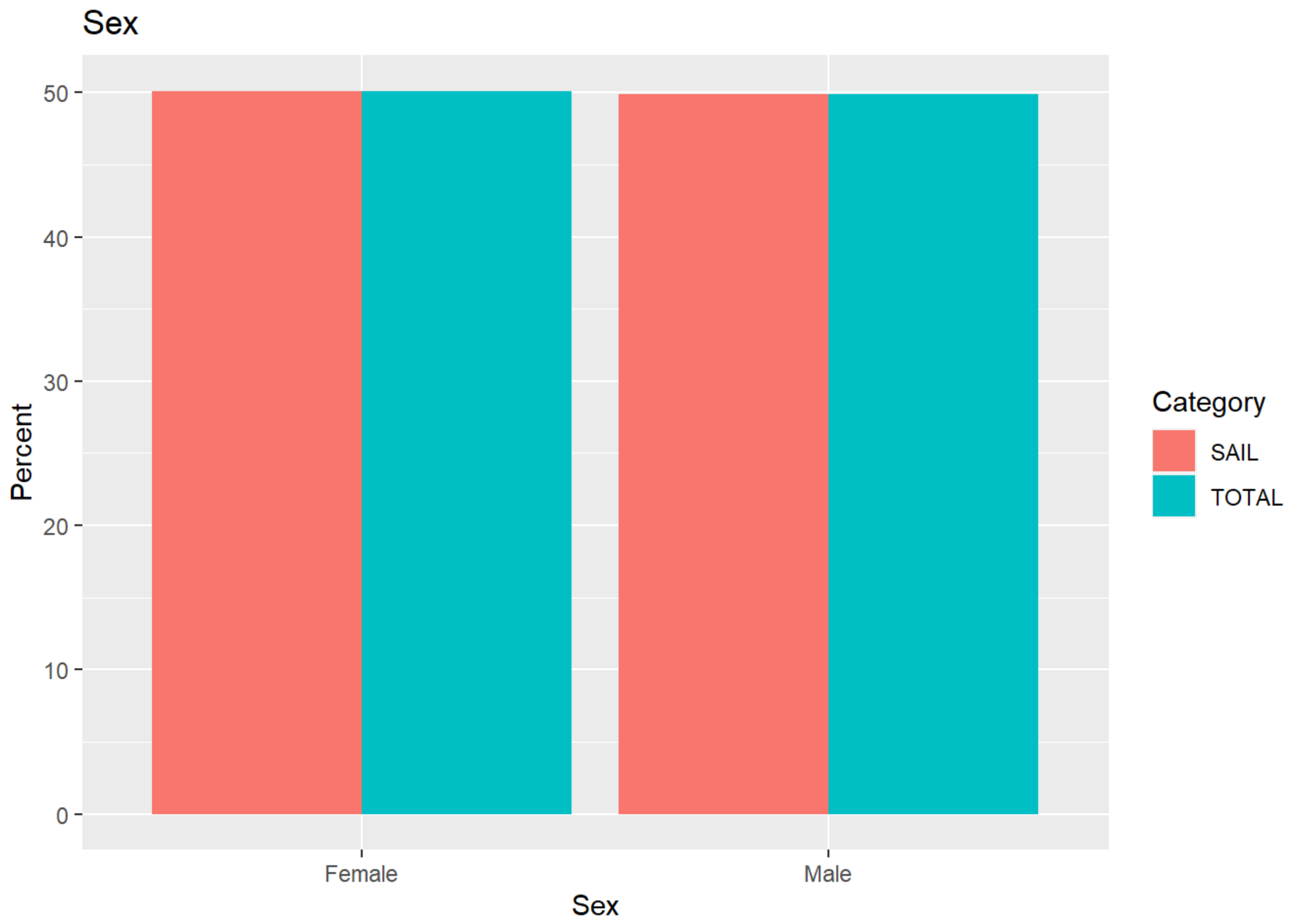

Health Board	SAIL General Practices (Percent by No. of Practices)
Aneurin Bevan LHB	72.36
Betsi Cadwaladr University LHB	75.75
Cardiff and Vale University LHB	83.60
Cwm Taf Morgannwg University Local Health Board	88.88
Hywel Dda LHB	79.16
Powys Teaching LHB	43.75
Swansea Bay University Local Health Board	95.38
TOTAL	79.80

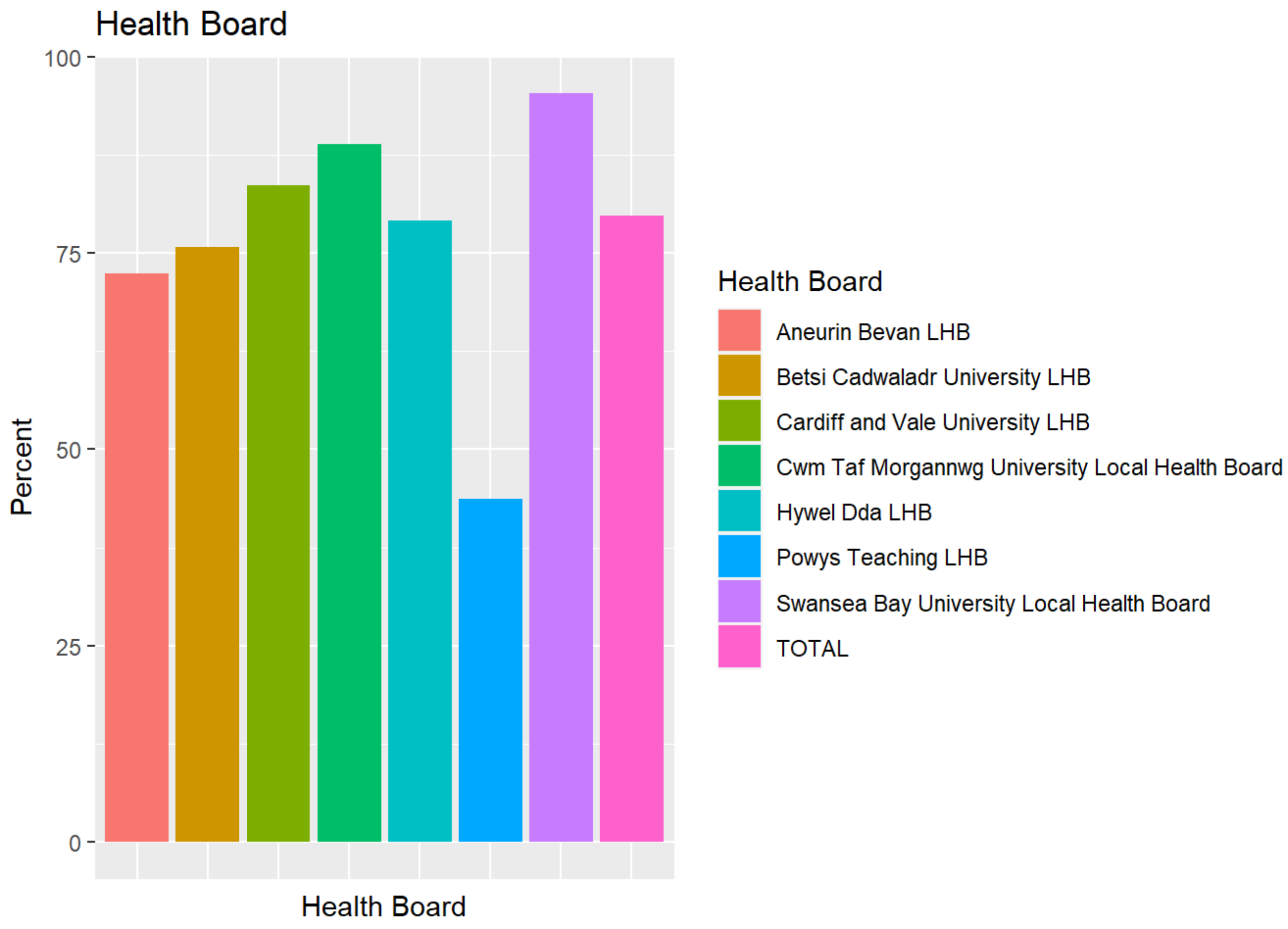
Health Board
100
75
50
25
0
Percent
Health Board
Health Board
Aneurin Bevan LHB
Betsi Cadwaladr University LHB
Cardiff and Vale University LHB
Cwm Taf Morgannwg University Local Health Board
Hywel Dda LHB
Powys Teaching LHB
Swansea Bay University Local Health Board
TOTAL

review only

Supplementary

10- year multi-morbidity patterns among people with and without rheumatic and musculoskeletal diseases: An observational cohort study using linked electronic health records from Wales, UK

For peer review only

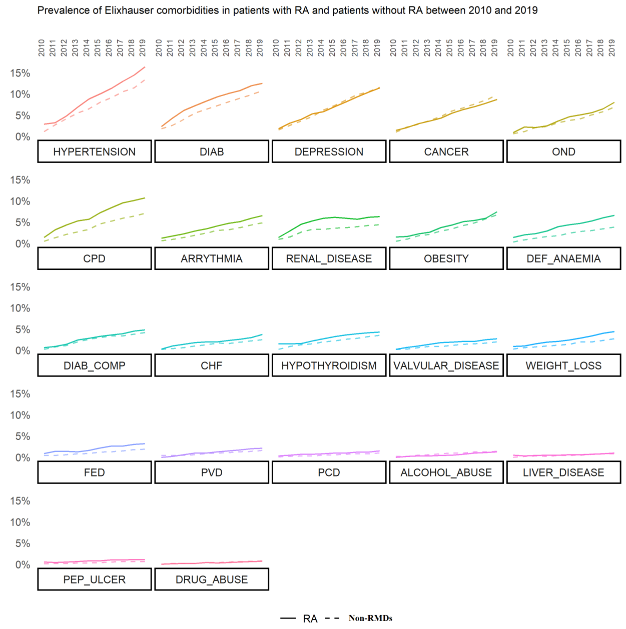

(a) Annual prevalence of Elixhauser co-morbidities in PMR cases and matched comparators 2010-2019

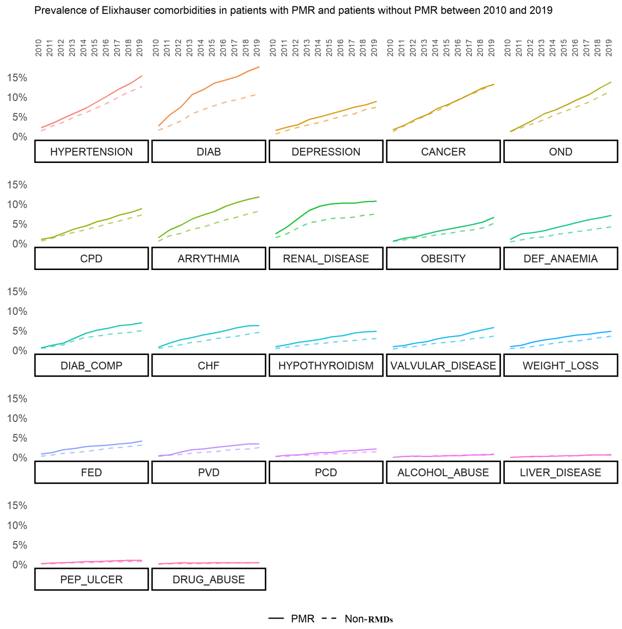

(b) Annual prevalence of Elixhauser co-morbidities in PMR cases and matched comparators 2010-2019

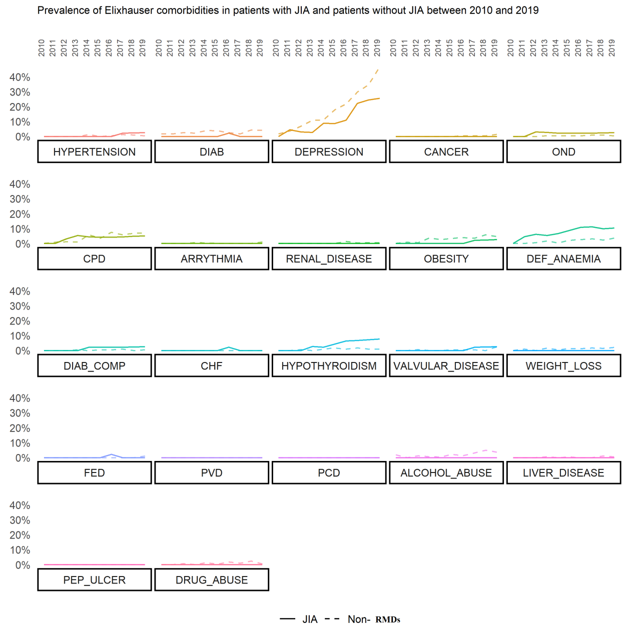

(c) Annual prevalence of Elixhauser co-morbidities in JIA cases and matched comparators 2010-2019

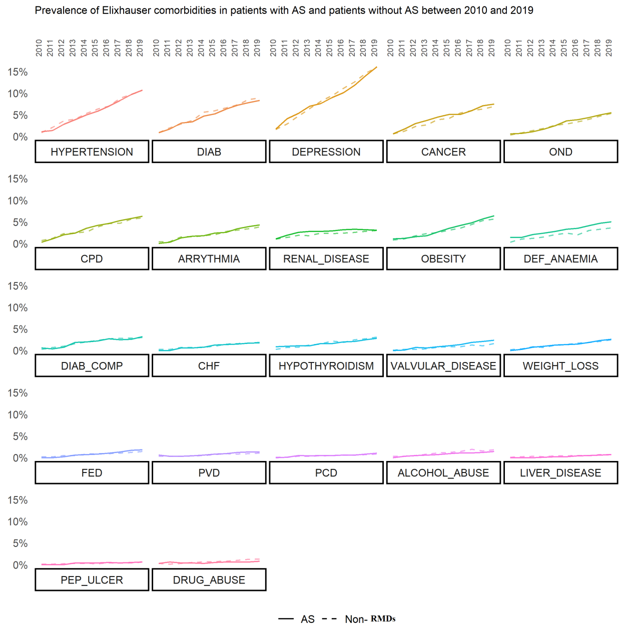

(d) Annual prevalence of Elixhauser co-morbidities in JIA cases and matched comparators 2010-2019

Figure 1. Annual prevalence of Elixhauser co-morbidities in RMDs subtype 2010-2019

The Figures 1 illustrate the annual prevalence for of RMDs subtype: RA(Rheumatoid arthritis), CTD - connective tissue diseases-, PMR (polymyalgia rheumatica and giant cell arteritis cases) and JIA: juvenile arthritis and non RMDs from 2010-2019.

....

The Figures 2 illustrate the age-specific analysis, in relation to specific co-Elixhauser morbidities for RMDs and comparators between 2010-2018.

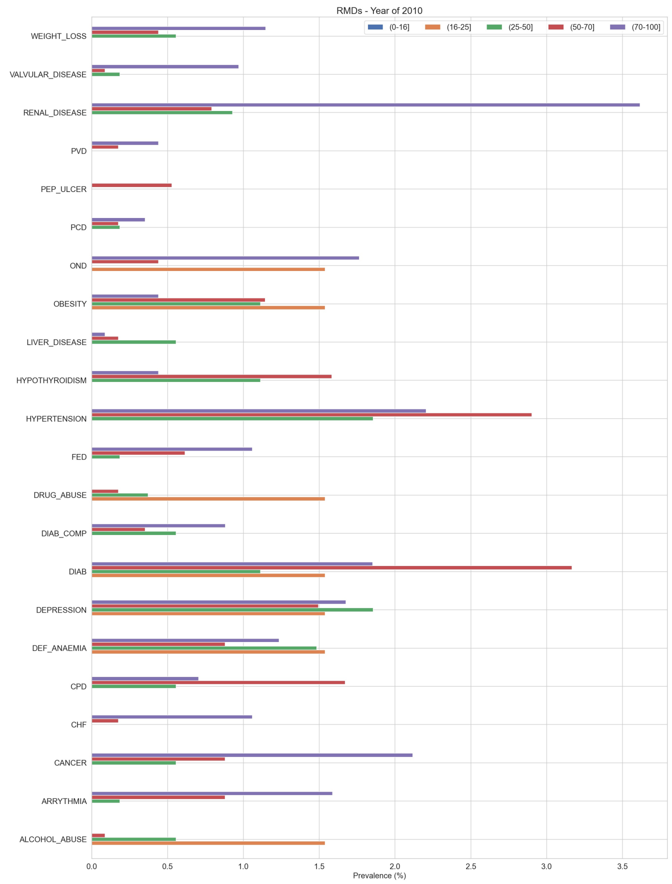

(a) age-specific analysis, especially in relation to specific co-morbidities

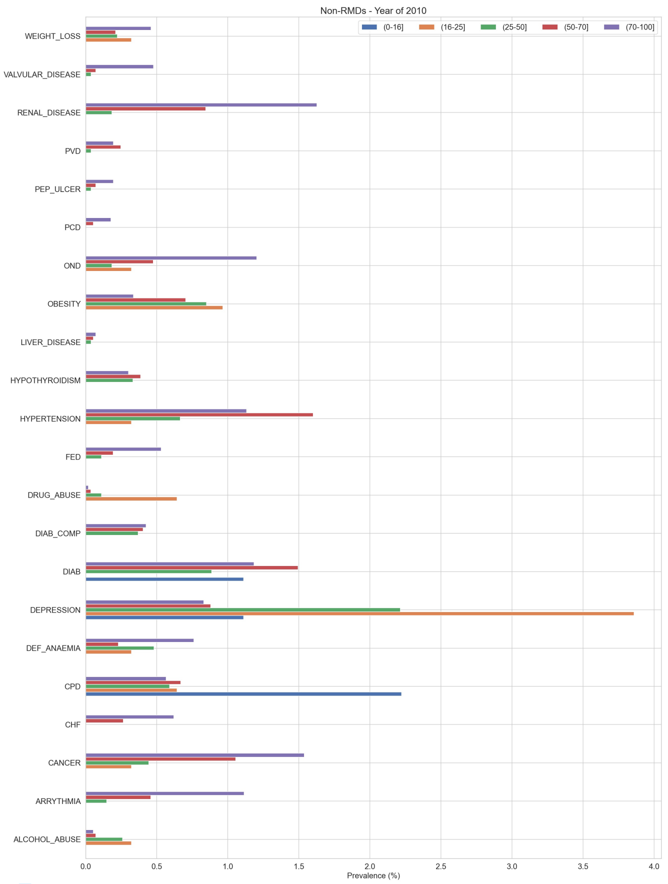

(b) age-specific analysis, especially in relation to specific co-morbidities

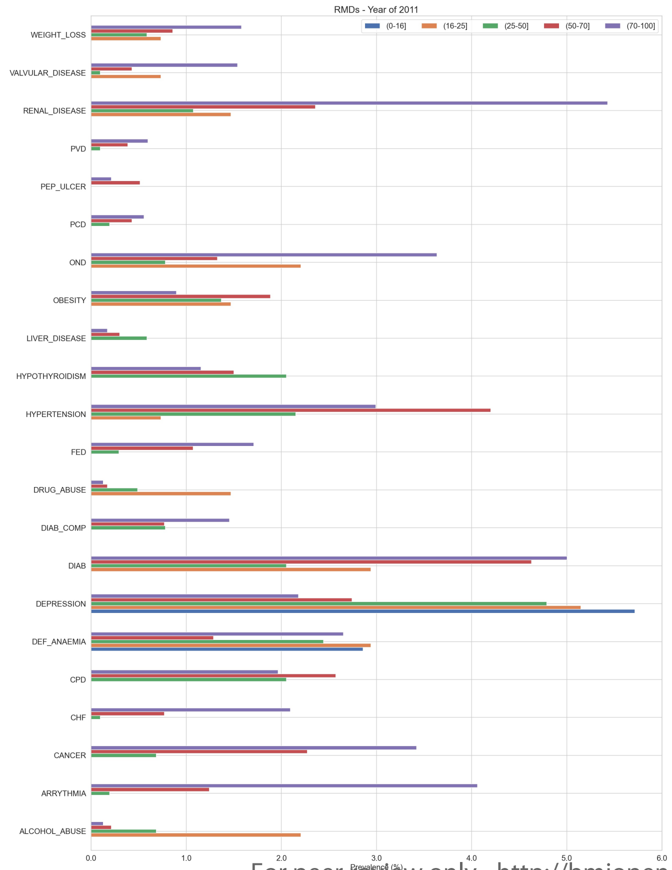

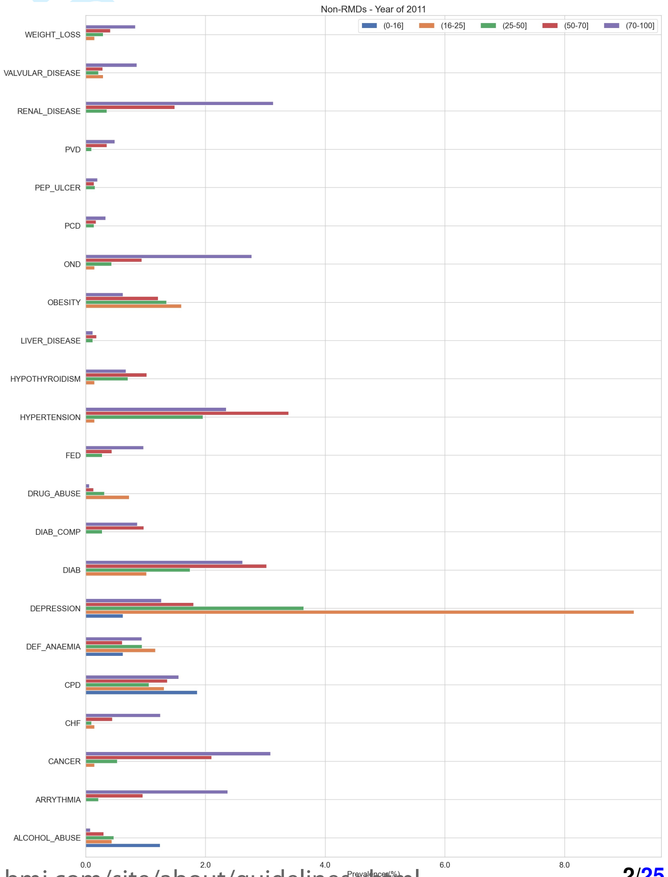

For peer review only - http://bmjopen.bmj.com/site/about/guidelines.xhtml

(c) age-specific analysis, especially in relation to specific co-morbidities

(d) age-specific analysis, especially in relation to specific co-morbidities

Figure 2. age-specific analysis, in relation to specific Elixhauser co-morbidities

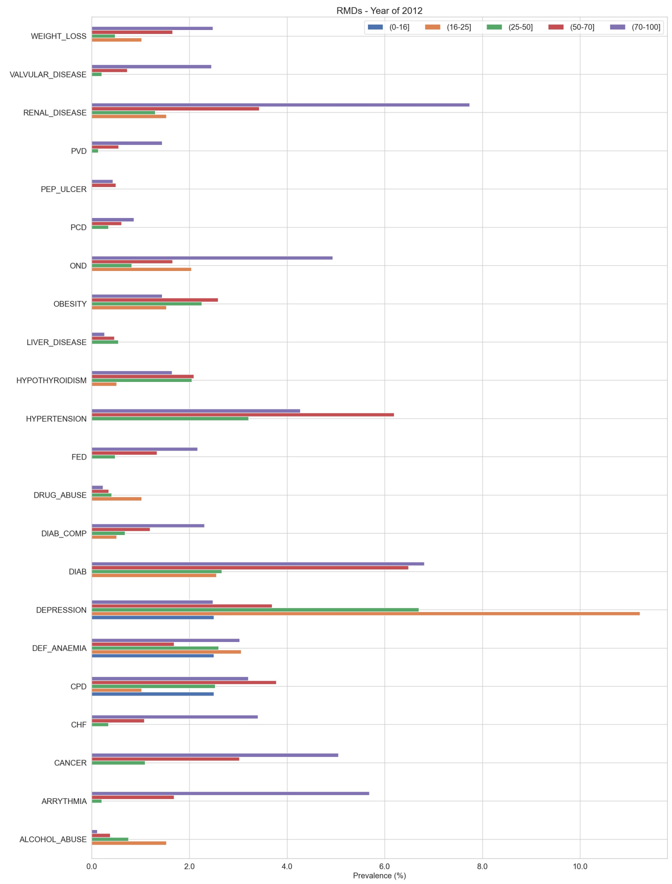

(e) age-specific analysis, especially in relation to specific co-morbidities

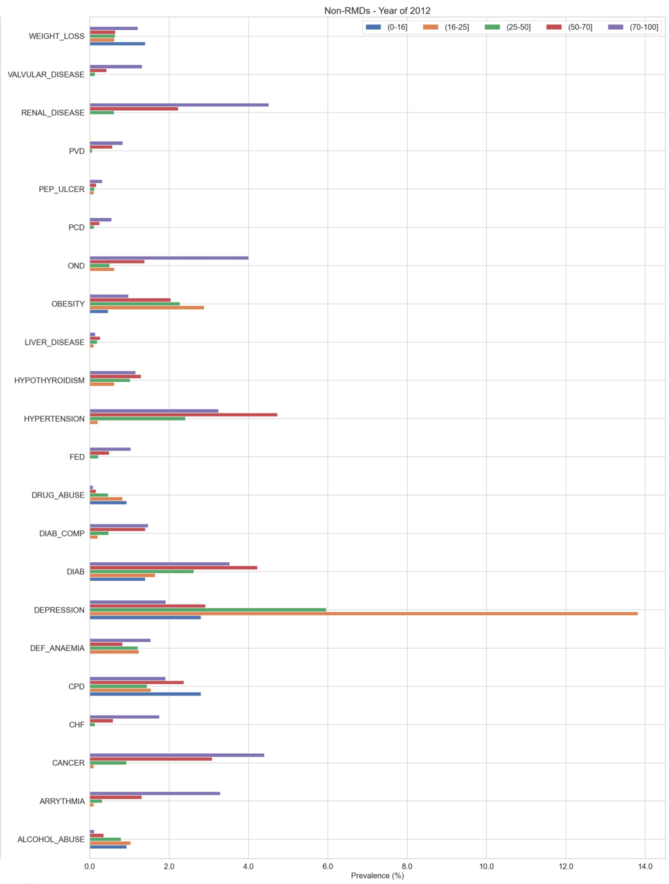

(f) age-specific analysis, especially in relation to specific co-morbidities

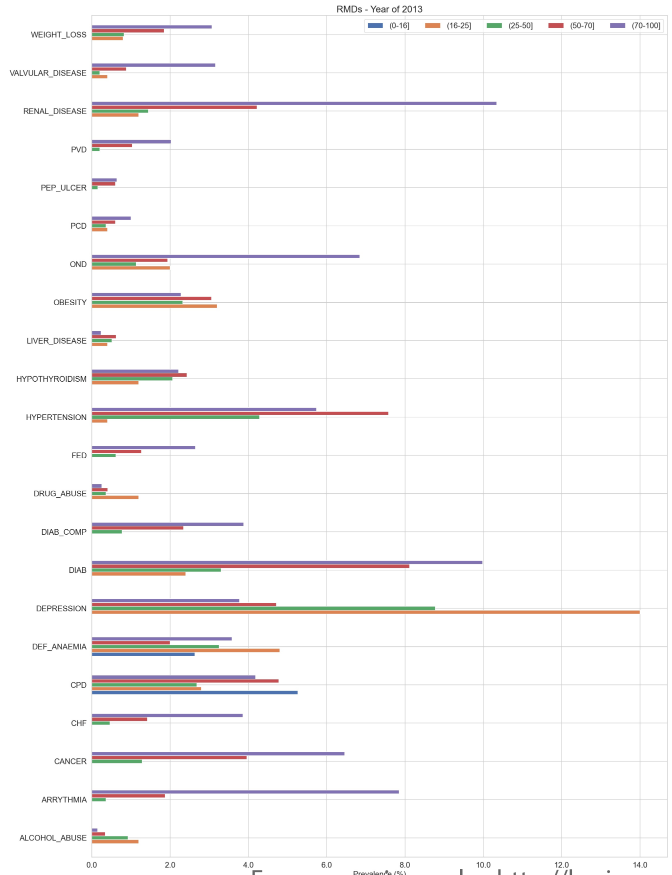

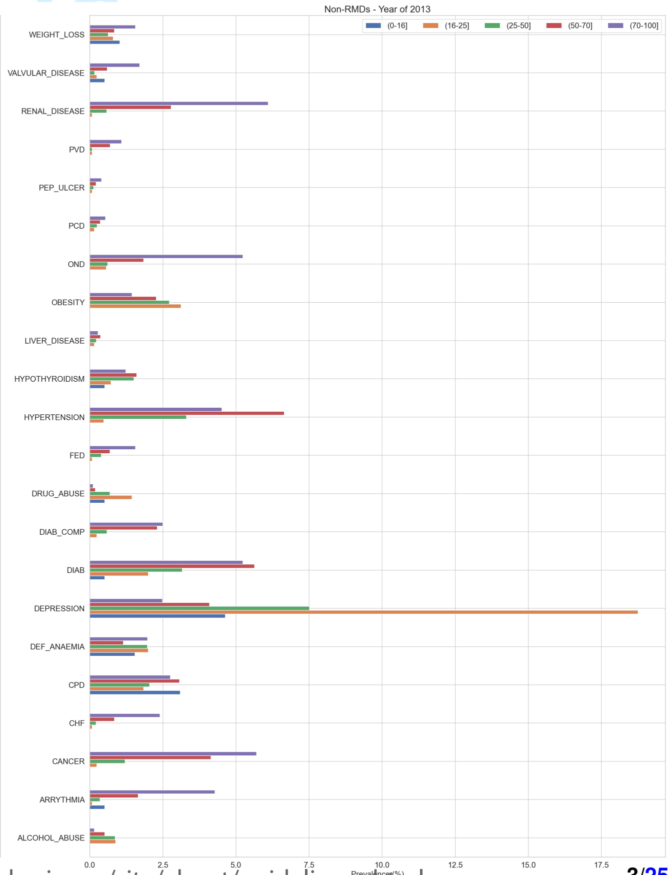

(g) age-specific analysis, especially in relation to specific co-morbidities

(h) age-specific analysis, especially in relation to specific co-morbidities

Figure 2. age-specific analysis, in relation to specific Elixhauser co-morbidities

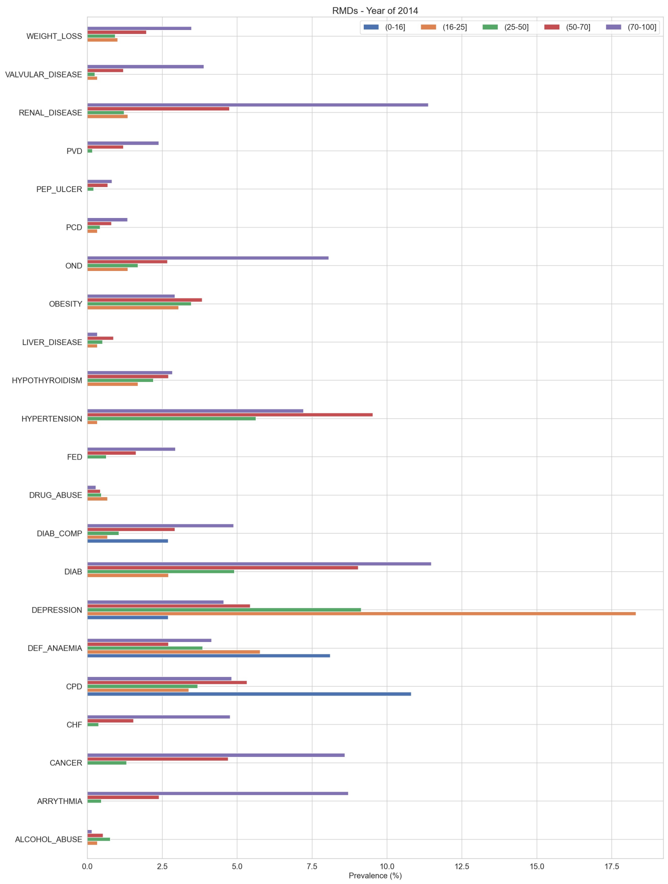

(i) age-specific analysis, especially in relation to specific co-morbidities,

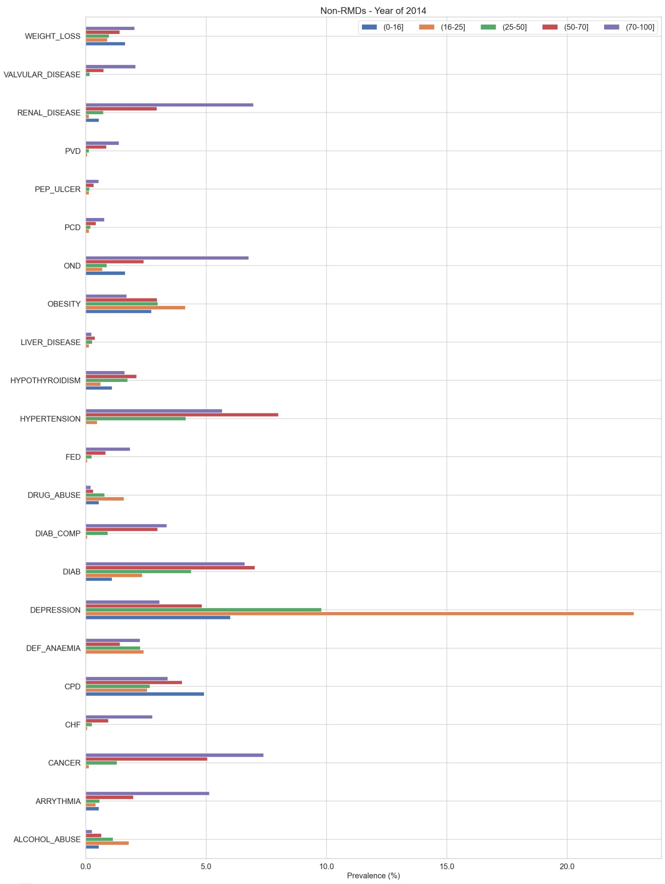

(j) age-specific analysis, especially in relation to specific co-morbidities,

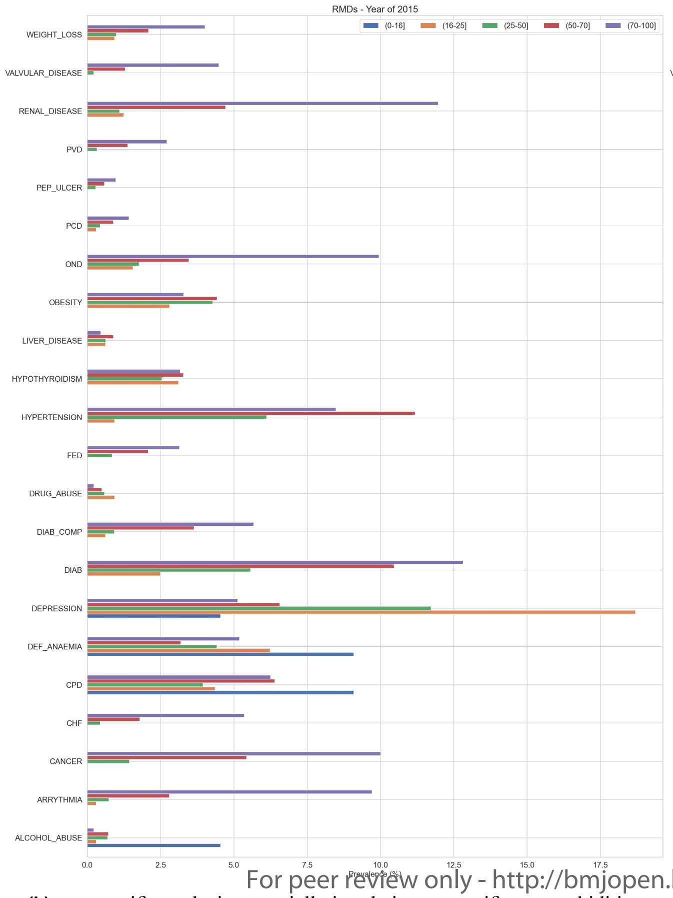

(k) age-specific analysis, especially in relation to specific co-morbidities

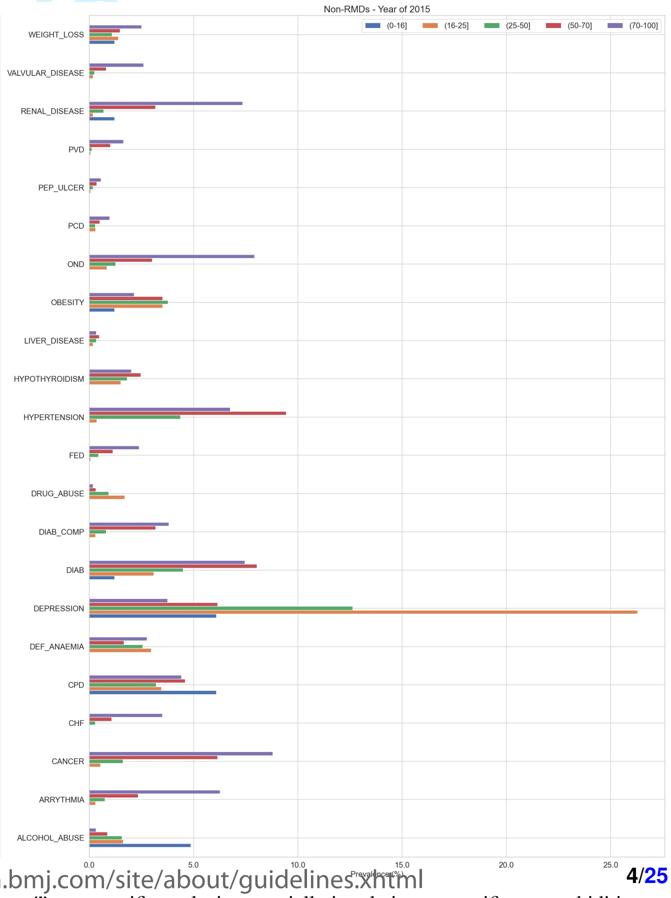

(l) age-specific analysis, especially in relation to specific co-morbidities

Figure 2. age-specific analysis, in relation to specific Elixhauser co-morbidities

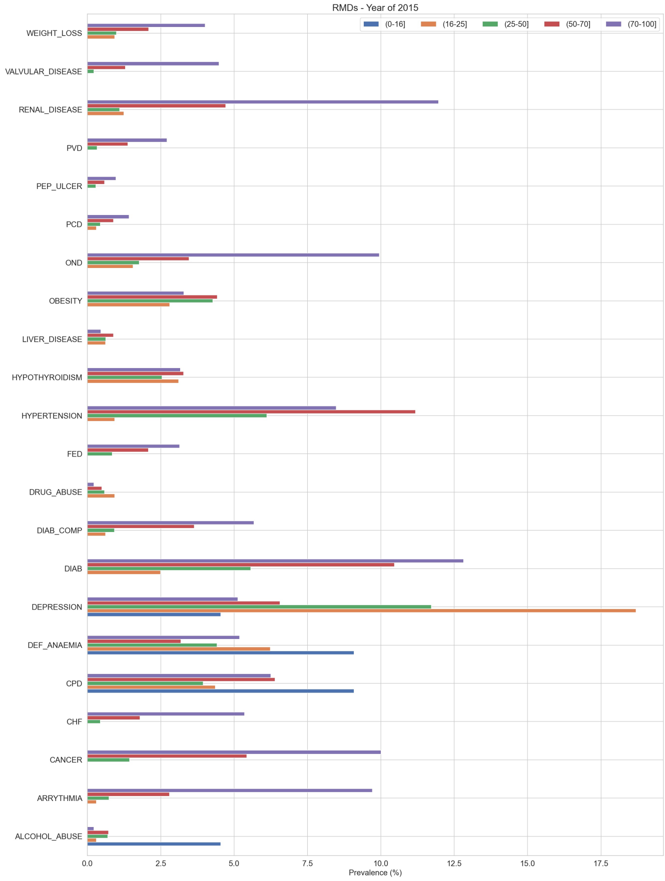

(m) age-specific analysis, especially in relation to specific co-morbidities,

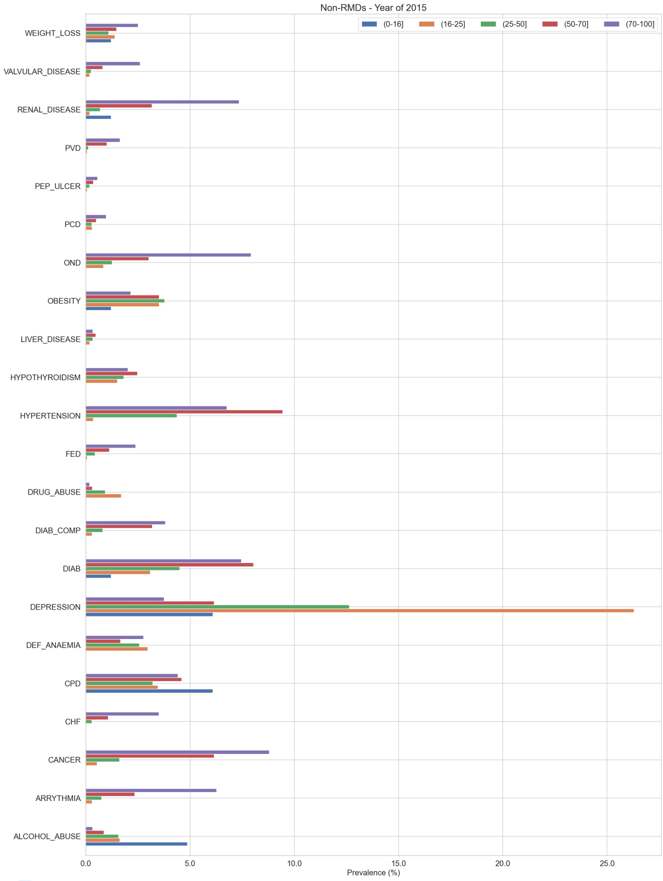

(n) age-specific analysis, especially in relation to specific co-morbidities,

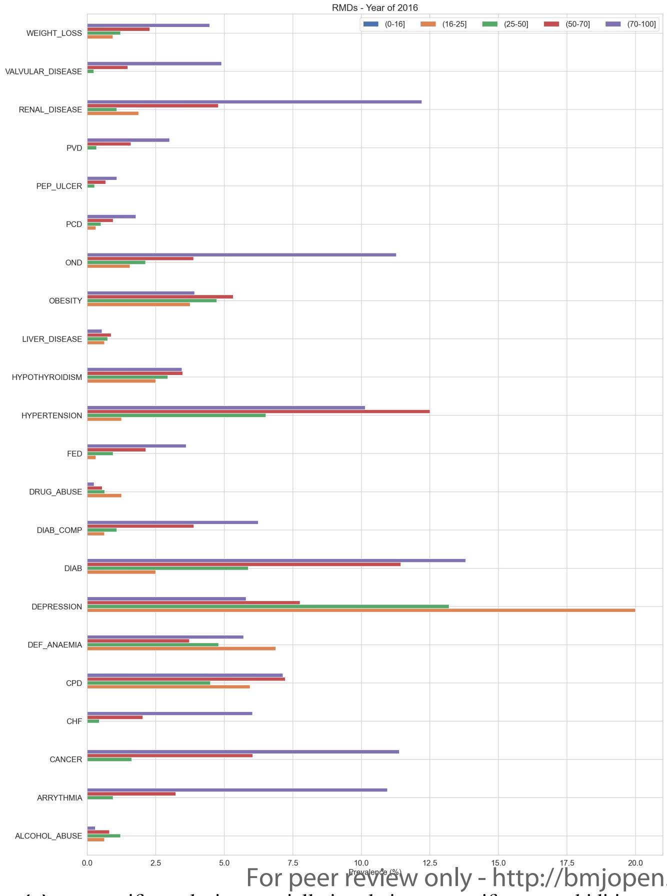

(o) age-specific analysis, especially in relation to specific co-morbidities,

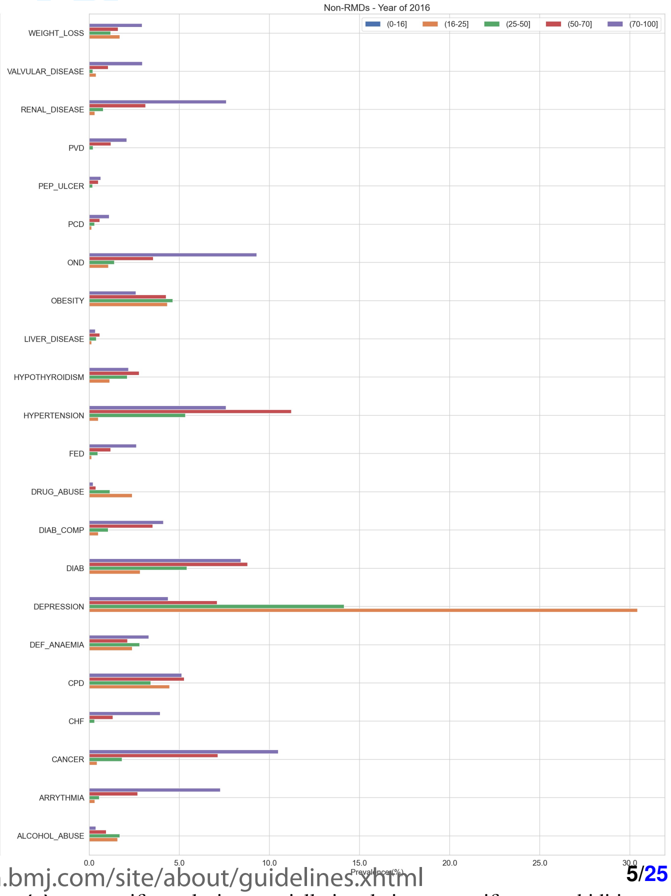

(p) age-specific analysis, especially in relation to specific co-morbidities,

Figure 2. age-specific analysis, in relation to specific Elixhauser co-morbidities

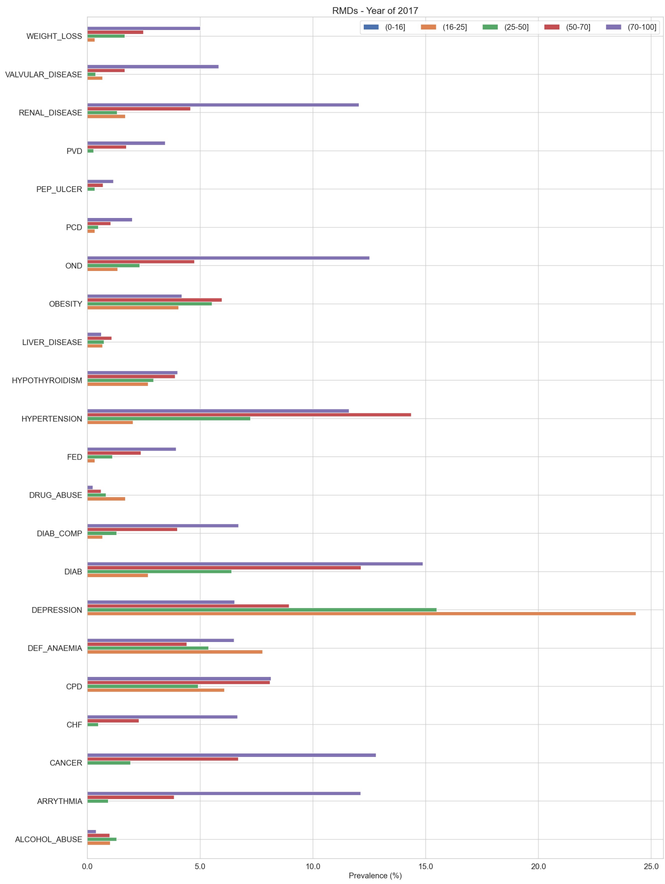

(q) age-specific analysis, especially in relation to specific co-morbidities,

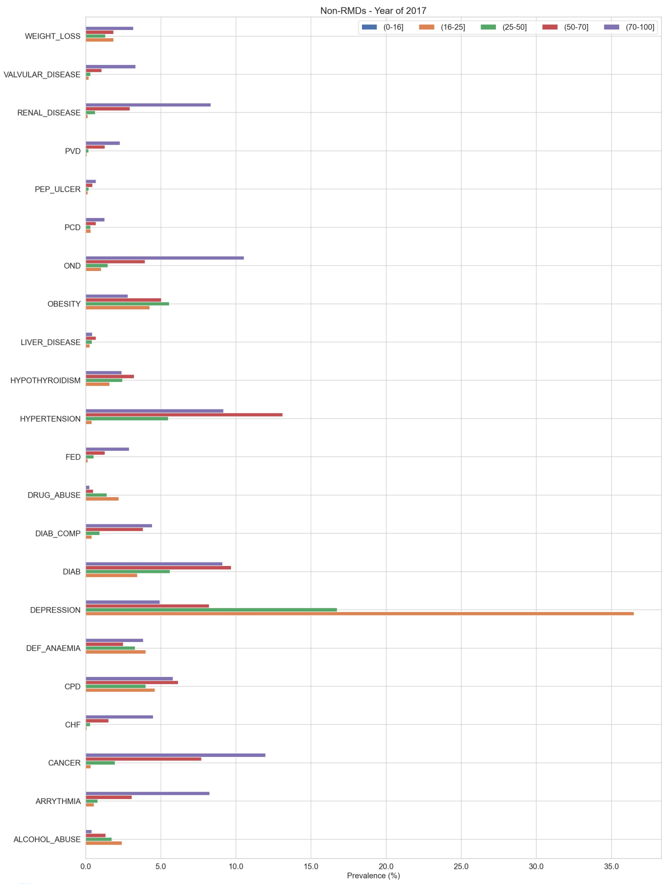

(r) age-specific analysis, especially in relation to specific co-morbidities

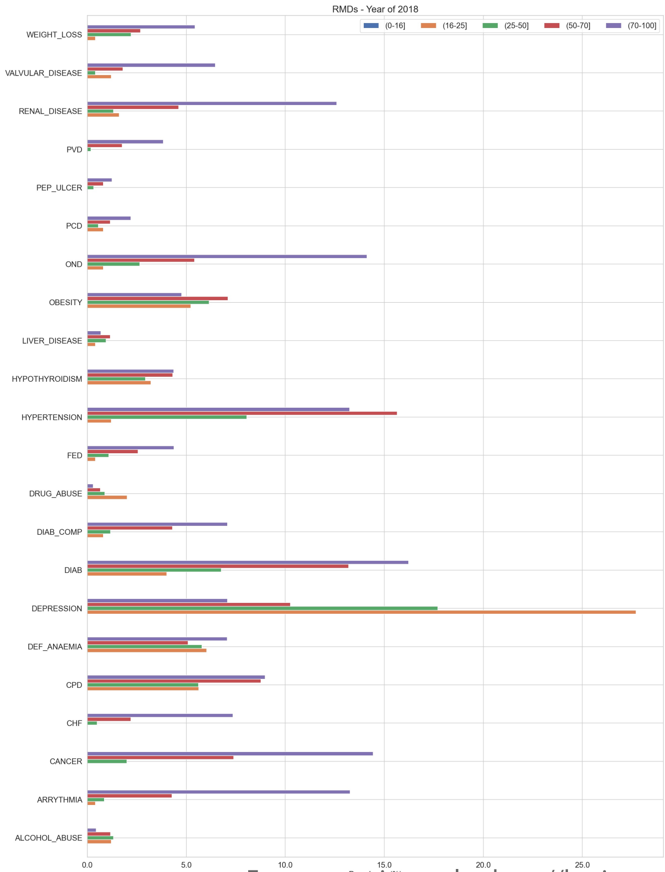

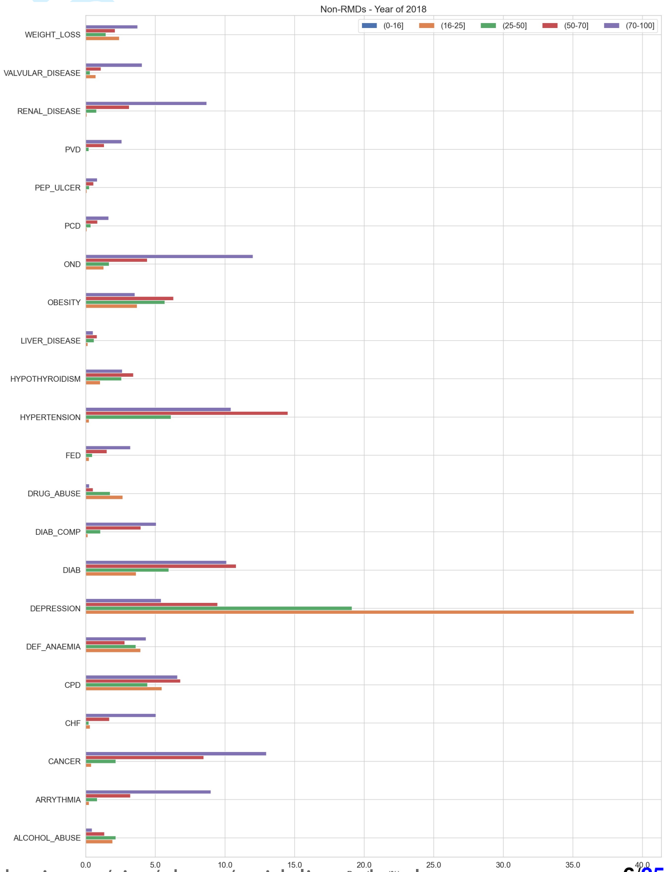

6/25

(s) age-specific analysis, especially in relation to specific co-morbidities, **(t)** age-specific analysis, especially in relation to specific co-morbidities,

Figure 2. age-specific analysis, in relation to specific Elixhauser co-morbidities

	2010		2011)		(2012)		2013		2014		2015		2016		2017		2018		2019	
Conditions	RMD	Comp	RMD	Comp	RMD	Comp	RMD	Comp	RMD	Comp	RMD	Comp	RMD	Comp	RMD	Comp	RMD	Comp	RMD	Comp
ALCOHOL_ABUSE	0.2 (0.02 0.32)	0.1 (0.05-0.16)	0.3 (0.17 0.45)	0.2 (0.19-0.31)	0.4 (0.23 0.49)	0.4 (0.30-0.41)	0.4 (0.27 0.49)	0.4 (0.38-0.49)	0.4 (0.30 0.51)	0.6 (0.54-0.65)	0.5 (0.40 0.61)	0.8 (0.72-0.84)	0.6 (0.53 0.76)	0.8 (0.76-0.88)	0.8 (0.66 0.89)	1.0 (0.94-1.06)	0.9 (0.74 0.99)	1.1 (1.02-1.14)	1.0 (0.86 1.11)	1.3 (1.21-1.33)
ARRYTHMIA	1.0 (0.64 1.37)	0.6 (0.51-0.77)	2.1 (1.78 2.52)	1.4 (1.23-1.50)	3.0 (2.64 3.36)	1.9 (1.77-2.03)	4.0 (3.64 4.35)	2.5 (2.33-2.59)	4.6 (4.30 4.98)	3.0 (2.90-3.15)	5.4 (5.01 5.69)	3.7 (3.58-3.84)	6.1 (5.80 6.48)	4.3 (4.16-4.42)	7.0 (6.65 7.34)	5.0 (4.84-5.11)	7.8 (7.45 8.15)	5.4 (5.30-5.57)	8.4 (8.02 8.71)	6.2 (6.11-6.38)
CPD	1.0 (0.67 1.41)	0.6 (0.49-0.75)	2.2 (1.79 2.54)	1.4 (1.25-1.52)	3.3 (2.89 3.64)	2.0 (1.88-2.14)	4.1 (3.78 4.51)	2.7 (2.61-2.88)	4.8 (4.47 5.17)	3.5 (3.38-3.65)	5.9 (5.55 6.26)	4.3 (4.15-4.42)	6.7 (6.38 7.10)	4.9 (4.77-5.04)	7.6 (7.24 7.96)	5.6 (5.51-5.79)	8.4 (7.99 8.72)	6.3 (6.19-6.48)	9.1 (8.74 9.46)	6.9 (6.80-7.09)
CHF	0.5 (0.23 0.74)	0.3 (0.25-0.44)	1.2 (0.89 1.43)	0.7 (0.60-0.79)	1.9 (1.57 2.15)	1.0 (0.87-1.06)	2.2 (1.95 2.48)	1.3 (1.26-1.44)	2.7 (2.39 2.91)	1.6 (1.48-1.66)	3.0 (2.79 3.31)	2.0 (1.86-2.05)	3.5 (3.21 3.73)	2.3 (2.16-2.35)	3.9 (3.65 4.17)	2.6 (2.53-2.72)	4.3 (3.99 4.52)	3.0 (2.87-3.07)	4.5 (4.28 4.81)	3.2 (3.15-3.35)
DEF_ANAEMIA	1.1 (0.75 1.53)	0.5 (0.37-0.60)	2.1 (1.71 2.44)	0.8 (0.71-0.91)	2.4 (2.09 2.74)	1.2 (1.08-1.29)	2.9 (2.61 3.22)	1.6 (1.54-1.75)	3.6 (3.27 3.87)	1.9 (1.83-2.03)	4.3 (4.00 4.61)	2.3 (2.20-2.41)	4.8 (4.50 5.10)	2.7 (2.64-2.84)	5.5 (5.23 5.85)	3.2 (3.12-3.34)	6.1 (5.79 6.41)	3.6 (3.52-3.74)	6.8 (6.44 7.07)	4.0 (3.86-4.08)
DEPRESSION	1.6 (1.16 2.09)	1.2 (1.00-1.35)	2.9 (2.52 3.38)	2.1 (1.91-2.24)	3.9 (3.47 4.29)	3.3 (3.11-3.45)	5.2 (4.79 5.60)	4.3 (4.17-4.50)	5.9 (5.55 6.32)	5.3 (5.12-5.45)	7.0 (6.63 7.40)	6.6 (6.41-6.75)	8.0 (7.59 8.36)	7.4 (7.26-7.59)	9.1 (8.72 9.50)	8.5 (8.31-8.65)	10.1 (9.74 10.53	9.4 (9.25-9.59)	11.2 (10.7 11.58	10.5 (10.31-10.65)
DIAB	2.2 (1.68 2.75)	1.2 (1.05-1.40)	4.3 (3.74 4.78)	2.6 (2.39-2.76)	5.8 (5.35 6.34)	3.6 (3.43-3.78)	7.9 (7.42 8.41)	5.0 (4.79-5.14)	9.2 (8.75 9.69)	6.3 (6.13-6.49)	10.5 (10.0 10.97	7.1 (6.95-7.30)	11.4 (10.9 11.89	8.0 (7.83-8.17)	12.3 (11.8 12.77	8.7 (8.54-8.89)	13.5 (13.0 13.94	9.7 (9.51-9.85)	14.4 (13.9 14.80	10.4 (10.21-10.56)
DIAB_COMP	0.6 (0.31 0.87)	0.4 (0.29-0.50)	1.0 (0.77 1.28)	0.8 (0.68-0.88)	1.5 (1.27 1.79)	1.2 (1.13-1.34)	2.6 (2.35 2.94)	2.0 (1.93-2.16)	3.4 (3.09 3.68)	2.7 (2.62-2.86)	4.0 (3.71 4.30)	3.0 (2.90-3.13)	4.4 (4.11 4.70)	3.3 (3.22-3.45)	4.7 (4.44 5.02)	3.6 (3.48-3.71)	5.1 (4.76 5.34)	4.0 (3.86-4.09)	5.5 (5.18 5.76)	4.3 (4.22-4.45)
DRUG_ABUSE	0.2 (0.02 0.32)	0.1 (0.02-0.09)	0.2 (0.11 0.36)	0.1 (0.10-0.19)	0.3 (0.21 0.45)	0.2 (0.16-0.24)	0.4 (0.25 0.46)	0.3 (0.23-0.31)	0.4 (0.28 0.48)	0.4 (0.33-0.42)	0.4 (0.31 0.50)	0.4 (0.34-0.43)	0.4 (0.35 0.54)	0.5 (0.41-0.50)	0.5 (0.41 0.60)	0.6 (0.51-0.60)	0.5 (0.45 0.64)	0.6 (0.57-0.66)	0.6 (0.53 0.73)	0.7 (0.63-0.72)

Table 1 – continued from previous page

FED	0.7 (0.39 0.99)	0.3 (0.21-0.39)	1.2 (0.89 1.43)	0.6 (0.52-0.70)	1.5 (1.23 1.74)	0.6 (0.57-0.72)	1.7 (1.45 1.92)	1.0 (0.90-1.06)	2.0 (1.74 2.20)	1.1 (1.06-1.21)	2.3 (2.06 2.52)	1.5 (1.45-1.62)	2.6 (2.33 2.78)	1.7 (1.59-1.75)	2.8 (2.62 3.07)	1.9 (1.78-1.94)	3.1 (2.91 3.37)	2.1 (2.03-2.20)	3.4 (3.19 3.65)	2.4 (2.29-2.46)
HYPERTENSION	2.4 (1.80 2.90)	1.2 (1.03-1.38)	3.3 (2.80 3.71)	2.6 (2.45-2.81)	4.7 (4.29 5.19)	3.6 (3.44-3.79)	6.1 (5.66 6.53)	5.1 (4.89-5.25)	7.7 (7.28 8.15)	6.2 (6.06-6.41)	9.0 (8.58 9.45)	7.3 (7.13-7.48)	10.4 (9.93 10.80	8.6 (8.38-8.73)	11.9 (11.4 12.31	10.0 (9.85-10.21)	13.3 (12.8 13.70	11.2 (11.06-11.43)	15.0 (14.5 15.40	12.6 (12.39-12.77)
HYPOTHYROIDISM	1.0 (0.64 1.37)	0.3 (0.24-0.43)	1.4 (1.11 1.72)	0.8 (0.70-0.90)	1.9 (1.57 2.15)	1.2 (1.07-1.27)	2.2 (1.98 2.52)	1.4 (1.32-1.51)	2.7 (2.39 2.91)	1.8 (1.72-1.91)	3.1 (2.85 3.37)	2.2 (2.06-2.26)	3.4 (3.11 3.62)	2.4 (2.29-2.48)	3.8 (3.52 4.04)	2.7 (2.62-2.81)	4.1 (3.86 4.38)	2.9 (2.81-3.01)	4.3 (4.06 4.58)	3.1 (3.02-3.22)
LIVER_DISEASE	0.2 (0.04 0.37)	0.1 (0.02-0.09)	0.3 (0.15 0.43)	0.1 (0.10-0.18)	0.4 (0.25 0.51)	0.2 (0.16-0.24)	0.4 (0.32 0.56)	0.3 (0.26-0.35)	0.6 (0.45 0.70)	0.3 (0.26-0.34)	0.7 (0.54 0.78)	0.4 (0.35-0.43)	0.7 (0.59 0.83)	0.4 (0.39-0.48)	0.8 (0.70 0.94)	0.5 (0.49-0.57)	0.9 (0.78 1.02)	0.6 (0.59-0.69)	1.0 (0.91 1.17)	0.7 (0.68-0.78)
OBESITY	0.9 (0.53 1.20)	0.6 (0.46-0.71)	1.4 (1.08 1.68)	1.0 (0.89-1.12)	2.0 (1.74 2.33)	1.7 (1.54-1.79)	2.6 (2.32 2.90)	2.0 (1.91-2.14)	3.4 (3.07 3.66)	2.5 (2.36-2.59)	3.9 (3.59 4.17)	3.0 (2.87-3.10)	4.6 (4.30 4.90)	3.6 (3.49-3.72)	5.1 (4.79 5.39)	4.1 (4.00-4.25)	5.9 (5.57 6.18)	4.9 (4.80-5.05)	7.0 (6.69 7.33)	5.9 (5.74-6.00)
OND	0.9 (0.56 1.24)	0.7 (0.56-0.83)	2.2 (1.79 2.54)	1.6 (1.42-1.70)	2.8 (2.49 3.19)	2.3 (2.12-2.40)	3.8 (3.44 4.14)	3.0 (2.84-3.11)	4.7 (4.36 5.06)	3.9 (3.79-4.07)	5.9 (5.55 6.26)	4.8 (4.63-4.91)	6.7 (6.39 7.11)	5.6 (5.50-5.79)	7.8 (7.40 8.12)	6.4 (6.29-6.58)	8.9 (8.53 9.27)	7.4 (7.26-7.56)	10.2 (9.86 10.62	8.6 (8.41-8.73)
PEP_ULCER	0.2 (0.04 0.37)	0.1 (0.06-0.16)	0.3 (0.15 0.43)	0.2 (0.11-0.21)	0.4 (0.24 0.50)	0.2 (0.17-0.26)	0.5 (0.40 0.66)	0.3 (0.23-0.32)	0.6 (0.52 0.78)	0.4 (0.34-0.43)	0.7 (0.57 0.82)	0.4 (0.37-0.45)	0.8 (0.65 0.90)	0.5 (0.46-0.55)	0.8 (0.71 0.96)	0.5 (0.47-0.55)	0.9 (0.80 1.05)	0.6 (0.58-0.67)	1.0 (0.85 1.09)	0.7 (0.63-0.72)
PVD	0.2 (0.06 0.42)	0.2 (0.11-0.25)	0.4 (0.25 0.57)	0.4 (0.28-0.42)	0.8 (0.63 1.02)	0.6 (0.50-0.65)	1.3 (1.07 1.48)	0.7 (0.67-0.81)	1.5 (1.30 1.69)	0.9 (0.87-1.01)	1.8 (1.55 1.95)	1.1 (1.05-1.19)	2.0 (1.78 2.17)	1.4 (1.33-1.48)	2.2 (2.04 2.44)	1.5 (1.46-1.61)	2.4 (2.24 2.64)	1.7 (1.64-1.79)	2.5 (2.31 2.71)	1.9 (1.79-1.94)
PCD	0.2 (0.06 0.42)	0.1 (0.04-0.14)	0.4 (0.26 0.59)	0.2 (0.17-0.28)	0.7 (0.48 0.82)	0.3 (0.28-0.39)	0.7 (0.56 0.87)	0.4 (0.35-0.46)	1.0 (0.79 1.11)	0.5 (0.47-0.58)	1.0 (0.88 1.19)	0.7 (0.62-0.73)	1.2 (1.07 1.38)	0.8 (0.70-0.81)	1.4 (1.21 1.52)	0.9 (0.82-0.94)	1.5 (1.37 1.69)	1.1 (1.07-1.19)	1.7 (1.58 1.91)	1.2 (1.10-1.22)
RENAL_DISEASE	1.9 (1.40 2.40)	1.0 (0.84-1.17)	3.3 (2.87 3.78)	1.9 (1.75-2.06)	4.7 (4.29 5.19)	2.8 (2.65-2.97)	6.2 (5.72 6.60)	3.7 (3.53-3.84)	6.8 (6.42 7.24)	4.2 (4.05-4.34)	7.1 (6.75 7.53)	4.5 (4.35-4.63)	7.3 (6.98 7.72)	4.6 (4.51-4.77)	7.3 (6.98 7.68)	4.9 (4.79-5.05)	7.7 (7.36 8.06)	5.3 (5.13-5.39)	7.8 (7.44 8.12)	5.5 (5.39-5.64)

Table 1 – continued from previous page

CANCER	1.3 (0.87 1.69)	1.1 (0.94-1.28)	2.4 (2.00 2.78)	2.2 (2.00-2.33)	3.4 (3.04 3.81)	3.2 (3.00-3.33)	4.4 (4.05 4.80)	4.2 (4.02-4.35)	5.6 (5.27 6.03)	5.3 (5.13-5.46)	6.6 (6.25 7.00)	6.4 (6.27-6.60)	7.6 (7.17 7.93)	7.6 (7.46-7.80)	8.6 (8.19 8.95)	8.6 (8.43-8.77)	9.7 (9.31 10.08	9.5 (9.30-9.64)	10.5 (10.1 10.90	10.6 (10.43-10.78)
VALVULAR_DISEASE	0.4 (0.21 0.69)	0.2 (0.14-0.30)	0.8 (0.59 1.05)	0.5 (0.42-0.58)	1.3 (1.07 1.55)	0.7 (0.64-0.80)	1.7 (1.44 1.91)	1.0 (0.89-1.05)	2.1 (1.91 2.38)	1.2 (1.10-1.26)	2.5 (2.22 2.68)	1.5 (1.39-1.55)	2.7 (2.50 2.96)	1.5 (1.39-1.55)	3.3 (3.05 3.53)	1.9 (1.85-2.02)	3.7 (3.46 3.95)	2.3 (2.22-2.40)	4.2 (3.92 4.42)	2.5 (2.46-2.64)
WEIGHT_LOSS	0.7(0	0.3 (0.22-0.40)	4)1.1(0	0.5 (0.46-0.63)	1.8 6)(1.49 2.05)	0.9 (0.79-0.96)	2.1 (1.88 2.41)	1.1 (1.01-1.18)	2.4 (2.15 2.65)	1.6 (1.50-1.68)	2.7 (2.46 2.95)	1.9 (1.76-1.94)	3.0 (2.79 3.28)	2.1 (2.02-2.20)	3.4 (3.20 3.69)	2.4 (2.26-2.45)	3.8 (3.59 4.09)	2.7 (2.65-2.84)	4.2 (3.92 4.42)	3.1 (2.98-3.17)

Table 1. Annual prevalence rates for comorbidities in RMDs cases and matched comparators 2010-2019

For peer review only

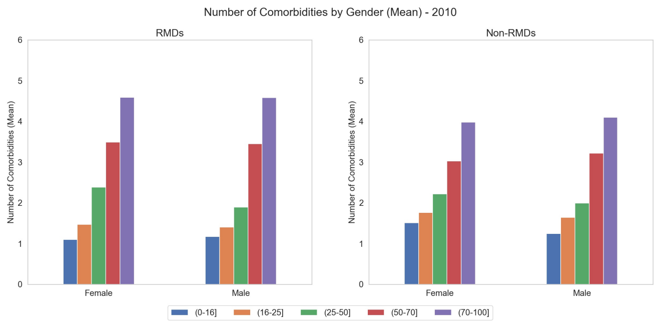

(a) Mean number of comorbidities in patients with RMDs and comparators by age categories and sex 2010

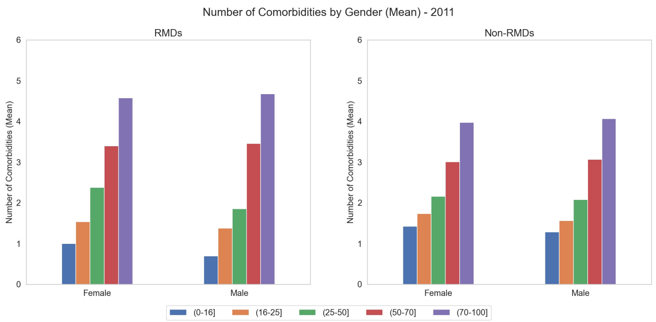

(b) Mean number of comorbidities in patients with RMDs and comparators by age categories and sex 2011

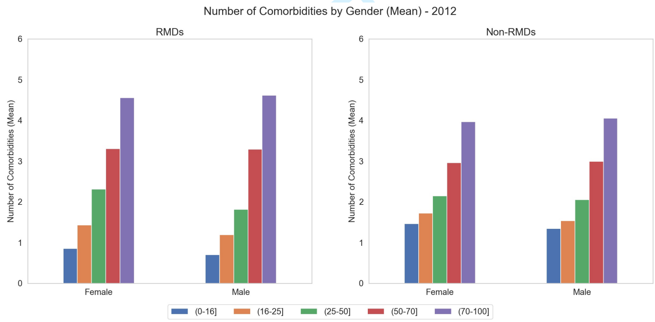

(c) Mean number of comorbidities in patients with RMDs and comparators by age categories and sex 2012

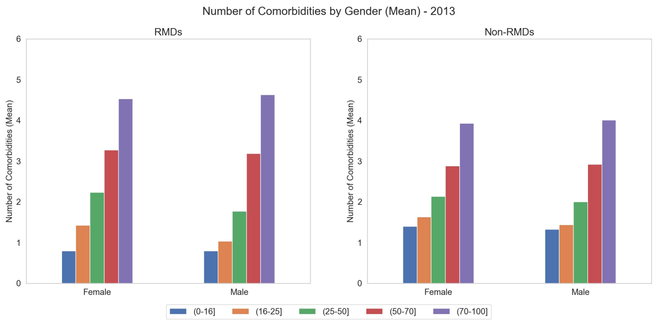

(d) Mean number of comorbidities in patients with RMDs and comparators by age categories and sex 2013

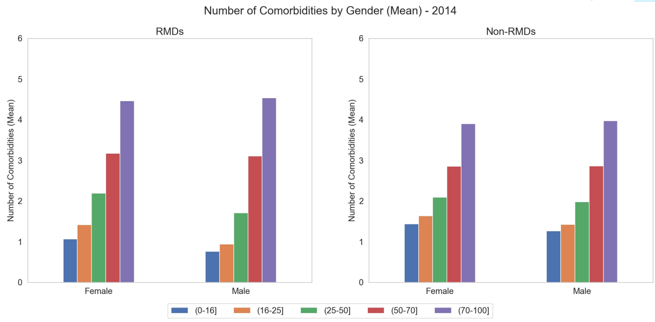

(e) Mean number of comorbidities in patients with RMDs and comparators by age categories and sex 2014

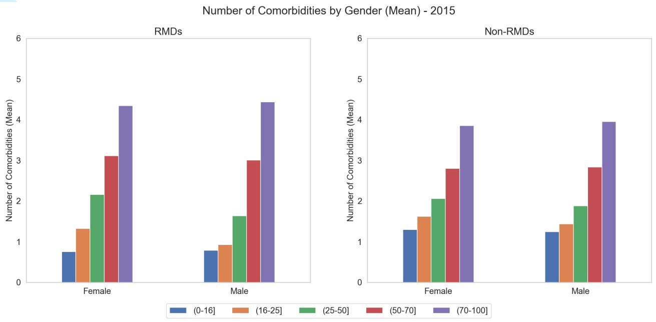

(f) Mean number of comorbidities in patients with RMDs and comparators by age categories and sex 2015

Figure 3. Mean number of comorbidities in patients with RMDs and comparators by age categories and sex 2010 and 2015

1 abbreviations

The Table 2 describes the significance of various abbreviations and acronyms used throughout of this paper.

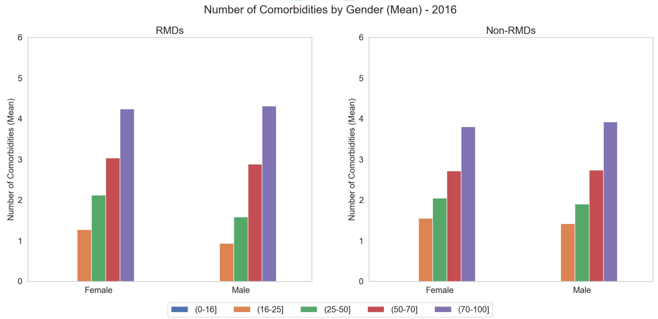

(g) Mean number of comorbidities in patients with RMDs and comparators by age categories and sex 2016

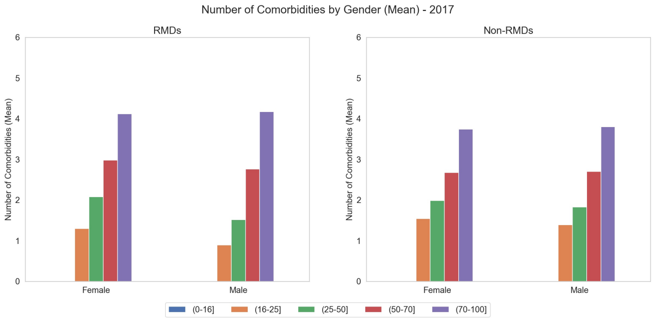

(h) Mean number of comorbidities in patients with RMDs and comparators by age categories and sex 2017

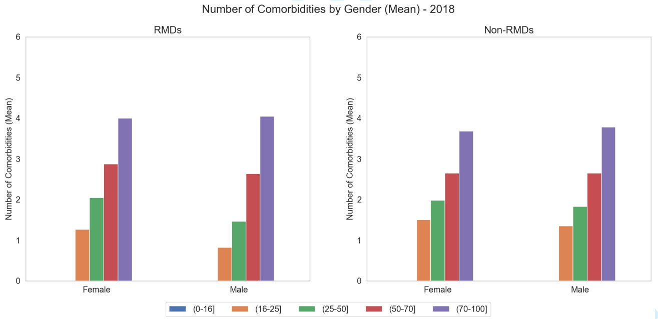

(i) Mean number of comorbidities in patients with RMDs and comparators by age categories and sex 2018

Figure 3. Mean number of comorbidities in patients with RMDs and comparators by age categories and sex 2016 and 2018

For peer review only - http://bmjopen.bmj.com/site/about/guidelines.xhtml

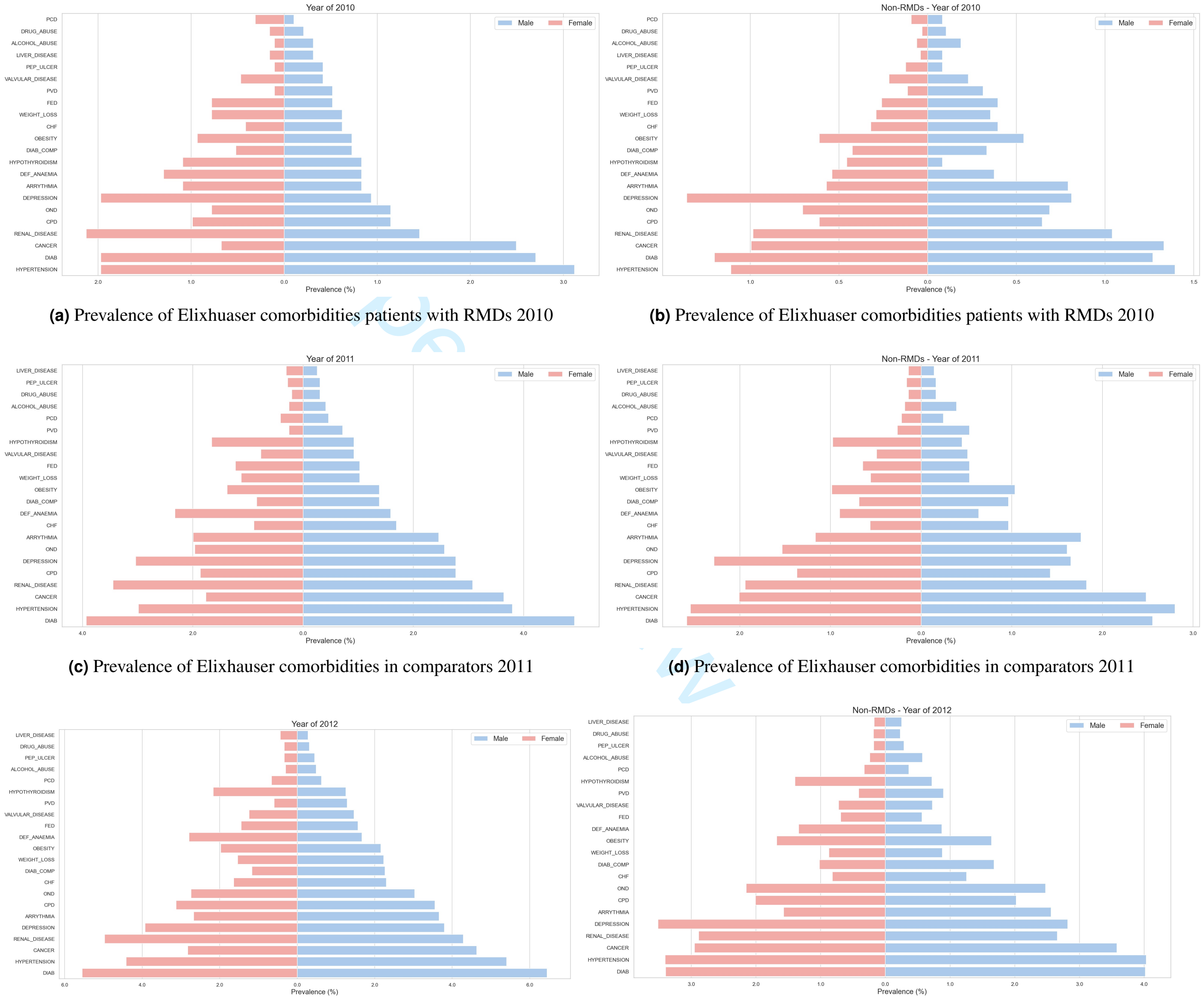

(a) Prevalence of Elixhuaser comorbidities patients with RMDs 2010

(b) Prevalence of Elixhuaser comorbidities patients with RMDs 2010

(c) Prevalence of Elixhauser comorbidities in comparators 2011

(d) Prevalence of Elixhauser comorbidities in comparators 2011

(e) Prevalence of Elixhuaser comorbidities patients with RMDs 2012

(f) Prevalence of Elixhauser comorbidities in comparators 2012

Figure 4. Prevalence of Elixhauser comorbidities in patients with RMDs and patients without RMDS in 2010 and 2012

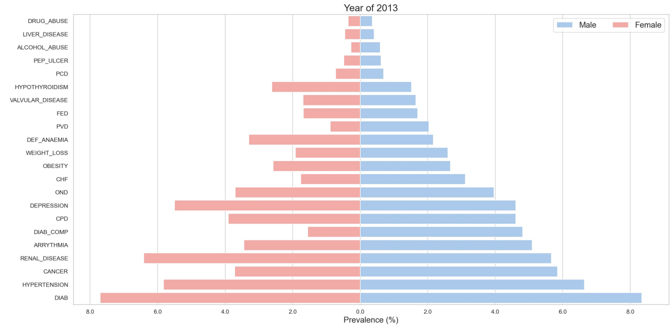

(g) Prevalence of Elixhuaser comorbidities patients with RMDs 2013

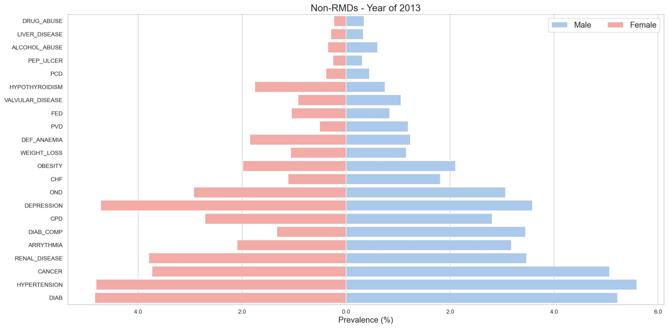

(h) Prevalence of Elixhuaser comorbidities patients without RMDs 2013

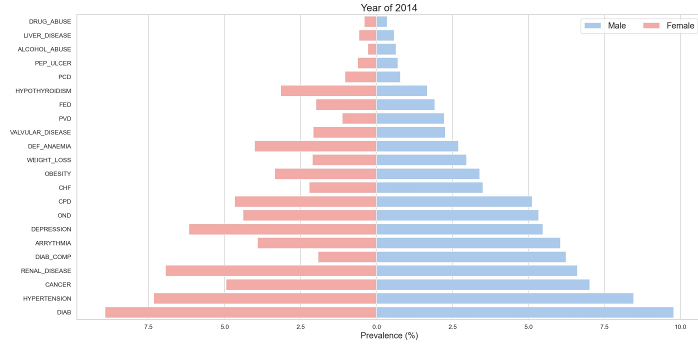

(i) Prevalence of Eixhuaser comorbidities patients with RMDs 2014

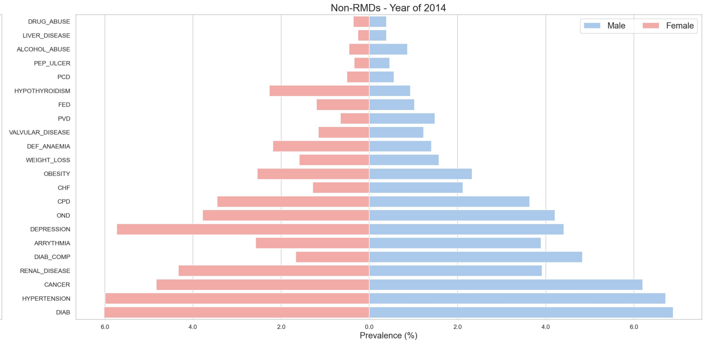

(j) Prevalence of Elixhuaser comorbidities patients with RMDs 2014

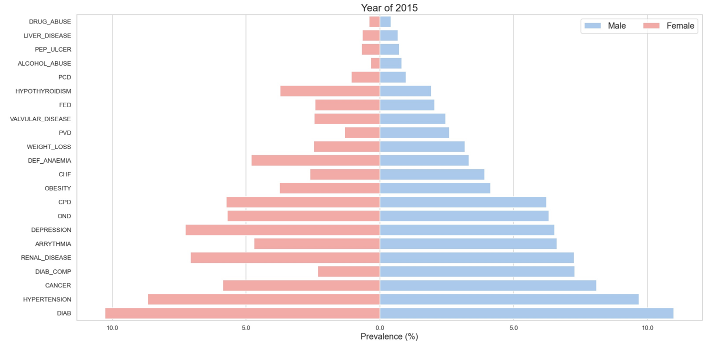

(k) Prevalence of Elixhuaser comorbidities patients without RMDs 2015

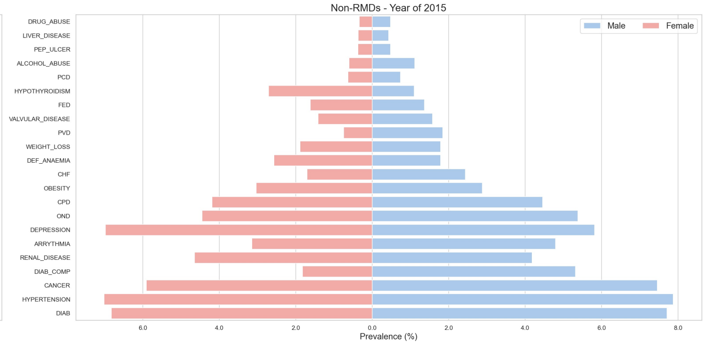

(l) Prevalence of Elixhuaser comorbidities patients with RMDs 2015

Figure 4. Prevalence of Elixhauser comorbidities in patients with RMDs and patients without RMDS in 2013 and 2015

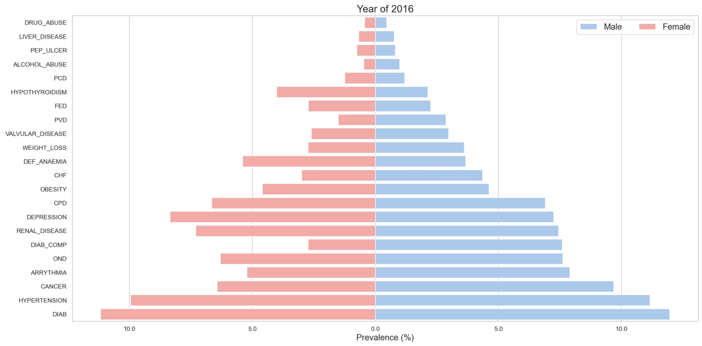

(m) Prevalence of Elixhuaser comorbidities patients with RMDs 2016

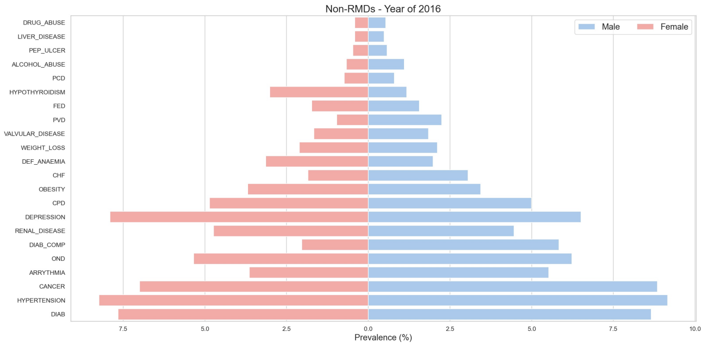

(n) Prevalence of Elixhuaser comorbidities patients with RMDs 2016

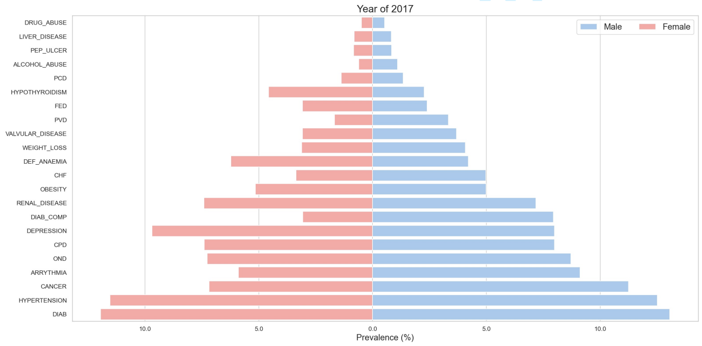

(o) Prevalence of Elixhuaser comorbidities patients without RMDs 2017

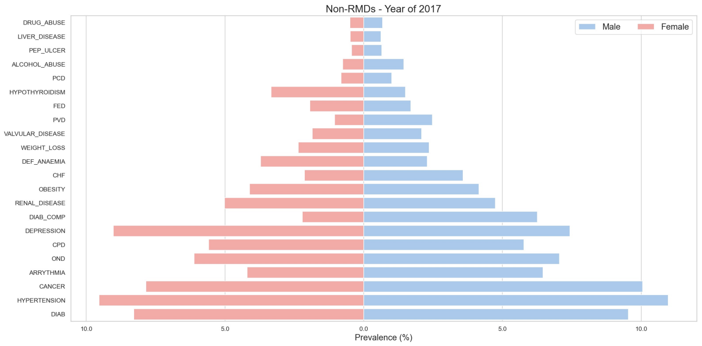

(p) Prevalence of Elixhuaser comorbidities patients with RMDs 2017

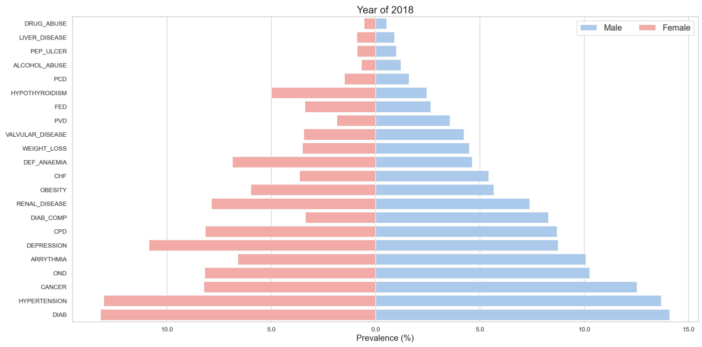

(q) Prevalence of Elixhuaser comorbidities patients without RMDs 2018

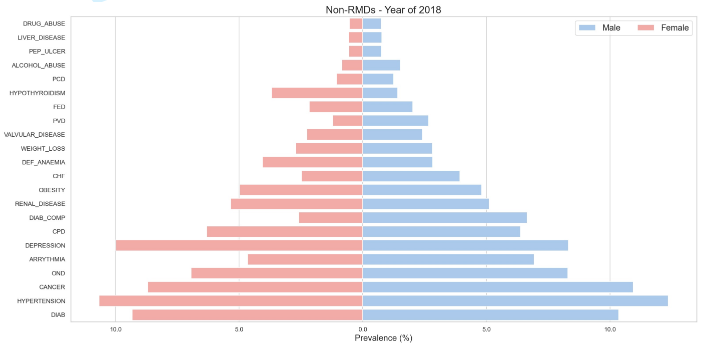

(r) Prevalence of Elixhuaser comorbidities patients with RMDs 2018

Figure 4. Prevalence of Elixhauser comorbidities in patients with RMDs and patients without RMDS in 2016 and 2018

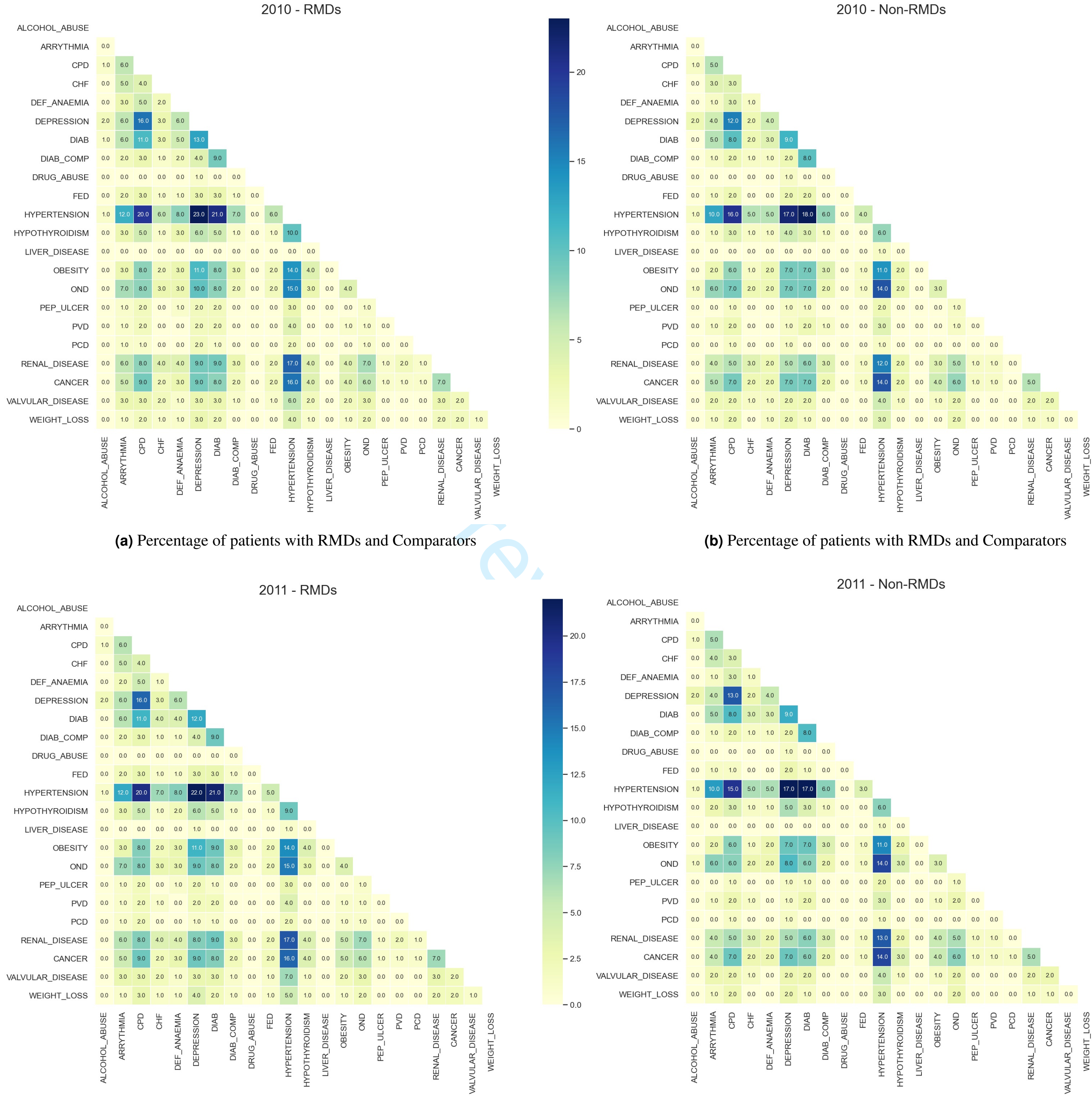

(a) Percentage of patients with RMDs and Comparators

(b) Percentage of patients with RMDs and Comparators

(c) Percentage of patients with RMDs and Comparators

(d) Percentage of patients with RMDs and Comparators

Figure 5. Proportion of patients with RMDs and Comparators

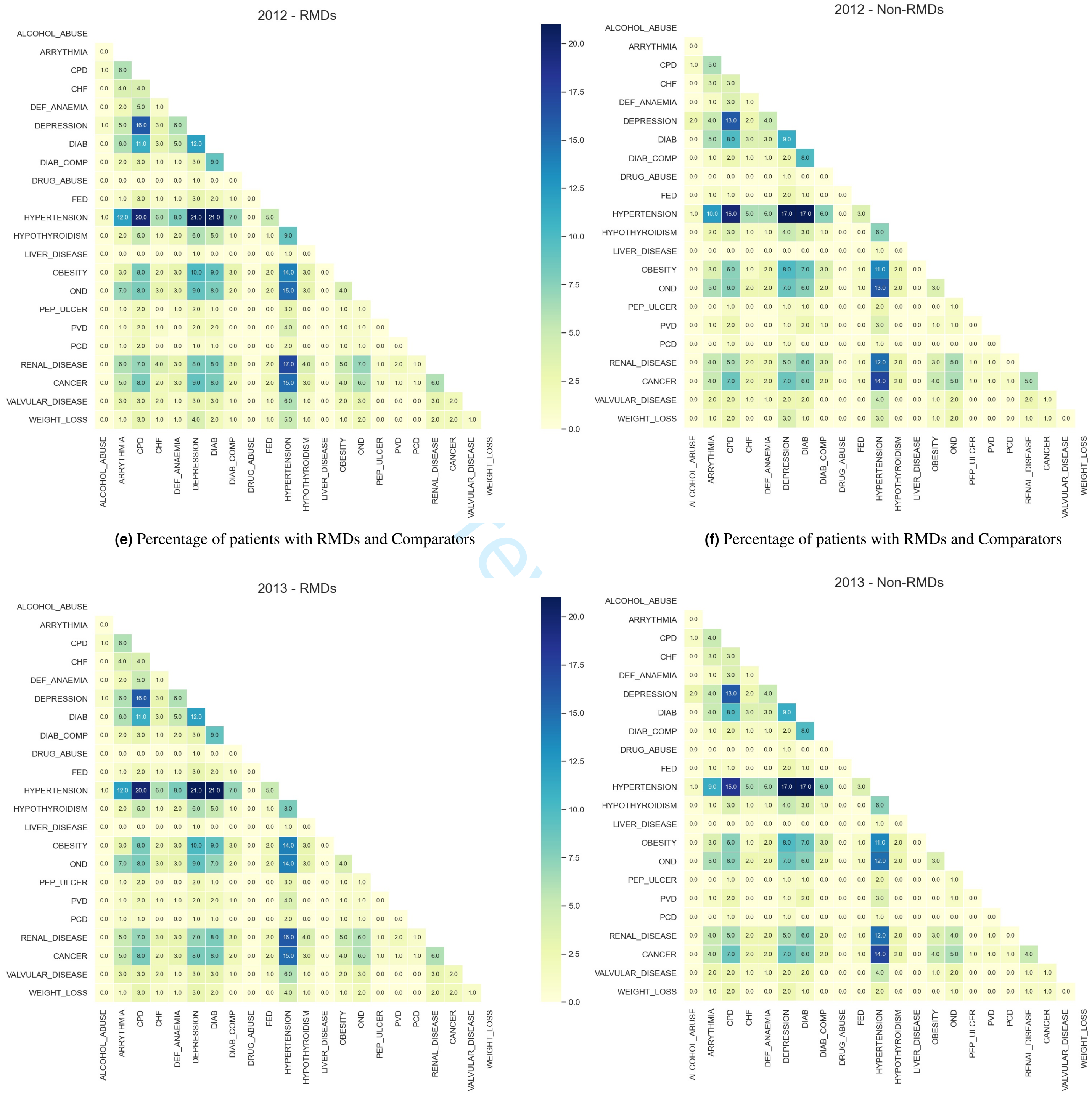

(e) Percentage of patients with RMDs and Comparators

(f) Percentage of patients with RMDs and Comparators

(g) Percentage of patients with RMDs and Comparators

(h) Percentage of patients with RMDs and Comparators

Figure 5. Proportion of patients with RMDs an Comparators

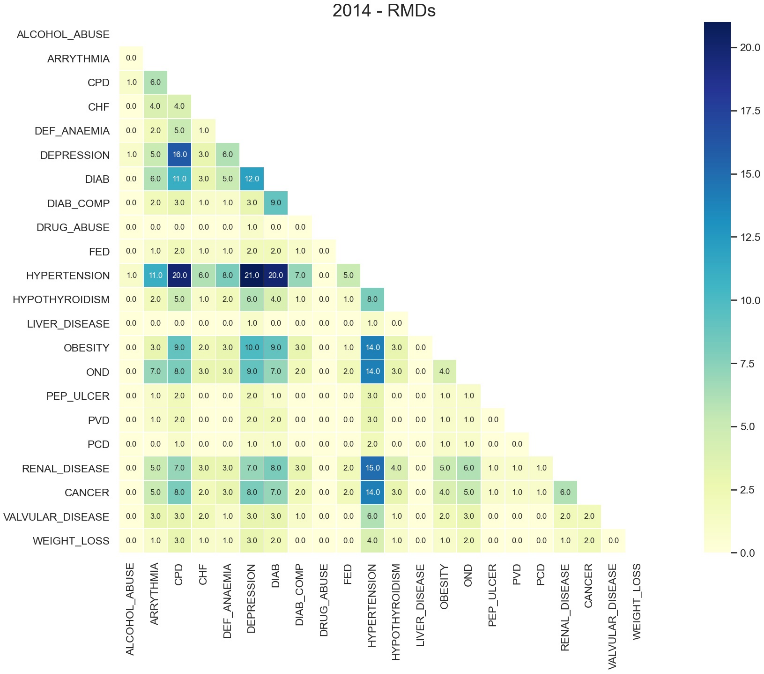

(i) Percentage of patients with RMDs and Comparators

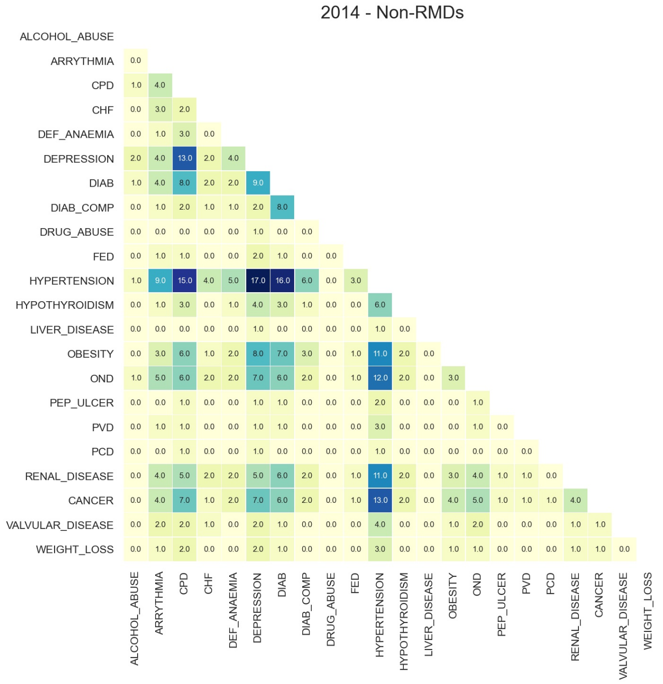

(j) Percentage of patients with RMDs and Comparators

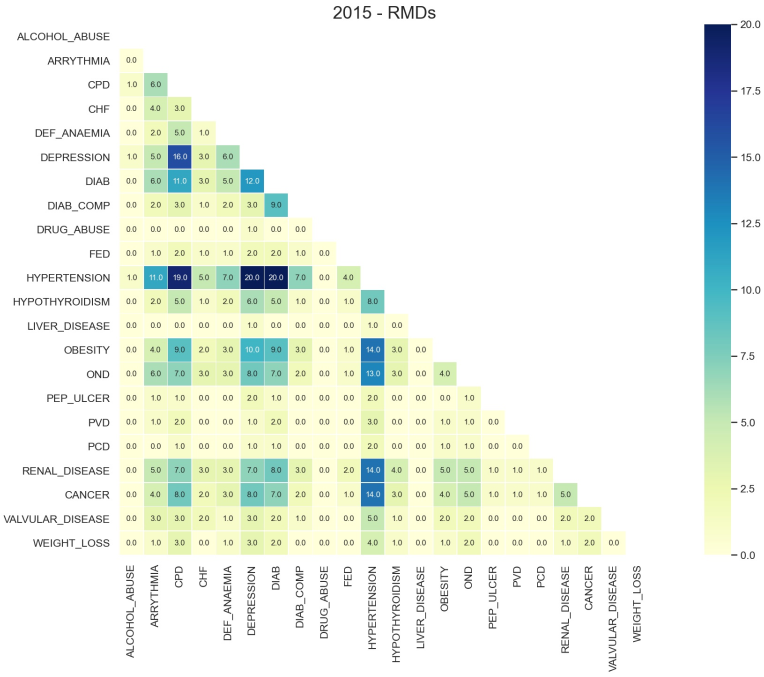

(k) Percentage of patients with RMDs and Comparators

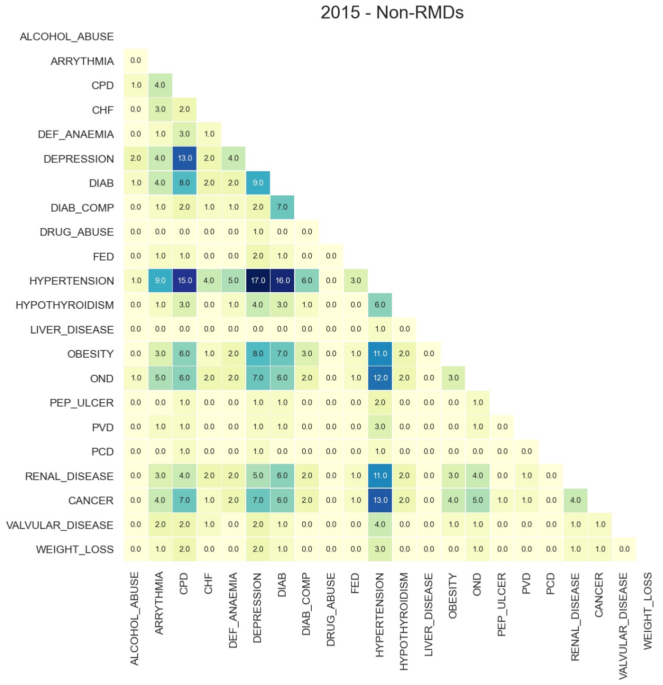

(l) Percentage of patients with RMDs and Comparators

Figure 5. Proportion of patients with RMDs an Comparators

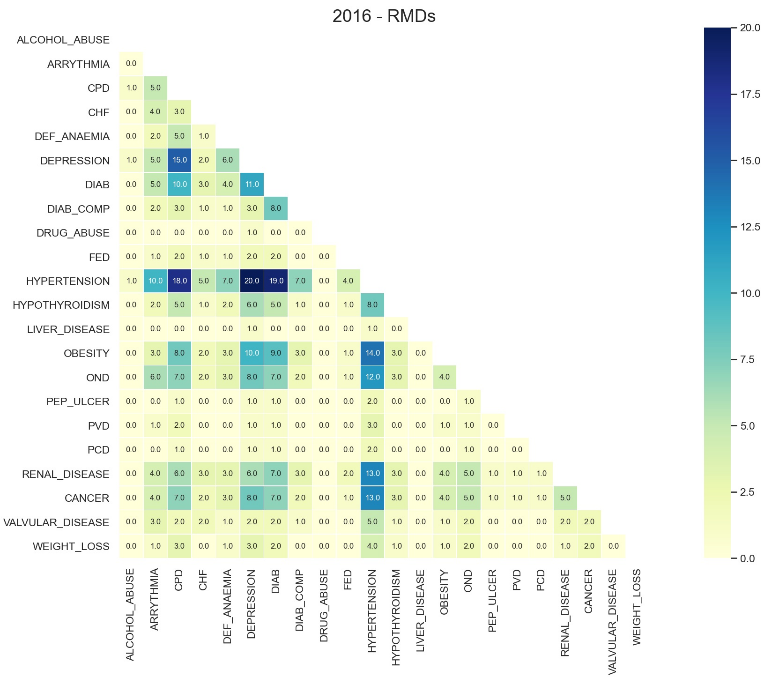

(m) Percentage of patients with RMDs and Comparators

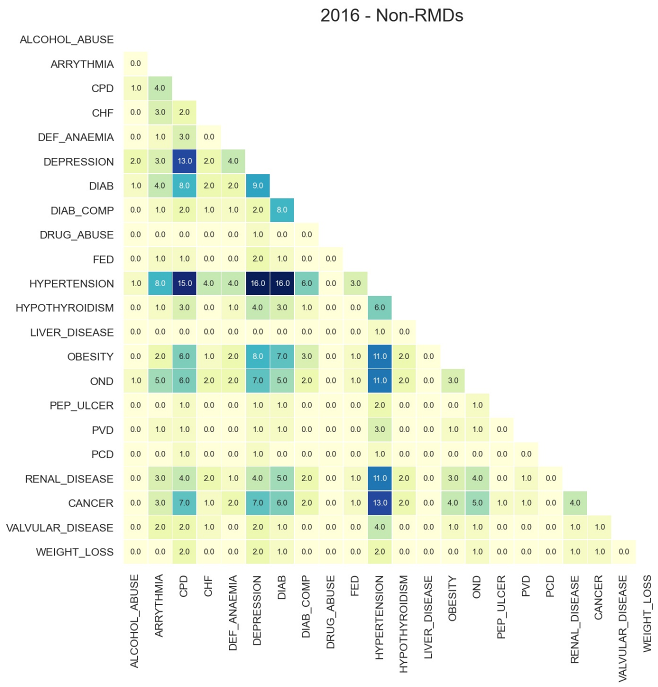

(n) Percentage of patients with RMDs and Comparators

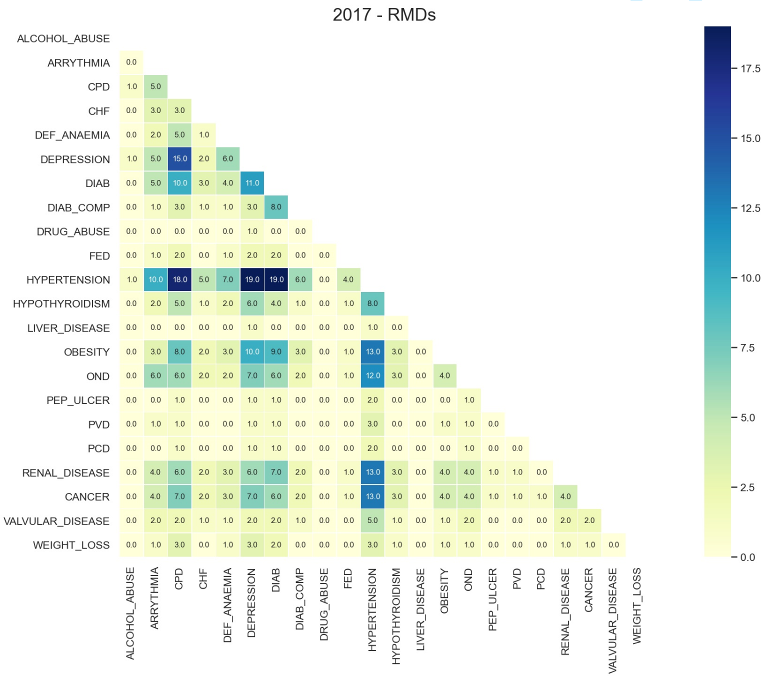

(o) Percentage of patients with RMDs and Comparators

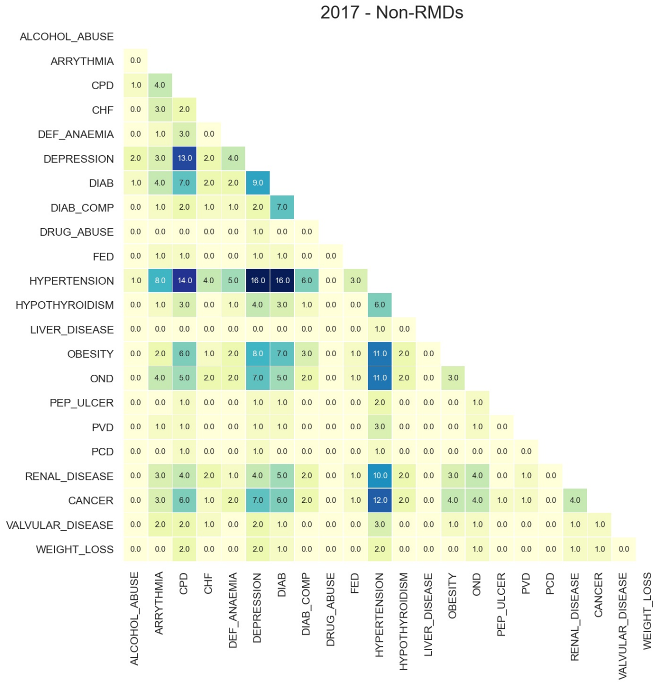

(p) Percentage of patients with RMDs and Comparators

Figure 5. Proportion of patients with RMDs and Comparators

For peer review only - http://bmjopen.bmj.com/site/about/guidelines.xhtml

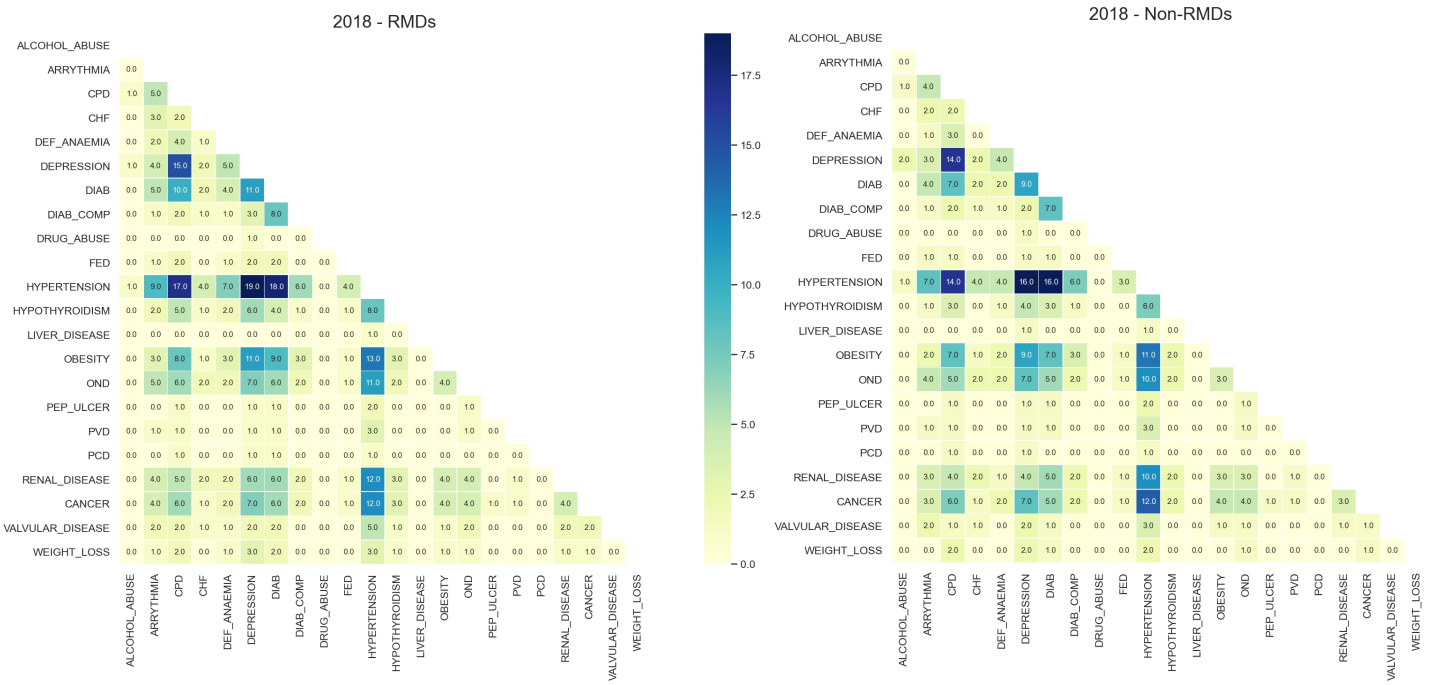

(q) Percentage of patients with RMDs and Comparators

(r) Percentage of patients with RMDs and Comparators

Figure 5. Proportion of patients with RMDs and Comparators

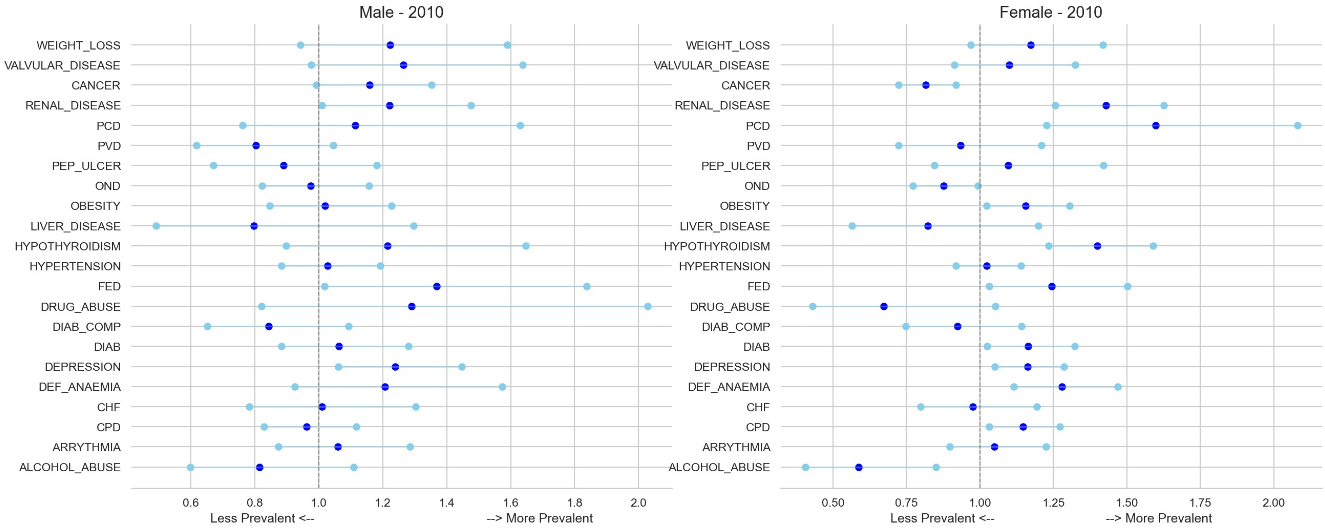

(a) ORs for association of RMDs with comorbidities by gender in 2010

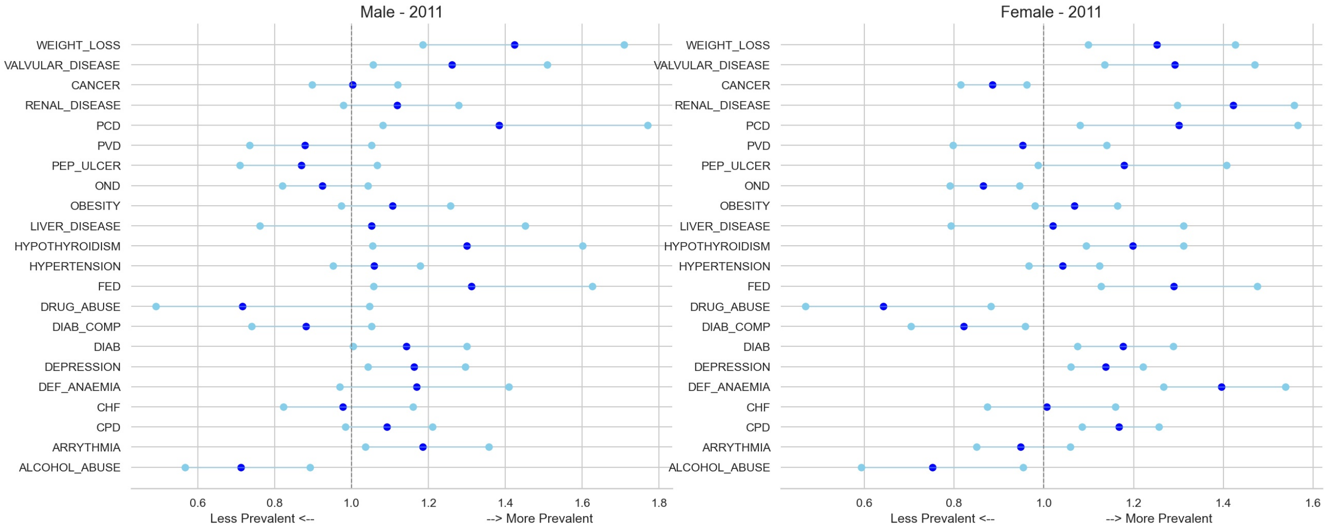

(b) Percentage of patients with RMDs and Comparators

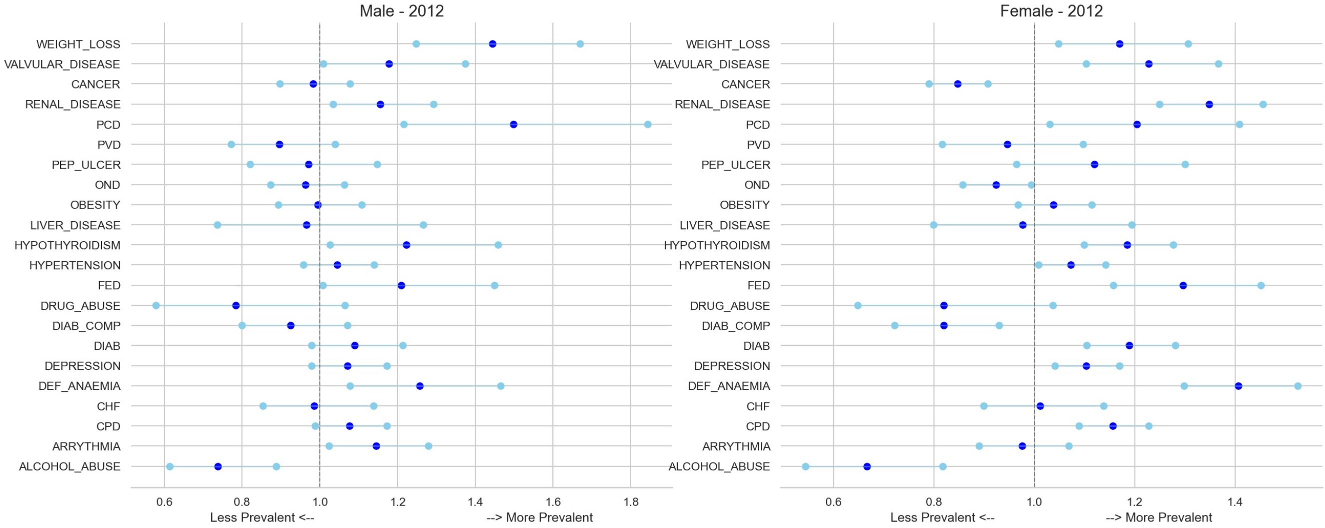

(c) Percentage of patients with RMDs an Comparators

Figure 5. ORs for association of RMDs with comorbidities by sex in 2012-2013

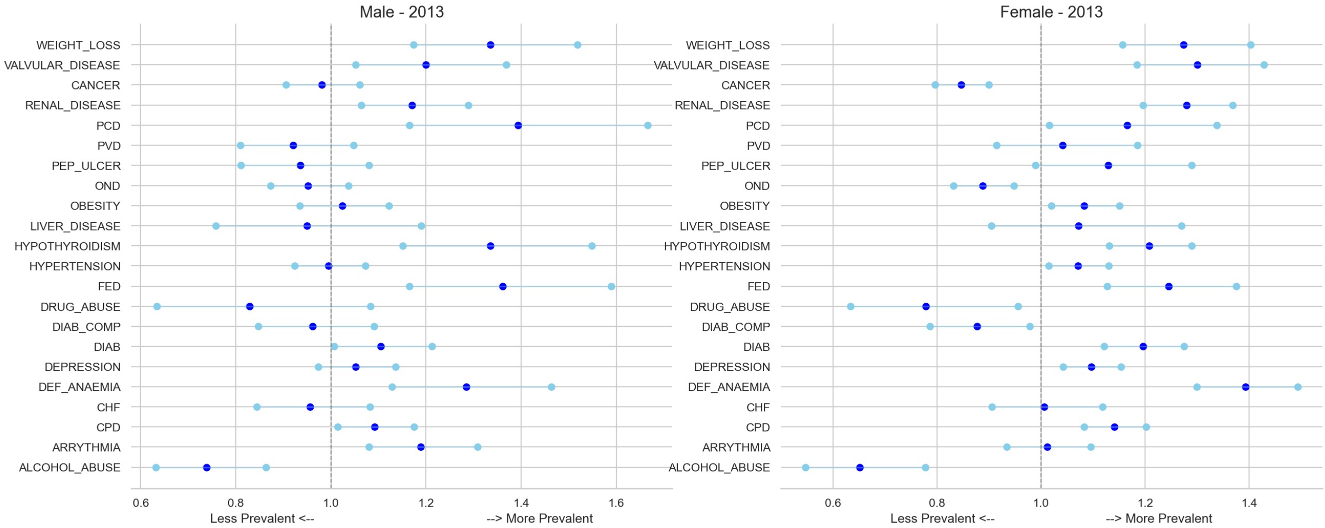

(d) Percentage of patients with RMDs and Comparators

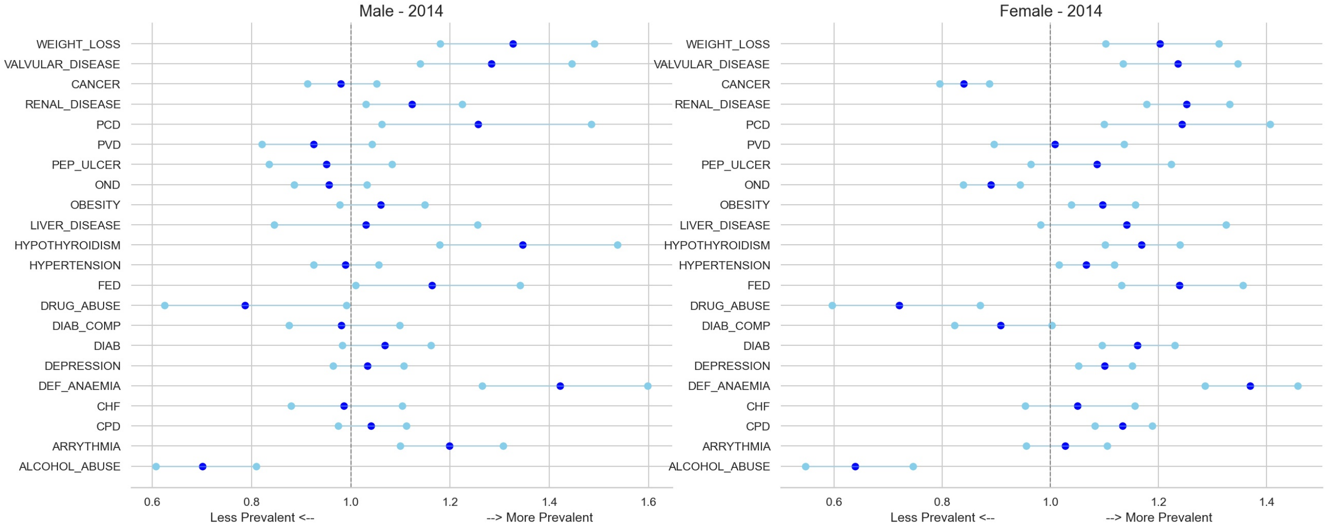

(e) Percentage of patients with RMDs an Comparators

Figure 5. ORs for association of RMDs with comorbidities by sex in 2013-2014

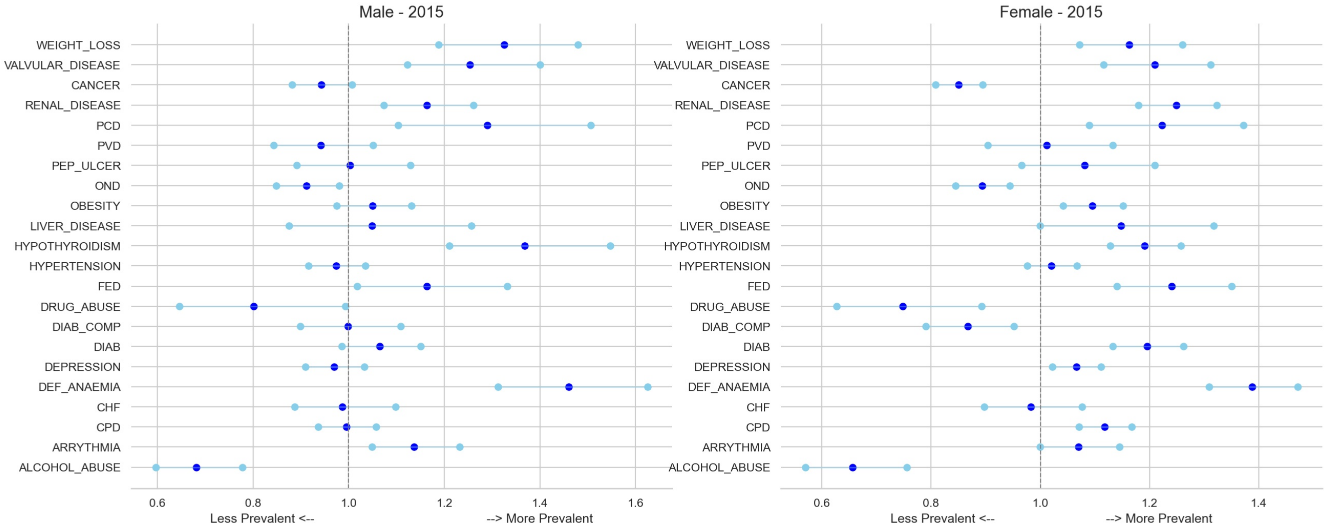

(f) Percentage of patients with RMDs and Comparators

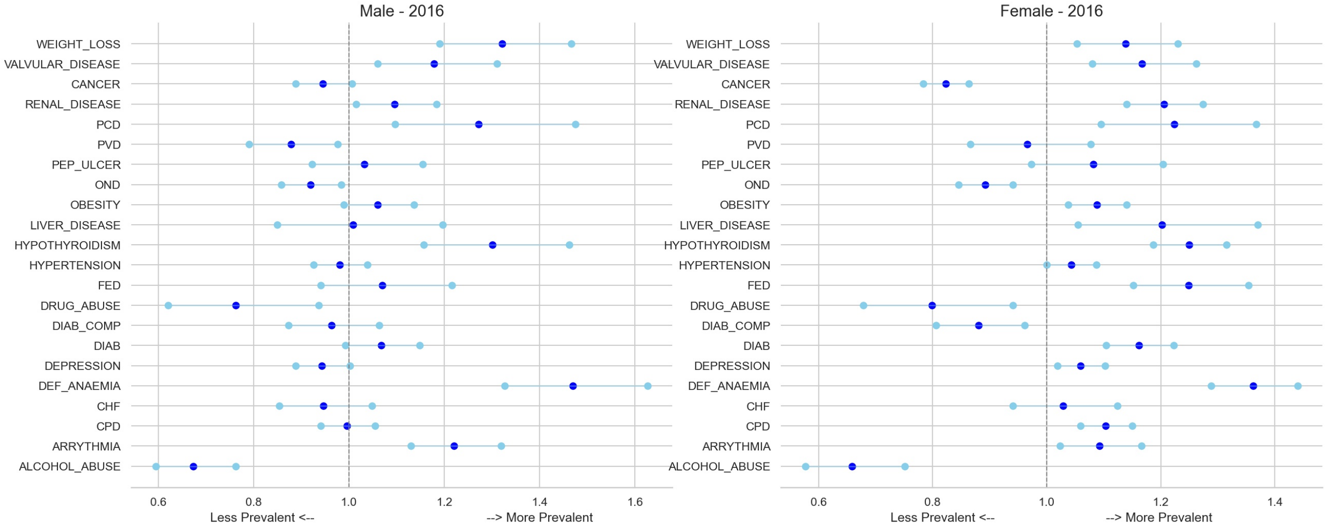

(g) Percentage of patients with RMDs an Comparators

Figure 5. ORs for association of RMDs with comorbidities by sex in 2015-2016

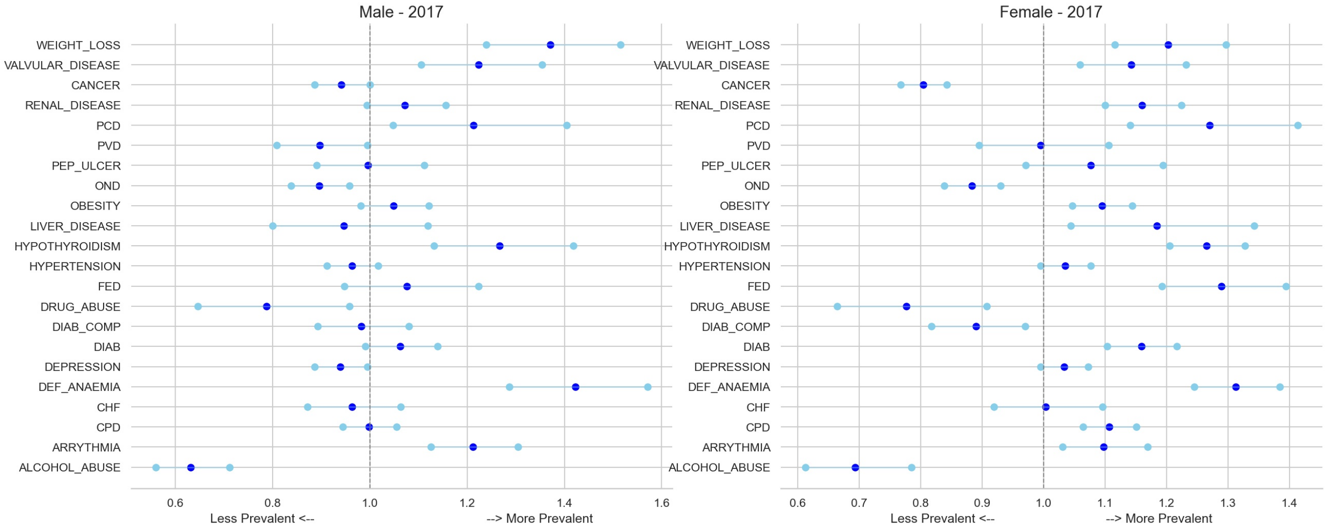

(h) Percentage of patients with RMDs and Comparators

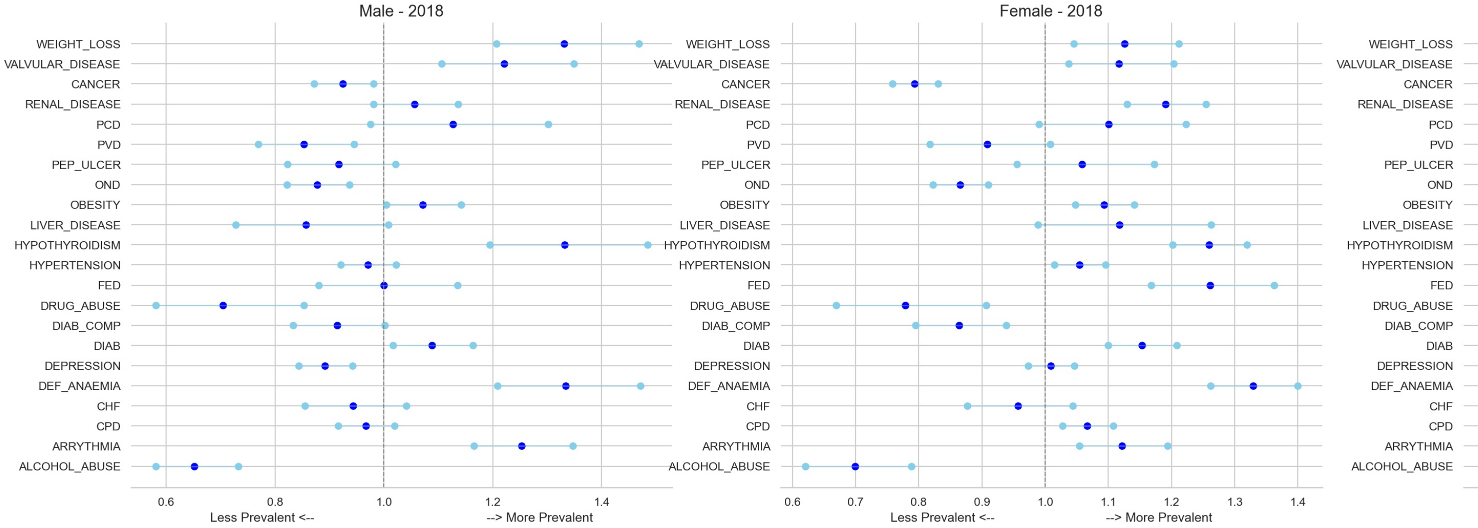

(i) Percentage of patients with RMDs and Comparators

Figure 5. ORs for association of RMDs with comorbidities by sex in 2017-2018

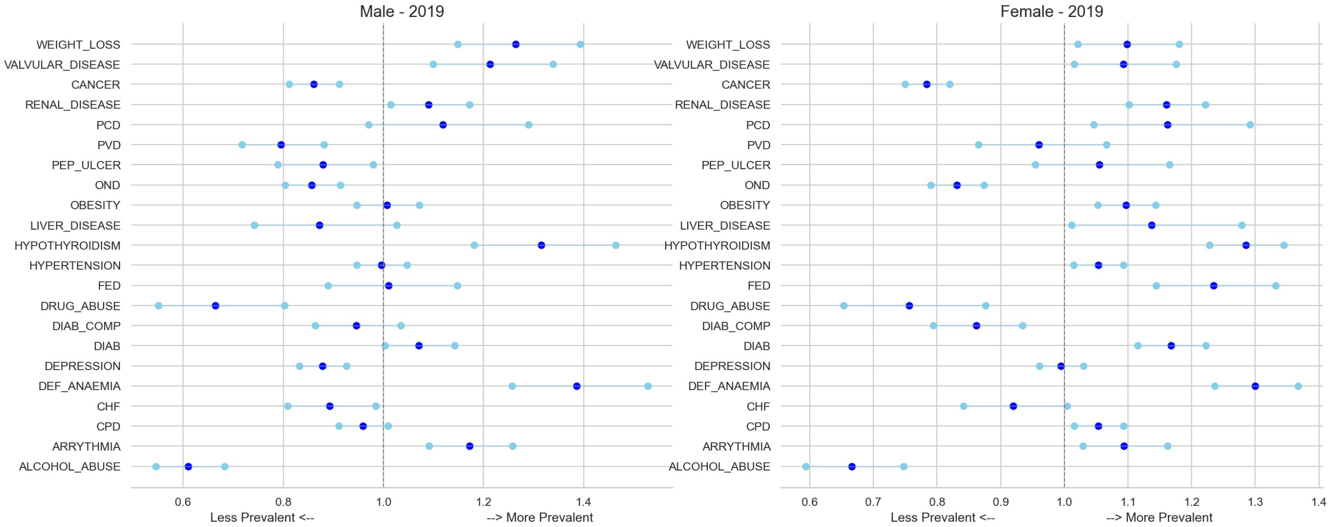

(k) Percentage of patients with RMDs and Comparators

Figure 5. ORs for association of RMDs with comorbidities by sex in 2019

For peer review only

Full description	abbreviations
Rheumatic and musculoskeletal diseases	RMDs
General Practices	GPs
Secure Anonymised Information Linkage	SAIL
Odds ratios	ORs
Trusted Research Environment	TRE
International Classification of Diseases 10th revision	ICD-10 codes
OPCS Classification of Interventions and Procedures version 4	(OPCS-4)
Multiple longterm conditions	MLTCs
Welsh Multi-morbidity e-Cohort	WMC
Alcohol abuse	ALCOHOL_ABUSE
Blood loss anemia	BL_ANAEMIA
Cardiac arrhythmia	CARD_ARRYTHMIA
Chronic pulmonary disease	CPD
Coagulopathy	COAGUL
Congestive heart failure	CHF
Deficiency anemia	DEF_ANAEMIA
Depression	DEPRESSION
Diabetes complicated	DIAB_COMB
Diabetes, uncomplicated	DIAB
Drug abuse	DRUG_ABUSE
FLuid and electrolyte disorders	FED
Hypertension	HYPERTENSION
Hypothyroidism	HYPOTHYROIDISM
Liver disease	LIVER_DIS
Lymphoma	LYMPHOMA
Metastatic cancer	MET_CANCER
Obesity	OBESITY
Other Neurological disorders	OND
Paralysis	PARALYSIS
Peptic ulcer disease excluding bleeding	PEPTIC_ULCER
Periheral vascular disorders	PVD
Psychoses	PSYCHOSES
Pulmonary circulation disorders	PCD
Renal failure	RENAL
Rheumatoid arthritis/collagen vascular diseases	RA
Solid tumor without metastasis	CANCER
Valvular disease	VALVULAR
Weight loss	WEIGHT_LOSS

Table 2. Abbreviations list

For peer review only

Reporting checklist for cohort study.

Based on the STROBE cohort guidelines.

Instructions to authors

Complete this checklist by entering the page numbers from your manuscript where readers will find each of the items listed below.

Your article may not currently address all the items on the checklist. Please modify your text to include the missing information. If you are certain that an item does not apply, please write "n/a" and provide a short explanation.

Upload your completed checklist as an extra file when you submit to a journal.

In your methods section, say that you used the STROBE cohortreporting guidelines, and cite them as:

von Elm E, Altman DG, Egger M, Pocock SJ, Gotzsche PC, Vandenbroucke JP. The Strengthening the Reporting of Observational Studies in Epidemiology (STROBE) Statement: guidelines for reporting observational studies.

		Reporting Item	Page Number
**Title and abstract**			
Title	#1a	Indicate the study's design with a commonly used term in the title or the abstract	1
Abstract	#1b	Provide in the abstract an informative and balanced summary of what was done and what was found	2
**Introduction**			
Background / rationale	#2	Explain the scientific background and rationale for the investigation being reported	4,5
Objectives	#3	State specific objectives, including any prespecified hypotheses	1
**Methods**			
Study design	#4	Present key elements of study design early in the paper	2,4

For peer review only

Setting	#5	Describe the setting, locations, and relevant dates, including periods of recruitment, exposure, follow-up, and data collection	2,4
Eligibility criteria	#6a	Give the eligibility criteria, and the sources and methods of selection of participants. Describe methods of follow-up.	2,4
Eligibility criteria	#6b	For matched studies, give matching criteria and number of exposed and unexposed	4
Variables	#7	Clearly define all outcomes, exposures, predictors, potential confounders, and effect modifiers. Give diagnostic criteria, if applicable	5
Data sources / measurement	#8	For each variable of interest give sources of data and details of methods of assessment (measurement). Describe comparability of assessment methods if there is more than one group. Give information separately for for exposed and unexposed groups if applicable.	2,4
Bias	#9	Describe any efforts to address potential sources of bias	n/a
Study size	#10	Explain how the study size was arrived at	2,4
Quantitative variables	#11	Explain how quantitative variables were handled in the analyses. If applicable, describe which groupings were chosen, and why	4
Statistical methods	#12a	Describe all statistical methods, including those used to control for confounding	5
Statistical methods	#12b	Describe any methods used to examine subgroups and interactions	n/a
Statistical methods	#12c	Explain how missing data were addressed	5
Statistical methods	#12d	If applicable, explain how loss to follow-up was addressed	5
Statistical methods	#12e	Describe any sensitivity analyses	n/a

Results

For peer review only

Participants	#13a	Report numbers of individuals at each stage of study—eg numbers potentially eligible, examined for eligibility, confirmed eligible, included in the study, completing follow-up, and analysed. Give information separately for for exposed and unexposed groups if applicable.	2,6
Participants	#13b	Give reasons for non-participation at each stage	n/a
Participants	#13c	Consider use of a flow diagram	7
Descriptive data	#14a	Give characteristics of study participants (eg demographic, clinical, social) and information on exposures and potential confounders. Give information separately for exposed and unexposed groups if applicable.	5
Descriptive data	#14b	Indicate number of participants with missing data for each variable of interest	n/a
Descriptive data	#14c	Summarise follow-up time (eg, average and total amount)	5
Outcome data	#15	Report numbers of outcome events or summary measures over time. Give information separately for exposed and unexposed groups if applicable.	4
Main results	#16a	Give unadjusted estimates and, if applicable, confounder-adjusted estimates and their precision (eg, 95% confidence interval). Make clear which confounders were adjusted for and why they were included	6
Main results	#16b	Report category boundaries when continuous variables were categorized	n/a
Main results	#16c	If relevant, consider translating estimates of relative risk into absolute risk for a meaningful time period	n/a
Other analyses	#17	Report other analyses done—eg analyses of subgroups and interactions, and sensitivity analyses	6
**Discussion**			
Key results	#18	Summarise key results with reference to study objectives	11
Limitations	#19	Discuss limitations of the study, taking into account sources of potential bias or imprecision. Discuss both direction and magnitude of any potential bias.	11,12

For peer review only

Interpretation	#20	Give a cautious overall interpretation considering objectives, limitations, multiplicity of analyses, results from similar studies, and other relevant evidence.	11,12,13
Generalisability	#21	Discuss the generalisability (external validity) of the study results	12
**Other Information**			
Funding	#22	Give the source of funding and the role of the funders for the present study and, if applicable, for the original study on which the present article is based	16

The STROBE checklist is distributed under the terms of the Creative Commons Attribution License CC-BY. This checklist was completed on 09. August 2023 using https://www.goodreports.org/, a tool made by the EQUATOR Network in collaboration with Penelope.ai

For peer review only
